# Towards the rational design of ylide-substituted phosphines for gold(i)-catalysis: from inactive to ppm-level catalysis†

**DOI:** 10.1039/d1sc00105a

**Published:** 2021-02-02

**Authors:** Jens Handelmann, Chatla Naga Babu, Henning Steinert, Christopher Schwarz, Thorsten Scherpf, Alexander Kroll, Viktoria H. Gessner

**Affiliations:** Faculty of Chemistry and Biochemistry, Chair of Inorganic Chemistry II, Ruhr University Bochum Universitätsstr. 150 44801 Bochum Germany viktoria.gessner@rub.de

## Abstract

The implementation of gold catalysis into large-scale processes suffers from the fact that most reactions still require high catalyst loadings to achieve efficient catalysis thus making upscaling impractical. Here, we report systematic studies on the impact of the substituent in the backbone of ylide-substituted phosphines (YPhos) on the catalytic activity in the hydroamination of alkynes, which allowed us to increase the catalyst performance by orders of magnitude. While electronic changes of the ligand properties by introduction of aryl groups with electron-withdrawing or electron-donating groups had surprisingly little impact on the activity of the gold complexes, the use of bulky aryl groups with *ortho*-substituents led to a remarkable boost in the catalyst activity. However, this catalyst improvement is not a result of an increased steric demand of the ligand towards the metal center, but due to steric protection of the reactive ylidic carbon centre in the ligand backbone. The gold complex of the thus designed mesityl-substituted YPhos ligand Y_Mes_PCy_2_, which is readily accessible in one step from a simple phosphonium salt, exhibited a high catalyst stability and allowed for turnover numbers up to 20 000 in the hydroamination of a series of different alkynes and amines. Furthermore, the catalyst was also active in more challenging reactions including enyne cyclisation and the formation of 1,2-dihydroquinolines.

## Abstract 2

Modification of the backbone in ylide-substituted phosphines allowed a remarkable boost in the catalytic activity, thus enabling a series of gold catalyzed transformations at very low catalyst loadings.

## Graphical abstract

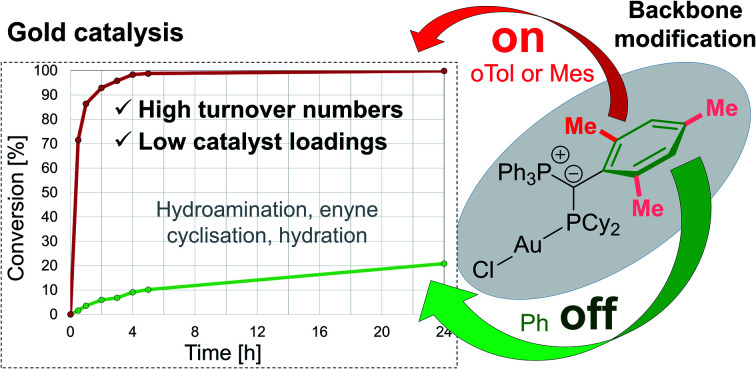

## Introduction

Phosphine ligands are a privileged class of ligands in homogenous catalysis.^1^ Their continuous development and tailoring has led to many important advances and decisively contributed to the development of homogenous catalysis into a mature field in chemistry. Despite the advances made in the past years, the design of new ligands is still needed to improve existing reaction protocols and to enable new transformations. Notable examples for the importance of the design of phosphine ligands for the improvement of the catalyst performance are achievements made in hydrogenations,^2^ C–C and C–X couplings,^3^ as well as gold(i) catalysed reactions.^4^

In gold catalysis, ligand design is particularly important for the generation of highly productive catalysts that can operate at low catalysts loadings. However, despite the costs of the metal, most catalytic transformations with gold still require loadings of more than 0.5 mol% of catalyst to achieve high conversions, thus making large-scale applications impractical. In the past years, several groups have addressed this issue. Thus, a number of ligands have been reported which enable conversions at remarkably low loadings, in some cases also on ppm-level. For example, Zhang and coworkers described the design of an electron-rich biarylphosphine ligand, which due to additional substitution at the biaryl moiety enabled a ligand-directed nucleophilic attack of the alkyne and thus turnover numbers of 99 000 for the addition of acids to alkynes.^5,6^ A similar strategy of improving the catalyst activity by modification of the catalytic pocket was recently applied to N-heterocyclic carbenes (NHCs)^7^ as well as further dialkylbiaryl phosphines.^8^ In case of hydroamination reactions, the best results however, were – to the best of our knowledge – reported by Lavallo and coworkers using an anionic phosphine and NHC with carborate substituents, giving rise to TONs close to 100 000.^9^ Drawback of these highly sophisticated ligands however, is that they are often rather complicated to synthesize thus considerably contributing to the total costs of the catalyst.

**Fig. 1:**
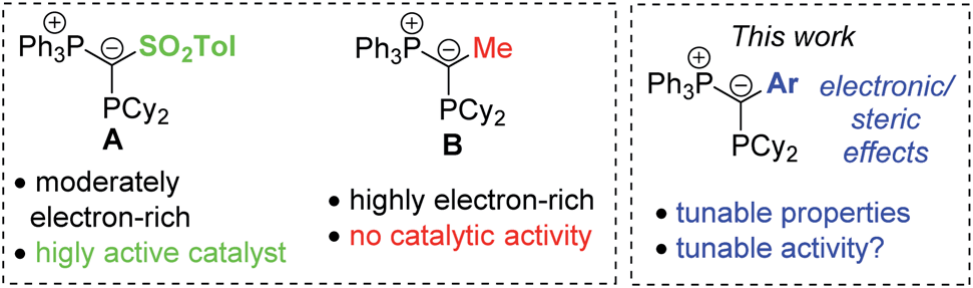
(Left) Gold complexes based on ylide-substituted phosphines (YPhos) and comparison of different YPhos ligands and their activity in catalysis.

Recently, we reported on the class of ylide-substituted phosphines (YPhos) as easy-to-synthesize and highly efficient ligands in catalysis.^10^ Due to the electron-donation from the ylide to the phosphorus centre YPhos ligands are in general strong donor ligands with donor strengths comparable to those of N-heterocyclic carbenes (NHC). Accordingly, high activities were observed with YPhos-ligated complexes in Au-catalysed hydroaminations^10*a*,11^ as well as Pd-catalysed coupling^12^ and α-arylation reactions.^13^ In case of gold-catalysed hydroaminations we observed that YPhos ligands such as **A** (Fig. 1) with electron-withdrawing groups in the backbone (–SO_2_Tol or –CN) and only moderate donor strengths gave the most active catalysts, while the most electron-rich donors (*e.g.***B**) showed no activity at all. This is surprising since the rate-limiting protodeauration step of the hydroamination is facilitated by electron-rich ligands.^14^ Hence, strong donors ligands typically showed the highest activities.^5–9,15^

To understand the inactivity of the most electron-rich YPhos ligands and to develop principles for future ligand design, we turned our attention towards ligands with aryl substituents in the ylide-backbone. This decision was based on two considerations: (i) the ylidic carbon atom in the electron-rich ligands is highly reactive and thus prone to side-reactions (protonation/auration). Stabilization of the carbanionic charge might be crucial and thus aryl groups should be suitable substituents due to the possible charge delocalization into the aromatic moiety. (ii) Aryl groups can be tuned electronically and sterically and thus allow for a detailed study of the different factors influencing the catalytic ability of the gold complexes.

## Results and discussion

### Ligand synthesis and properties

**Scheme 1:**
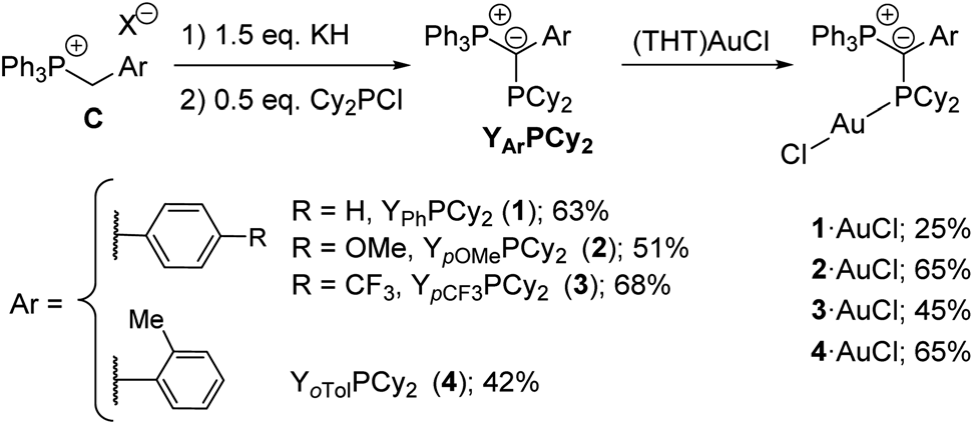
Preparation of the YPhos ligands **1–4**.

**Fig. 2:**
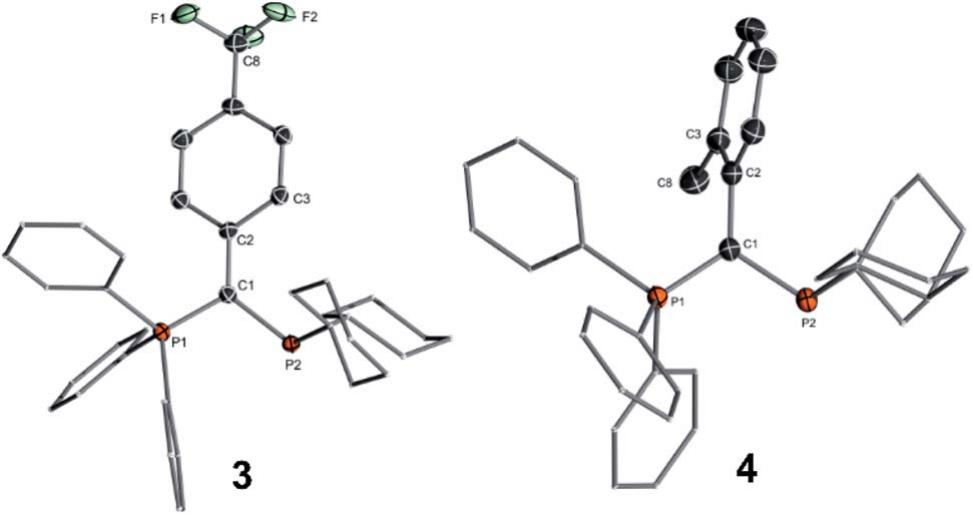
Molecular structures of ligands **3** and **4**. Ellipsoids are drawn at the 50% probability level. Structures of the other ligands are given in the ESI,† important parameters are listed in Table 1.

We started our studies by preparing a series of YPhos ligands of type Y_Ar_PCy_2_ (Scheme 1). In this ligand structure, electron-donating (Y_*p*OMe_, **2**) and electron-withdrawing (Y_*p*CF_3__PCy_2_, **3**) as well as sterically demanding groups (Y_*o*Tol_PCy_2_, **4**) were compared with the parent phenyl compound Y_Ph_PCy_2_ (**1**). All ligands were prepared in one step from simple phosphonium salts **C** (prepared from PPh_3_ and the corresponding benzyl halides)^10^ using a slight excess of base (KH) and half an equiv. Cy_2_PCl. Thus, ligands **1–4** were obtained as yellow solids in moderate to good yields of 42–68%. The YPhos ligands are characterized by two sets of doublets in the ^31^P{^1^H} NMR spectrum. While these signals appear in a similar region for the ligands **1–3** (Table 1), they are clearly shifted for the *ortho*-tolyl-substituted compound. Likewise, the ^2^*J*_PP_ coupling constant of **4** (^2^*J*_PP_ = 160.4 Hz) considerably differs from those of the other ligands (^2^*J*_PP_ = 182.4–185.0 Hz) thus indicating differences in the geometry of **4** compared to the other ligands. This is confirmed by the crystal structures of the compounds (Fig. 2b). The most striking difference between **4** and **1–3** concerns the orientation of the aryl group relative to the P–C–P plane. While the ligands **1–3** exhibit small P–C–C–C torsion angles between 8.0 and 21.7° and thus an almost co-planar arrangement of the P–C–P and the aryl moiety, the aryl group in **4** is almost perpendicularly arranged (P–C–C–C = 75.5(3)°). Hence, no delocalization of the carbanionic charge is possible in case of the tolyl system. This also results in the shortest P1–C1 bond in **4** for all ligands due to the strongest electrostatic interactions in the ylide linkage.

**Table 1:** ^31^P{^1^H} NMR spectroscopic and crystallographic data for ligands **1–4**

	Y_Ph_PCy_2_ (**1**)	Y_*p*OMe_PCy_2_ (**2**)	Y_*p*CF_3__PCy_2_ (**3**)	Y_*o*Tol_PCy_2_ (**4**)	Y_Mes_PCy2 (**5**)
*δ*_P_(PPh_3_) [ppm]	19.8	18.8	21.2	13.9	9.9
*δ*_P_(PCy_2_) [ppm]	−5.3	−5.0	−5.1	−1.3	7.2
^2^ *J*_PP_ [Hz]	185.0	182.4	184.3	160.4	170.3
P1–C1 [Å]	1.721(2)	1.721(3)	1.726(2)	1.696(2)	1.700(2)
P1–C1–P2 [°]	115.1(1)	113.2(2)	112.5(1)	113.4(1)	111.9(1)
P1–C1–C2–C3 [°]	21.7(1)	8.0(4)	17.2(2)	75.5(3)	87.2(1)

**Fig. 3:**
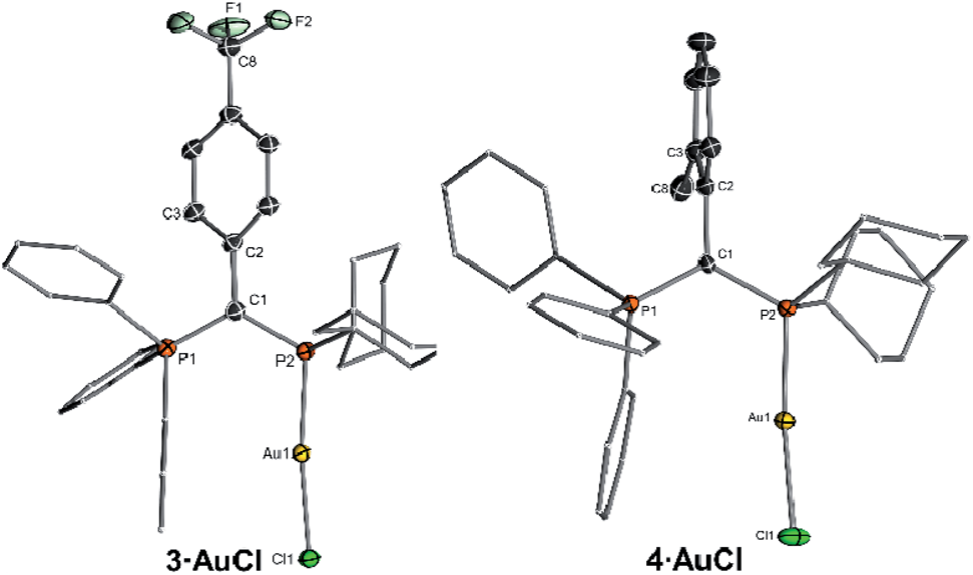
Molecular structures of the gold(i) complexes of the ligands **3** and **4**.

To determine the steric properties of the ligands the buried volume (%*V*_bur_) was calculated based on the structures of the isolated LAuCl complexes,^16^ which were synthesized from the free ligands and (THT)AuCl (Scheme 1) and isolated as off-white solids (Fig. 3). All four ligands exhibit similar spatial properties and approx. cover half of the sphere around the metal atom. %*V*_bur_ varies only slightly within the ligand series thus adopting values between 47.0 and 49.9% (Table 2). The same holds true for the calculated Tolman cone angles,^17^ thus confirming the bulkiness of the ligands, but also indicating that catalytic differences due to varying spatial protection of the metal by the ligands should be minimal.

**Table 2:** Comparison of the electronic and steric properties of the YPhos ligands **1–4**

Ligand	*ν*_Rh_ [cm^−1^]	TEP^a^ [cm^−1^]	%*V*_bur_^b^	Cone angle [°]^c^	P1–C1–C2–C3 in L·AuCl [°]
Y_Ph_PCy_2_ (**1**)	1948.9	2052.5	48.3	194.5	50.4(1)
Y_*p*OMe_PCy_2_ (**2**)	1947.2	2051.5	47.0	197.2	71.2(1)
Y_*p*CF_3__PCy_2_ (**3**)	1952.6	2054.6	49.9	204.1	36.6(1)
Y_*o*Tol_PCy_2_ (**4**)	1947.5	2051.7	48.9	199.4	81.7(3)
Y_Mes_PCy_2_ (**5**)	n.d.^d^	n.d.	50.4	194.7	85.3(1)

a TEPs were determined by *ν*_CO_ in the Rh(acac)(CO)(L) complexes using the linear relationship between *ν*_CO_ for Ni(CO)_3_(L) and Rh(acac)(CO)(L) reported in ref. 19.

b Calculated with the SambVca 2.1 program for the LAuCl complexes; M–P distance = 2.28 Å, including H atoms.^20^

c Calculated from the crystal structure according to the method described by Müller and Mingos, see ref. 21.

d The complex eliminates both CO molecules and therefore no stretching frequency could be recorded.

It must be noted that all ligands feature a similar geometry in the gold complexes. As such, the PPh_3_ moiety always points towards the gold centre, suggesting that the ligand should be able to stabilize a cationic gold species by additional arene–gold interactions. This was also observed for other YPhos gold complexes^10,11^ and has become a more general design principle for ligands in gold catalysis including phosphines and N-heterocyclic carbenes.^7,18^

Interestingly, the aryl substituents in the ylide-backbone of all LAuCl complexes rotate out of the P–C–P plane. This contrasts with the free ligands where only the *ortho*-tolyl ligand **4** showed this behaviour. Presumably, this rotation of the aryl group is enforced by steric effects. Upon coordination of gold the cyclohexyl groups adjust their orientation relative to each other and to the metal, thus forcing the aryl unit out of the P–C–P plane. This can nicely be seen by comparison of the structures of the free ligands (Fig. 2) and their gold complexes (Fig. 3). The steric pressure on the aryl substituent is further amplified by the widening of the P–C–P angle upon metal coordination. As such, P–C–P angles larger than 120° are found in the gold complexes, while they range between 112.5(1) and 115.1(1)° in the free ligands. Since the rearrangement of the ligand structure already occurs upon coordination of the small, linear AuCl fragment, it can be assumed that this will also be the case for any other metal fragment binding to the YPhos ligands. Hence, no π-delocalization of the carbanionic charge into the aryl substituents should be possible in the metal complexes, so that only inductive effects should play a role in the ligand donor strength. Accordingly, the Tolman electronic parameters (TEP) of all ligands are in the same range and reflect the different inductive effects of the substituents at the aryl groups. Thus, the donor strength follows the order: Y_CF_3__PCy_2_ < Y_Ph_PCy_2_ < Y_*p*OMe_PCy_2_ ≈ Y_oTol_PCy_2_. Y_oTol_PCy_2_ and Y_*p*OMe_PCy_2_ exhibit similar donor strengths and are thus similarly strong donors than the YPhos ligand **B** with a methyl group in the backbone (Fig. 1).

### Comparison of the catalytic performance

**Fig. 4:**
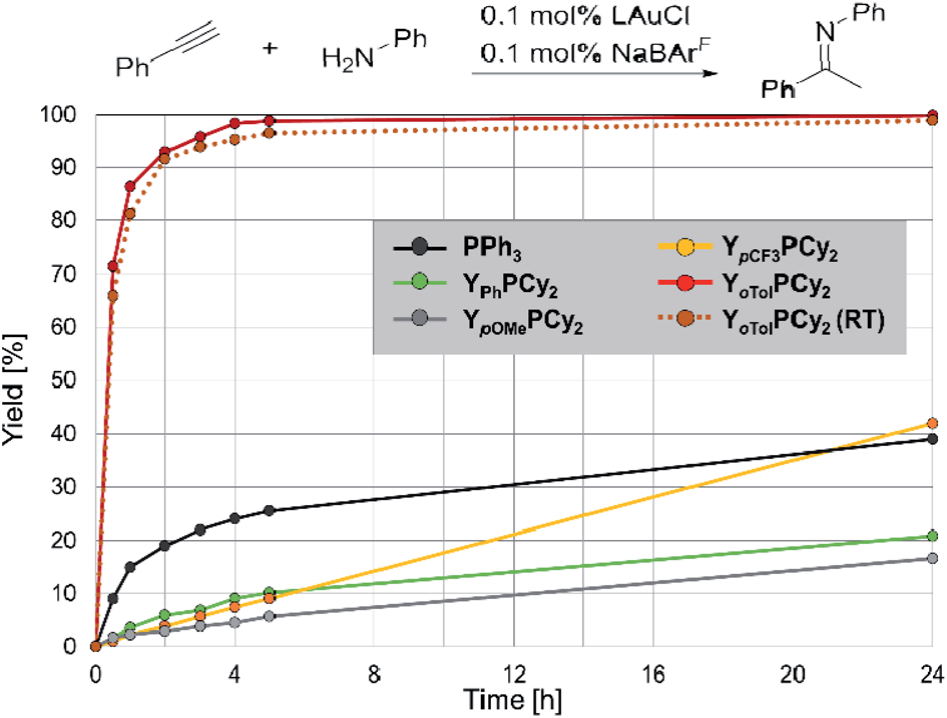
Comparison of the catalytic efficiency of the gold complexes of **L1–L4** in catalysis. Reaction conditions: aniline (1 eq.), phenylacetylene (1 eq.), LAuCl : NaBAr^F^ = 1 : 1, 50 °C (if not stated otherwise). Conversion was determined by NMR spectroscopy.

To examine the impact of the different aryl substituents on the activity of the complexes in gold(i) catalysed hydroaminations we chose the reaction of phenylacetylene with aniline as test reaction, since many other ligands have been tested in this transformation, thus allowing for a more detailed comparison.^9–20,22^ The reactions were conducted at mild conditions (50 °C) with low catalyst loadings of only 0.1 mol% LAuCl and NaBAr^F^_4_ for halide abstraction. Surprisingly, only small changes were observed with the ligands **1–3** (Fig. 4), suggesting that electronic changes in the aryl group have only little impact on the catalytic activity. Y_Ph_PCy_2_ and its methoxy analogue gave even lower yields than simple PPh_3_ after 24 h. Y_*p*CF_3__PCy_2_ also delivered only approx. 40% conversion. However, the almost linear reaction profile with **3** suggests a higher stability (but low activity) of the catalytically active species, which we attributed to the better stabilization of the negative charge by the *p*-CF_3_C_6_H_4_ group. This corroborates with previous findings which showed that only YPhos ligands with highly electron-withdrawing groups (–SO_2_Tol and –CN) in the ylide-backbone exhibit high activities in gold catalysis (see above).

To our delight, introduction of the *ortho*-tolyl moiety resulted in a surprisingly strong increase of activity. Full conversion to the imine was already reached after 4 h reaction time. Fortunately, **4** was also active at RT and gave almost full conversion after 5 h. This superior activity of ligand **4** clearly demonstrates that changes of the steric profile of the aryl group have a larger impact on the activity than electronic effects (note that **2** and **4** are similarly strong donors according to their TEP, Table 2). However, it is not the spatial influence that the ligand passes onto the metal which is decisive here (note that %*V*_bur_ only marginally changes in whole ligand series), but the steric protection of the carbanionic centre in the ylide-backbone. As suggested by the previously observed inactivity of the most electron-rich YPhos ligands,^10*a*^ the ylidic centre might be susceptible to protonation reactions or migration of the metal from the phosphine donor to the ylidic carbon atom, which might lead to a complete shutdown of the catalytic ability. Steric protection of the carbanionic carbon centre seems to suppress these side-reactions and thus improves the catalyst stability. This observation represents an important finding for further ligand design. Whereas electronic stabilization of the ylide is effective for stabilizing the catalytically active gold species, it also limits the tunability of the electronic properties since the electron-withdrawing group ultimately leads to a decrease of the ligand donor strength. This can be circumvented by the steric protection of the ylide moiety, which also allows the generation of ligands with stronger donor properties.

It is noteworthy that besides the steric protection of the ylidic carbon atom also the increased rigidity of the ligand structure caused by the introduction of the *ortho*-methyl group might play a role in the catalytic activity. Earlier studies have shown that the P–C–P angle in the ligand structure varies strongly depending on changes at the metal centre. This flexibility is limited by bulky groups in the ligand backbone thus resulting in more compact/rigid structures. This ultimately leads to a closer contact between the phenyl group of the PPh_3_ moiety and the gold centre and hence to stronger secondary ligand metal interactions. Quantum theory of atoms-in-molecules (QTAIM) calculations confirmed the presence of weak arene–gold interactions in all complexes by the detection of a bond critical point (Table 3, see ESI† for details). This complexation behaviour is similar to well established biaryl phosphines. Interestingly, the highest electron density at the bond critical point was found for Y_Tol_, thus suggesting the strongest stabilization in this complex.^23^

**Table 3:** Comparison of the arene gold interaction in the gold complexes of the YPhos ligands **1–5**; calculated values^a^, bond lengths in [Å]

Ligand	Au–C (*ipso*/*ortho*/sum)	*ρ*(BCP)	
Y_Ph_PCy_2_·AuCl	3.234/3.244/6.478	0.0138	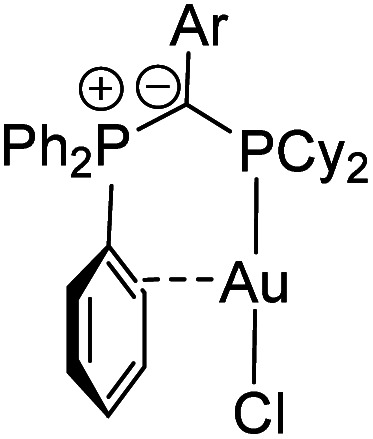
Y_*p*OMe_PCy_2_·AuCl	3.195/3.297/6.492	0.0137	
Y_*p*CF_3__PCy_2_·AuCl	3.361/3.369/6.730	0.0113	
Y_*o*Tol_PCy_2_·AuCl	3.207/3.130/6.337	0.0153	
Y_Mes_PCy_2_·AuCl	3.241/3.106/6.347	0.0153	

a Level of theory: PW6B95D3/def2tzvp (MWB60 for Au).

Motivated by the excellent performance of **4** we envisioned that a further steric protection of the ylidic carbon atom could further improve the catalyst performance. Thus, the mesityl analogue **5** (Mes = 2,4,6-trimethylphenyl), in which the carbanionic centre is protected from both sides of the P–C–P plane, was targeted.

### Ligand optimization

**Scheme 2:**
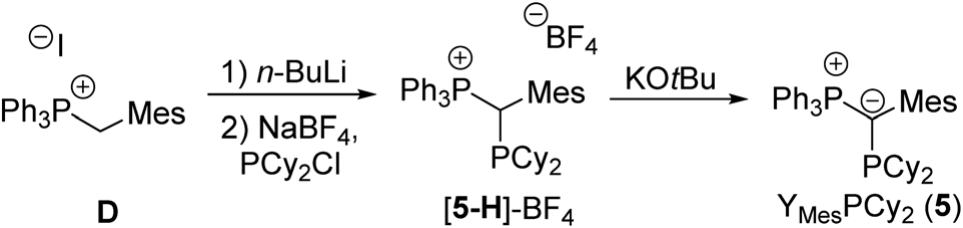
Preparation of the mesityl-substituted YPhos ligand **5** (Mes = 2,4,6-trimethylphenyl).

The synthesis of Y_Mes_PCy_2_ (**5**) was attempted *via* the same strategy as used for **1–4** (Scheme 2). However, phosphorylation of the phosphonium salt **D** turned out to be less straight-forward. Treatment of **D** with *n*-BuLi and Cy_2_PCl led to an equilibrium between the ylide and the phosphonium salt **5-H**, which due to steric hinderance was largely on the reactant side (Scheme 2). This problem was bypassed by addition of NaBF_4_ to remove the chloride from the equilibrium (precipitation of NaCl) thus preventing the reformation of Cy_2_PCl. Deprotonation finally yielded Y_Mes_PCy_2_, which was isolated as yellow solid in 49% yield in a one-pot reaction starting from the phosphonium salt **D**.

**Fig. 5:**
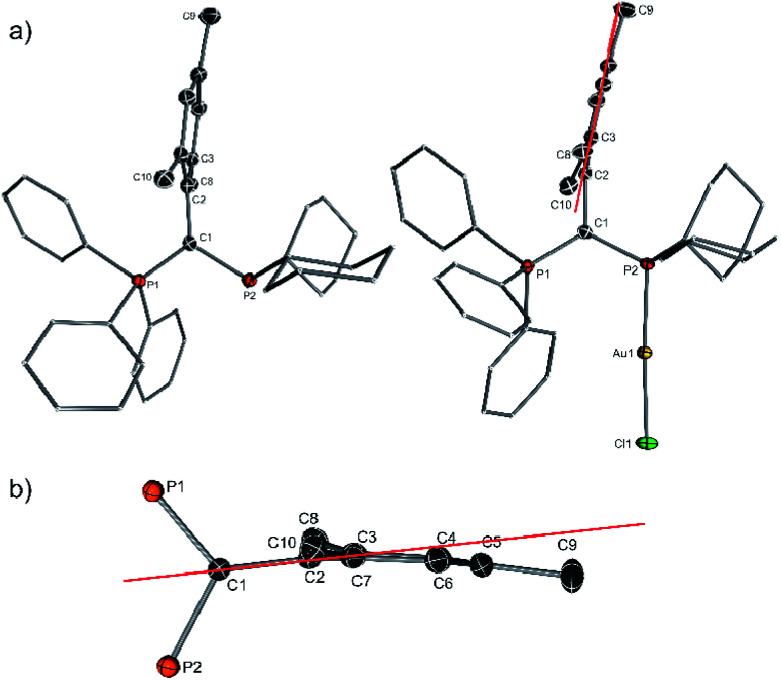
(a) Molecular structures of **5** and its gold complex and (b) extract of the molecular structure of **5** showing the distortion of the mesityl substituent.

Y_Mes_PCy_2_ features two doublets in the ^31^P{^1^H} NMR spectrum (9.90 and 7.16 ppm) with a large coupling constant of 170.2 Hz. The crystal structures of **5** and **5**·AuCl (Fig. 5) confirm the increased steric bulk of the ligand: besides the almost ideal perpendicular orientation of the mesityl group relative to the P–C–P plane (**5**: 87.2(1)°; **5**·AuCl: 85.3(1)°), the mesityl groups are also extremely distorted, showing a marked deviation from the ideal planarity of an aromatic substituent. In both structures, the *para*-(C1 and C9) and the *ortho*-methyl groups (C87 and C10) are tilted above and below the plane of the aromatic ring, respectively, thus resulting in an overall bending of the aryl ring (Fig. 5b). This distortion is necessary to allow the mesityl group to fit into the pocket of the PPh_3_ and PCy_2_ moieties. The increased steric bulk in the backbone also causes a slight increase of the steric demand of the ligand towards the metal. With %*V*_bur_ = 50.4 Y_Mes_PCy_2_ is the bulkiest ligand in the series of YPhos ligands **1–5** (Table 2). Unfortunately, attempts to determine the TEP of **5** repeatedly failed. Although treatment of **5** with Rh(acac)CO_2_ resulted in gas evolution, the IR spectrum showed no signal in the region expected for the C–O vibration. This is probably due to the elimination of both CO ligands from the metal as was also observed for bulky NHCs and other YPhos ligands.^24^

### PPM-level catalysis

**Fig. 6:**
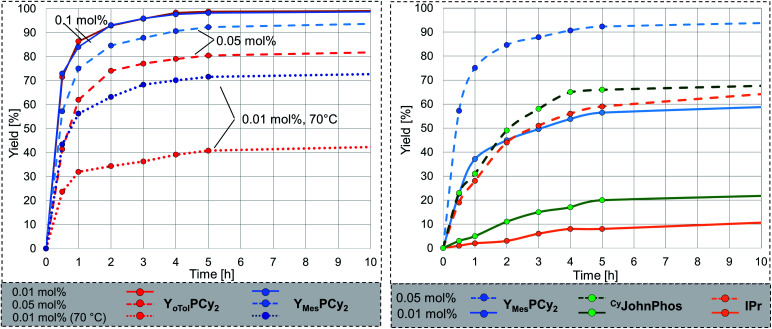
(Left) Comparison of the catalytic activity of the gold complexes with **4** and **5** at different catalyst loadings; (Right) comparison of the activity of the AuCl complexes of **5**, IPr and ^Cy^JohnPhos at 0.05 and 0.01 mol% catalyst loading. Reaction conditions: aniline (1 eq.), phenylacetylene (1 eq.), gold complex : NaBAr^F^ = 1 : 1, 50 °C (if not stated otherwise). Conversion was determined by NMR spectroscopy.

With Y_Mes_PCy_2_·AuCl in hand we next addressed the evaluation of its catalytic performance. To our delight, the complex performed equally well as the complex with the tolyl-substituted phosphine **4** at 50 °C with 0.1 mol% catalyst. However, in contrast to Y_*o*Tol_PCy_2_ it even kept its high performance when further decreasing the catalyst loading (Fig. 6, left). While for example, Y_*o*Tol_PCy_2_ only delivered approx. 85% conversion after 24 h with 0.05 mol% (TON = 1610), Y_Mes_PCy_2_ gave full conversion. Hence, the mesityl group in Y_Mes_PCy_2_ leads to the expected further increase of the catalyst stability and thus to a better performance at low catalyst loadings. This high activity is also seen at room temperature (entry 1, Table 4). Even with only 0.1 mol% catalyst full conversion could be reached within 24 h. The increased robustness of the active Y_Mes_PCy_2_-Au species allowed a further decrease of the catalyst loading. With 0.005 mol% (50 ppm) high yields of 76% (TON = 15 200, Table 3) can be reached at 70 °C, while turnover numbers as high as 20.000 can be obtained at 80 °C.

**Table 4:** Hydroamination of alkynes with different primary amines with catalysts based on **5**^a^

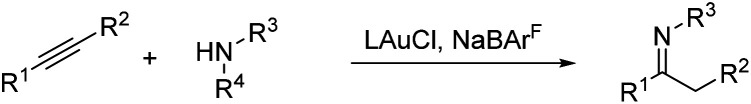									
Entry	Ligand	R^1^	R^2^	R^3^, R^4^	*T* [°C]	Time [h]	Mol% cat	Yield [%]	TON
1	**5**	Ph	H	Ph, H	25	24	0.1	99 (85)^b^	990
2	**5**	Ph	H	Ph, H	50	24	0.05	97	1950
3	**5**	Ph	H	Ph, H	70	24	0.01	74	7400
4	**4**	Ph	H	Ph, H	50	24	0.1	99	990
5	**4**	Ph	H	Ph, H	50	24	0.05	85	1610
6	**5**	Ph	H	Ph, H	70	72	0.005	76	15 200
7	**5**	Ph	H	Ph, H	70	48	0.0025	47^c^	18 800
8	**5**	Ph	H	Ph, H	80	72	0.001	20	20 000
9	**5**	Ph	H	*p*-OMeC_6_H_4_, H	50	1	0.1	99	990
10	**5**	Ph	H	*p*-OMeC_6_H_4_, H	70	72	0.01	74	7400
11	**5**	Ph	H	*p*-OMeC_6_H_4_, H	80	72	0.001	13 (8)^b^	13 043
12	**5**	*p*-MeOC_6_H_4_	H	*p*-OMeC_6_H_4_, H	50	1	0.1	99	990
13	**5**	*p*-MeOC_6_H_4_	H	*p*-OMeC_6_H_4_, H	70	24	0.01	66	6600
14	**5**	*p*-MeOC_6_H_4_	H	*p*-OMeC_6_H_4_, H	80	24	0.001	18 (11)^b^	18 256
15	**5**	Ph	H	*o*-MeC_6_H_4_, H	50	24	0.1	99	990
16	**5**	*n*-Bu	H	Ph, H	80	24	0.2	98	490
17	**5**	Ph	Me	Ph, H	80	24	0.2	98	490
18	**5**	Ph	Ph	Ph, H	80	24	0.2	53	265
19	**5**	Ph	H	Mes, H	50	24	0.1	99	990
20	**5**	Ph	H	Ph, Me	70	48	0.5	69 (65)	138
21	**5**	Ph	H	*p*-MeC_6_H_4_, Me	70	48	0.5	72	144
22	**5**	Ph	H	*p*-MeC_6_H_4_, Et	70	48	0.7	70 (62)	100
23	**5**	Ph	H	–C_2_H_4_OC_2_H_4_-morpholine	70	24	0.1	99	990

a Reaction conditions: 5 mmol acetylene, 5 mmol amine, gold complex : NaBAr^F^ = 1 : 1. Yields were determined by NMR spectroscopy.

b 20 mmol acetylene, 20 mmol amine, gold complex : NaBAr^F^ = 1 : 1. Yields were determined by NMR spectroscopy. Values in brackets are isolated yields.

c 50 mmol acetylene, 50 mmol amine, gold complex : NaBAr^F^ = 1 : 1. With additional substrate after 24 h (50 mmol). Yields were determined by NMR spectroscopy. Values in brackets are isolated yields.

**Fig. 7:**
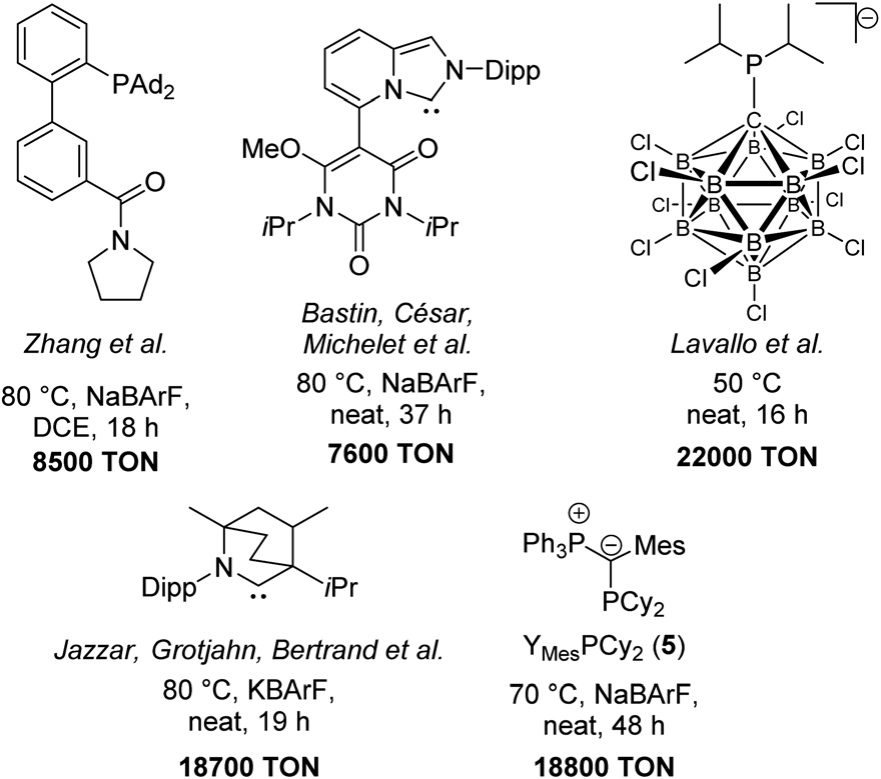
Comparison of the most efficient gold(i) catalysts in the hydroamination of phenylacetylene with aniline (Dipp = 2,6-diisopropylphenyl).

To further evaluate the activity of **5** we compared the performance of its gold complex with that of gold chloride complexes of well-established, commercially available ligands. We chose the N-heterocyclic carbene IPr (IPr = 1,3-bis(2,6-diisopropylphenyl)imidazol-2-ylidene) and the biaryl phosphine ^Cy^JohnPhos which have been used in gold catalysis in the past (Fig. 6, right).^8,25^ All ligands were tested under the same reaction conditions, *i.e.* at 50 °C with 0.05 and 0.01 mol% catalyst loading. The conversion over time plots clearly confirm the superior performance of **5** at low catalyst loadings. For example, while Y_Mes_PCy_2_·AuCl provides approx. 95% yield after 10 h reaction time with 0.05 mol% catalyst loading, only approx. 65% of product are formed when using the NHC- and biaryl-phosphine catalysts. Overall, Y_Mes_PCy_2_ provides a highly active and efficient catalyst for the hydroamination of acetylene. It's activity is higher than those of simple phosphine ligands and NHC and even higher than that observed for the sulfonyl-substituted YPhos ligand **A**, which is more difficult to synthesise since it requires the formation of a highly reactive metalated intermediate.^10^ Thus, Y_Mes_PCy_2_ is not only more active but also easier to synthesise. This also represents a decisive advantage compared to the most efficient gold(i) catalysts reported for the hydroamination of acetylene with aniline in literature. A comparison of the ligand structures and the performance of their gold complexes is shown in Fig. 7.^5,7,9,26^

Next, the performance of **5** in the hydroamination of a series of alkynes with different amines was tested. Excellent activities were achieved for a variety of substrates (Table 3). Even internal alkynes (entries 17 and 18), which are in general difficult to couple with other ligands including YPhos ligand **A**, were readily converted to the imine, albeit requiring somewhat higher reaction temperatures. Also, secondary amines, could be converted into the corresponding enamines and isolated in high yields (entries 20 to 21). To our delight, also the hydroamination with the aliphatic amine morpholine could be realized giving the product in near quantitative yield after 24 h (entry 23). In contrast, no activity was seen for *n*-butylamine and phenylacetylene under the same reaction conditions.

**Scheme 3:**
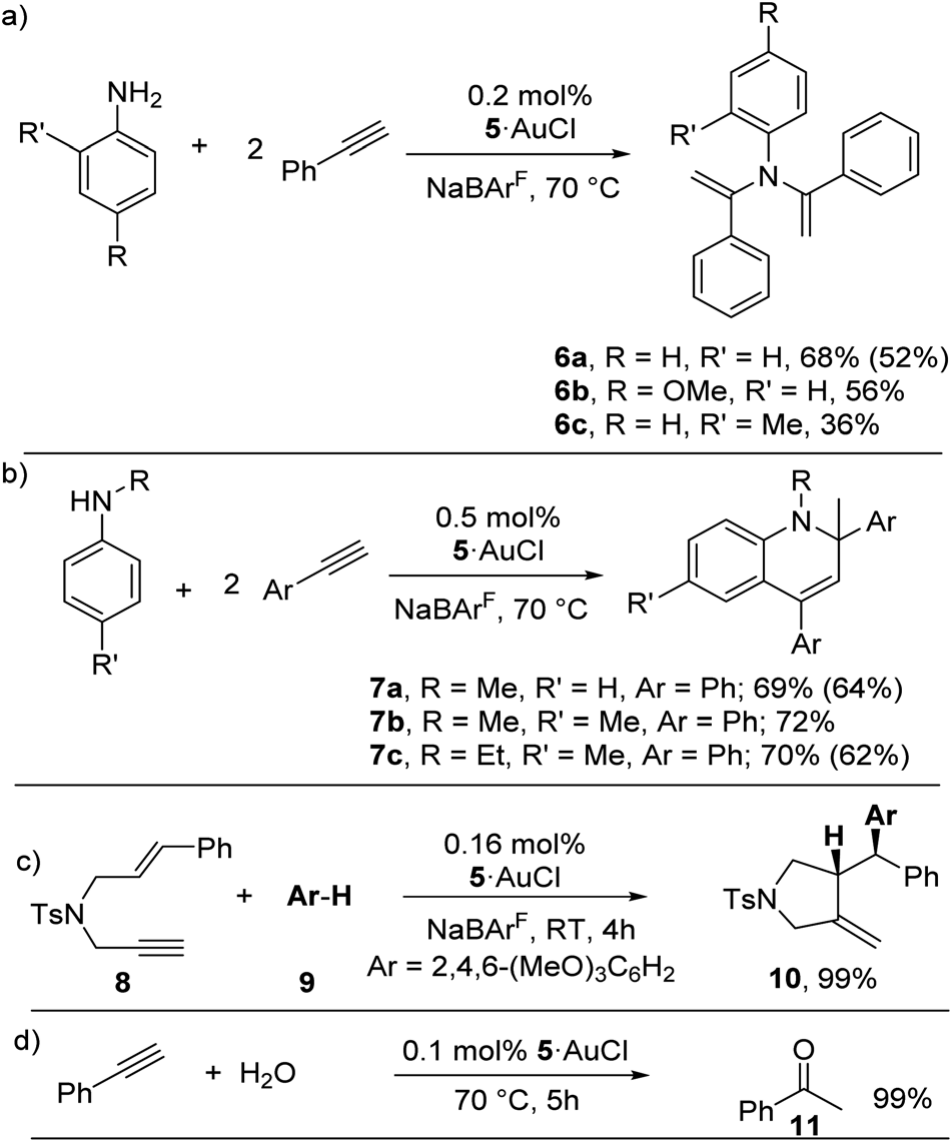
Application of Y_Mes_PCy_2_·AuCl in bisenamine and 1,2-dihydroquinoline formation. Yields were determined by NMR spectroscopy. Values in brackets correspond to isolated yields.

The activity of Y_Mes_PCy_2_·AuCl in the hydroamination with secondary amines led us to investigate its performance in reactions with two equiv. or excess of alkyne. Primary amines, such as aniline underwent double hydroamination to form bisenamines such as **6** (Scheme 3a). This reaction also proceeded at low catalyst loadings and mild reaction temperatures of only 50 °C, thus giving **6a** in 52% isolated yield.^27^ In contrast, secondary amines were found to react with two equiv. of alkyne to form 1,2-dihydroquinolines of type **7***via* a hydroamination, C–H activation and C–C bond formation sequence (Scheme 3b). Such a cyclization reaction has been performed with gold complexes with N-heterocyclic carbenes and cyclic alkyl(amino)carbenes (CAAC), however, under more forcing reaction conditions.^28^

Next, we tested the activity of **5** in a more complex reaction to further prove the scope of application of the YPhos-gold complex. We chose the domino process with 1,6-enyne **8** and trimethoxy benzene **9** as nucleophile.^29,30^ To our delight, **5**·AuCl proofed to be highly efficient also in this transformation, thus giving the cyclized compound **10** in quantitative yields after 4 h at room temperature with only 0.16 mol% catalyst loading. Thus, **5**·AuCl is similarly active in this reaction than a specially designed NHC-based catalyst described previously.^7*a*^ The formation of **10** thus also confirms the propensity of **5**·AuCl to serve as (pre)catalyst in more complex C–C bond formation reactions. Lastly, the catalyst also showed high activity in the hydration of phenylacetylene with water, thus also demonstrating the stability of the complex under aqueous conditions (Scheme 3d).

## Conclusions

In conclusion, we developed a highly active, easy-to-synthesise YPhos-based gold catalyst for hydroamination reactions of alkynes. Systematic studies on the impact of the electronic and steric properties of aryl groups in the ligand backbone impressively demonstrated the importance of steric protection of the ylidic carbon centre for the activity and stability of the catalyst. While electronic changes at the aryl substituent only had a minor impact on the catalytic activity and only gave way to low conversions under mild reaction conditions, an increase of the steric bulk by introduction of an *ortho*-tolyl and mesityl substituent led to a boost in the catalytic activity by several orders of magnitude. The gold complex of the mesityl-substituted ligand Y_Mes_PCy_2_ thus showed high conversions with only 50 ppm of catalyst loading and reached turnover numbers of 20 000. Thus, this complex competes with the most active catalysts based on highly sophisticated phosphine and NHC complexes reported in literature, albeit its simple molecular design and facile synthesis. Furthermore, Y_Mes_PCy_2_ can also be applied in further gold catalysed reactions including the formation of dihydroquinolines *via* an amination and C–C bond formation sequence or enyne cyclisation.

Overall, the remarkable increase of activity by backbone modification emphasizes the importance of controlling ligand properties also beyond typical electronic and steric properties as measured by the Tolman electronic parameter or cone angle. These results demonstrate the importance of ligand design and will be helpful for further improvements of the ligand structures also in other catalytic transformations.

## Conflicts of interest

The authors have filed patent WO2019030304 covering the YPhos ligands and precatalysts discussed, which is held by UMICORE and products will be made commercially available from.

## Supplementary Material

SC-012-D1SC00105A-s001

SC-012-D1SC00105A-s002
