# Supplementary material for: Towards the rational design of ylide-substituted phosphines for gold(i)-catalysis: from inactive to ppm-level catalysis
Source: Chem Sci. 2021 Feb 2;12(12):4329–37. doi: 10.1039/d1sc00105a (PMC8179644; doi:10.1039/d1sc00105a)
Supplement: SC-012-D1SC00105A-s001 [file SC-012-D1SC00105A-s001.pdf]

Electronic Supplementary Material (ESI) for Chemical Science.
This journal is © The Royal Society of Chemistry 2021

Towards the Rational Design of Ylide-Substituted Phosphines for Gold(I)-Catalysis: From Inactive to ppm-Level Catalysis

Jens Handelmann, Chatla Naga Babu, Henning Steinert, Christopher Schwarz, Thorsten Scherpf, Alexander Kroll, Viktoria H. Gessner*

Ruhr University Bochum, Faculty of Chemistry and Biochemistry, Chair of Inorganic Chemistry II, Universitätsstrasse 150, 44780 Bochum, Germany

Index

1. Experimental Details 2
1.1. General methods 2
1.2. Preparation of the ligands and their gold complexes 2
1.3. General procedure for gold-catalyzed hydroamination 11
1.4. Procedures for the isolation of the imines and enamines 11
1.5. Double amination: Isolation of *N,N* -bis-(1-phenylvinyl)aniline (**6a**) 15
1.6. Synthesis of the 1,2-dihydroquinolines and enyne cyclization 16
1.7. Detailled results of the catalytic screenings 18
1.8. Procedure for measurement of the IR spectra 20
2. NMR Spectra 21
2.1. NMR spectra of the isolated ligands and gold complexes 21
2.2. NMR spectra of the isolated products of the catalysis 39
3. Crystal Structure Determination 47
3.1. General information 47
3.2. Crystal strutures of the YPhos ligands 52
3.3. Crystal structures of the gold complexes 67
4. Computational Studies 83
4.1. General information 83
4.2. Energies of the structures 83
4.3. Natural charges and BCP analysis 84
4.4. Calculated Au–C distances 84
4.5. Coordinates of the energy-optimized structures 85
4.6. Exemplary Input Files 102
5. References 106

1. Experimental Details

1.1. General methods

Synthesis and materials. All experiments (if not stated otherwise) were carried out under a dry, oxygen-free argon atmosphere using standard Schlenk techniques. Argon (99.999%) was a product of *Air Liquide*. Involved solvents were dried using an MBraun SPS 800 (THF, toluene, Et_2O, acetonitrile, *n*-pentane, *n*-hexane) or dried in accordance with standard procedures and stored under an argon atmosphere over molecular sieves. Chlorodicyclohexylphosphine[1], (THT)AuCl[2] were prepared according to literature procedures, 4-methoxyaniline[3] was purified *via* sublimation prior to use. The *N*-Methylanilline derivatives were synthesized according to literature procedure.[4] The precursor for the 1,6 En-Yne cyclization was synthesized according to literature procedure.[5] All other reagents were purchased from Sigma Aldrich, ABCR, Rockwood Lithium or Acros Organics and used without further purification.

Analytical methods. ^{1}H,$^{13}C\{^{1}H\}$, $^{31}P\{^{1}H\}$ NMR spectra were recorded on an Avance 400 spectrometer at 25°C if not stated otherwise. All values of the chemical shift are in ppm regarding the δ-scale. All spin-spin coupling constants (*J*) are printed in Hertz (Hz). To display multiplicities and signal forms correctly the following abbreviations were used: s = singlet, d = doublet, t = triplet, m = multiplet, dd = doublet of doublet, dt = doublet of triplet, ddd= doublet of doublet of doublet, br = broad signal. Signal assignment was supported by DEPT, APT, HSQC and HMBC experiments and by literature studies on similar compounds. Elemental analyses were performed on an Elementar vario MICRO cube elemental analyzer. IR-Spectra were recorded on a Thermo Nicolet iS5 FT-IR in transmission mode with a Specac "Omni-cell" with KBr plates and a 0.1 mm spacer or with a ATR module at 22 °C. Melting points were measured with the SMP30 melting point apparatus from Stuart.

1.2. Preparation of the ligands and their gold complexes

1.2.1. Preparation of $\mathbf{Y_{Ph}PCy_2}$ (**1**) and its gold complex

Preparation of triphenylbenzylphosphonium bromide

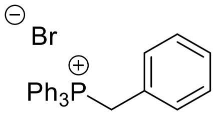

1 g (3.81 mmol) triphenylphosphine was dissolved in 50 ml toluene. 0.45 mL (3.81 mmol) 2-methylbenzylchlorid were added dropwise to the solution and stirred for 72 h. During that time the product precipitated from the solution and was filtered off and washed with 5 ml toluene. The white solid was dried *in vacuo* to afford pure product (1.32 g, 3.05 mmol, 80 %).

^{1}H NMR (400 MHz, $CDCl_3$) δ = 7.79 – 7.64 (m, 9H, $CH_{PPh3,\ ortho+para}$), 7.59 (td, $^{3}J_{HH}$ = 7.8, $^{3}J_{HH}$ = 3.6 Hz, 6H, $CH_{PPh3,\ meta}$), 7.20 – 7.16 (m, 1H, $CH_{Ph,\ para}$), 7.13 – 6.99 (m, 4H, $CH_{Ph,\ ortho+meta}$), 5.31 (d, $^{2}J_{HP}$ = 14.4 Hz, 2H, CH_2) ppm. **^{31}P{^{1}H} NMR** (162 MHz, $CDCl_3$) δ = 23.1 ppm.

Spectroscopic data match those reported in literature.[6]

Preparation of $Y_{Ph}PCy_2$ (**1**)

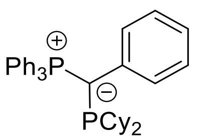

2.05 g (4,73 mmol) phosphonium salt **$Y_{Ph}H_2$** and 280 mg potassium hydride (6.98 mmol, 1.48 eq.) were dissolved in 125 mL THF and stirred for 16 h. Subsequently, the deep red solution was filtered through a cannula and the solvent was removed *in vacuo,* to yield an orange solid. The solid was suspended in 125 mL acetonitrile. To this suspension, 0.52 mL chlorodicyclohexylphosphine (2.37 mmol, 0.5 eq.) was added. The suspension became clear and after 16 h a yellow solid precipitated from the solution. The solution was stirred for an additional 24 h . The solution was filtered through a cannula and the remaining yellow solid was dried *in vacuo* to afford pure product (0.81 g, 1.48 mmol, 63 %)

^{1}H NMR (400 MHz, C_6D_6) δ = 7.87 – 7.66 (m, 6H, $CH_{PPh3\ ortho}$), 7.21 (d, $^3J_{HH}$ = 7.9 Hz, 2H, $CH_{arom.,\ ortho}$), 7.11 – 6.95 (m, 11H, $CH_{arom.,\ meta\ +\ PPh3,\ meta\ +\ PPh3\ para}$), 6.75 (t, $^3J_{HH}$ = 7.2 Hz, 1H, $CH_{arom.,\ para}$), 2.48 – 2.29 (m, 4H, $CH_{Cy\text{-}1}$ $CH_{Cy\text{-}2}$), 2.05 – 1.89 (m, 2H, $CH_{Cy\text{-}2'}$), 1.87 – 1.80 (m, 2H, $CH_{Cy\text{-}3}$), 1.80 – 1.70 (m, 2H, $CH_{Cy\text{-}3'}$), 1.69 – 1.61 (m, 2H, $CH_{Cy\text{-}4}$), 1.50 – 1.02 (m, 10H, $CH_{Cy,\ ax.}$) ppm. **^{13}C{^{1}H} NMR** (101 MHz, C_6D_6) δ = 146.2 (d, $^2J_{CP}$ = 13.6 Hz, $C_{arom.,\ ipso}$), 135.3 (dd, $^2J_{CP}$ = 8.6, $^4J_{CP}$ = 2.8 Hz, $CH_{PPh3,\ ortho}$), 131.4 (dd, $^1J_{CP}$ = 85.5, $^3J_{CP}$ = 6.5 Hz, $C_{PPh3,\ ipso}$), 131.2 (d, $^4J_{CP}$ = 2.8 Hz, $CH_{PPh3,\ para}$), 128.3 (d, $^3J_{CP}$ = 22.6 Hz, $CH_{PPh3.,\ meta}$), 127.8 (d, $^4J_{CP}$ = 19.8 Hz, $CH_{arom.,\ meta}$) 127.0 (d, $^3J_{CP}$ = 12.4 Hz, $CH_{arom.\ ortho}$), 118.6 (s, $CH_{arom.\ para}$), 37.7 (dd, $^1J_{CP}$ = 16.2, $^3J_{CP}$ = 7.8 Hz, $CH_{Cy\text{-}1}$), 34.2 (d, $^2J_{CP}$ = 26.7 Hz, $CH_{,\ Cy\text{-}2'}$), 31.9 (d, $^2J_{CP}$ = 9.0 Hz, $CH_{,\ Cy\text{-}2}$), 28.2 (d, $^3J_{CP}$ = 7.8 Hz, $CH_{,\ Cy\text{-}3}$), 27.7 (d, $^3J_{CP}$ = 14.2 Hz, $CH_{,\ Cy\text{-}3'}$), 27.3 (s, $CH_{2,\ Cy\text{-}4}$), 26.5 (dd, $^1J_{CP}$ = 86.0, $^1J_{CP}$ = 29.1 Hz, PCP) ppm. **^{31}P{^{1}H} NMR** (162 MHz, C_6D_6) δ = 19.8 (d, $^2J_{PP}$ = 185.0 Hz, PPh_3), -5.3 (d, $^2J_{PP}$ = 185.0 Hz, PCy_2) ppm. **FT-IR** (KBr, ATR, cm^{-1}): ṽ = 3055 (vw, ν (arom. C–H)), 2910 (w, ν (aliph. C–H)), 2844 (w, ν (aliph. C–H)), 1586 (w, ν (arom. C=C)), 1484 (w), 1434 (m), 1217 (m), 1104 (w), 1094 (m), 973 (w), 903 (w), 745 (s), 710 (w), 688 (s), 523 (m), 504 (s), 462 (w), 452 (w), 407 (w). **m.p.:** 170.8°C. **Anal. Calcd**. for $C_{37}H_{42}P_2$: C, 80.99; H, 7.72. Found: C, 81.02; H, 7.64.

Preparation of $Y_{Ph}PCy_2$·AuCl (**1·AuCl**)

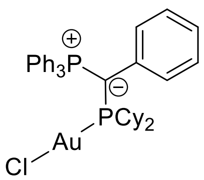

100 mg (182 µmol) phosphonium ylide **$Y_{Ph}PCy_2$** (**1**) and 55.5 mg (173 µmol, 0.95 eq.) (THT)AuCl were dissolved in 3 mL acetonitrile and the yellow suspension was stirred for 16 h. The suspension became clear and a white precipitate formed. The solution was filtered through cannula and the remaining white solid was washed with 1 mL acetonitrile and dried *in vacuo* to afford pure product (35.0 mg, 44.8 µmol, 25 % non-optimized)

^{1}H NMR (400 MHz, C_6D_6) δ = 7.86 – 7.75 (m, 6H, $CH_{PPh3,\ ortho}$), 7.12 – 7.02 (m, 11H, $CH_{arom.,\ ortho}$ $CH_{PPh3,\ meta}$ $CH_{PPh3,\ para}$), 6.92 (t, $^3J_{HH}$ = 7.5 Hz, 2H, $CH_{arom.,\ meta}$), 6.84 (m, 1H, $CH_{arom.,\ para}$), 2.22 (m, 2H, $CH_{Cy\text{-}2}$), 2.02 (m, 2H, $CH_{Cy,\ ipso}$), 1.82 – 1.64 (m, 4H, $CH_{Cy\text{-}2'}$ $CH_{Cy\text{-}3}$), 1.62 – 1.53 (m, 2H, $CH_{Cy\text{-}3'}$), 1.53 – 1.35 (m, 4H, $CH_{Cy\text{-}4}$ $CH_{Cy\ ax.}$), 1.25 – 0.90 (m, 8H, $CH_{Cy\ ax.}$) ppm. **^{13}C{^{1}H} NMR** (101 MHz, C_6D_6) δ = 142.4 (dd, $^2J_{CP}$ = 7.7, $^2J_{CP}$ = 1.9 Hz, $C_{arom.,\ ipso}$), 134.8 (d, $^2J_{CP}$ = 8.9 Hz, $CH_{PPh3,\ ortho}$), 134.3 (dd, $^3J_{CP}$ = 7.1, $^3J_{CP}$ = 3.0 Hz, $CH_{arom.,\ ortho}$), 131.9 (d, $^4J_{CP}$ 2.8 Hz, $CH_{PPh3,\ para}$), 129.8 (dd, $^1J_{CP}$ = 88.6, $^3J_{CP}$ = 1.5 Hz, $C_{PPh3,\ ipso}$), 128.7 (d, $^3J_{CP}$ = 11.8 Hz, $CH_{PPh3,\ meta}$), 128.1 (d, $^4J_{CP}$ = 23.9 Hz, $CH_{arom.,\ meta}$), 124.4 (s, $CH_{arom.,\ para}$), 40.5 – 39.3 (m, $C_{Cy\text{-}1}$), 31.8 (d,

$^2J_{CP}$ = 2.8 Hz, $CH_{Cy\text{-}2'}$), 31.0 (s, $CH_{Cy\text{-}2}$), 27.7 (d, $^3J_{CP}$ = 12.6 Hz, $CH_{Cy\text{-}3}$), 27.2 (d, $^3J_{CP}$ = 13.7 Hz, $CH_{Cy\text{-}3'}$), 26.4 (s, $CH_{Cy\text{-}4}$), 21.7 (d, $^1J_{CP}$ = 61.3 Hz, P*C*P) ppm. **^{31}P{^{1}H} NMR** (162 MHz, C_6D_6) δ = 33.6 (d, $^2J_{PP}$ = 77.2 Hz, *P*Cy_2), 20.8 (d, $^2J_{PP}$ = 77.2 Hz, *P*Ph_3) ppm. **FT-IR** (KBr, ATR, cm^{-1}): ṽ = 3057 (vw, ν (arom. C–H)), 2932 (w, ν (aliph. C–H)), 2844 (w, ν (aliph. C–H)), 1587 (w), 1483 (m), 1439 (m), 1213 (m), 1094 (m), 1077 (w), 1041 (m), 996 (m), 913 (m), 852 (w), 751 (m), 696 (s), 653 (m), 546 (m), 535 (m), 524 (m), 504 (s), 487 (m), 411 (m). **m.p.:** 193.8°C. **Anal. Calcd.** for $C_{37}H_{42}P_2AuCl$: C, 56.89; H, 5.42. Found: C, 56.51; H, 5.47.

1.2.2. Preparation of **$Y_{pOMe}PCy_2$** (**2**) and its gold complex

Preparation of $Y_{pOMe}H_2$

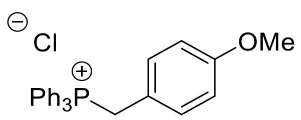

1.00 g (3.81 mmol) triphenylphosphine were dissolved in 50 mL toluene. To the stirred solution 0.69 mL (3.81 mmol) 4-methoxybenzylchlorid was added dropwise. The solution was refluxed for 72 h and the precipitate was filtered off. The white solid was washed with 5 ml toluene and dried *in vacuo* to afford pure product (1.28 g, 3.06 mmol, 80 %).

^{1}H NMR (400 MHz, $CDCl_3$) δ = 7.85 – 7.71 (m, 3H, $CH_{PPh3,\ para}$), 7.71 – 7.51 (m, 12H, $CH_{PPh3,\ ortho+para}$), 7.16 – 7.04 (m, 2H, $CH_{arom.}$), 6.95 (m, 2H, $CH_{arom.}$), 5.35 (d, $^2J_{HP}$ = 14.2 Hz, 2H, CH_2), 1.63 (s, 3H, OCH_3) ppm. **^{31}P{^{1}H} NMR** (162 MHz, $CDCl_3$) δ = 22.2 ppm.

Spectroscopic data match those reported in literature.[7]

Preparation of $Y_{pOMe}PCy_2$ (**2**)

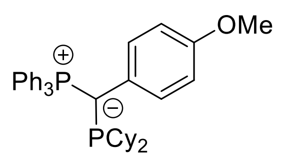

2.48 g (5.92 mmol) phosphonium salt **$Y_{pOMe}H_2$** and 356 mg potassium hydride (8.88 mmol, 1.5 eq.) were dissolved in 125 mL THF and stirred for 16 h. The deep red solution was filtered through a canula and the solvent was removed *in vacuo* to yield an orange solid. The solid was subsequently suspended in 125 mL acetonitrile. To this suspension 0.65 mL chloro-dicyclohexylphosphine (2.96 mmol, 0.5 eq.) were added. The suspension became clear and after 16 h an orange solid precipitated out of solution. The solution was stirred for an additional 24 h. The solution was filtered through a cannula and the remaining yellow solid was dried *in vacuo* to afford pure product (0.86 g, 1.49 mmol, 51 %).

^{1}H NMR (400 MHz, C_6D_6) δ = 7.88 – 7.69 (m, 6H, $CH_{PPh3,\ ortho}$), 7.14 (d, $^3J_{HH}$ = 9.1 Hz, 2H, $CH_{arom.,\ ortho}$), 7.09 – 6.97 (m, 9H, $CH_{PPh3,\ meta}$, $CH_{PPh3,\ para}$), 6.68 (d, $^3J_{HH}$ = 9.1 Hz, 2H, $CH_{arom.,\ meta}$), 3.31 (s, 3H, OCH_3), 2.56 – 2.16 (m, 4H, $CH_{Cy\text{-}1}$, $CH_{Cy\text{-}2}$), 2.00 – 1.91 (m, 2H, $CH_{Cy\text{-}2'}$), 1.89 – 1.81 (m, 2H, $CH_{Cy\text{-}3}$), 1.80 – 1.71 (m, 2H, $CH_{Cy\text{-}3'}$), 1.71 – 1.64 (m, 2H, $CH_{Cy\text{-}4}$), 1.48 – 1.07 (m, 10H, $CH_{Cy\ ax.}$) ppm. **^{13}C{^{1}H} NMR** (101 MHz, C_6D_6) δ = 153.9 (s, *C*OCH_3), 138.3 (d, $^2J_{CP}$ = 13.6 Hz, $CH_{arom.,\ ipso}$), 135.1 (dd, $^2J_{CP}$ = 8.6 Hz, $^4J_{CP}$ = 2.8 Hz, $CH_{PPh3,\ ortho}$), 132.1 (dd, $^1J_{CP}$ = 85.2, $^3J_{CP}$ = 6.2 Hz, $CH_{PPh3,\ ipso}$), 131.0 (d, $^4J_{CP}$ = 2.8 Hz, $CH_{PPh3,\ para}$), 128.6 (d, $^3J_{CP}$ = 11.5 Hz,

$CH_{arom., ortho}$), 128.1 (d, $^3J_{CP}$ = 11.6 Hz, $CH_{PPh3, meta}$), 113.6 (s, $CH_{arom., meta}$), 54.7 (s, $COCH_3$), 37.9 (dd, $^1J_{CP}$ = 15.9, $^3J_{CP}$ = 7.8 Hz, CH_{Cy-1}), 33.8 (d, $^2J_{CP}$ = 24.9 Hz, $CH_{, Cy-2}$), 31.8 (d, $^3J_{CP}$ = 9.6 Hz, $CH_{, Cy-2'}$), 28.3 (d, $^4J_{CP}$ = 8.0 Hz, $CH_{, Cy-3}$), 27.8 (d, $^4J_{CP}$ = 13.6 Hz, $CH_{, Cy-3'}$), 27.3 (s, $CH_{, Cy-4}$), 23.9 (dd, $^1J_{CP}$ = 117.3, $^1J_{CP}$ = 27.9 Hz, P*C*P) ppm. **^{31}P{^{1}H} NMR** (162 MHz, C_6D_6) δ = 18.8 (d, $^2J_{PP}$ = 182.4 Hz, *P*Ph_3), -5.0 (d, $^2J_{PP}$ = 182.4 Hz, *P*Cy_2) ppm. **FT-IR** (KBr, ATR, cm^{-1}): ṽ = 3051 (vw, ν (arom. C–H)), 2909 (w, ν (aliph. C–H)), 2843 (w, ν (aliph. C–H)), 1497 (m), 1434 (m), 1271 (w), 1238 (w), 1218 (m), 1179 (w) ,1092 (w), 1037 (m), 978 (m), 908 (m), 822 (m), 786 (m), 751 (m), 742 (m), 710 (m), 687 (m), 526 (m), 505 (s), 446 (m). **m.p.:** 172.2°C. **Anal. Calcd.** for $C_{38}H_{44}OP_2$: C, 78.87; H, 7.66. Found: C, 78.68; H, 7.46.

Preparation of $Y_{pOMe}PCy_2$·AuCl (**2·AuCl**)

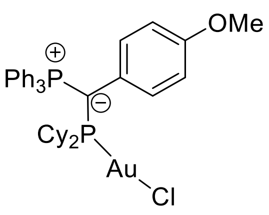

100 mg (173 µmol) phosphonium ylide **$Y_{pOMe}PCy_2$** (**2**) and 52.6 mg (164 µmol, 0.95 eq.) (THT)AuCl were dissolved in 4 mL acetonitrile and the yellow suspension was stirred for 16 h. The suspension became clear and a white precipitate formed. The solution was filtered through cannula and the remaining white solid was dried *in vacuo* to afford pure product (91.3 mg, 113 µmol, 65 %).

^{1}H NMR (400 MHz, C_6D_6) δ = 7.88 – 7.71 (m, 6H, $CH_{PPh3, ortho}$), 7.12 – 7.04 (m, 9H, $CH_{PPh3, meta + para}$), 7.04 – 6.99 (m, 2H, $CH_{arom., ortho}$), 6.56 (d, $^3J_{HH}$ = 8.5 Hz, 2H, $CH_{arom., meta}$), 3.21 (s, 3H, OCH_3), 2.33 – 2.19 (m, 2H, $CH_{Cy-2'}$), 2.11 – 1.94 (m, 2H, CH_{Cy-1}), 1.79 – 1.66 (m, 4H, $CH_{Cy-2 + Cy-3'}$), 1.64 – 1.55 (m, 2H, CH_{Cy-3}), 1.55 – 1.38 (m, 4H, $CH_{Cy-4 + Cy, ax.}$), 1.30 – 0.92 (m, 8H, $CH_{Cy, ax.}$) ppm.**^{13}C{^{1}H} NMR** (101 MHz, C_6D_6) δ = 157.9 (s, $C_{arom., para}$), 136.4 (dd, $^3J_{CP}$ = 6.2, $^3J_{CP}$ = 2.5 Hz, $CH_{arom., ortho}$), 134.7 (d, $^2J_{CP}$ = 8.7 Hz, $CH_{PPh3, ortho}$), 133.3 (dd, $^2J_{CP}$ = 7.5, $^2J_{CP}$ = 2.2 Hz, $CH_{arom., ipso}$), 131.9 (d, $^4J_{CP}$ = 2.9 Hz, $CH_{PPh3, para}$), 130.2 (dd, $^1J_{CP}$ = 88.5, $^3J_{CP}$ = 1.3 Hz, $CH_{PPh3 ipso}$), 128.7 (d, $^3J_{CP}$ = 11.7 Hz, $CH_{PPh3, meta}$), 113.7(s, $CH_{arom. meta}$), 54.6 (OCH_3), 39.8 (dd, $^1J_{CP}$ = 38.3, $^3J_{CP}$ = 3.2 Hz, CH_{Cy-1}), 31.5 (dd, $^1J_{CP}$ = 51.5, $^1J_{CP}$ = 3.5 Hz, P*C*P), 31.3 (d, $^2J_{CP}$ = 1.8 Hz, CH_{Cy-2}), 31.1 (m, $CH_{Cy-2'}$), 27.8 (d, $^3J_{CP}$ = 12.5 Hz, $CH_{Cy-3'}$), 27.3 (d, $^3J_{CP}$ = 13.5 Hz, CH_{Cy-3}), 26.5 (s, CH_{Cy-4}) ppm. **^{31}P{^{1}H}** NMR (162 MHz, C_6D_6) δ = 33.2 (d, $^2J_{PP}$ = 81.3 Hz, *P*Cy_2), 20.6 (d, $^2J_{PP}$ = 81.3 Hz, *P*Ph_3) ppm. **FT-IR** (KBr, ATR, cm^{-1}): ṽ = 3051 (vw, ν (arom. C–H)), 2922 (m, ν (aliph. C–H)), 2846 (m, ν (aliph. C–H)), 1598 (w, ν (arom. C=C)), 1500 (m, ν (arom. C=C)), 1435 (m), 1277 (w), 1244 (m), 1210 (m), 1172 (w), 1093 (s), 1060 (m), 1026 (s), 999 (m), 906 (m), 849 (w), 829 (w), 792 (m), 759 (m), 741 (m), 732 (m), 713 (m), 689 (s), 604 (w), 551 (m), 511 (m), 463 (m), 438 (w), 410 (w). **m.p.:** 193.1°C. **Anal. Calcd.** for $C_{38}H_{44}AuClOP_2$: C, 56.27; H, 5.47. Found: C, 56.67; H, 5.51.

1.2.3. Preparation of **$Y_{CF3}PCy_2$** (**3**) and its gold complex

Preparation of $Y_{pCF3}H_2$

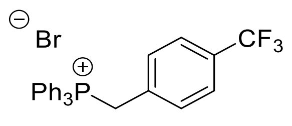

1.00 g (3.81 mmol) triphenylphosphine were dissolved in 50 mL toluene. While stirring 0.58 mL (3.81 mmol) 4-(trifluoromethyl)benzylbromide were added dropwise to the solution. The solution was refluxed for 24 h and the thus formed precipitate was filtered off and washed with 5 mL toluene. The colorless solid was dried *in vacuo* to afford pure product (1.60 g, 3.19 mmol, 84 %).

^{1}H NMR (400 MHz, $CDCl_3$) δ = 7.93 – 7.51 (m, 15H, CH_{PPh3}), 7.36 (dd, $^3J_{HH}$ = 8.1, $^3J_{HP}$ = 2.6 Hz, 2H, $CH_{arom., ortho}$), 7.28 (d, $^3J_{HH}$ = 8.1 Hz, 2H, $CH_{arom., meta}$), 5.81 (d, $^2J_{HP}$ = 15.3 Hz, 2H, CH_2) ppm. **^{19}F{^{1}H} NMR** (377 MHz, $CDCl_3$) δ = -62.6 (d, $^7J_{FP}$ = 2.8 Hz) ppm. **^{31}P{^{1}H} NMR** (162 MHz, $CDCl_3$) δ = 24.1 (q, $^7J_{PF}$ = 2.8 Hz) ppm.

Spectroscopic data match those reported in literature.[8]

Preparation of $Y_{pCF3}PCy_2$ (**3**)

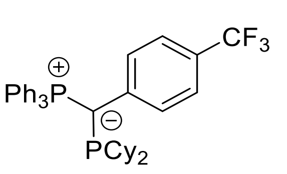

1.50 g (2.99 mmol) phosphonium salt **$Y_{pCF3}H_2$** and 0.18 g potassiumhydride (4.49 mmol, 1.5 eq.) were dissolved in 125 mL THF and stirred for 16 h. The orange solution was filtered through a cannula and the solvent was removed *in vacuo* to yield an orange solid. The solid was subsequently suspended in 125 mL acetonitrile. To this suspension 0.33 mL chlorodicyclohexylphosphine (1.50 mmol, 0.5 eq.) was added. The suspension became clear and after 16 h a yellow solid precipitated from the solution. The solution was stirred for an additional 24 h to guarantee quantitative precipitation. The solution was filtered through a cannula and the remaining yellow solid was dried *in vacuo* to afford pure product (0.62 g, 1.01 mmol, 68 %).

^{1}H NMR (400 MHz, CD_2Cl_2) δ = 7.70 – 7.58 (m, 6H, $CH_{PPh3, ortho}$), 7.58 – 7.51 (m, 3H, $CH_{PPh3, para}$), 7.49 – 7.34 (m, 6H, $CH_{PPh3, meta}$), 7.01 (d, ^3J_f = 8.5 Hz, 2H, $CH_{arom., meta}$), 6.78 (d, $^3J_{HH}$ = 8.4 Hz, 2H, $CH_{arom., ortho}$), 2.17 – 2.02 (m, 2H, $CH_{Cy\text{-}1}$), 1.91 – 1.78 (m, 2H, $CH_{Cy\text{-}2'}$), 1.72 – 1.43 (m, 8H, $CH_{Cy\text{-}2+3+3'+4}$), 1.35 – 0.77 (m, 10H, $CH_{Cy\ ax.}$) ppm.**^{13}C{^{1}H} NMR** (101 MHz, CD_2Cl_2) δ = 151.2 (d, $^2J_{CP}$ = 13.7 Hz, $C_{arom., ipso}$), 135.4 (dd, $^2J_{CP}$ = 8.8 Hz, $^4J_{CP}$ = 2.6 Hz, $CH_{PPh3, ortho}$), 132.1 (d, $^4J_{CP}$ = 2.8 Hz, $CH_{PPh3, para}$), 130.2 (dd, $^1J_{CP}$ = 86.4 Hz, $^3J_{CP}$ = 6.4 Hz, $CH_{PPh3, ipso}$), 128.8 (d, $^3J_{CP}$ = 11.6 Hz, $CH_{PPh3, meta}$) 125.0 (d, $^3J_{CP}$ = 13.3 Hz, $CH_{arom., ortho}$), 124.5 (d, $^4J_{CP}$ = 4.5 Hz, $CH_{arom., meta}$), 117.6 (q, $^2J_{CF}$ = 32.0 Hz, CCF_3), 37.2 (dd, $^1J_{CP}$ = 15.6, $^3J_{CP}$ = 7.8 Hz, $CH_{Cy\text{-}1}$), 34.3 (d, $^2J_{CP}$ = 27.2 Hz, $CH_{, Cy\text{-}2}$), 32.0 (d, $^2J_{CP}$ = 9.1 Hz, $CH_{, Cy\text{-}2'}$), 32.4 (dd, $^1J_{CP}$ = 144.3 Hz, $^1J_{CP}$ = 31.9 Hz, P*C*P), 28.2 (d, $^3J_{CP}$ = 8.0 Hz, $CH_{, Cy\text{-}3'}$), 27.8 (d, $^3J_{CP}$ = 14.6 Hz, $CH_{, Cy\text{-}3}$), 27.2 (s, $CH_{Cy\text{-}4}$) ppm. The signal of the CF_3 group could not be detected. **^{19}F{^{1}H} NMR** (377 MHz, C_6D_6) δ = -60.5 ppm. **^{31}P{^{1}H} NMR** (162 MHz, CD_2Cl_2) δ = 21.2 (d, $^2J_{PP}$ = 184.5 Hz, *P*Ph$_3$), -5.1 (d, $^2J_{PP}$ = 184.9 Hz, *P*Cy$_2$) ppm. **FT-IR** (KBr, ATR, cm^{-1}): ṽ = 3065 (vw, ν (arom. C–H)), 2921 (w, ν (aliph. C–H)), 2847 (w, ν (aliph. C–H)), 1600 (m, ν (arom. C=C)), 1505 (w, ν (arom. C=C)),

1440 (w), 1325 (m, ν (C–F)), 1248 (m), 1182 (m), 1151 (s), 1094 (m), 1065 (m), 964 (m), 908 (m), 832 (m), 768 (m), 745 (m), 711 (m), 690 (m), 667 (m), 595 (m), 539 (m), 524 (m), 504 (s), 458 (m), 448 (m). **m.p.:** 176.2°C. **Anal. Calcd.** for $C_{38}H_{41}F_3P_2$: C, 74.01; H, 6.7. Found: C, 73.89; H, 6.47.

Preparation of $Y_{pCF3}PCy_2$·AuCl (**3·AuCl**)

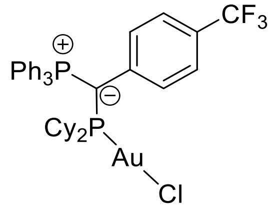

200 mg (324 µmol) phosphonium ylide **$Y_{CF3}PCy_2$** (**3**) and 98.6 mg (308 µmol, 0.95 eq.) (THT)AuCl were dissolved in 4 mL acetonitrile and the yellow suspension was stirred for 16 h. The suspension became clear and a white precipitate formed. The solution was filtered through cannula and the remaining white solid was dried *in vacuo* to afford pure product (113 mg, 145 µmol, 45 %).

^{1}H NMR (400 MHz, CD_2Cl_2) δ = 7.96 – 7.68 (m, 6H, $CH_{PPh, ortho}$), 7.65 – 7.50 (m, 3H, $CH_{PPh\ para}$), 7.50 – 7.34 (m, 6H, $CH_{PPh\ meta}$), 7.17 (d, $^4J_{HH}$ = 8.0 Hz, 2H, $CH_{arom., meta}$), 6.89 (d, $^4J_{HH}$ = 8.0 Hz, 2H, $CH_{arom., ortho}$), 2.15 – 1.85 (m, 4H, $CH_{Cy-1+2'}$), 1.80 – 1.70 (m, 6H, $CH_{Cy-2+3+3'}$), 1.66 – 1.55 (m, 2H, CH_{Cy-4}), 1.41 – 0.92 (m, 10H, $CH_{Cy\ ax.}$) ppm.**^{13}C{^{1}H} NMR** (101 MHz, C_6D_6) δ = 146.4 (d, $^2J_{CP}$ = 8.5 Hz, $C_{arom.,ipso}$), 133.7 (d, $^2J_{CP}$ = 8.9 Hz, $CH_{PPh3, ortho}$), 131.1 (d, $^4J_{CP}$ = 2.9 Hz, $CH_{PPh3, para}$), 130.7 (dd, $^3J_{CP}$ = 8.4 Hz, $^3J_{CP}$ = 3.7 Hz, $CH_{arom., ortho}$), 127.7 (dd, $^1J_{CP}$ = 57.6 Hz, $^3J_{CP}$ = 8.8 Hz, $C_{PPh3, ipso}$), 127.7 (d, $^3J_{CP}$ = 11.9 Hz, $CH_{PPh3, meta}$), 123.5 – 123.3 (m, $CH_{arom., meta}$), 38.7 (dd, $^1J_{CP}$ = 36.7 Hz, $^1J_{CP}$ = 3.2 Hz, CH_{Cy-1}), 31.4 (d, $^2J_{CP}$ = 3.8 Hz, CH_{Cy-2}), 29.9 (s, $CH_{Cy-2'}$), 26.4 (d, $^3J_{CP}$ = 12.7 Hz, CH_{Cy-3}), 25.9 (d, $^3J_{CP}$ = 14.2 Hz, $CH_{Cy-3'}$), 25.0 (d, $^4J_{CP}$ = 1.8 Hz, CH_{Cy-4}), 23.3 (dd, $^1J_{CP}$ = 118.6, $^1J_{CP}$ = 59.9 Hz, P*C*P) ppm. The signals of the CCF_3 and CF_3 groups could not be detected. **^{19}F{^{1}H} NMR** (377 MHz, C_6D_6) δ = -61.6 (s) ppm. **^{31}P{^{1}H} NMR** (162 MHz, CD_2Cl_2) δ = 35.0 (d, $^2J_{PP}$ = 73.3 Hz, *P*Cy_2), 21.7 (d, $^2J_{PP}$ = 73.3 Hz, *P*Ph_3) ppm. **FT-IR** (KBr, ATR, cm^{-1}): ṽ = 3058 (vw, ν (arom. C–H)), 2919 (m, ν (aliph. C–H)), 2848 (m, ν (aliph. C–H)), 1604 (m, ν (arom. C=C)), 1507 (w, ν (arom. C=C)), 1438 (m), 1327 (m, ν (C–F)), , 1228 (m), 1194 (m), 1160 (m), 1112 (m), 1069 (m), 995 (m), 908 (m), 829 (m), 741 (m), 709 (s), 691 (m), 669 (m), 601 (w), 550 (m), 528 (s), 507 (m), 488 (m), 445 (m), 411 (m). **m.p.:** 203.6°C. **Anal. Calcd.** for $C_{38}H_{41}AuClF_3P_2$: C, 53.57; H, 4.87. Found: C, 53.56; H, 4.93.

1.2.4.Preparation of **$Y_{oTol}PCy_2$** (**4**) and its gold complex

Preparation of $Y_{oTol}H_2$

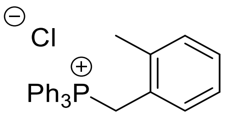

7.30 g (27.8 mmol) triphenylphosphine were dissolved in 100 mL toluene. To the stirred solution, 3.61 mL (27.8 mmol) 2-methylbenzyl chloride were added dropwise. The solution was refluxed for 72 h and the thus formed precipitate was filtered off. The white solid was dried *in vacuo* to afford pure product (8.01 g, 20.1 mmol, 72 %).

^{1}H NMR (400 MHz, $CDCl_3$) δ = 7.71 (m, 3H, C$H_{PPh3, para}$), 7.62 – 7.51 (m, 12H, C$H_{PPh3\ ortho + meta}$), 7.08 – 6.85 (m, 4H, C$H_{arom.}$), 5.23 (d, $^{2}J_{HP}$ = 14.2 Hz, 2H), 1.58 (s, 3H, CH_3) ppm. **^{31}P{^{1}H} NMR** (162 MHz, $CDCl_3$) δ = 22.2 ppm.

Spectroscopic data match those reported in literature.[8]

Preparation of $Y_{oTol}PCy_2$ (**4**)

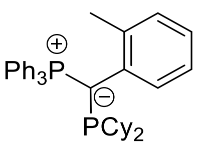

4.58 g (11.4 mmol) phosphonium salt **$Y_{oTol}H_2$** and 600 mg potassiumhydride (15 mmol, 1.30 eq.) were dissolved in 125 mL THF and stirred for 16 h. The deep red solution was filtered through a cannula and the solvent was removed *in vacuo* to yield an orange solid. The solid was subsequently suspended in 100 mL acetonitrile. To this suspension 1.26 mL chlorodicyclohexylphosphine (5.69 mmol, 0.5 eq.) were added. The suspension became clear and after 16 h a yellow solid precipitated from the of solution. The solution was stirred for an additional 24 h. The solution was filtered through a cannula and the remaining yellow solid was dried *in vacuo* to afford pure product (1.43 g, 2.39 mmol, 42 %).

^{1}H NMR (400 MHz, C_6D_6) δ = 7.89 – 7.60 (m, 6H, C$H_{ortho, PPh3}$), 7.52 – 7.44 (m, 1H, C$H_{ortho, Ar.}$), 7.13 – 7.07 (m, 1H, C$H_{meta-1,Ar.}$), 7.06 – 6.95 (m, 11H, C$H_{meta + para, PPh3, meta-2 + para, Ar.}$), 2.32 – 2.17 (m, 5H, C$H_3$ + CH_{Cy-2}), 2.17 – 2.05 (m, 2H, CH_{Cy-1}), 1.89 – 1.71 (m, 6H, C$H_{Cy-2' + Cy-3 + Cy-3'}$), 1.71 – 1.64 (m, 2H, CH_{Cy-4}), 1.52 – 1.10 (m, 10H, C$H_{Cy-ax.}$) ppm. **^{13}C{^{1}H} NMR** (101 MHz, C_6D_6) δ = 143.2 (dd, $^{2}J_{CP}$ = 11.1 Hz, $^{2}J_{CP}$ = 1.2 Hz, $C_{arom., ipso}$), 141.4 (d, $^{3}J_{CP}$ = 5.2 Hz, $C_{arom., ortho-2}$), 136.8 (d, $^{3}J_{CP}$ = 7.1 Hz, $CH_{arom., ortho-1}$), 134.4 (dd, $^{2}J_{CP}$ = 8.3, $^{4}J_{CP}$ = 3.0 Hz, $CH_{PPh3, ortho}$), 132.7 (dd, $^{1}J_{CP}$ = 84.3, $^{3}J_{CP}$ = 4.9 Hz, $C_{PPh3, ipso}$), 130.7 (d, $^{4}J_{CP}$ = 2.7 Hz, $CH_{PPh3, para}$), 130.5 (d, $^{4}J_{CP}$ = 1.8 Hz, $CH_{arom., meta-2}$), 128.0 (d, $^{3}J_{CP}$ = 11.3 Hz, $CH_{PPh3, meta}$), 125.5 (d, $^{4}J_{CP}$ = $CH_{arom., meta-1}$) 124.7 (d, $^{5}J_{CP}$ = 2.6 Hz, $CH_{arom., para}$), 39.0 (dd, $^{1}J_{CP}$ = 15.1, $^{3}J_{CP}$ = 7.3 Hz, CH_{Cy-1}), 32.7 (d, $^{2}J_{CP}$ = 17.1 Hz, CH_{Cy-2}), 31.4 (d, $^{2}J_{CP}$ = 10.3 Hz, $CH_{Cy-2'}$), 28.8 (d, $^{3}J_{CP}$ = 11.7 Hz, CH_{Cy-3}), 28.3 (d, $^{3}J_{CP}$ = 8.0 Hz, $CH_{Cy-3'}$), 27.4 (s, CH_{Cy-4}), 22.5 (s, CH_3), 20.0 (dd, $^{1}J_{CP}$ = 116.2, $^{1}J_{CP}$ = 28.1 Hz, P*C*P) ppm. **^{31}P{^{1}H} NMR** (162 MHz, C_6D_6) δ = 13.9 (d, $^{2}J_{PP}$ = 160.4 Hz, *P*Ph$_3$), -1.3 (d, $^{2}J_{PP}$ = 159.9 Hz, *P*Cy$_2$) ppm. **FT-IR** (KBr, ATR, cm^{-1}): ṽ = 3053 (vw, ν (arom. C–H)), 2917 (m, ν (aliph. C–H)), 2845 (m, ν (aliph. C–H)), 1473 (w), 1435 (m), 1235 (m), 1189 (w), 1107 (w), 1090 (m), 990 (m), 897 (m), 849 (m), 784 (m), 743 (m), 726 (m), 691 (s), 562 (m), 544 (m), 504 (m), 455 (m). **m.p.:** 128.7°C. **Anal. Calcd.** for $C_{38}H_{44}P_2$: C, 81.11; H, 7.88. Found: C, 80.90; H, 7.83.

Preparation of $Y_{oTol}PCy_2$·AuCl (**4·AuCl**)

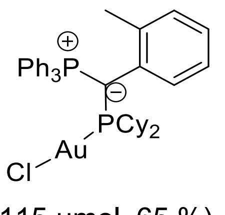

100 mg (178 µmol) phosphonium ylide **$Y_{oTol}PCy_2$** (**4**) and 54.1 mg (169 µmol, 0.95 eq.) (THT)AuCl were dissolved in 4 mL acetonitrile and the yellow suspension was stirred for 16 h. The suspension became clear and a white precipitate formed. The solution was filtered through cannula and the remaining white solid was dried *in vacuo* to afford pure product (91 mg, 115 µmol, 65 %).

^{1}H NMR (400 MHz, C_6D_6) δ = 7.71 – 7.61 (m, 6H, $CH_{PPh3, ortho}$), 7.38 – 7.32 (m, 1H), 7.13 – 7.00 (m, 9H), 6.98 – 6.92 (m, 2H), 6.84 (dd, *J* = 7.1, 2.2 Hz, 1H), 2.36 (m, 1H, CH_{Cy}). 2.29 – 2.10 (m, 2H, $CH_{Cy, ipso}$), 2.05 (s, 3H, CH_3), 2.01 – 1.95 (m, 1H, CH_{Cy}), 1.86 (m, 1H, CH_{Cy}), 1.79 – 1.71 (m, 1H, CH_{Cy}), 1.66 – 0.86 (m, 16H, CH_{Cy}) ppm. **^{13}C{^{1}H} NMR** (101 MHz, C_6D_6) δ = 143.6 – 143.3 (m, $C_{arom., ipso}$), 139.9 – 139.5 (m, $CCH_{3\,arom.}$), 138.3 – 137.7 (m, $CH_{arom., ortho}$), 134.7 (d, $^2J_{CP}$ = 8.7 Hz, $CH_{PPh3, ortho}$), 132.0 (d, $^4J_{CP}$ = 2.9 Hz, $CH_{PPh3, para}$), 131.1 (s, $CH_{arom., meta1}$), 129.7 (dd, $^1J_{CP}$ = 88.6, $^3J_{CP}$ = 1.5 Hz, $C_{PPh3, ipso}$), 128.5 (d, $^3J_{CP}$ = 11.6 Hz, $CH_{PPh3, meta}$), 126.9 (s, $CH_{arom., para}$), 125.8 (m, $CH_{arom., meta2}$), 42.98 – 40.07 (m, CH_{Cy}), 40.20 – 37.92 (m, CH_{Cy}), 32.79 – 32.03 (m, CH_{Cy}), 31.78 – 31.17 (m, CH_{Cy}), 30.99 – 30.23 (m, CH_{Cy}), 28.07 – 27.84 (m, CH_{Cy}), 27.79 – 27.56 (m, CH_{Cy}), 27.31 – 27.03 (m, CH_{Cy}), 26.70 – 26.12 (m, CH_{Cy}), 21.75 (s, CH_3), 19.55 (dd, $^1J_{CP}$ = 115.8, $^1J_{CP}$ = 59.5 Hz, P*C*P). **^{31}P{^{1}H} NMR** (162 MHz, C_6D_6) δ = 35.0 (d, $^2J_{PP}$ = 79.2 Hz, PCy_2), 18.7 (d, $^2J_{PP}$ = 79.2 Hz, PPh_3) ppm. **FT-IR** (KBr, ATR, cm^{-1}): ṽ = 3058 (vw, v (arom. C–H)), 2980 (w, v (aliph. C–H)), 2931 (m, v (aliph. C–H)), 2848 (m, v (aliph. C–H)), 1440 (m), 1216 (m), 1097 (m), 1087 (m), 990 (m), 919 (m), 851 (w), 756 (m), 747 (m), 729 (m), 701 (m), 565 (w), 552 (s), 534 (m), 513 (m), 504 (m), 493 (s), 454 (m), 437 (w), 424 (w), 407 (w). **m.p.:** 206.9°C. **Anal. Calcd.** for $C_{38}H_{44}AuClP_2$: C, 57.40; H, 5.58. Found: C, 57.46; H, 5.57.

1.2.5. Preparation of **$Y_{Mes}PCy_2$** (**5**) and its gold complex

Preparation of Iodoisodurene

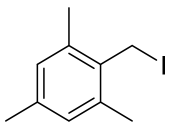

15.0 g (88.9 mmol) 2-(chloromethyl)-1,3,5-trimethylbenzene and 13.3 g (88.9 mmol) sodium iodide were dissolved in 200 mL acetonitrile and the solution was refluxed for 16 h. During this time the colourless solution slowly turned yellow. The resulting yellow solution was filtered through a cannula to remove the precipitated sodium chloride. The precipitate was washed 3 times with acetonitrile and the solutions were combined. The solvent was removed *in vacuo.* The product was collected as a yellow solid to afford pure product (20.0 g, 76.8 mmol, 87 %).

^{1}H NMR (400 MHz, $CDCl_3$) δ = 6.83 (s, 2H, $CH_{meta.}$), 4.46 (s, 2H, CH_2), 2.32 (s, 6H, $CH_{3, ortho}$), 2.25 (s, 3H, $CH_{3, para}$) ppm. Spectroscopic data match those reported in literature.[9]

Preparation of $Y_{Mes}H_2$

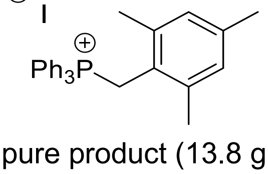

7.5 g (28.8 mmol) triphenylphosphine and 7.56 g (28.8 mmol) 2-(iodomethyl)-1,3,5-trimethylbenzene were dissolved in 125 mL toluene. The solution was refluxed for 24 h and the thus formed precipitate was filtered off. The white solid was dried *in vacuo* to afford pure product (13.8 g, 26.4 mmol, 92 %).

^{1}H NMR (400 MHz, $CDCl_3$) δ = 7.91 – 7.73 (m, 3H, $CH_{PPh3, para}$), 7.66 – 7.56 (m, 6H, $CH_{PPh3, meta}$), 7.55 – 7.45 (m, 6H, $CH_{PPh3, ortho}$), 6.71 (bs, 2H, $CH_{arom.}$), 5.02 (d, $^2J_{HP}$ = 13.6 Hz, 2H, CH_2), 2.22 (d, $^4J_{HH}$ = 2.81 Hz, 3H, $CH_{3\,para}$), 1.78 (d, $^4J_{HH}$ = 1.54 Hz, 6H, $CH_{3\,ortho}$) ppm. **^{13}C NMR** (101

MHz, $CDCl_3$) δ = 138.66 (d, $^5J_{CP}$ = 4.4 Hz, $C_{arom.\ para}$), 138.34 (d, $^3J_{CP}$ = 5.5 Hz, $C_{arom.\ ortho}$), 135.38 (d, $^4J_{CP}$ = 3.1 Hz, $CH_{PPh3,\ para}$), 134.35 (d, $^2J_{CP}$ = 9.7 Hz, $CH_{PPh3,\ ortho}$), 130.36 (d, $^3J_{CP}$ = 12.4 Hz $CH_{PPh3,\ meta}$), 130.06 (d, $^4J_{CP}$ = 3.6 Hz, $CH_{arom.\ meta}$), 121.87 (d, $^2J_{CP}$ = 9.2 Hz, $C_{arom.\ ipso}$), 117.93 (d, $^1J_{CP}$ = 84.1 Hz, $C_{PPh3,\ ipso}$), 28.54 (d, $^1J_{CP}$ = 46.4 Hz, CH_2), 21.15 (d, $^4J_{CP}$ = 1.5 Hz, $CH_{3\ ortho}$), 20.96 (d, $^6J_{CP}$ = 1.5 Hz, $CH_{3\ para}$) ppm. ^{31}**P{^{1}H} NMR** (162 MHz, $CDCl_3$) δ = 18.4 ppm. **FT-IR** (KBr, ATR, cm^{-1}): ṽ =3635 (w), 3402 (w), 3048 (w, ν (arom. C–H), 2922 (w, ν (aliph. C–H)), 2851 (w, ν (aliph. C–H)), 1587 (w), 1437 (m), 1398 (w)1299 (w), 1109 (s), 858 (m), 811 (s), 704 (s),524 (s), 505 (s). **m.p.:** 211.8°C (decomposition).

Preparation of $Y_{Mes}PCy_2$ (**5**)

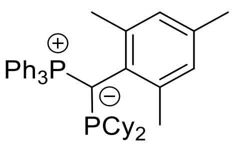

3.85 g (7.37 mmol) phosphonium salt $\mathbf{Y_{Mes}H_2}$ were suspended in 250 mL toluene and 4.64 mL *n*-butyllithium (1.6 M solution in hexane, 7.37 mmol) were added slowly to the mixture while stirring at room temperature, upon which the solution turned deep red. After 12 hours of stirring the solution was filtered through cannula. The remaining solid was washed twice with 50 ml toluene and the solvent from the combined filtrates was removed *in vacuo* The remaining red solid was transferred into a glovebox, where 809 mg (7.37 mmol, 1 eq.) sodium tetrafluoroborate were added. The solids were dissolved in 125 mL acetonitrile and 1.63 mL (7.37 mmol) chlorodicyclohexylphosphine were added. The red solution was stirred over night and a white precipitate formed. The solution was filtered through cannula and the solvent was removed *in vacuo*. The residue was redissolved in 100 ml Et_2O stirred over night and a white precipitate formed. The solution was filtered through cannula and the solvent was removed *in vacuo*. The remaining solids were transferred into a glovebox and 826 mg (7.37 mmol) KO*t*Bu were added. The solids were dissolved in toluene and stirred over night. The red solution was filtered through cannula and the solvent was removed *in vacuo*. The residue was redissolved in 125 mL acetonitrile and stirred over night. The red solution was filtered through cannula and the remaining yellow solid was washed with 10 mL acetonitrile and dried *in vacuo* to afford pure product (2.17 g, 3.67 mmol, 49 %).

^{1}**H NMR** (400 MHz, C_6D_6) δ = 7.78 – 7.61 (m, 6H, $CH_{PPh3,\ ortho}$), 7.07 – 6.88 (m, 9H, $CH_{PPh3,\ meta\ +\ para}$), 6.83 (s, 2H, $CH_{arom.}$), 2.47 (s, 6H, $CH_{3\ ortho}$), 2.27 – 2.18 (m, 4H, $CH_{Cy\text{-}1\ Cy\text{-}2}$), 2.15 (s, 3H, $CH_{3\ para}$), 1.94 – 1.85 (m, 2H, $CH_{Cy\text{-}2'}$), 1.85 – 1.64 (m, 6H, $CH_{Cy\text{-}3\ +\ Cy\text{-}3'\ +\ Cy\text{-}4}$), 1.55 – 1.09 (m, 10H, $CH_{Cy\ ax.}$) ppm. ^{13}**C{^{1}H} NMR** (101 MHz, C_6D_6) δ = 143.2 (d, $^3J_{CP}$ = 5.5 Hz, $C_{arom.,\ ortho}$), 139.4 (d, $^2J_{CP}$ = 9.8 Hz, $C_{arom.,\ ipso}$), 134.1 (dd, $^2J_{CP}$ = 7.9, $^4J_{CP}$ = 3.8 Hz, $CH_{PPh3,\ ortho}$), 133.9 (d, $^5J_{CP}$ = 2.9 Hz, $C_{arom.,\ para}$), 133.9 (dd, $^1J_{CP}$ = 81.0, $^3J_{CP}$ = 8.6 Hz, $C_{PPh3,\ ipso}$), 130.6 (d, $^4J_{CP}$ = 2.6 Hz, $CH_{PPh3,\ para}$), 129.0 (d, $^3J_{CP}$ = 1.9 Hz), 128.0 (m, $CH_{PPh3,\ meta}$), 40.4 (dd, $^1J_{CP}$ = 16.2, $^3J_{CP}$ = 6.8 Hz, $CH_{Cy\text{-}1}$), 34.3 (d, $^2J_{CP}$ = 22.3 Hz, $CH_{2\ Cy\text{-}2}$), 31.6 (d, $^2J_{CP}$ = 4.5 Hz, $CH_{2\ Cy\text{-}2'}$), 29.1 (d, $^3J_{CP}$ = 14.4 Hz, $CH_{2\ Cy\text{-}3}$), 28.7 (d, $^3J_{CP}$ = 4.9 Hz), 27.4 (s, $CH_{2\ Cy\text{-}4}$), 23.6 (s, $CH_{3\ ortho}$), 21.0 ($CH_{3\ para}$) ppm. (P*C*P was not observed). ^{31}**P{^{1}H} NMR** (162 MHz, C_6D_6) δ = 9.90 (d, $^2J_{PP}$ = 170.2 Hz, *P*Ph_3), 7.16 (d, $^2J_{PP}$ = 170.2 Hz, *P*Cy_2) ppm. **FT-IR** (KBr, ATR, cm^{-1}): ṽ = 3053 (vw, ν (arom. C–H), 2981 (w, ν (aliph. C–H)), 2915 (m, ν (aliph. C–H)), 2844 (w, ν (aliph. C–H)), 1432 (m), 1212 (w), 1107 (m), 1091 (m), 984 (m), 951 (w), 878 (m), 747 (m), 731 (w), 695 (s), 595 (w), 572 (m), 556 (m), 507 (s), 468 (m), 445 (m). **m.p.:** 133.2°C. **Anal. Calcd.** for $C_{40}H_{48}P_2$: C, 81.32; H, 8.19. Found: C, 81.07; H, 7.80.

Preparation of $Y_{Mes}PCy_2$·AuCl (**5·AuCl**)

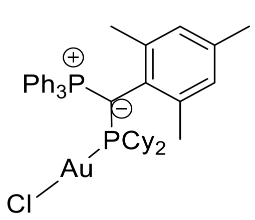

100 mg (169 µmol) phosphonium ylide **$Y_{Mes}PCy_2$** (**5**) and 50.2 mg (160 µmol, 0.95 eq.) (THT)AuCl were dissolved in 3 mL acetonitrile and the yellow suspension was stirred for 16 h and a white precipitate formed. The solution was filtered through cannula and the remaining off-white solid was washed with 6 ml pentane and dried *in vacuo* to afford pure product (87.2 mg, 106 µmol, 63 %).

^{1}H NMR (400 MHz, C_6D_6) δ = 7.81 – 7.65 (m, 6H, C$H_{PPh3, ortho}$), 7.12 – 6.93 (m, 9H, C$H_{PPh3, meta + para}$), 6.68 (s, 2H, C$H_{arom.}$), 2.42 – 2.33 (m, 2H, CH_{Cy-2}), 2.31 (s, 6H, C$H_{3\,ortho}$), 2.18 – 2.08 (m, 2H, CH_{Cy-1}), 2.05 (s, 3H, C$H_{3\,para}$), 1.72 – 1.63 (m, 2H, CH_{Cy-3}), 1.64 – 0.91 (m, 16H, CH_{Cy}) ppm. **^{13}C{^{1}H} NMR** (101 MHz, C_6D_6) δ = 144.2 (dd, $^2J_{CP}$ = 4.8, ^2J = 2.5 Hz, $C_{arom., ipso}$), 136.3 – 135.7 (m, CH$_{arom. ortho + para}$), 134.6 (d, $^2J_{CP}$ = 8.7 Hz, CH$_{PPh3, ortho}$), 131.9 (d, $^4J_{CP}$ = 2.9 Hz, CH$_{PPh3, para}$), 129.5 (m, CH$_{arom., meta}$), 128.5 (d, $^3J_{CP}$ = 11.8 Hz, CH$_{PPh3, meta}$), 41.7 (dd, $^1J_{CP}$ = 36.2, $^3J_{CP}$ = 3.5 Hz, CH$_{Cy-1}$), 34.3 (d, $^2J_{CP}$ = 4.0 Hz, CH$_{Cy-2}$), 30.5 (s, CH$_{Cy-2'}$), 28.0 (d, $^3J_{CP}$ = 14.5 Hz, CH$_{Cy-3}$), 27.8 (d, $^3J_{CP}$ = 11.4 Hz, CH$_{Cy-3'}$), 26.4 (s, CH$_{Cy-4}$), 23.4 (s, CH$_{3\,ortho}$), 20.8 (s, CH$_{3\,para}$) ppm. (PCP und $C_{PPh3, ipso}$ were not observed). **^{31}P{^{1}H} NMR** (162 MHz, C_6D_6) δ = 36.7 (d, $^2J_{PP}$ = 87.0 Hz, *P*Cy$_2$), 13.6 (d, $^2J_{PP}$ = 87.0 Hz, *P*Ph$_3$) ppm. **FT-IR** (KBr, ATR, cm^{-1}): ṽ = 3059 (vw, ν (arom. C–H), 2917 (m, ν (aliph. C–H)), 2846 (m, ν (aliph. C–H)), 1434 (m), 1203 (m), 1153 (w), 1105 (m), 1092 (m), 1012 (m), 995 (m), 953 (w), 886 (m), 854 (w), 749 (m), 706 (m), 692 (s), 601 (m), 576 (m), 564 (m), 537 (m), 514 (m), 504 (s), 479 (m), 451 (m). **m.p.:** 227.5°C. **Anal. Calcd.** for $C_{40}H_{48}AuClP_2$: C, 58.36; H, 5.88. Found: C, 58.62; H, 5.52.

1.3. General procedure for gold-catalyzed hydroamination

A 2 mL glass vial with a rubber cap and a stir bar was charged in a glovebox with the indicated amount of LAuCl and NaBAr^F. The amine (5.25 mmol) and the alkyne (5.00 mmol) were added via syringe. The vial was heated on a hotplate to the indicated temperature while stirring. Small aliqouts were removed via a syringe and added directly to an NMR tube to monitor the reaction progress. Yields were calculated by integration of the peak for the alkyne starting material with respect to the peak for the imine product in the ^{1}H NMR spectrum.

1.4. Procedures for the isolation of the imines and enamines

Synthesis of 1-Phenyl-*N*-phenylethan-1-imine

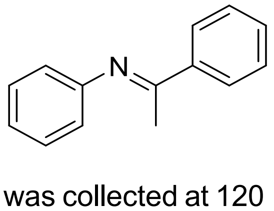

$Y_{Mes}PCy_2$•AuCl (16 mg, 20 µmol), NaBAr$^F_4$ (18 mg, 20 µmol), aniline (1.8 mL, 1.9 g, 20 mmol) and phenylacetylene (2.2 mL, 2.0 g, 20 mmol) were added to a flask. The solution was stirred at room temperature for 24 h. The contents of the flask were directly distilled through Kugelrohr distillation at 1 · 10^{-3} mbar. After a small forerun at 40 °C the product was collected at 120 °C as a colourless oil that solidified upon standing (3.3 g, 17 mmol, 85 %).

^{1}H NMR (400 MHz, $CDCl_3$) δ = 8.02 – 7.93 (m, 2H), 7.50 – 7.43 (m, 3H), 7.41 – 7.32 (m, 1H), 7.15 – 7.06 (m, 2H), 6.87 – 6.75 (m, 2H), 2.24 (s, 3H) ppm. **^{13}C{^{1}H} NMR** (101 MHz, CDCl3) δ = 165.6, 151.8, 139.6, 130.6, 129.1, 128.5, 127.3, 123.3, 119.5, 17.5. **FT-IR** (KBr, ATR, cm^{-1}): ṽ = 3053 (w), 3027 (w), 2980 (w), 1623 (w), 1590 (w), 1575 (w), 1480 (w), 1444 m 1362 (w), 1287 (w), 1275 (w), 1212 m 1166 (w), 1074 (m), 908 (w), 810 (m), 781 (m), 758 (s), 731 (s), 699 (vs), 690 (vs), 570 (m). **m.p.:** 40°C. Spectroscopic data matches the literature[10].**m.p.** matches the literature[11]

Synthesis of 1-(4-Methoxyphenyl)-*N*-phenylethan-1-imine

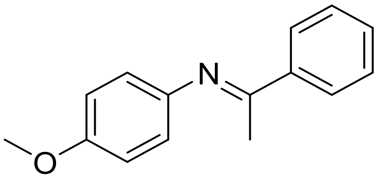

An equimolar solution of 4-methoxyaniline and phenylacetylen was prepared. **$Y_{Mes}PCy_2$•AuCl** (4 mg, 5 µmol) and $NaBAr^F_4$ (4 mg, 5 µmol), were dissolved in 1 mL of the prepared solution. Aliquots were taken from this solution and further diluted until the desired concentration of catalyst was obtained. 0.1 mL of that solution were added to a solution of phenylacetylen (2.2 mL, 2.0 g, 20 mmol) and 4-methoxyaniline (2.5 g, 20 mmol). The solution was stirred at 80 °C for 24 h. The contents of the flask were directly distilled through Kugelrohr distillation at $1 \cdot 10^{-3}$ mbar. After a small forerun at 40 °C the product was collected at 100 °C as a yellow oil which solidified upon standing (0.36 g, 1.6 mmol, 8%).

^{1}H NMR (400 MHz, $CDCl_3$) δ = 8.11 – 7.87 (m, 2H), 7.45 (m, 3H), 6.92 (m, 2H), 6.87 – 6.71 (m, 2H), 3.82 (s, 3H), 2.26 (s, 3H) ppm. **^{13}C{^{1}H} NMR** (101 MHz, CDCl3) δ = 165.7, 156.0, 144.9, 139.8, 130.4, 128.4, 127.2, 120.8, 114.3, 55.5, 17.4. **FT-IR** (KBr, ATR, cm^{-1}): ṽ = 3058 (vw), 3029 (w), 2995 (w), 2953 (w), 2928 (w), 2905 (w), 2834 (w), 1615 (m), 1575 (m), 1500 (s) 1459 (m), 1443 (s), 1365 (m), 1282 (m), 1238 (s), 1209 (s), 1105 (m), 1030 (vs), 844 (s), 770 (s), 760 (s), 752 (s), 708 (m), 697 (vs), 573 (m), 533 (s). **m.p.:** 83.5°C.

Spectroscopic data matches the literature.[12]

Synthesis of 1-(4-Methoxyphenyl)-*N*-(4-methoxyphenyl)ethan-1-imine

An equimolar solution of 4-methoxyaniline and 4-methoxyphenylacetylen was prepared.

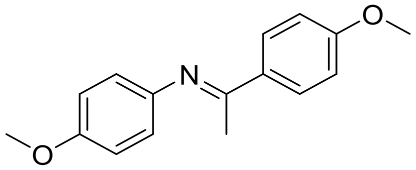

$Y_{Mes}PCy_2$•AuCl (4 mg, 5 µmol) and $NaBAr^F_4$ (4 mg, 5 µmol), were dissolved in 1 mL of the prepared solution. Aliquots were taken from this solution and further diluted until the desired concentration of catalyst was obtained. 0.1 mL of that solution were added to a solution of 4-Methoxyphenylacetylen (2.6 mL, 2.6 g, 20 mmol) and 4-Methoxyaniline (2.5 g, 20 mmol). The solution was stirred at 80 °C for 24 h. The contents of the flask were directly distilled through Kugelrohr distillation at $1 \cdot 10^{-3}$ mbar. After a small forerun at 40 °C the product was collected at 110 °C as a yellow oil which solidified upon standing. (0.56 g, 2.2 mmol, 11%)

^{1}H NMR (400 MHz, $CDCl_3$) δ = 7.94 (d, $^3J_{HH}$ = 8.9 Hz, 2H), 6.95 (d, $^3J_{HH}$ = 8.9 Hz, 2H), 6.90 (d, $^3J_{HH}$ = 8.9 Hz, 2H), 6.75 (d, J = 8.9 Hz, 2H), 3.86 (s, 3H), 3.81 (s, 3H), 2.22 (s, 3H) ppm. **^{13}C{^{1}H} NMR** (101 MHz, CDCl3) δ = 164.8, 161.5, 155.8, 145.1, 132.5, 128.8, 120.9, 114.3, 113.6, 55.5, 55.4, 17.1. **FT-IR** (KBr, ATR, cm^{-1}): ṽ = 2968 (w), 2936 (w), 2909 (w), 2836 (w), 1599 (m), 1509 (m), 1500 (s), 1439 (m), 1237 (s), 1205 (s), 1184m 1169 (s), 1115 (m), 1103 (m), 1025 (s), 842 (m), 831 (vs), 803 (s), 757 (m), 568 (s), 541 (m), 526 (m), 445 (m). **m.p.:** 134.7°C

Spectroscopic data matches the literature. [13]

1-Phenyl-*N*-(o-tolyl)ethan-1-imine (Table 4 Entry 15) was synthesized according to the general procedure using 0.1 mol% of catalyst **$Y_{Mes}PCy_2$•AuCl** and a reaction temperature of 50°C. The NMR yield was determined *via* comparison of the integral of the alkyne starting material at 3.00 ppm with respect to the methyl group of the imine product at 2.11 ppm. NMR yield: 99%.
Spectroscopic data matches the literature.[14]

E*- and *Z*-*N*-phenylhexan-2-imine** (Table 4 Entry 16) was synthesized according to the general procedure using 0.2 mol% of catalyst **$Y_{Mes}PCy_2$•AuCl** and a reaction temperature of 80°C. The NMR yield was determined *via* comparison of the integral of the alkyne starting material at 1.95 ppm with respect to the methyl group of the imine product at 0.97 ppm (t, $^3J_{HH}$ = 7.4 Hz, ***Z) and 0.81 ppm (t, $^3J_{HH}$ = 7.4 Hz, ***E***). NMR yield: 98% as a mixture of ***E*** and ***Z*** Isomers.
Spectroscopic data matches the literature.[14]

***N*,1-diphenylpropan-1-imine (A) and *N*,1-diphenylpropan-2-imine (B)** (Table 4 Entry 17) was synthesized according to the general procedure using 0.2 mol% of catalyst **$Y_{Mes}PCy_2$•AuCl** and a reaction temperature of 80°C. The NMR yield was determined *via* comparison of the integral of the alkyne starting material at 2.05 ppm with respect to the methyl group of the imine product at 1.71 ppm (s, **A**) and 1.08 ppm (t, 7.6 Hz, **B**). NMR yield: 98% as a mixture of two regioisomers in a 1:1 ratio.
Spectroscopic data matches the literature.[14]

***N*,1,2-triphenylethan-1-imine** (Table 4 Entry 18) was synthesized according to the general procedure using 0.2 mol% of catalyst **$Y_{Mes}PCy_2$•AuCl** and a reaction temperature of 80°C. The NMR yield was determined *via* comparison of the integral of the alkyne starting material at around 7.5 ppm with respect to the methylene group of the amine product at 4.08 ppm. NMR yield: 53%.
Spectroscopic data matches the literature.[14]

1-Phenyl-*N*-(mesityl)ethan-1-imine (Table 4 Entry 19) was synthesized according to the general procedure using 0.1 mol% of catalyst **$Y_{Mes}PCy_2$•AuCl** and a reaction temperature of 50°C. The NMR yield was determined *via* comparison of the integral of the alkyne starting material at 3.00 ppm with respect to the methyl group of the imine product at 2.15 ppm. NMR yield: 99%.
Spectroscopic data matches the literature.[15]

4-(1-phenylvinyl)morpholine (Table 4 Entry 23) was synthesized according to the general procedure using 0.1 mol% of catalyst **$Y_{Mes}PCy_2$•AuCl** and a reaction temperature of 70°C. The NMR yield was determined *via* comparison of the integral of the alkyne starting material at 3.00 ppm with respect to the vinylene group of the enamine product at 4.12 ppm and 4.32 ppm. NMR yield: 99%.
Spectroscopic data matches the literature.[16]

Acetophenone (**11**) was synthesized according to the general procedure using 0.1 mol% of catalyst **$Y_{Mes}PCy_2$•AuCl** and a reaction temperature of 70°. The NMR yield was determined *via* comparison of the integral of the alkyne starting material at 3.00 ppm with respect to the methyl group of the ketone product at 2.57 ppm. NMR yield: 99%.
Spectroscopic data matches the literature.[17]

Synthesis of (E/Z)-*N*-methyl-*N*-(1-phenylprop-1-en-1-yl)aniline

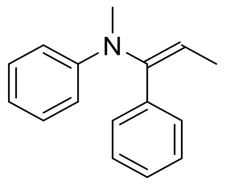

A

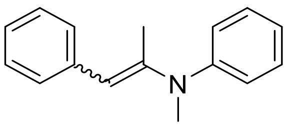

B

$Y_{Mes}PCy_2$•AuCl (8 mg, 10 µmol), $NaBAr^F_4$ (9 mg, 10 µmol), *N*-methylaniline (0.57 mL, 563 mg, 5.25 mmol) and 1-phenylpropin (0.58 mL, 581 mg, 5 mmol) were added to a flask. The solution was stirred at 65 °C for 24 h. The contents of the flask were directly distilled through Kugelrohr distillation at $1 \cdot 10^{-3}$ mbar. After a small forerun at 40 °C the product was collected at 110 °C as an orange oil. (612 mg, 2.7 mmol, 55 %). The product was obtained as a mixture of isomers.

^{1}H NMR (400 MHz, $CDCl_3$) δ = 7.47 – 7.08 (m, 17H), 7.07 – 6.96 (m, 2H), 6.81 – 6.74 (m, 2H), 6.74 – 6.62 (m, 3H), 6.20 – 6.12 (m, 1H), 6.05 – 5.97 (m, 1H), 5.96 – 5.91 (m, 1H), 3.68 – 3.65 (m, 0H), 3.20 – 3.16 (m, 2H), 3.12 – 3.04 (m, 3H), 2.98 – 2.93 (m, 2H), 2.83 – 2.79 (m, 1H), 2.14 – 2.10 (m, 1H), 1.98 – 1.89 (m, 4H), 1.67 – 1.60 (m, 3H) ppm. **^{13}C{^{1}H} NMR** (101 MHz, $CDCl_3$) δ = 148.8, 148.1, 147.0, 144.3, 144.2, 142.1, 138.9, 138.5, 136.3, 129.3, 129.1, 128.8, 128.6, 128.5, 128.2, 128.0, 127.6, 127.0, 126.2, 125.1, 124.7, 122.7, 122.4, 121.8, 117.7, 116.6, 114.1, 112.3, 41.0, 37.7, 36.9, 19.7, 17.8, 14.1 ppm. **FT-IR** (KBr, ATR, cm^{-1}): ṽ = 3058 (w), 3025 (w), 2881 (w), 2812 (w), 2361 (w), 2161 (w), 1713 (w), 1598 (vs), 1575 (w), 1500

(m), 1476 (w), 1445 (w), 1376 (w), 1356 (m), 1318 (m), 1298 (m), 1194 (m), 1156 (w), 1133 (w), 1073 (w), 1031 (vw), 994 (m), 903 (m), 870 (m), 767 (m), 751 (s), 696 (m), 514 (vw).

Spectroscopic data matches the literature.[18]

1.5. Double amination: Isolation of *N,N* -bis-(1-phenylvinyl)aniline (**6a**)

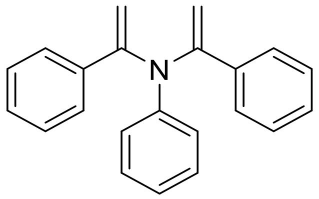

$Y_{Mes}PCy_2$•AuCl (4 mg, 5 µmol), $NaBAr^F_4$ (4 mg, 5 µmol), aniline (0.25 mL, 255 mg, 2.7 mmol) and phenylacetylene (0.8 mL, 700 mg, 6.9 mmol) were added to a flask. The solution was stirred at 50 °C for 24 h. The contents of the flask were directly distilled through Kugelrohr distillation at $1 \cdot 10^{-3}$ mbar. After a small forerun at 40 °C the product was collected at 160 °C as a red viscous oil. (421 mg, 1.4 mmol, 52 %)

^{1}H NMR (400 MHz, $CDCl_3$) δ 7.59 (m, 4H), 7.30 – 7.16 (m, 6H), 7.14 – 7.02 (m, 4H), 6.89 – 6.84 (m, 1H), 5.04 (s, 2H), 4.68 (s, 2H). ppm. **^{13}C{^{1}H} NMR** (101 MHz, $CDCl_3$) δ = 151.8, 147.2, 138.9, 128.8, 128.3, 128.1, 126.9, 125.3, 123.0, 106.8. **FT-IR** (KBr, ATR, cm^{-1}): ṽ = 3056 (w), 3026 (w),1685 (w),1620 (w),1609 (m), 1594 (m), 1573 (m), 1492 (s), 1445 (w), 1331 (m), 1319 (s), 1302 (m), 1286 (m), 1252 (w), 1133 (w), 1076 (w), 1026 (w), 914 (w), 858 (w), 788 (w), 770 (s), 756 (m), 731 (w), 696 (s), 661 (w), 641 (w), 617 (w), 601 (w), 585 (w).

Spectroscopic data matches the literature.[19]

Compound **6b** was synthesized according to the above described procedure. The NMR yield was determined *via* comparison of the integral of the alkyne starting material at 3.00 ppm with respect to the vinyl groups of the amine product at 4.55 ppm and 4.96 ppm. NMR yield: 56%.

Compound **6c** was synthesized according to the above described procedure. The NMR yield was determined *via* comparison of the integral of the alkyne starting material at 3.00 ppm with respect to the vinyl groups of the amine product at 4.39 ppm and 4.98 ppm. NMR yield: 36%.

Spectroscopic data were compared to other similar compounds.[19,20]

1.6. Synthesis of the 1,2-dihydroquinolines and enyne cyclization

Synthesis of 1,2-Dimethyl-2,4-diphenyl-1,2-dihydroquinoline

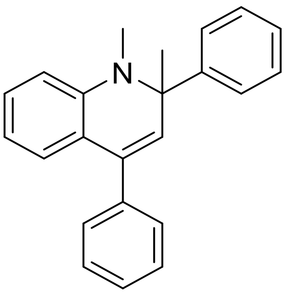

$Y_{Mes}PCy_2$•AuCl (17 mg, 20 µmol) and $NaBAr^F_4$ (18 mg, 20 µmol), were dissolved in 0.5 ml of *N*-Methylanilline (5 mmol, 1 eq.) and subsequently 2.2 ml Phenylacetylene (20 mmol, 4 eq.) were added to this solution. The solution was stirred at 70 °C for 48 h. The contents of the flask were directly distilled through Kugelrohr distillation at $1 \cdot 10^{-3}$ mbar. After a small forerun at 40 °C the product was collected at 180 °C as a red viscous oil which solidified upon standing. The solid was recrystallized from dry hexane to yield the title compound as a colourless solid. (1 g, 3.2 mmol, 64%)

^{1}H NMR (400 MHz, $CDCl_3$) δ = 7.55 – 7.46 (m, 2H), 7.34 – 7.20 (m, 7H), 7.20 – 7.13 (m, 1H), 7.11 – 7.05 (m, 1H), 6.86 (dd, *J* = 7.7, 1.7 Hz, 1H), 6.50 (m, 2H), 5.25 (s, 1H), 2.53 (s, 3H), 1.71 (s, 3H). **^{13}C{^{1}H} NMR** (101 MHz, $CDCl_3$) δ = 147.7, 145.2, 139.6, 133.9, 130.5, 129.5, 129.2, 128.4, 128.3, 127.4, 127.0, 126.9, 126.0, 121.1, 116.1, 110.3, 63.5, 33.0, 23.3. **FT-IR** (KBr, ATR, cm^{-1}): ṽ = 3058 (w), 3030 (w), 2998 (w), 2970 (w) 2820 (w), 1641 (m), 1590 (m), 1564 (w), 1487 (s), 1443 (m), 1177 (m), 1166 (m), 1027 (m), 589 (m), 777 (m), 762 (vs), 754 (vs), 745 (cs), 735 (s), 700 (vs), 606 (m), 505 (m). **m.p.:** 96.2°C.

Spectroscopic data matches the literature.[21]

Synthesis of 1-Ethyl-2,6-dimethyl-2,4-diphenyl-1,2-dihydroquinoline

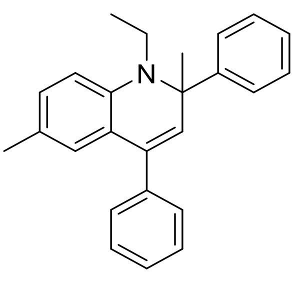

$Y_{Mes}PCy_2$•AuCl (4 mg, 5 µmol), $NaBAr^F_4$ (4 mg, 5 µmol), were dissolved in 93 mg of *N*-Ethyl-*p*-toluidin (0.68 mmol, 1 eq.) and subsequently 0,43 ml Phenylacetylene (3.98 mmol, 6 eq.) were added to this solution. The solution was stirred at 70°C for 48 h. The contents of the flask were directly distilled through Kugelrohr distillation at $1 \cdot 10^{-3}$ mbar. After a small forerun at 40°C the product was collected at 190 °C as a orange viscous oil which solidified upon standing. The solid was recrystallized from dry acetonitrile to yield the title compound as a colourless solid. (142 mg, 0.42 mmol, 62%)

^{1}H NMR (400 MHz, $CDCl_3$) δ = 7.62 – 7.44 (m, 2H), 7.32 (tt, J = 7.4, 2.5 Hz, 6H), 7.29 – 7.14 (m, 1H), 7.06 – 6.87 (m, 2H), 6.72 (d, J = 2.2 Hz, 1H), 6.47 (d, J = 8.3 Hz, 1H), 5.26 (s, 1H), 3.05 (q, $^3J_{HH}$ = 7.0 Hz, 2H), 2.13 (s, 3H), 1.78 (s, 3H), 1.05 (t, $^3J_{HH}$ = 7.0 Hz, 3H) ppm. **^{13}C{^{1}H} NMR** (101 MHz, $CDCl_3$) δ = 148.1, 141.6, 139.9, 133.9, 130.7, 129.7, 129.2, 128.3, 128.3, 127.3, 127.1, 127.0, 124.5, 121.2, 110.4, 63.8, 40.4, 24.9, 20.4, 13.7 ppm.

Compound **7b** was synthesized according to the above described procedure. The NMR yield was determined *via* comparison of the integral of the alkyne starting material at 3.00 ppm with respect to the CH-group of the cyclic product product at 5.13 ppm. NMR yield: 72%.

Catalysis of 1,6 En-Yne-cyclization

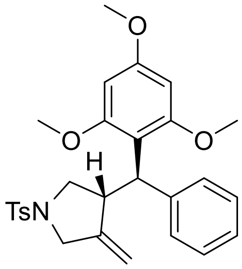

$Y_{Mes}PCy_2$•AuCl (1 mg, 1 µmol), $NaBAr^F_4$ (1 mg, 1 µmol), *N*-cinnamyl-4-methyl-*N*-(prop-2-yn-1-yl)benzensulfonamide (210 mg, 645 µmol) and 1,3,5-trimethoxybenzene (325 mg, 1.94 mmol, 3 eq.) were added to a schlenk tube. 1 ml DCM was added and the solution was stirred at room temperature for 4 h. An NMR spectrum was recorded to confirm complete conversion. The yield was calculated by integration of the peak for the alkyne starting material with respect to the peak for the cyclic product in the ^{1}H NMR spectrum.

^{1}H NMR (200 MHz, $CDCl_3$) δ = 7.73 – 7.57 (m, 2H), 7.41 – 7.28 (m, 4H), 7.24 – 7.01 (m, 3H), 6.07 (s, 2H), 4.71 (m, 1H), 4.45 (s, 1H), 4.36 (m, 1H), 4.01 (m, 1H), 3.92 (m, 1H), 3.85 (m, 1H), 3.77 (s, 3H) 3.74 (s, 6H), 3.41 – 3.21 (m, 1H), 2.79 (dd, *J* = 9.6, 7.9 Hz, 1H), 2.44 (s, 3H) ppm.

Spectrospcopic data match the literature.[5,22]

1.7. Detailled results of the catalytic screenings

Table 1.1: Full results of the catalytic screening

**Catalyst**	1•AuCl	2•AuCl	3•AuCl	4•AuCl	4•AuCl	4•AuCl	4•AuCl	5•AuCl	5•AuCl	5•AuCl	5•AuCl	5•AuCl	5•AuCl
**Loading**	0.1 mol%	0.1 mol%	0.1 mol%	0.1 mol%	0.05 mol%	0.01 mol%	0.1 mol%	0.1 mol%	0.05 mol%	0.01 mol%	0.1 mol%	0.01 mol%	0.005 mol%
**Temp.**	50°C	50°C	50°C	50°C	50°C	70°C	25°C	50°C	50°C	50°C	25°C	70°C	70°C
**0 h**	0	0	0	0	0	0	0	0	0	0	0	0	0
**0.5 h**	1.64	0.99	1.64	71.5	41.4	23.7	65.9	73.0	57.2	22.7	43.8	43.4	26.0
**1 h**	3.54	2.28	2.28	86.4	62.0	32.0	81.2	84.1	75.0	37.1	56.5	56.3	37.4
**2 h**	5.97	3.85	2.91	92.9	74.1	34.4	91.6	93.2	84.6	45.0	69.1	63.2	45.8
**3 h**	6.83	5.67	3.85	95.8	77.1	36.3	93.9	95.9	87.8	49.6	76.9	68.3	49.2
**4 h**	9.10	7.41	4.46	98.4	79.0	39.1	95.3	97.7	90.7	53.8	81.9	70.1	50.9
**5 h**	10.2	9.01	5.66	98.8	80.5	40.8	96.5	98.2	92.3	56.5	85.6	71.6	52.3
**24 h**	20.8	42.0	16.7	99.9	85.4	46.5	99.0	100	97.5	64.6	98.7	75.9	61.1
**48 h**	29.1	72.2	19.6	100	-	-	-	-	-	-	-	-	69.1
**72 h**	31.5	89.2	24.1	-	-	-	-	-	-	-	-	-	75.6

Table 1.2: Full results of the catalytic screening of the literature known compounds

**Catalyst**	PPh_3•AuCl	IPr•AuCl	IPr•AuCl	IPr•AuCl	^{Cy}JohnPhos•AuCl	^{Cy}JohnPhos•AuCl	^{Cy}JohnPhos•AuCl
**Loading**	0.1 mol%	0.1 mol%	0.05 mol%	0.01 mol%	0.1 mol%	0.05 mol%	0.01 mol%
**Temp.**	50°C	50°C	50°C	50°C	50°C	50°C	50°C
**0 h**	0	0	0	0	0	0	0
**0.5 h**	9	33	19	1	67	23	3
**1 h**	15	46	28	2	81	31	5
**2 h**	19	67	44	3	95	49	11
**3 h**	22	75	51	6	96	58	15
**4 h**	24	83	56	8	97	65	17
**5 h**	26	86	59	8	98	66	20
**24 h**	39	93	78	18	99	72	26

1.8. Procedure for measurement of the IR spectra

IR spectra were recorded on a Nicolet iS5 FT-IR in transmission mode with a Specac "Omni-cell" with KBr plates and a 0.1 mm spacer at 22 °C or with an ATR unit.

Procedure for $\tilde{\nu}(CO)_{Rh}$ determination: 5.00 mg (19.4 µmol) Rh(acac)(CO_2) were dissolved in 1 mL of DCM in a glovebox. 19.4 µmol of the phosphine were added to the solution and the solution was stirred for 15 min until gas evolution ceased. The solution was added into the IR cell using a syringe. The cell was closed, taken outside the glovebox and an IR spectrum was recorded. The TEP value was calculated *via* the linear correlation of TEP and $\tilde{\nu}(CO)_{Rh}$ described by Carrow.[23]

Table 1.3: Comparison of the determined $\tilde{\nu}(CO)_{Rh}$ and the corresponding calculated TEP values.

**Compound**	**$\tilde{\nu}(CO)_{Rh}$**	**calcd. TEP**
$Y_{Ph}PCy_2$ (1)	1948,9	2052,5
$Y_{pOMe}PCy_2$ (2)	1947,2	2051,5
$Y_{pCF3}PCy_2$ (3)	1952,6	2054,6
$Y_{oTol}PCy_2$ (4)	1947,5	2051,7

2. NMR Spectra

2.1. NMR spectra of the isolated ligands and gold complexes

<u>$Y_{Ph}PCy_2$ (1)</u>

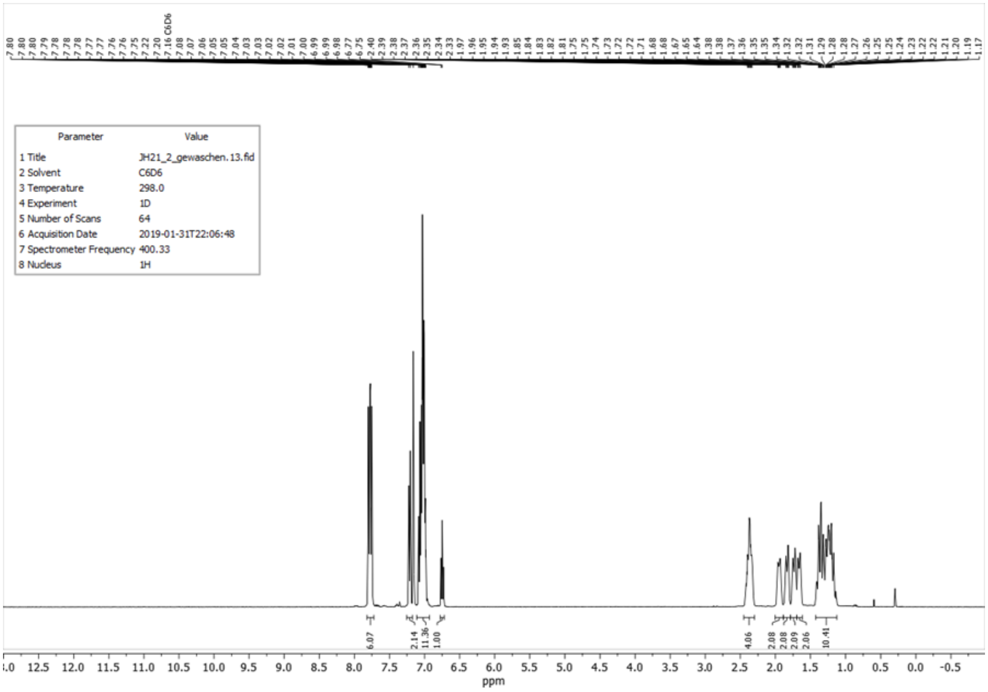

Figure 2.1: ^{1}H NMR spectrum of $Y_{Ph}PCy_2$ (**1**).

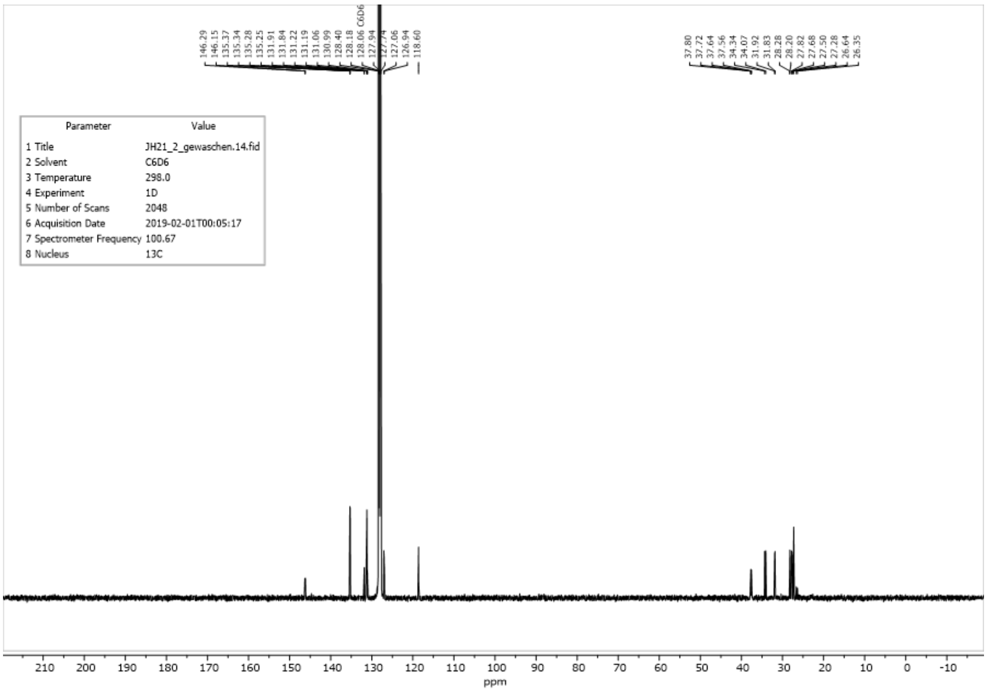

Figure 2.2: $^{13}C\{^1H\}$ NMR spectrum of $Y_{Ph}PCy_2$ (**1**).

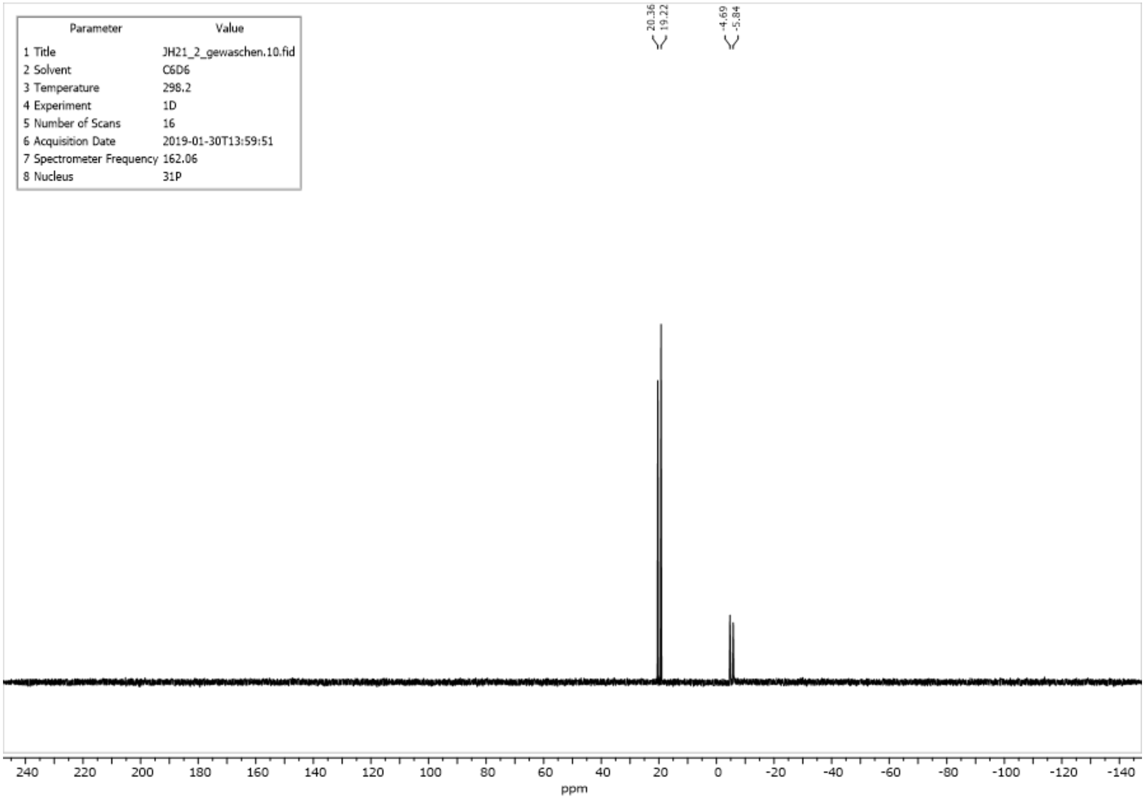

Figure 2.3: $^{31}P\{^{1}H\}$ NMR spectrum of $Y_{Ph}PCy_2$ (**1**).

<u>$Y_{Ph}PCy_2$•AuCl (1•AuCl)</u>

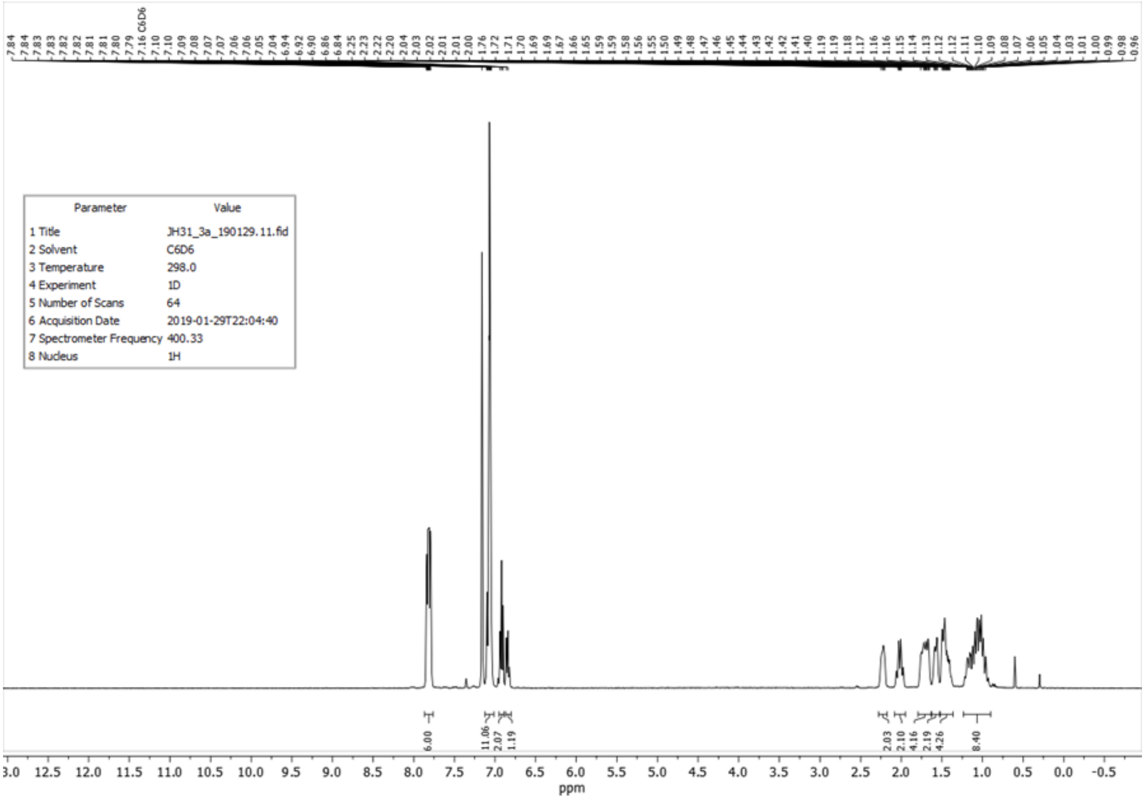

*Figure 2.4: ^{1}H NMR spectrum of $Y_{Ph}PCy_2$•AuCl (**1•AuCl**).*

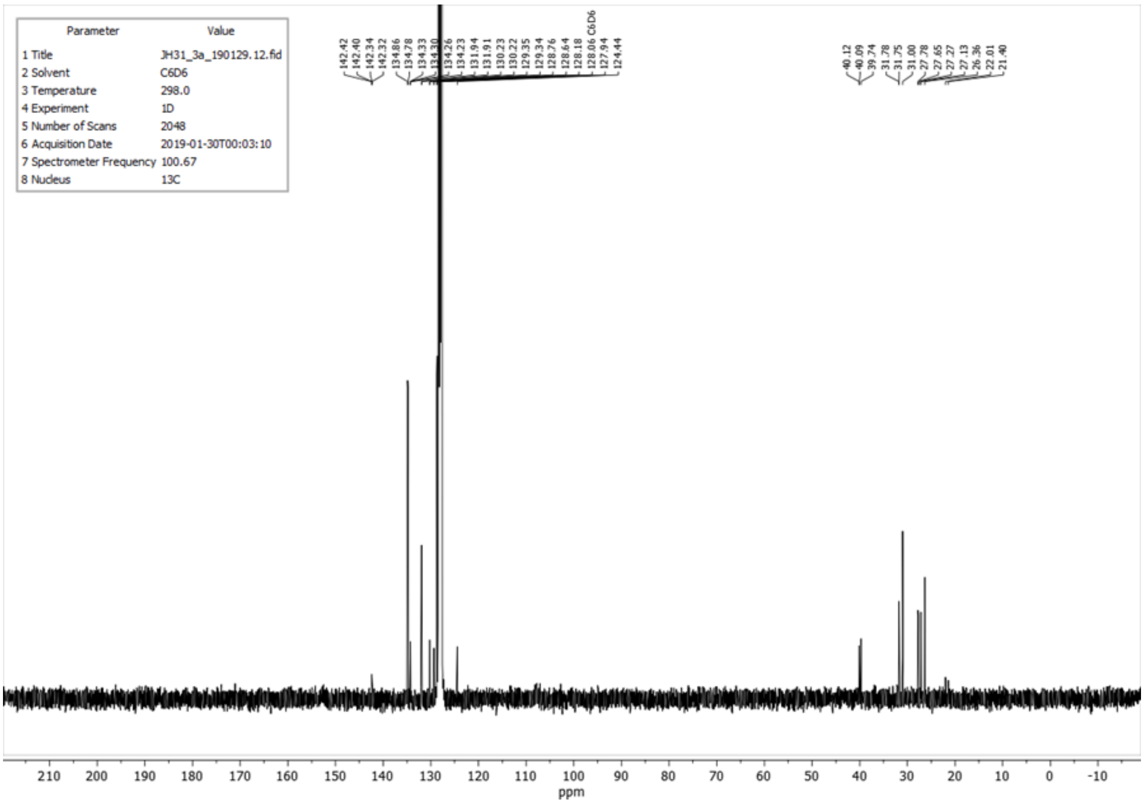

Figure 2.5: $^{13}C\{^{1}H\}$ NMR spectrum of $Y_{Ph}PCy_2$•AuCl (**1•AuCl**).

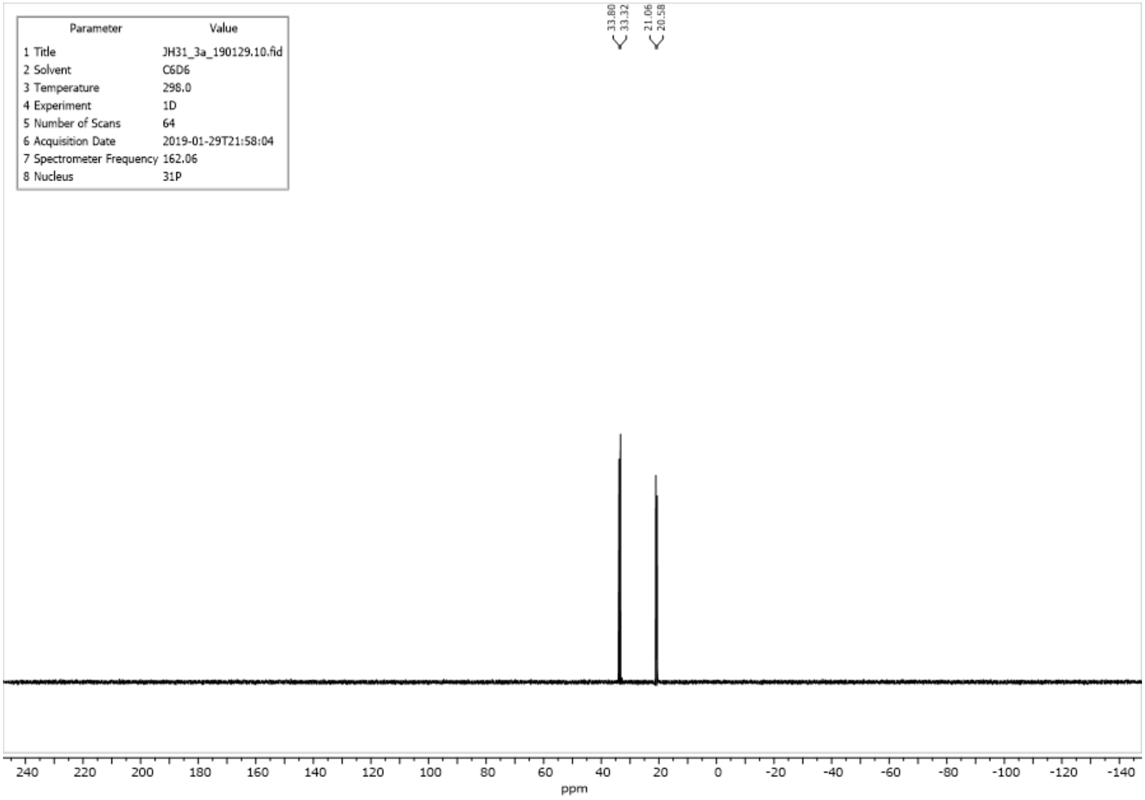

Figure 2.6: $^{31}P\{^{1}H\}$ NMR spectrum of $Y_{Ph}PCy_2$•AuCl (**1•AuCl**).

$Y_{pOMe}PCy_2$ (**2**)

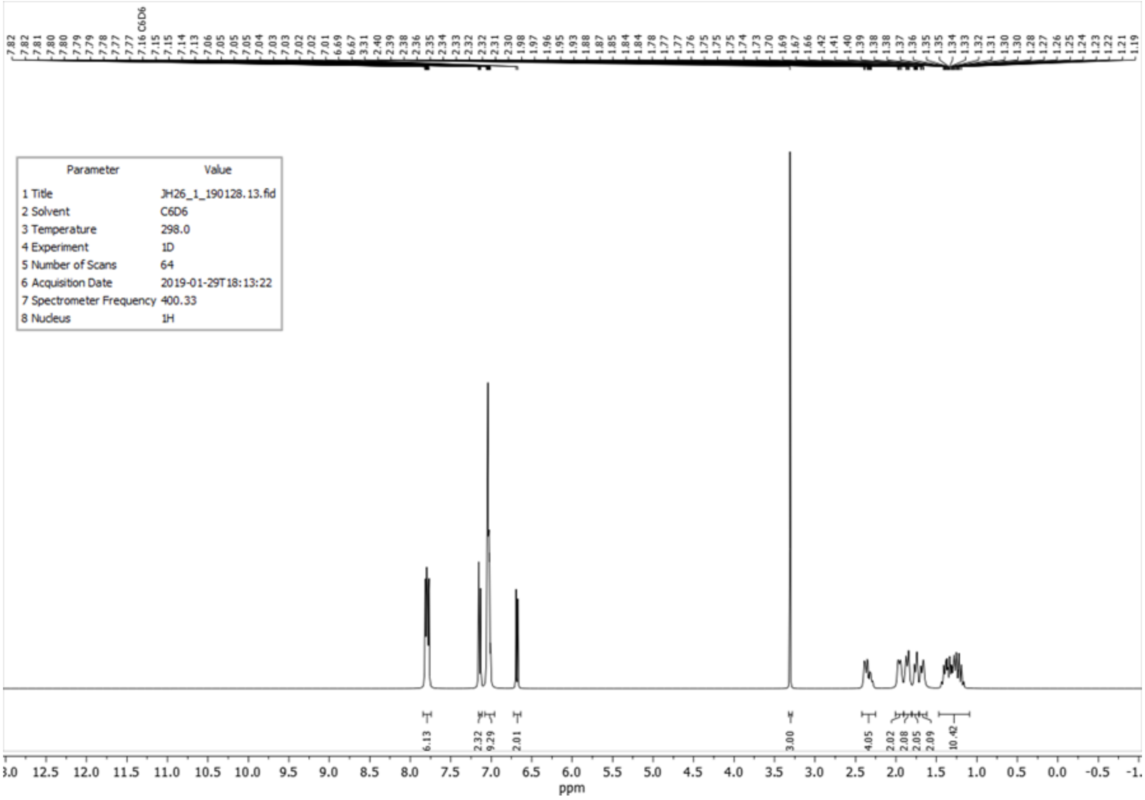

Figure 2.7: ^{1}H NMR spectrum of $Y_{pOMe}PCy_2$ (**2**).

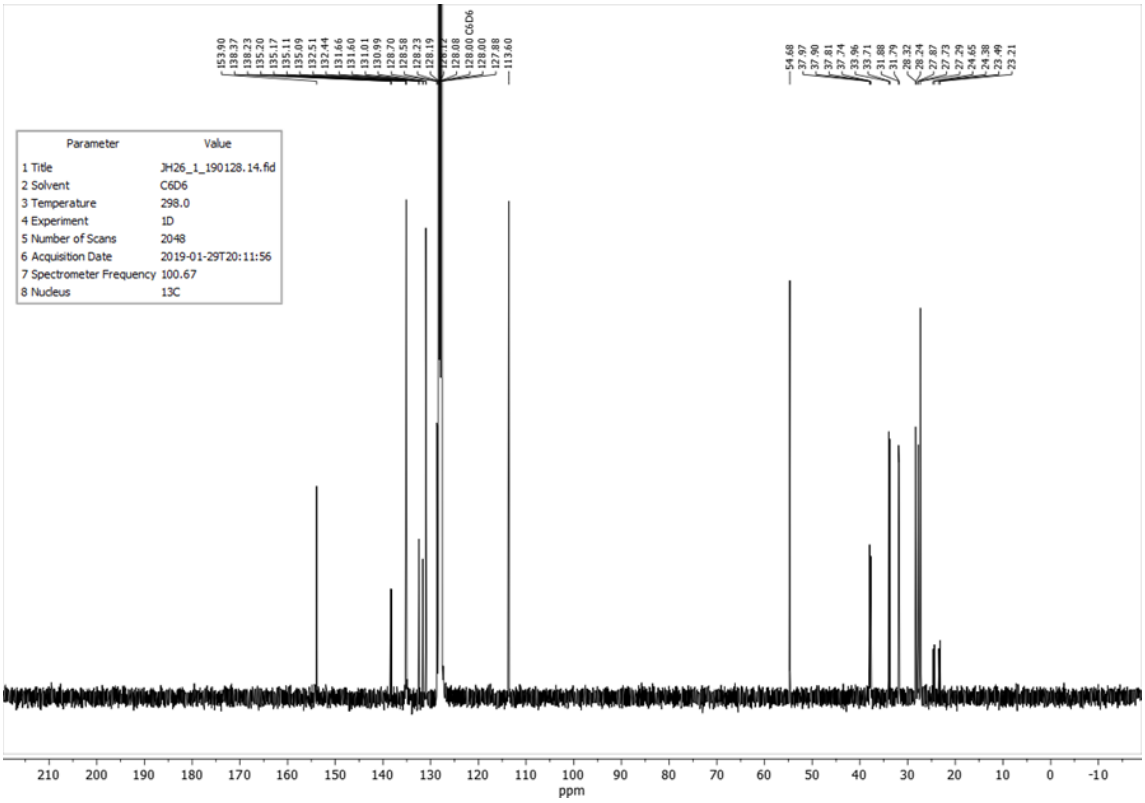

Figure 2.8: $^{13}C\{^{1}H\}$ NMR spectrum of $Y_{pOMe}PCy_2$ (**2**).

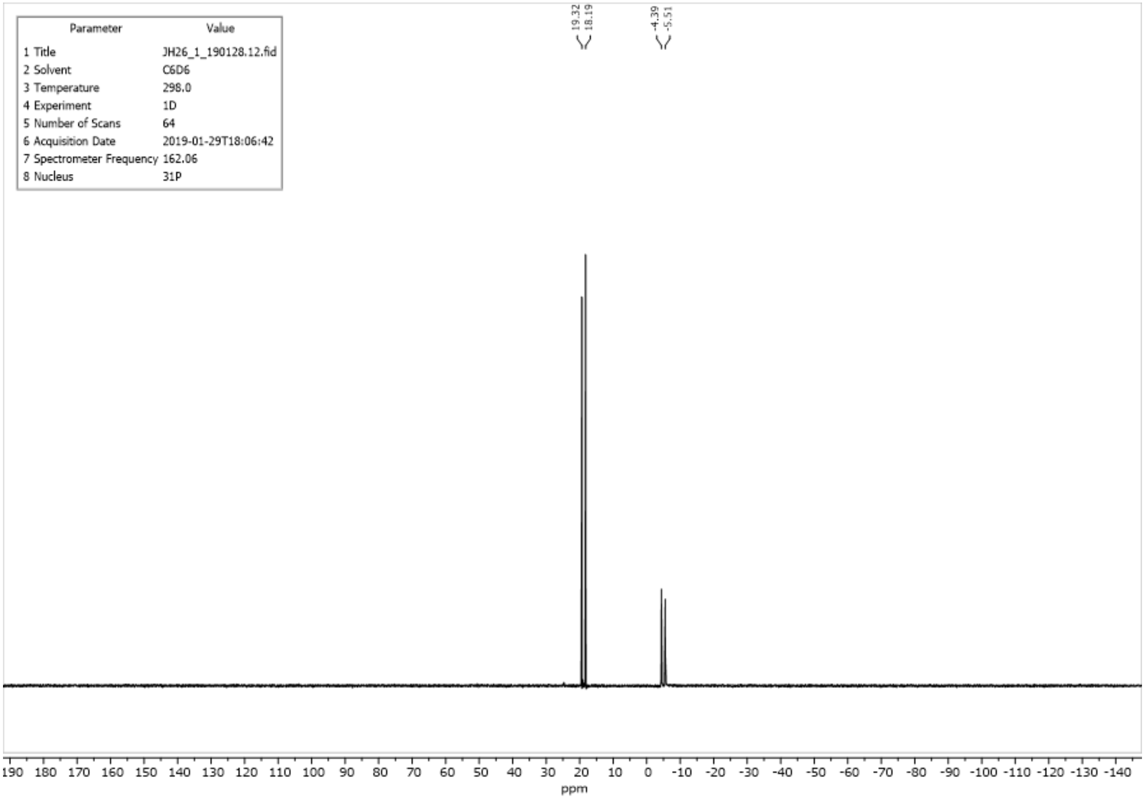

Figure 2.9: $^{31}P\{^1H\}$ NMR spectrum of $Y_{pOMe}PCy_2$ (**2**).

$Y_{pOMe}PCy_2$•AuCl (2•AuCl)

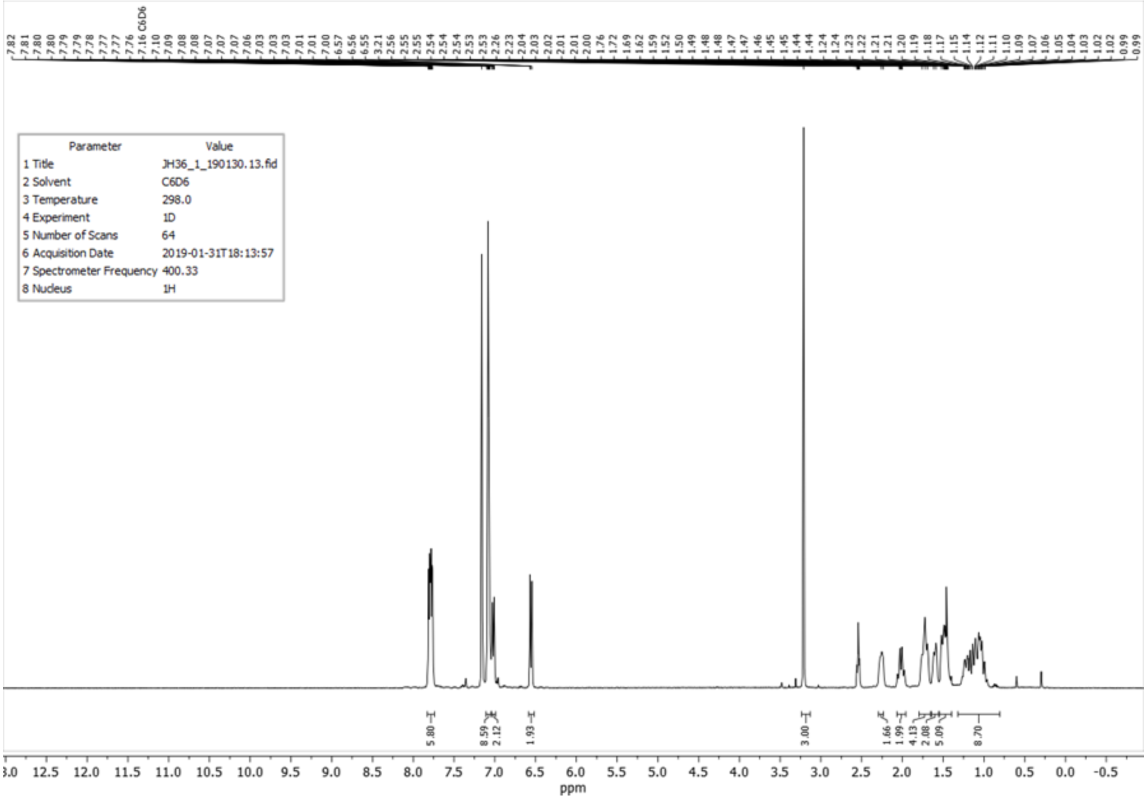

Figure 2.10: ^{1}H NMR spectrum of $Y_{pOMe}PCy_2$•AuCl (**2•AuCl**).

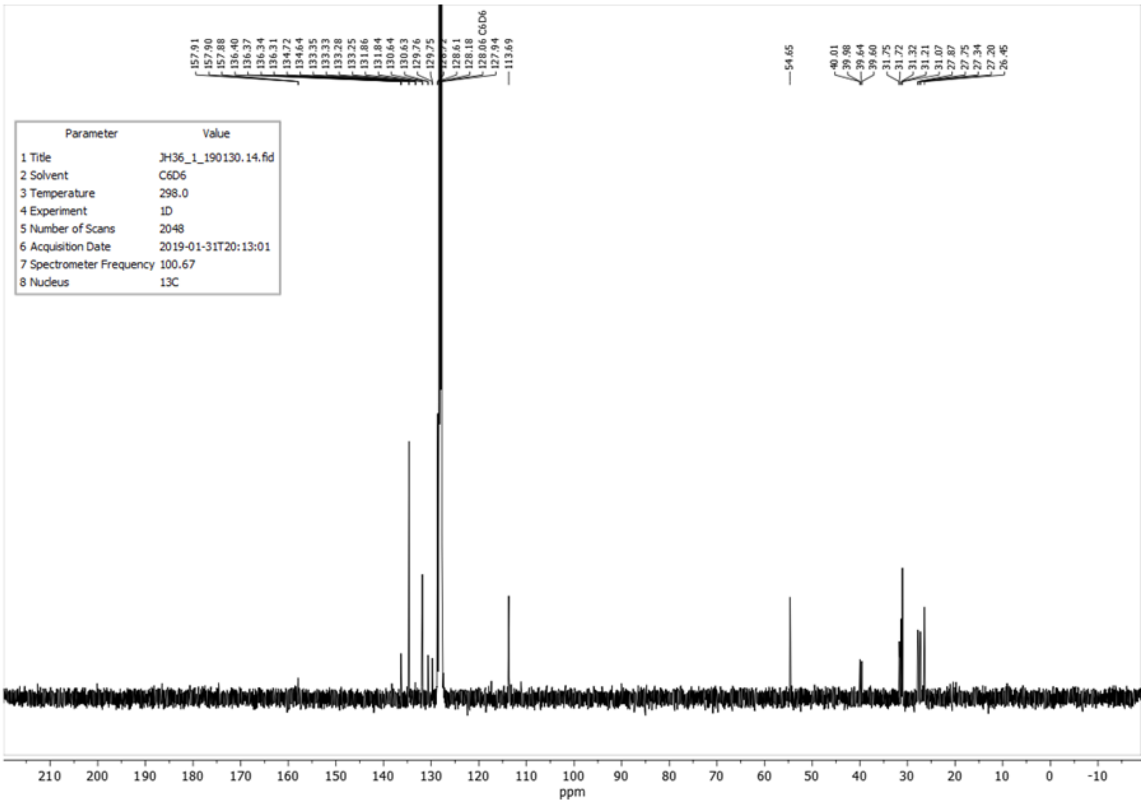

Figure 2.11: $^{13}C\{^1H\}$ NMR spectrum of $Y_{pOMe}PCy_2$•AuCl (**2•AuCl**).

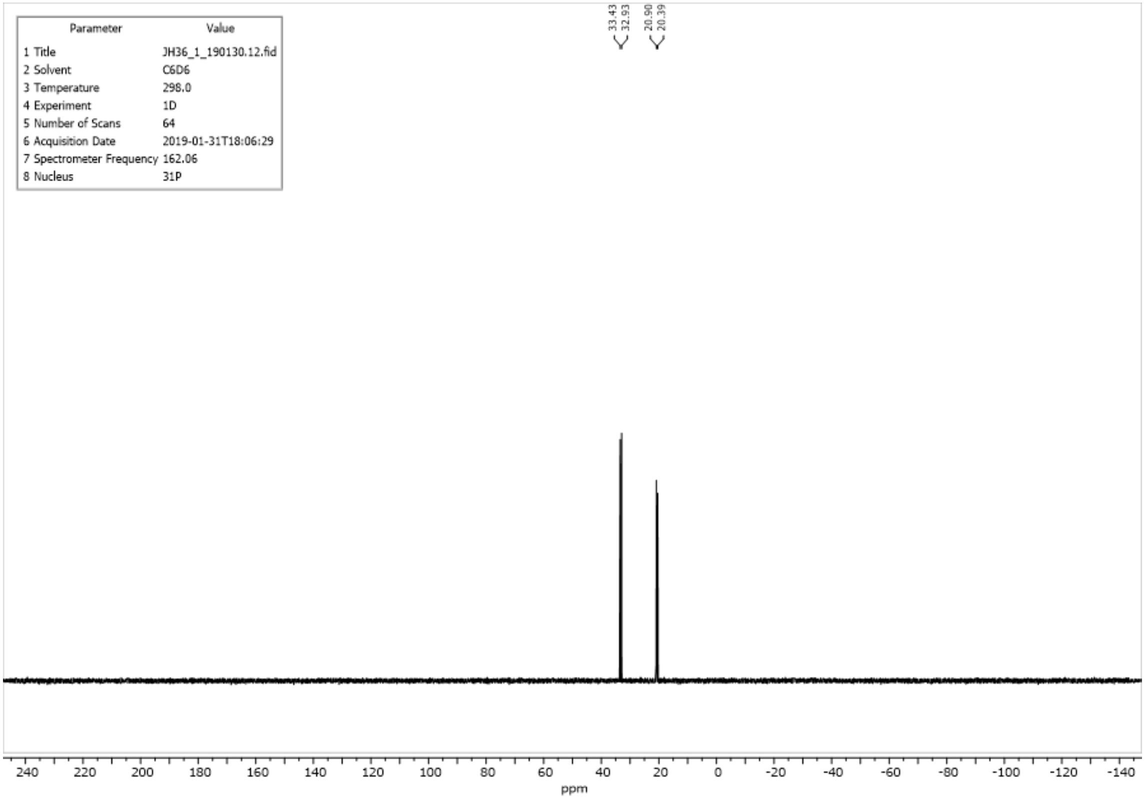

Figure 2.12: $^{31}P\{^1H\}$ NMR spectrum of $Y_{pOMe}PCy_2$•AuCl (**2•AuCl**).

Y$_{pCF3}$PCy$_2$ (3)

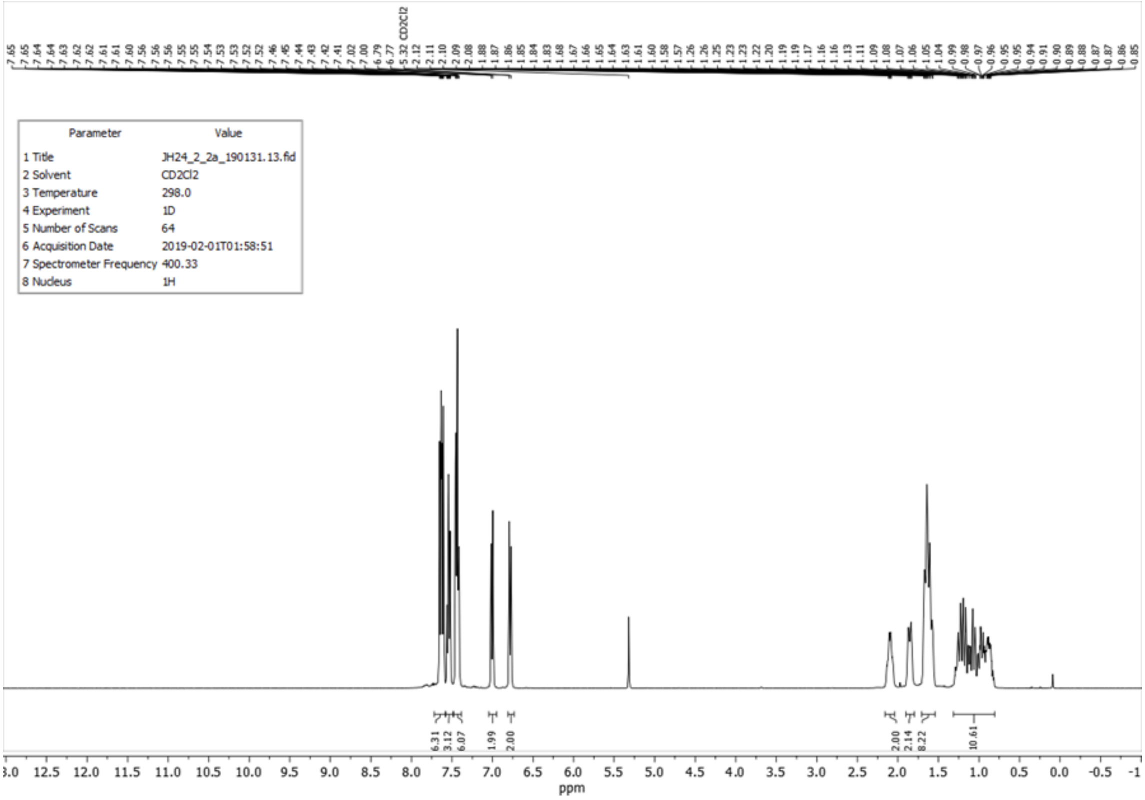

Figure 2.13: ^{1}H NMR spectrum of Y$_{pCF3}$PCy$_2$ (**3**).

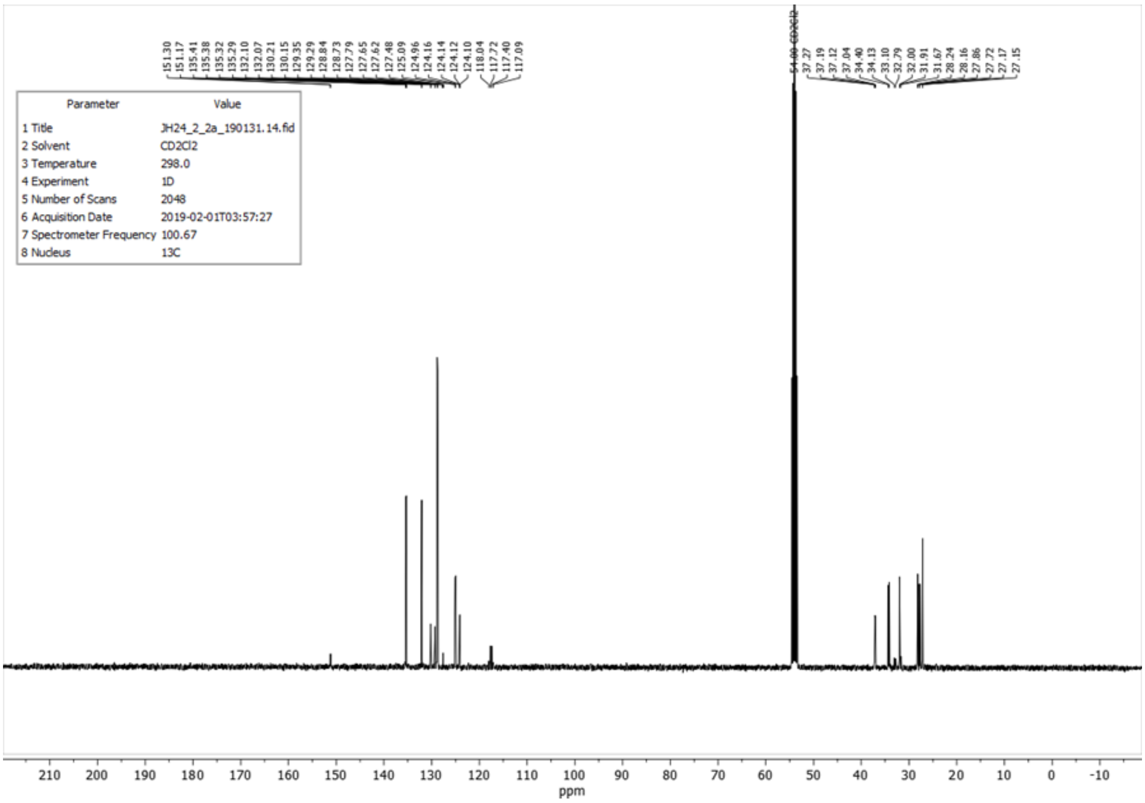

Figure 2.14: ^{13}C{^{1}H} NMR spectrum of Y$_{pCF3}$PCy$_2$ (**3**).

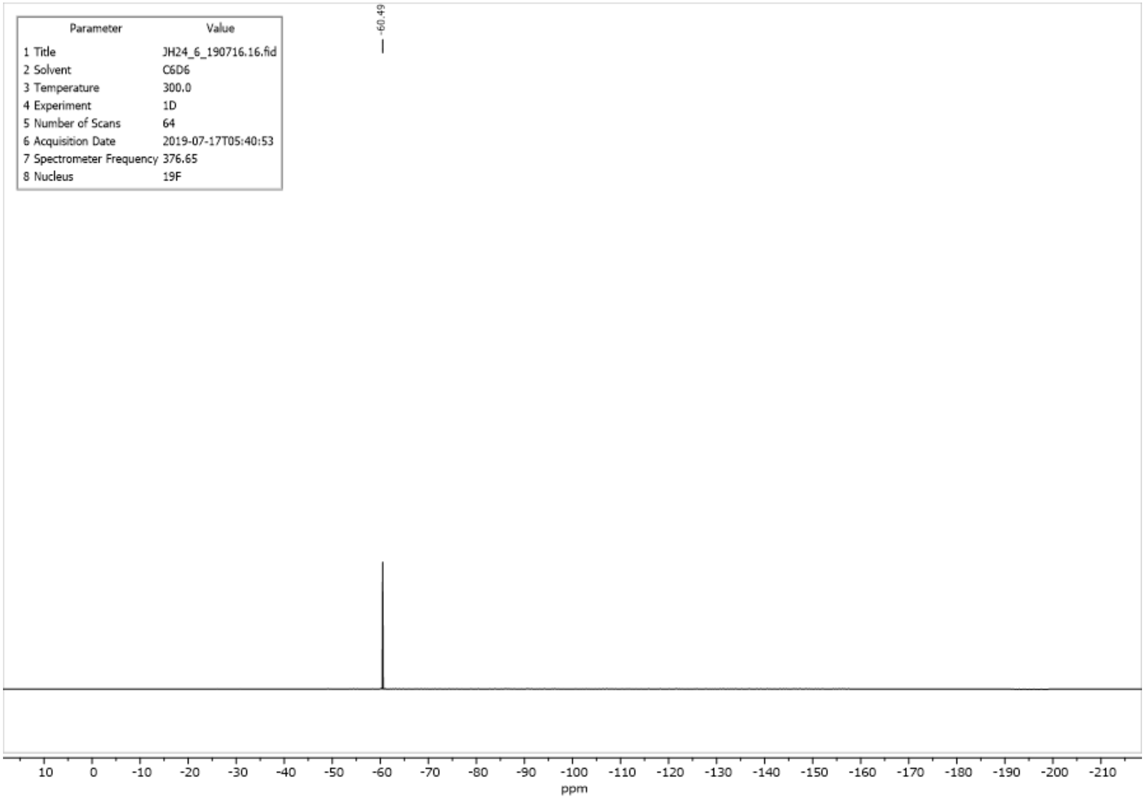

Figure 2.15: ^{19}F NMR spectrum of $Y_{pCF3}PCy_2$ (**3**).

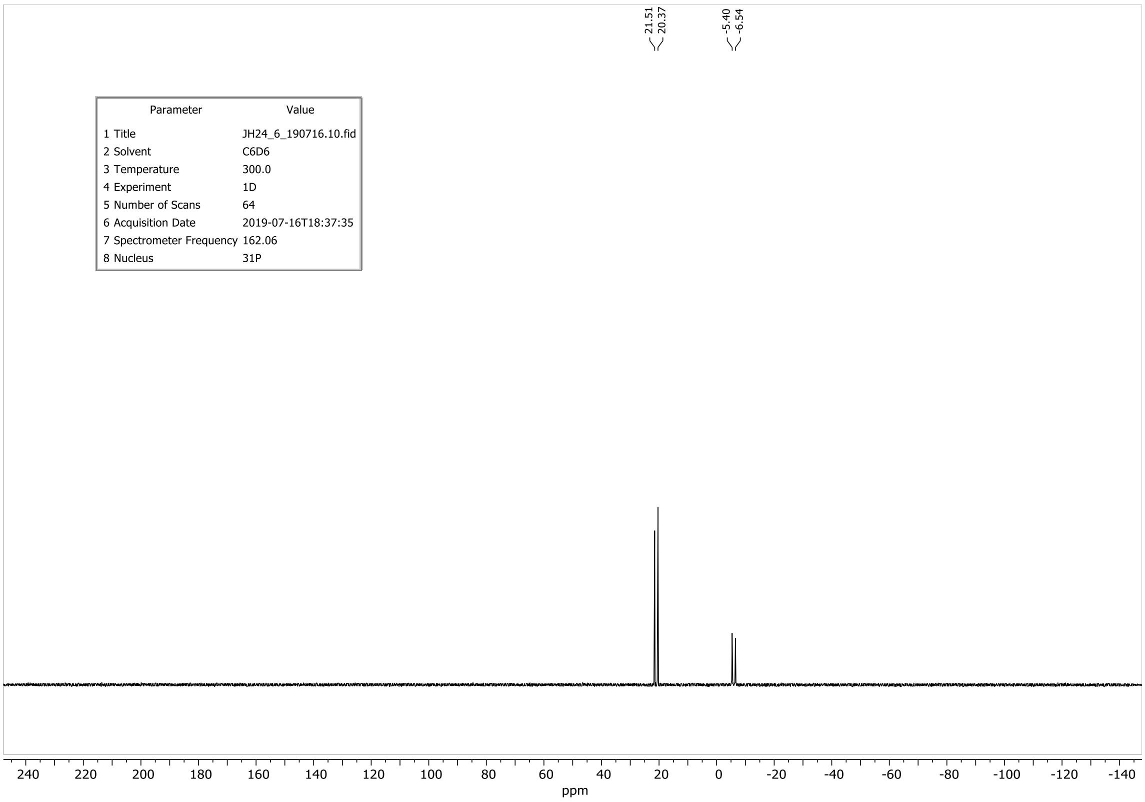

Figure 2.16: ^{31}P{^{1}H} NMR spectrum of $Y_{pCF3}PCy_2$ (**3**).

<u>$Y_{pCF3}PCy_2$•AuCl (3•AuCl)</u>

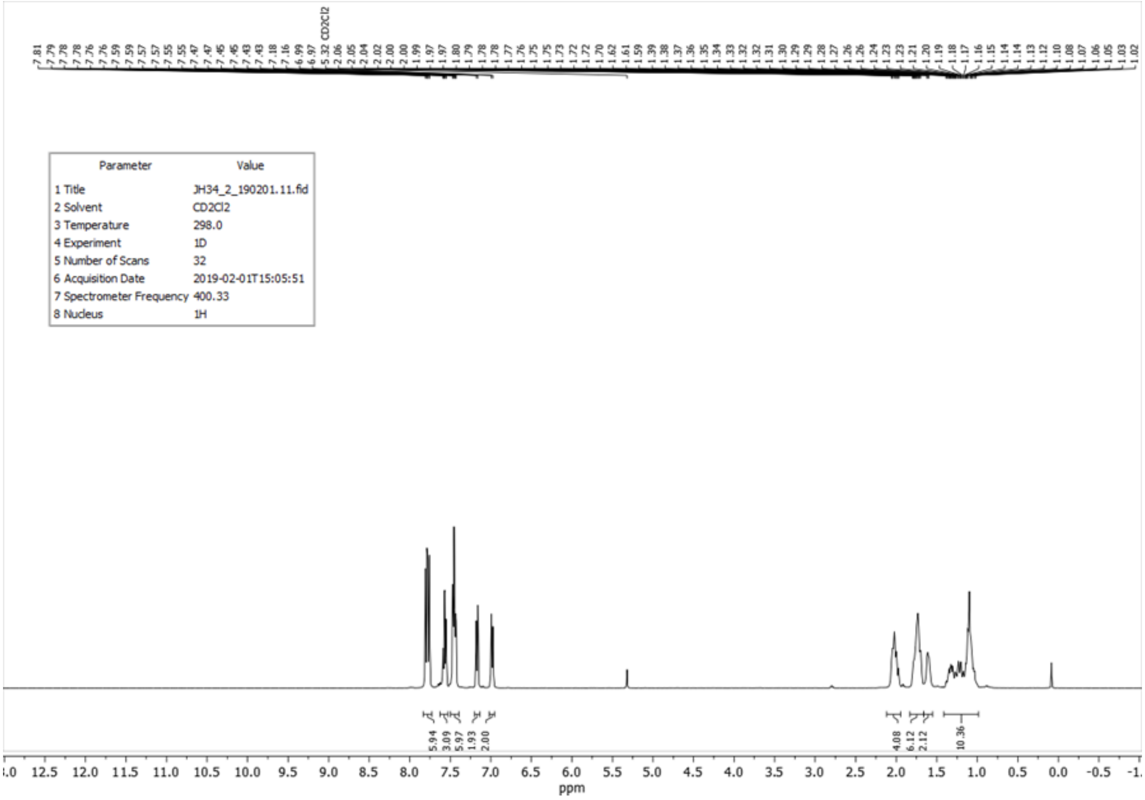

*Figure 2.17: ¹H NMR spectrum of $Y_{pCF3}PCy_2$•AuCl (**3•AuCl**).*

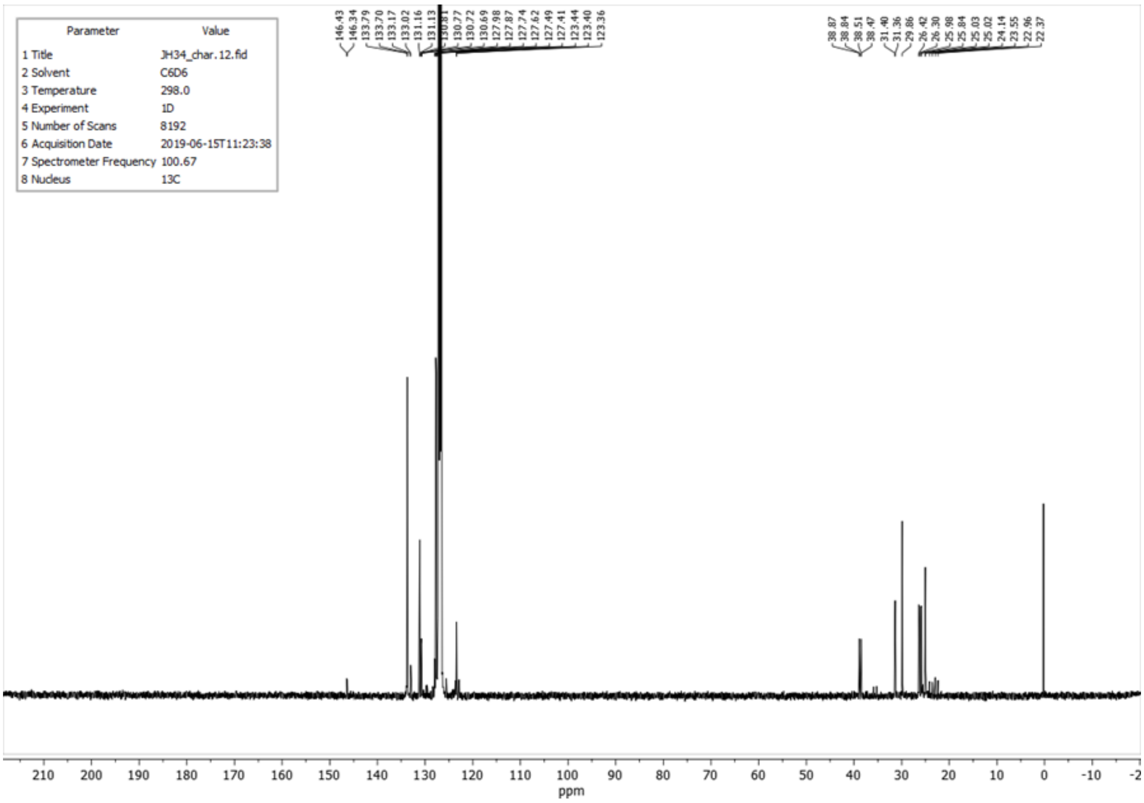

*Figure 2.18: ¹³C{¹H} NMR spectrum of $Y_{pCF3}PCy_2$•AuCl (**3•AuCl**).*

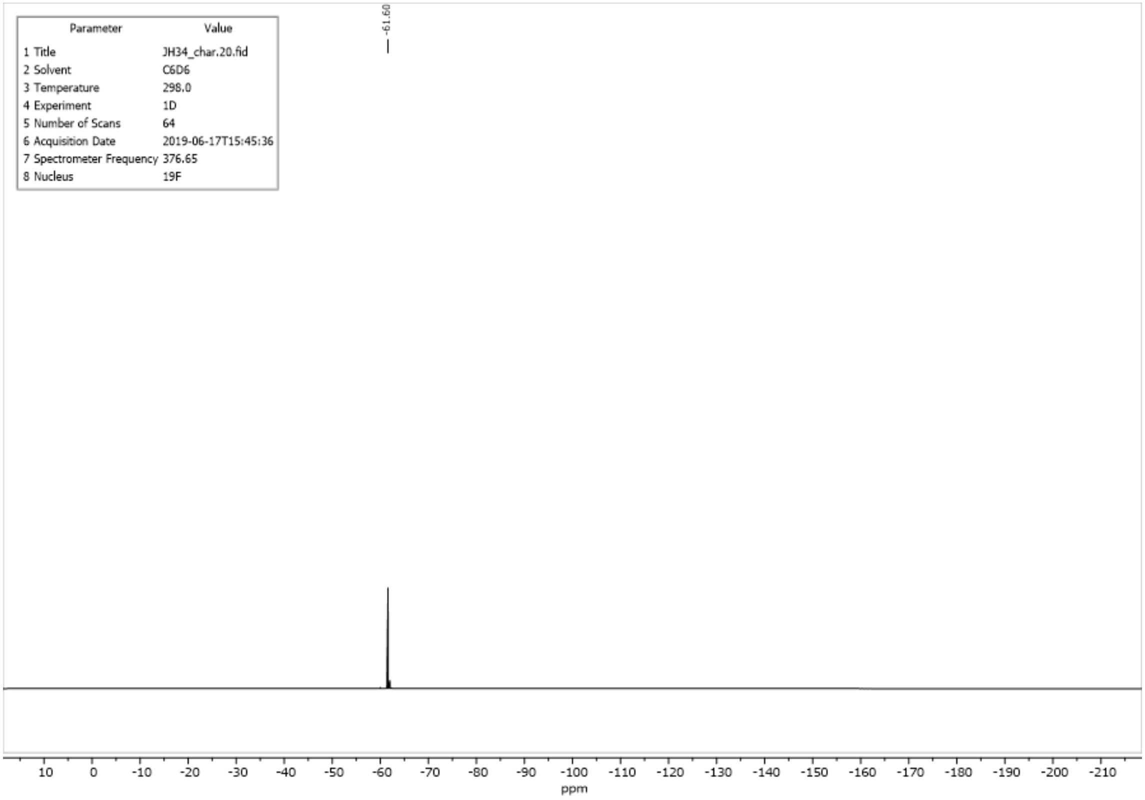

*Figure 2.19: ^{19}F NMR spectrum of $Y_{pCF3}PCy_2$•AuCl (**3•AuCl**).*

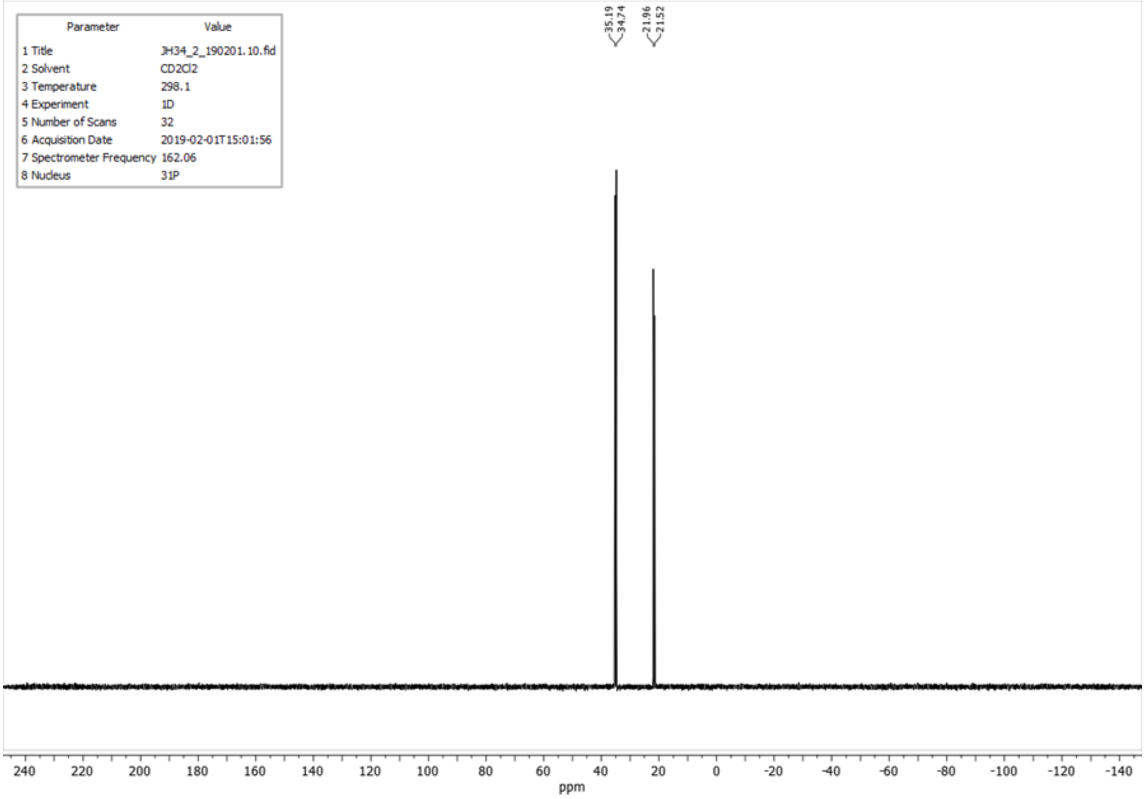

*Figure 2.20: $^{31}P\{^1H\}$ NMR spectrum of $Y_{pCF3}PCy_2$•AuCl (**3•AuCl**).*

$Y_{oTol}PCy_2$ (4)

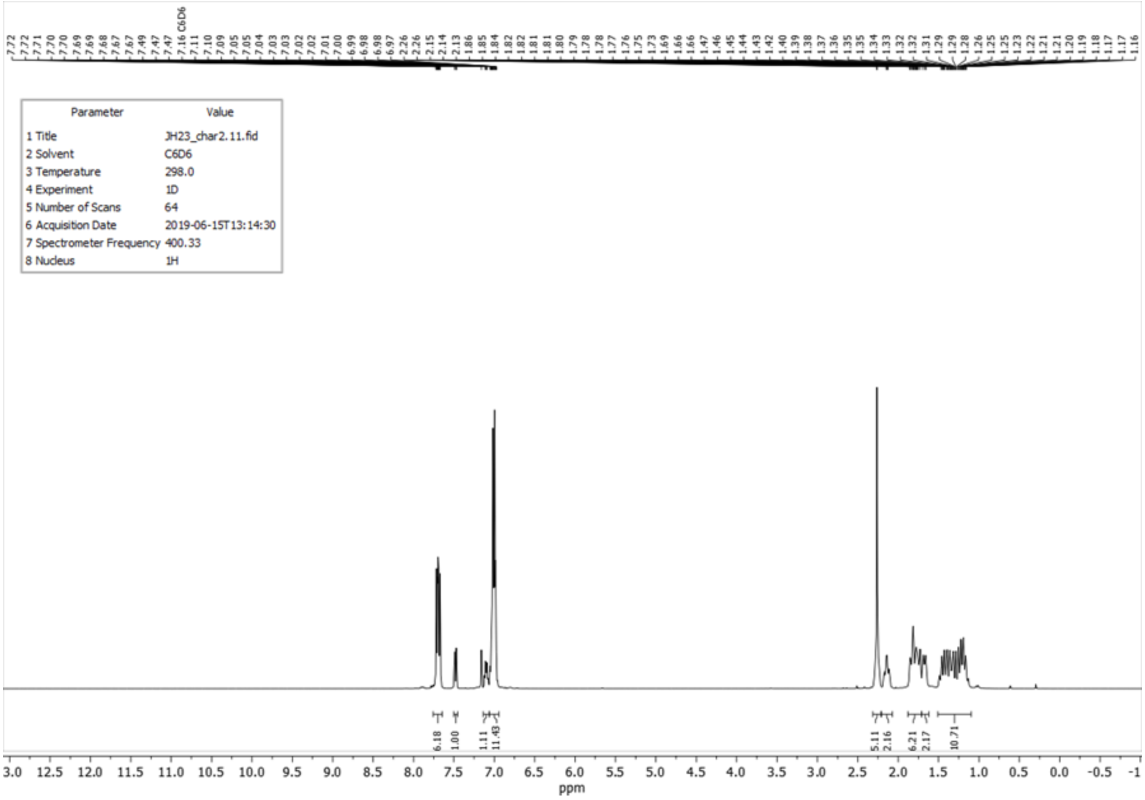

*Figure 2.21: ¹H NMR spectrum of $Y_{oTol}PCy_2$ (**4**).*

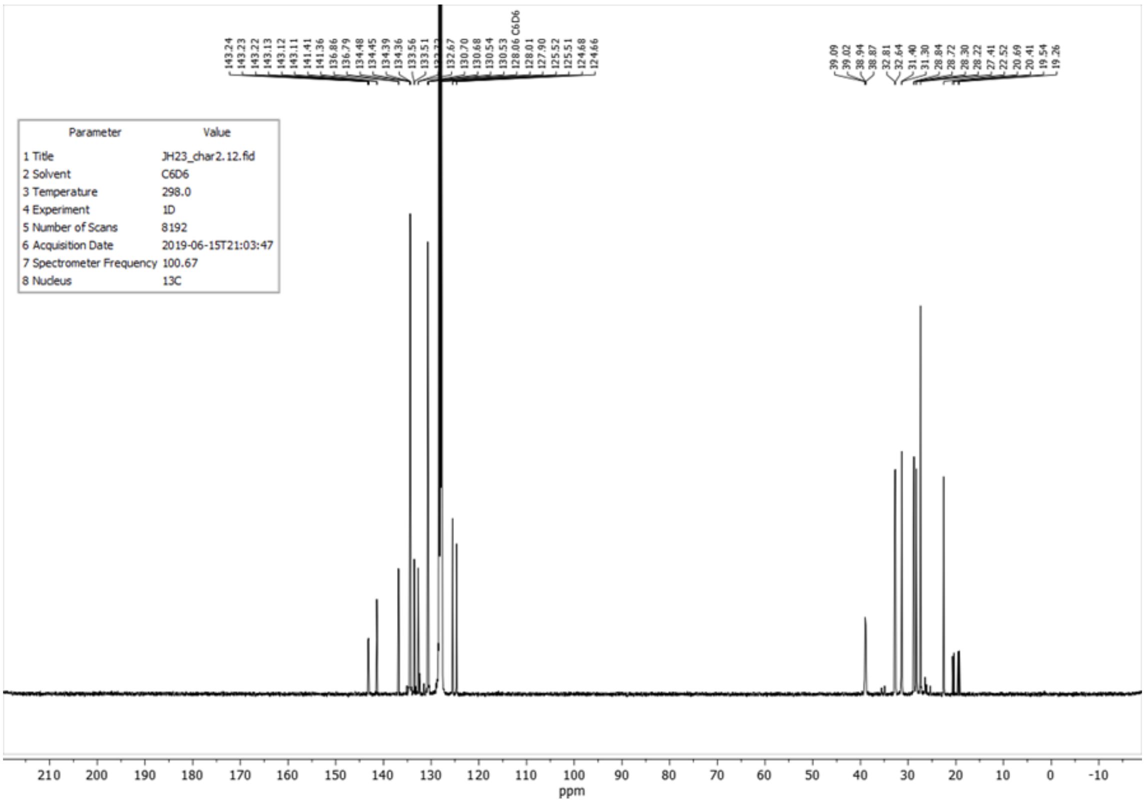

Figure 2.22: $^{13}C\{^1H\}$ NMR spectrum of $Y_{oTol}PCy_2$ (**4**).

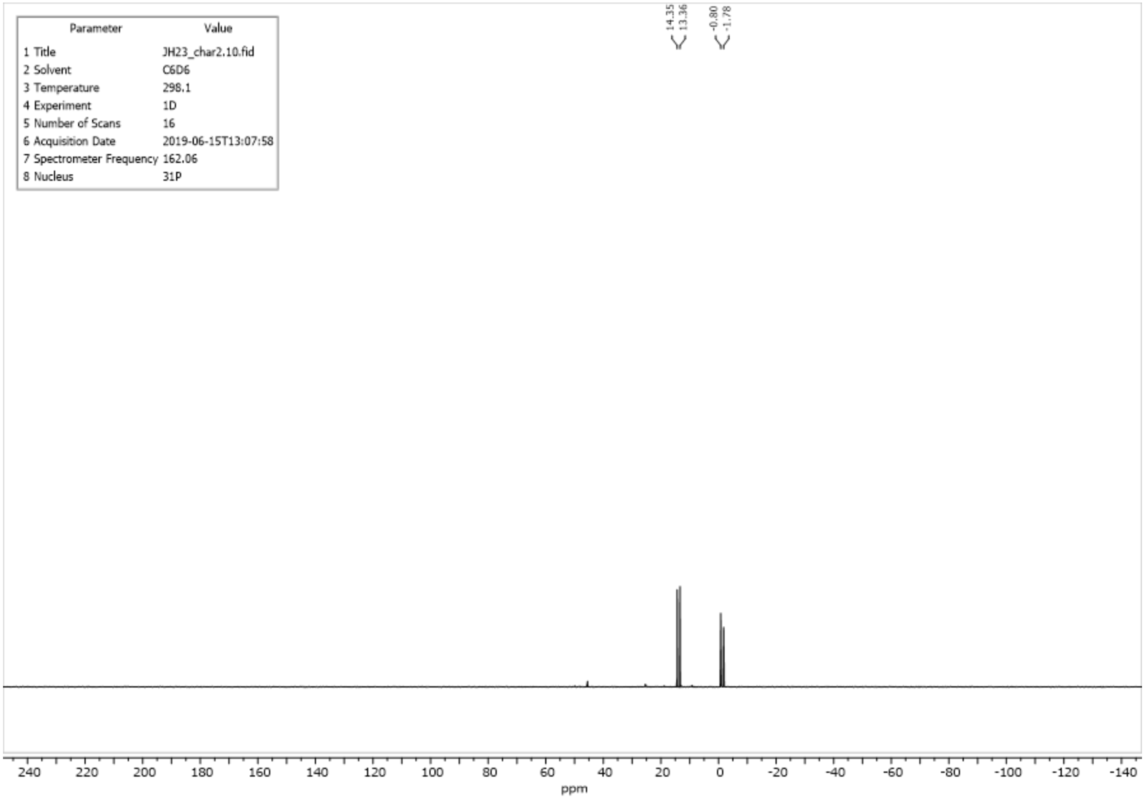

Figure 2.23: $^{31}P\{^{1}H\}$ NMR spectrum of $Y_{oTol}PCy_2$ (**4**).

<u>$Y_{oTol}PCy_2$•AuCl (4•AuCl)</u>

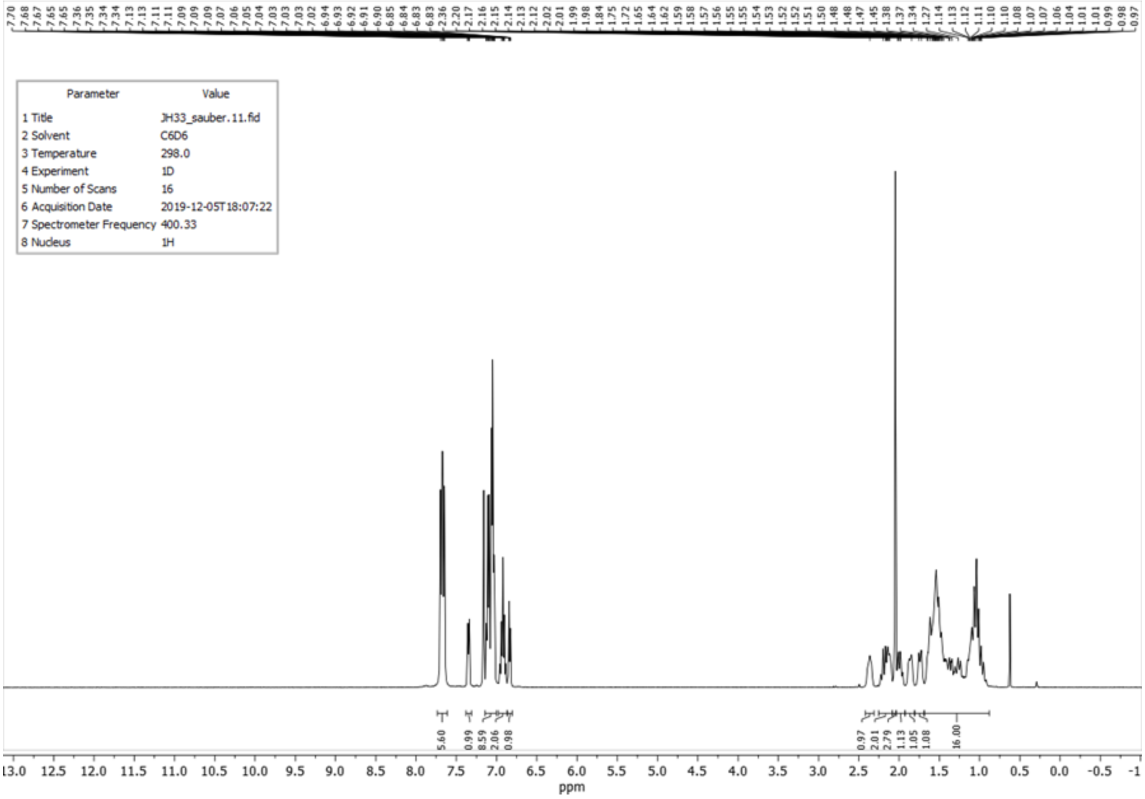

Figure 2.24: ^{1}H NMR spectrum of $Y_{oTol}PCy_2$•AuCl (**4•AuCl**).

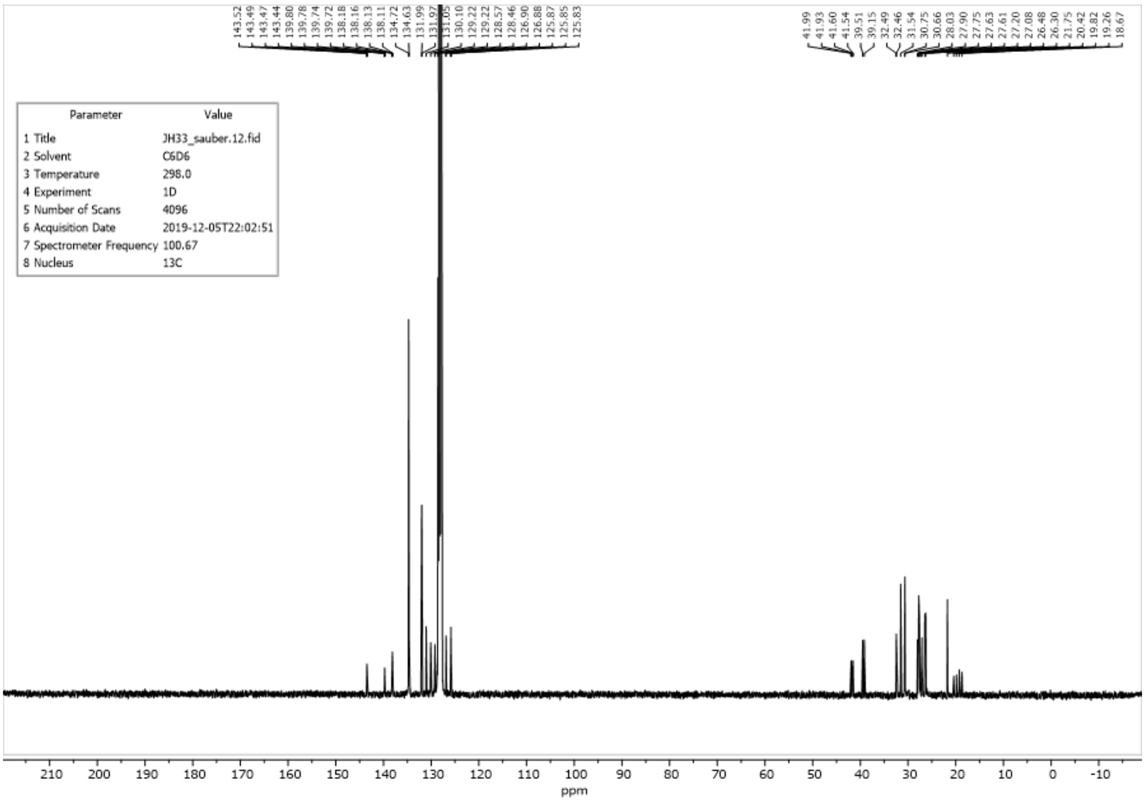

Figure 2.25: $^{13}C\{^1H\}$ NMR spectrum of $Y_{oTol}PCy_2$•AuCl (**4•AuCl**).

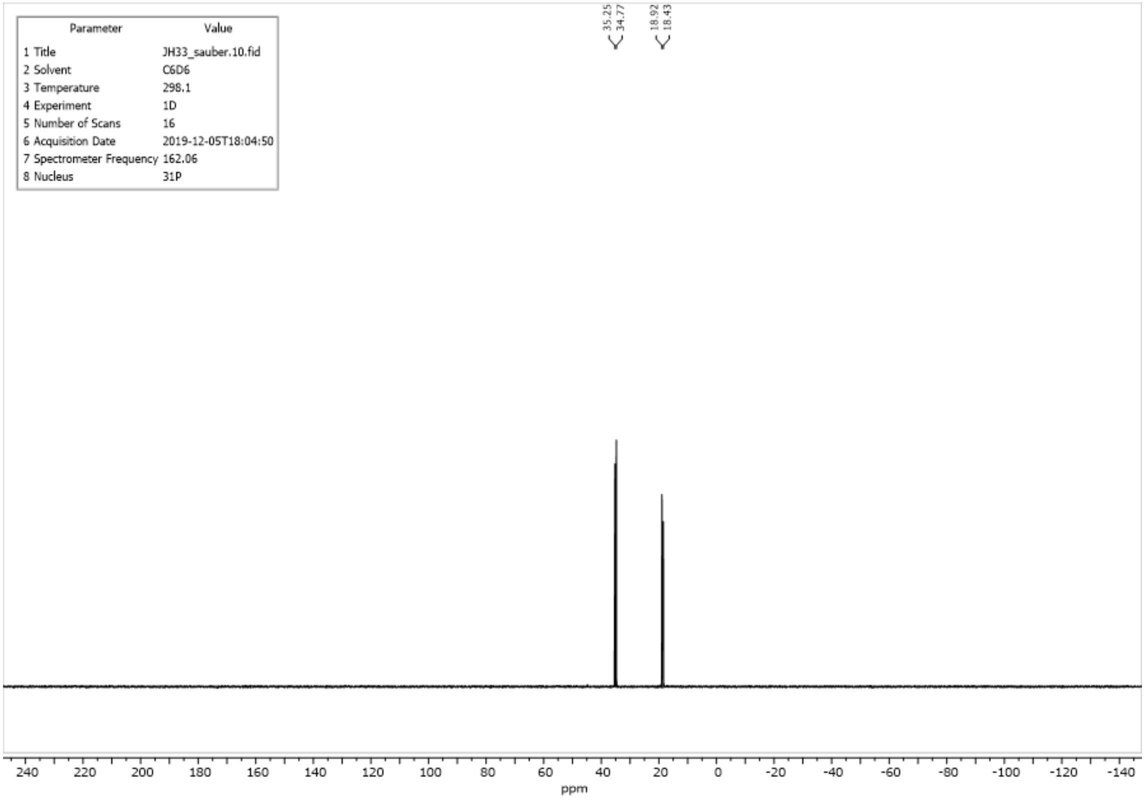

Figure 2.26: $^{31}P\{^1H\}$ NMR spectrum of $Y_{oTol}PCy_2$•AuCl (**4•AuCl**).

$Y_{Mes}H_2$

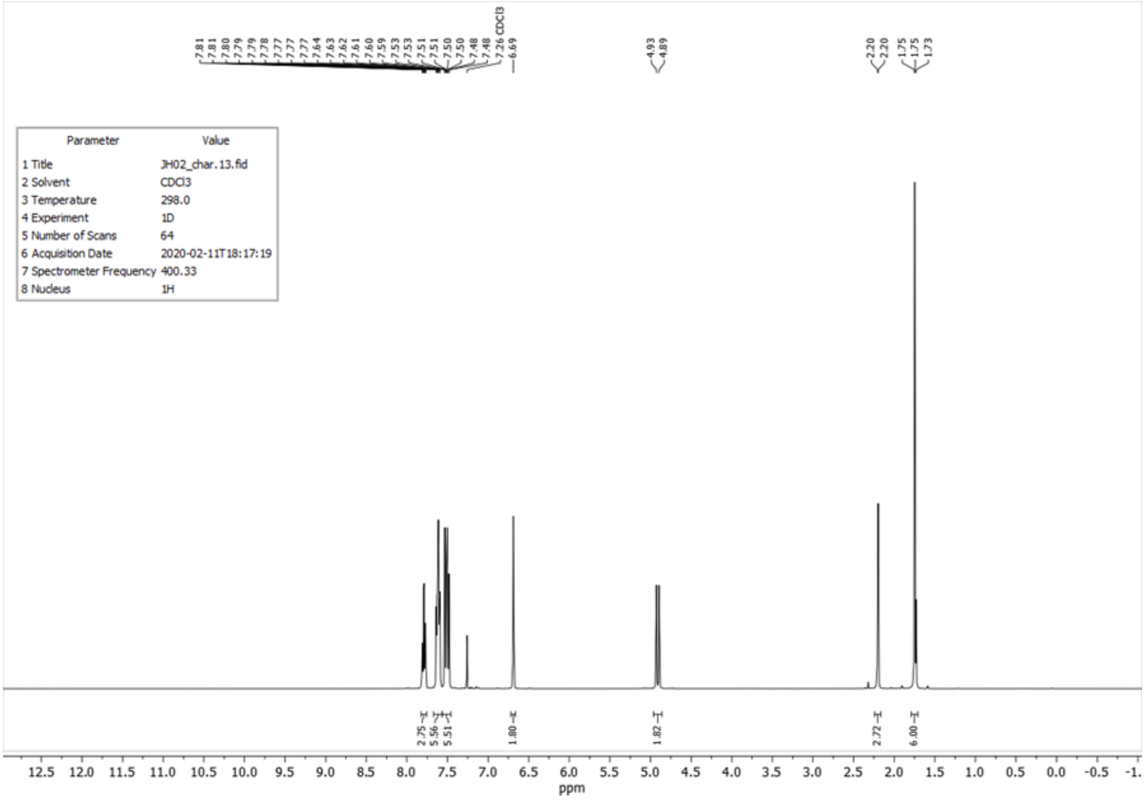

Figure 2.27: ^{1}H NMR spectrum of $Y_{Mes}H_2$.

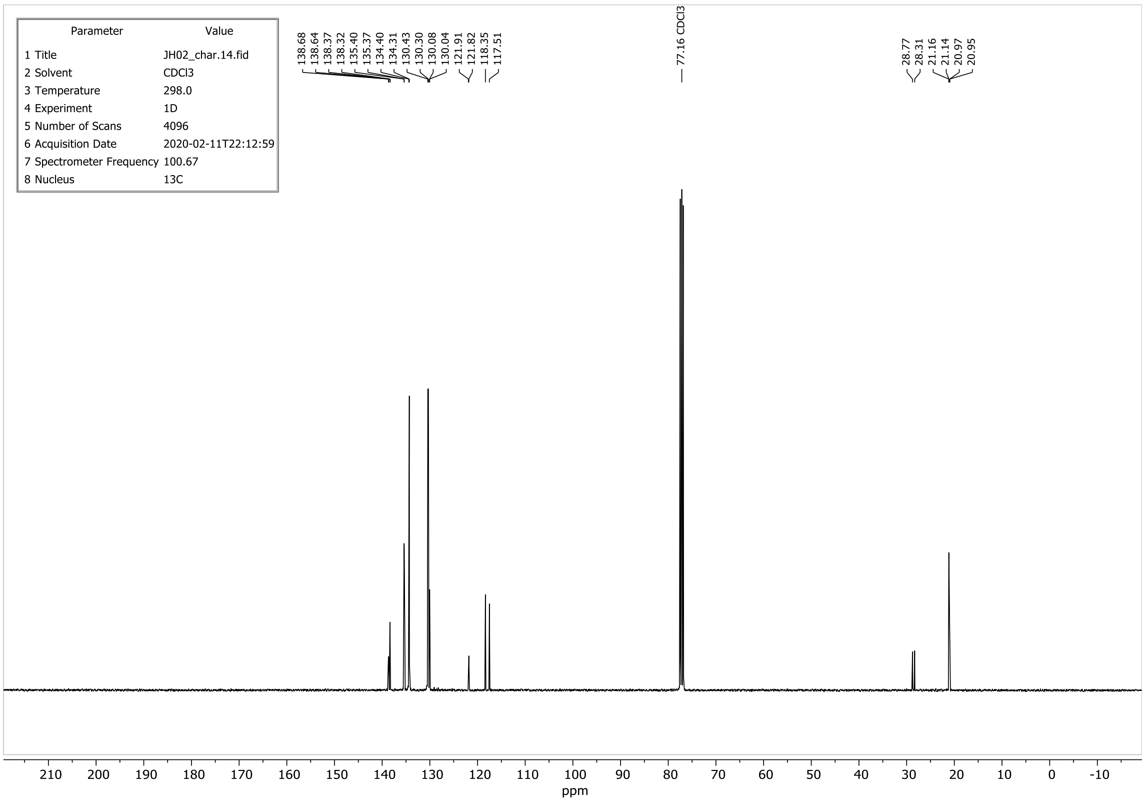

Figure 2.28: $^{13}C\{^{1}H\}$ NMR spectrum of $Y_{Mes}H_2$.

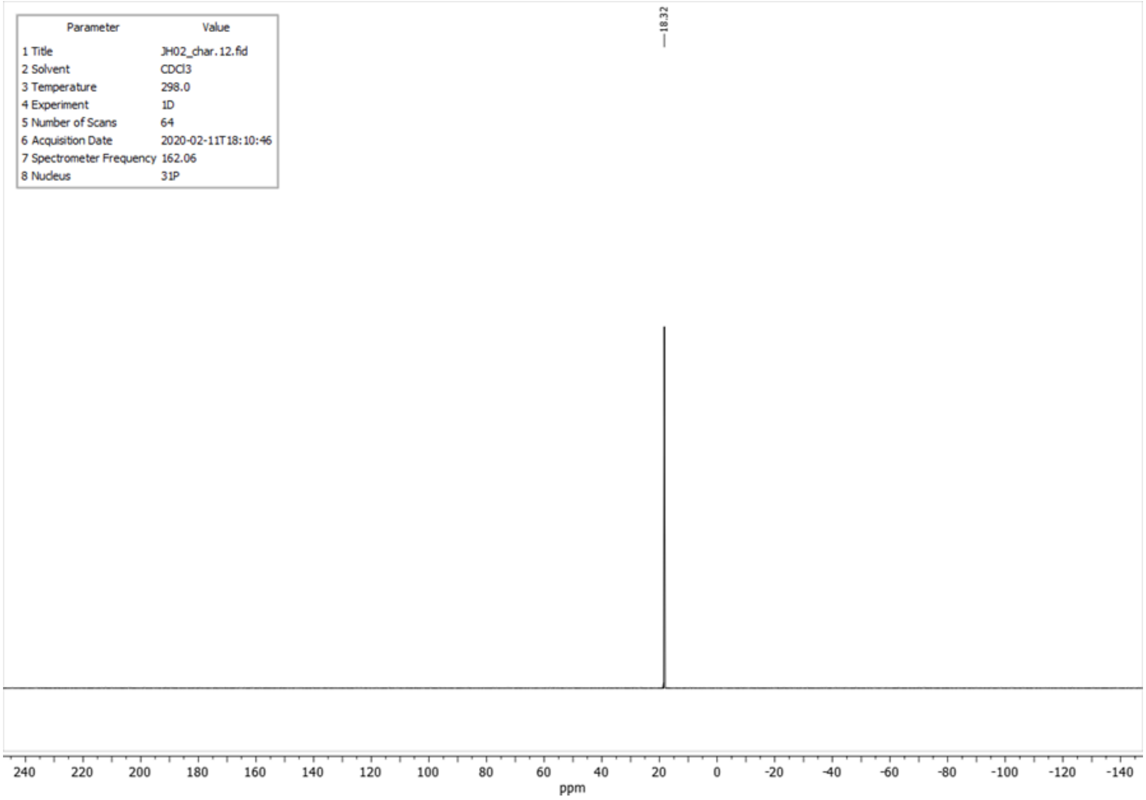

Figure 2.29: $^{31}P\{^1H\}$ NMR spectrum of $Y_{Mes}H_2$.

$Y_{Mes}PCy_2$ (5)

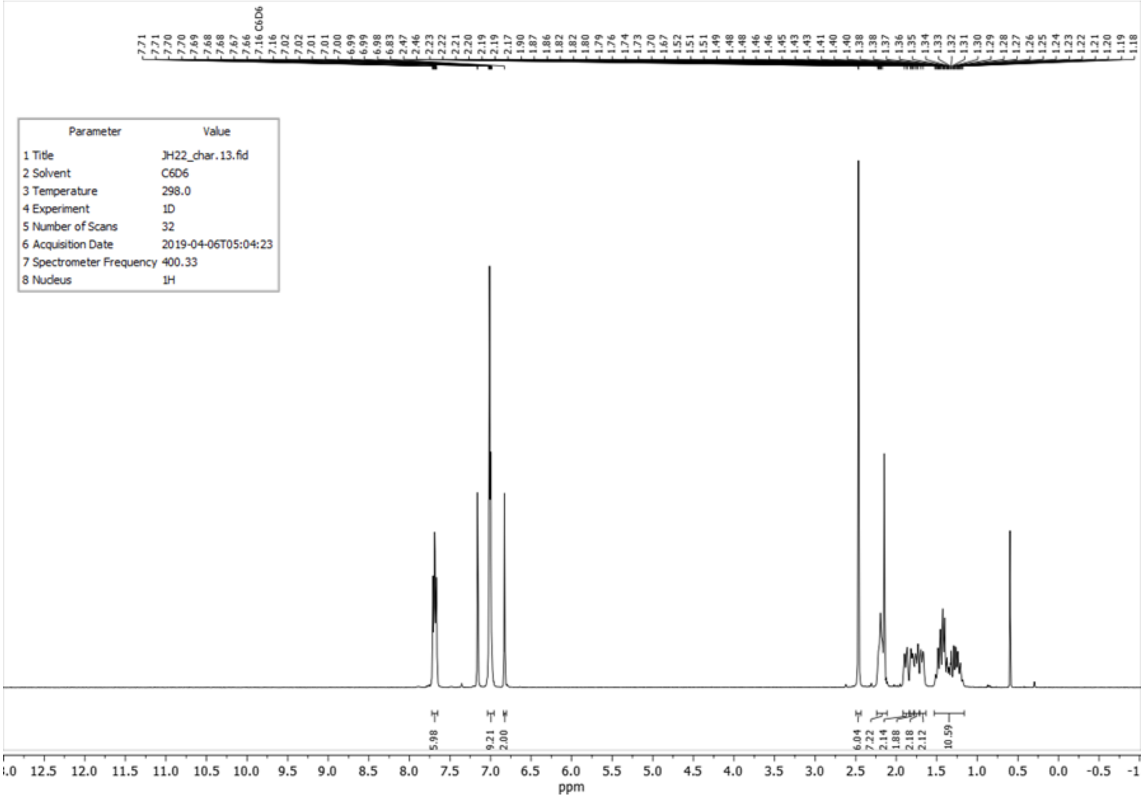

Figure 2.30: ^1H NMR spectrum of $Y_{Mes}PCy_2$ (**5**).

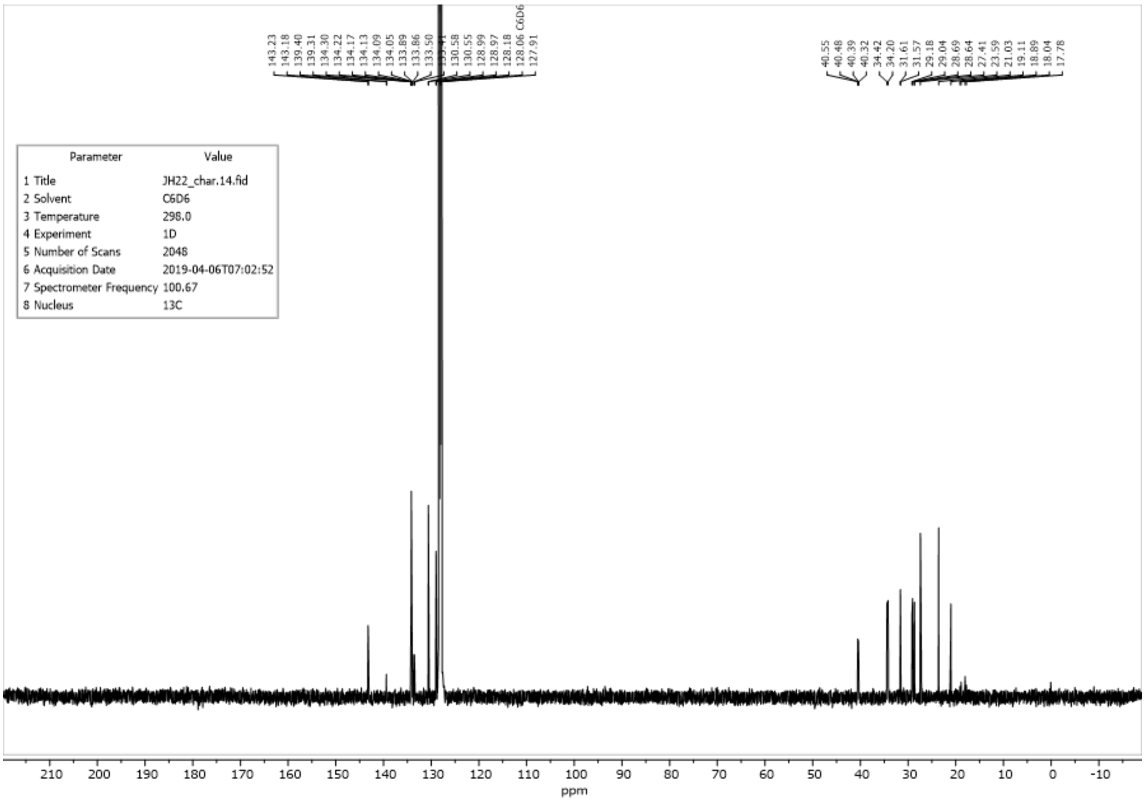

Figure 2.31: $^{13}C\{^1H\}$ NMR spectrum of $Y_{Mes}PCy_2$ (**5**).

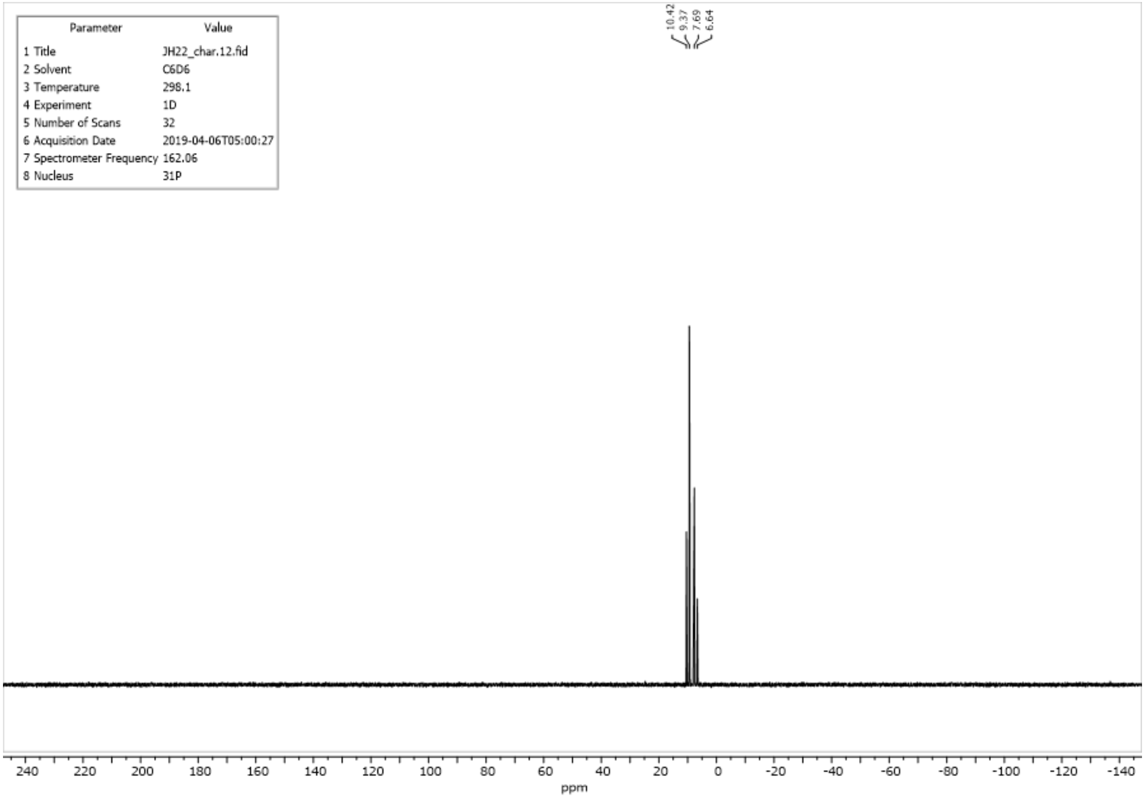

Figure 2.32: $^{31}P\{^1H\}$ NMR spectrum of $Y_{Mes}PCy_2$ (**5**).

$Y_{Mes}PCy_2$•AuCl (5•AuCl)

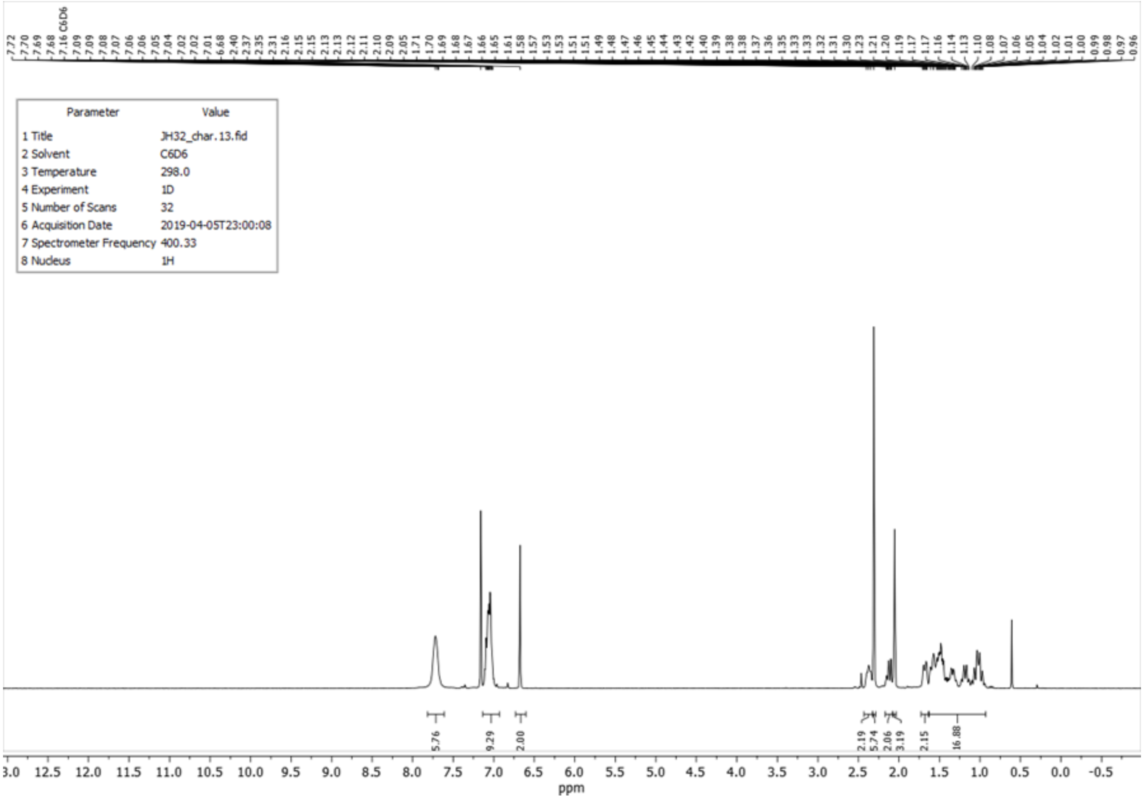

Figure 2.33: ^{1}H NMR spectrum of $Y_{Mes}PCy_2$•AuCl (**5•AuCl**).

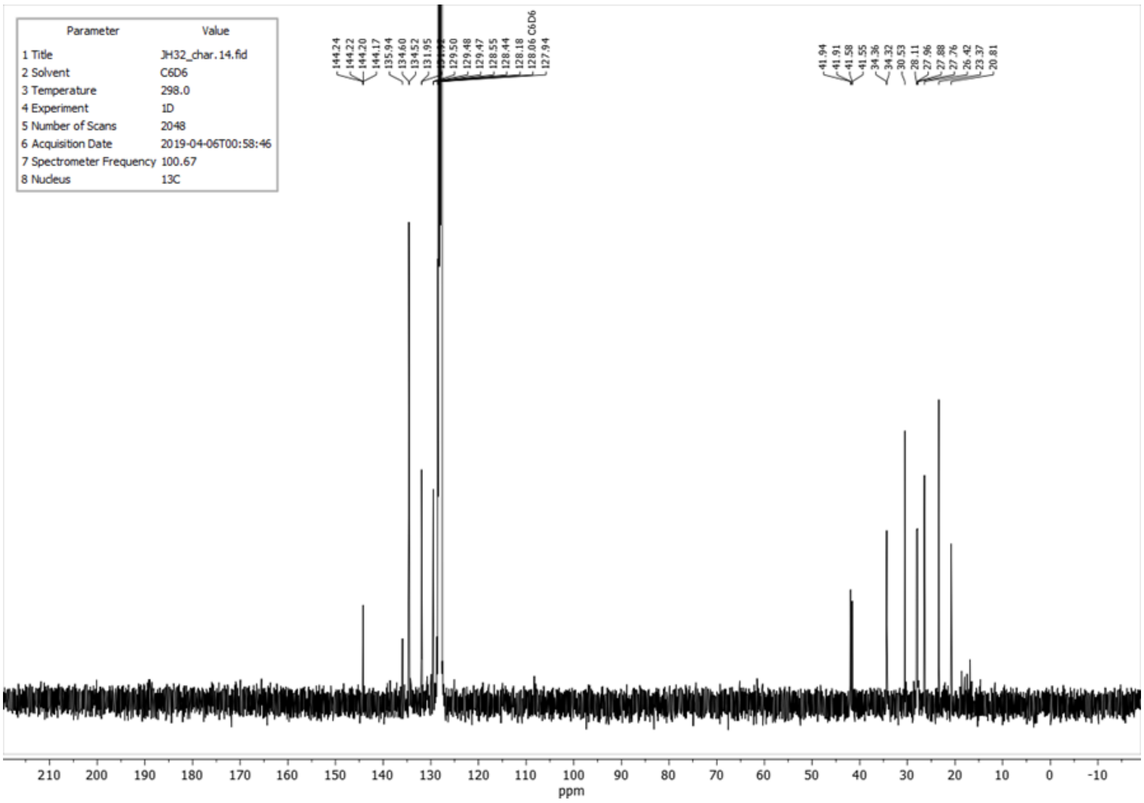

Figure 2.34: ^{13}C{^{1}H} NMR spectrum of $Y_{Mes}PCy_2$•AuCl (**5•AuCl**).

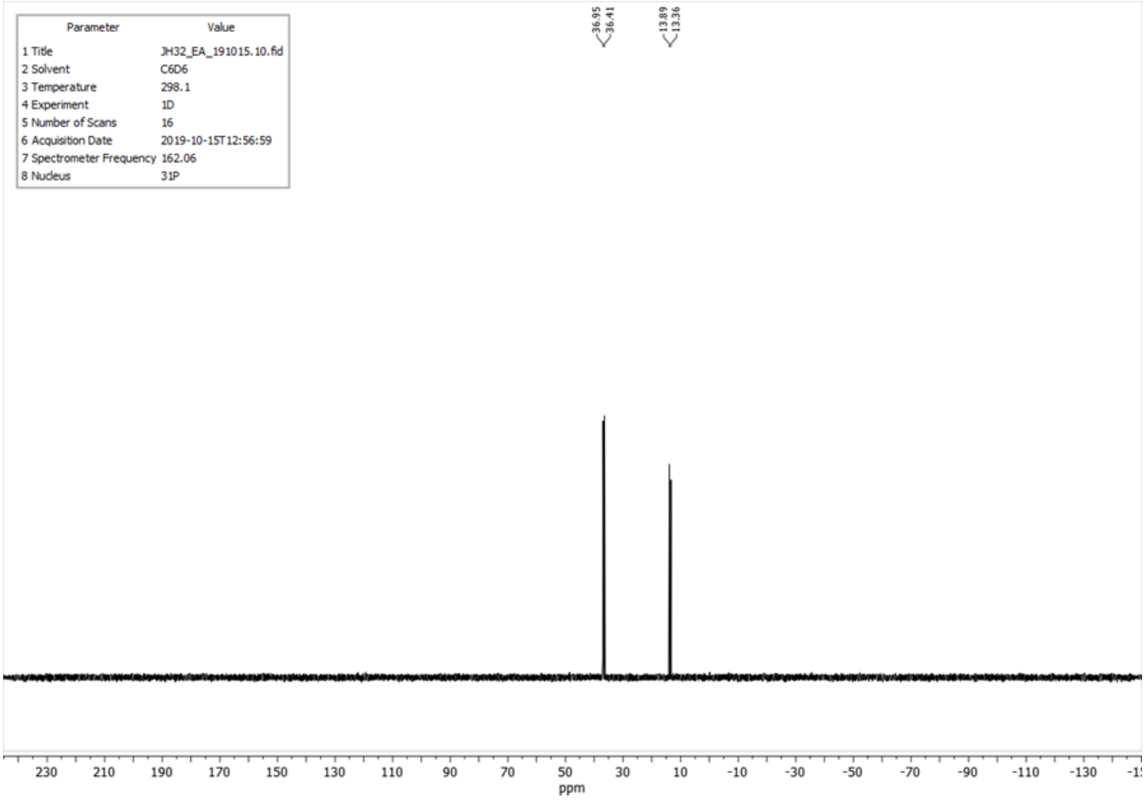

Figure 2.35: $^{31}P\{^{1}H\}$ NMR spectrum of $Y_{Mes}PCy_2$•AuCl (**5•AuCl**).

2.2. NMR spectra of the isolated products of the catalysis

1-Phenyl-*N*-phenylethan-1-imine

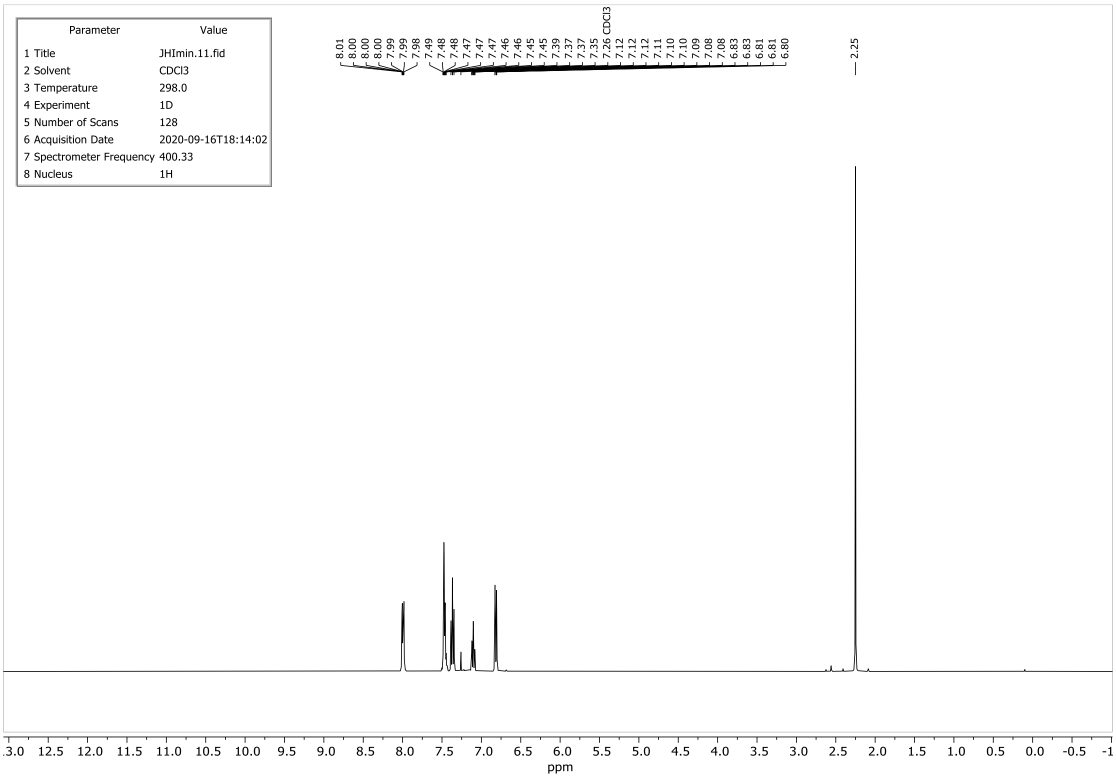

Figure 2.36: ^{1}H NMR spectrum of **1-Phenyl-*N*-phenylethan-1-imine**.

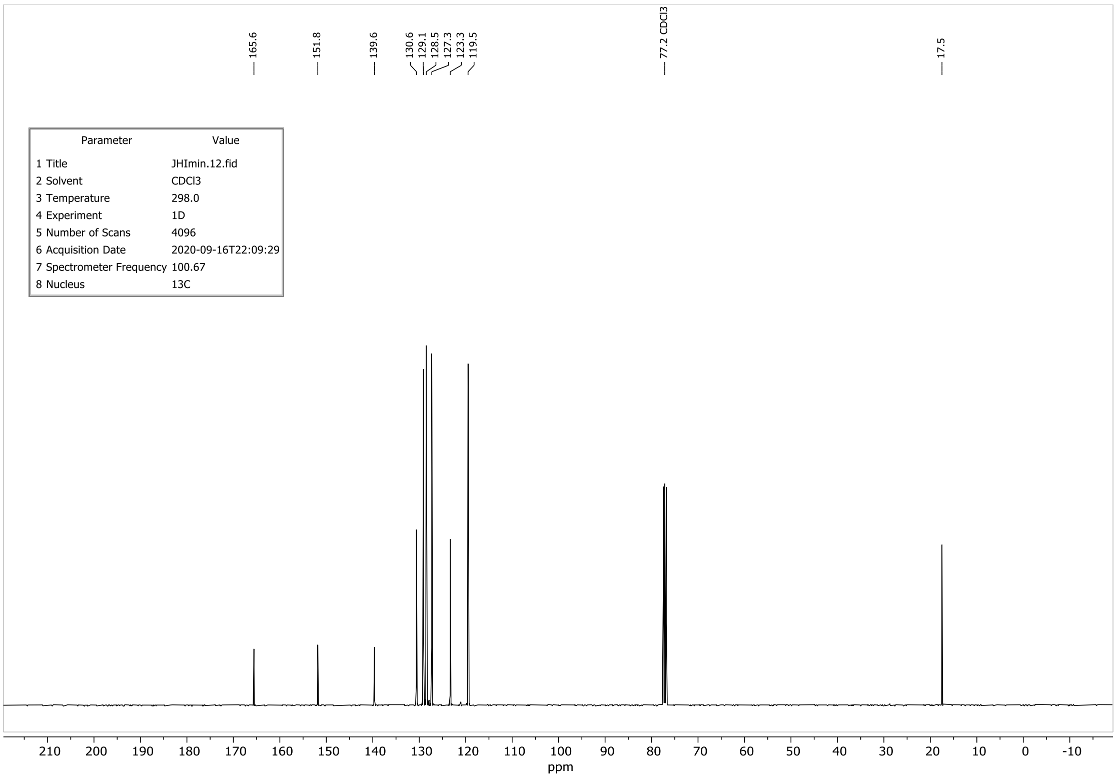

Figure 2.37: $^{13}C\{^{1}H\}$ NMR spectrum of **1-Phenyl-*N*-phenylethan-1-imine**.

1-(4-Methoxyphenyl)-*N*-phenylethan-1-imine

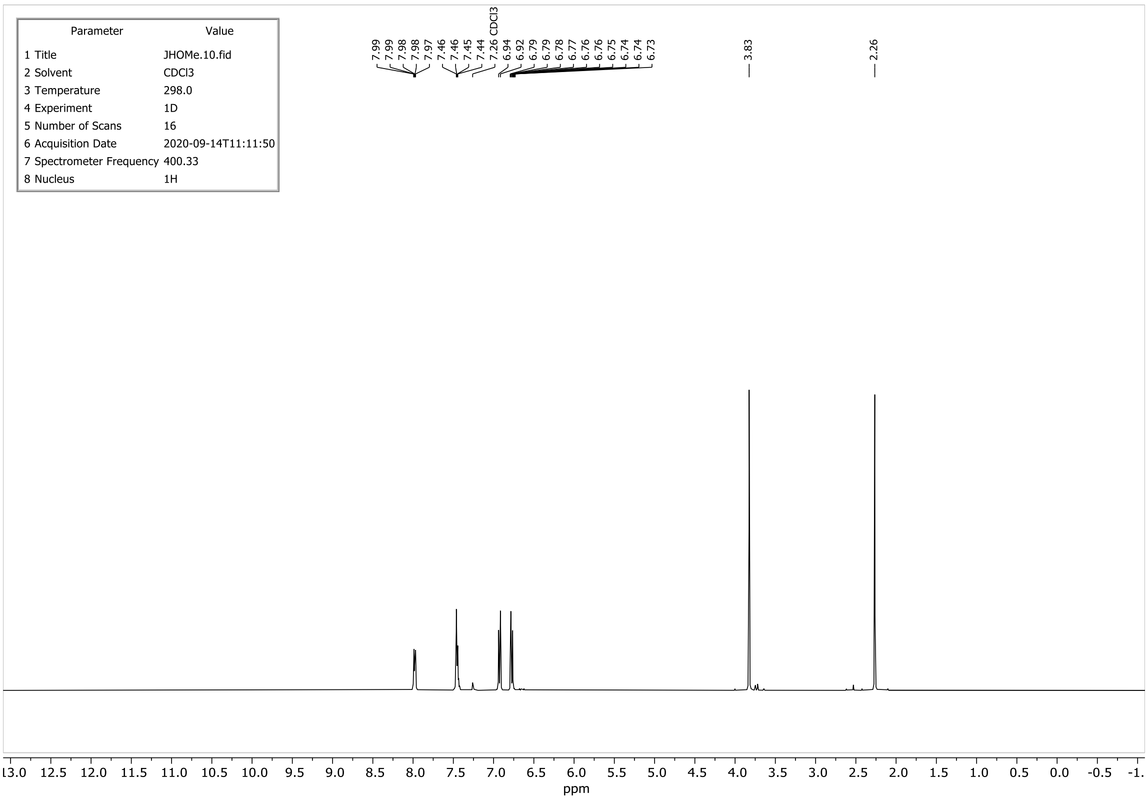

Figure 2.38: ^{1}H NMR spectrum of **1-(4-Methoxyphenyl)-*N*-phenylethan-1-imine**.

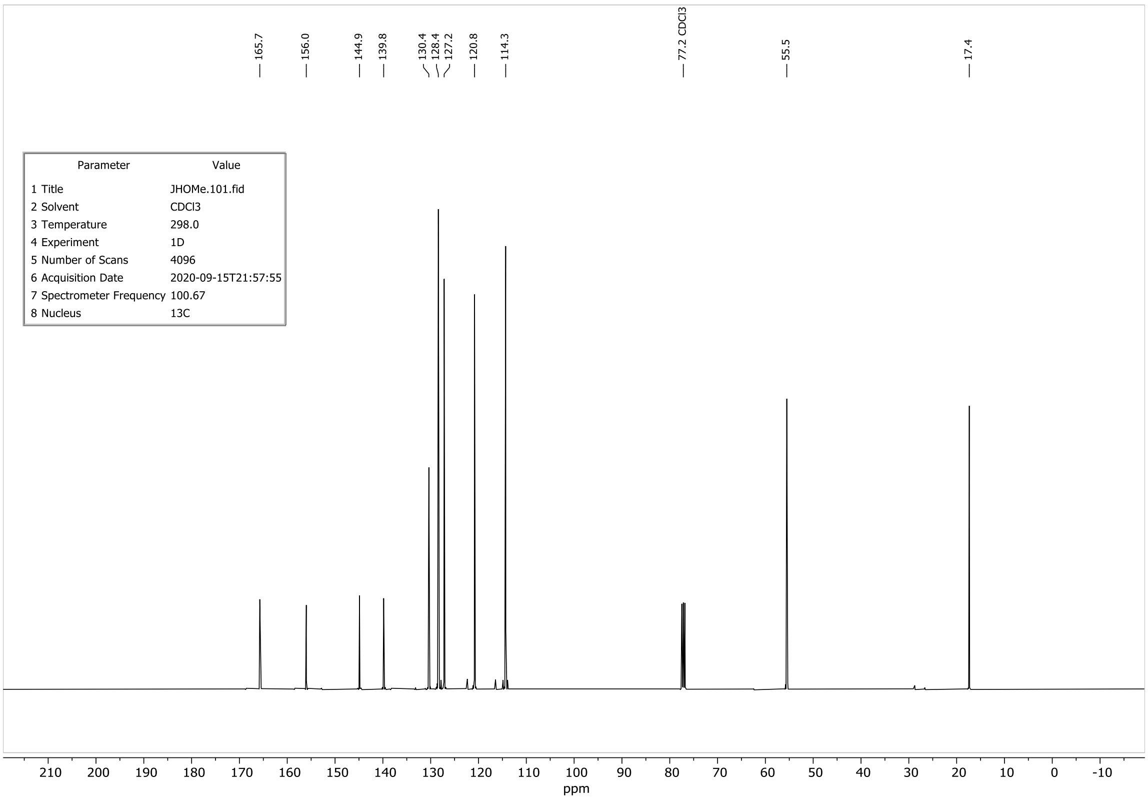

Figure 2.39: $^{13}C\{^{1}H\}$ NMR spectrum of **1-(4-Methoxyphenyl)-*N*-phenylethan-1-imine**.

1-(4-Methoxyphenyl)-*N*-(4-methoxyphenyl)ethan-1-imine

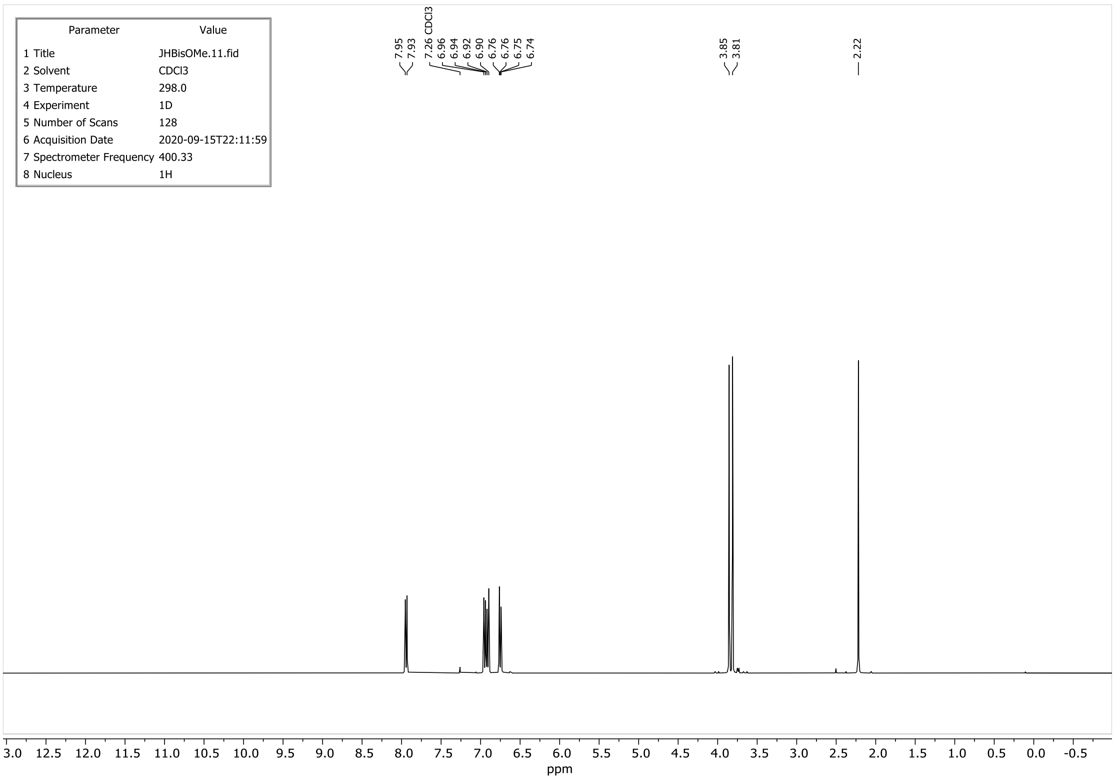

Figure 2.40: ^{1}H NMR spectrum of **1-(4-Methoxyphenyl)-*N*-(4-methoxyphenyl)ethan-1-imine**.

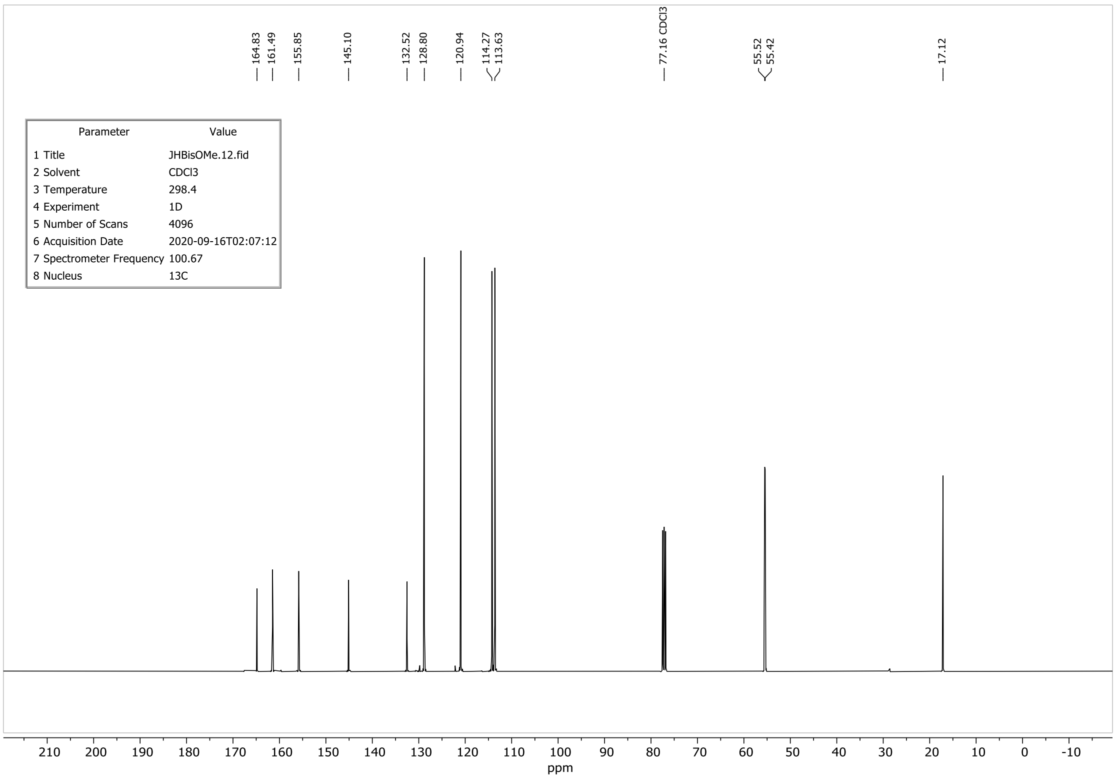

Figure 2.41:^{13}C{^{1}H} NMR spectrum of **1-(4-Methoxyphenyl)-*N*-(4-methoxyphenyl)ethan-1-imine**.

<u>*N*-methyl-*N*-(1-phenylprop-1-en-1-yl)aniline (Mixture of Isomers)</u>

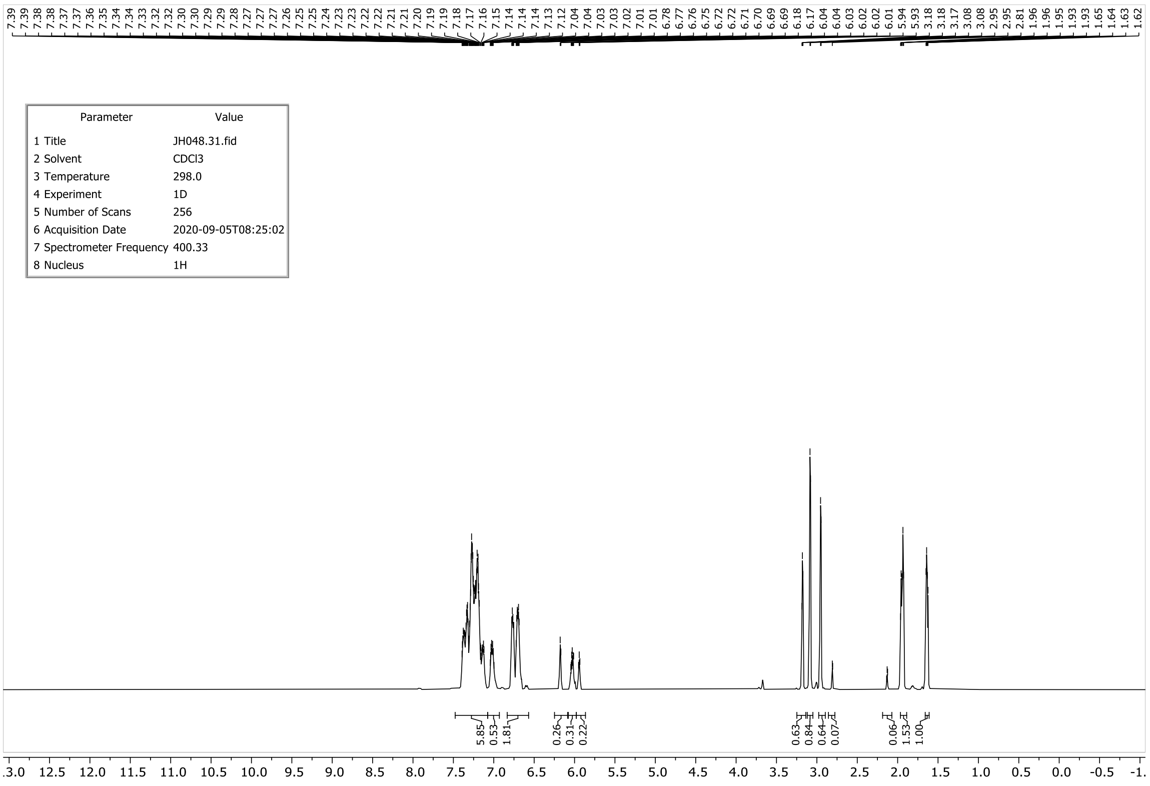

Figure 2.42: ^{1}H NMR spectrum of ***N*-methyl-*N*-(1-phenylprop-1-en-1-yl)aniline** (Mixture of Isomers).

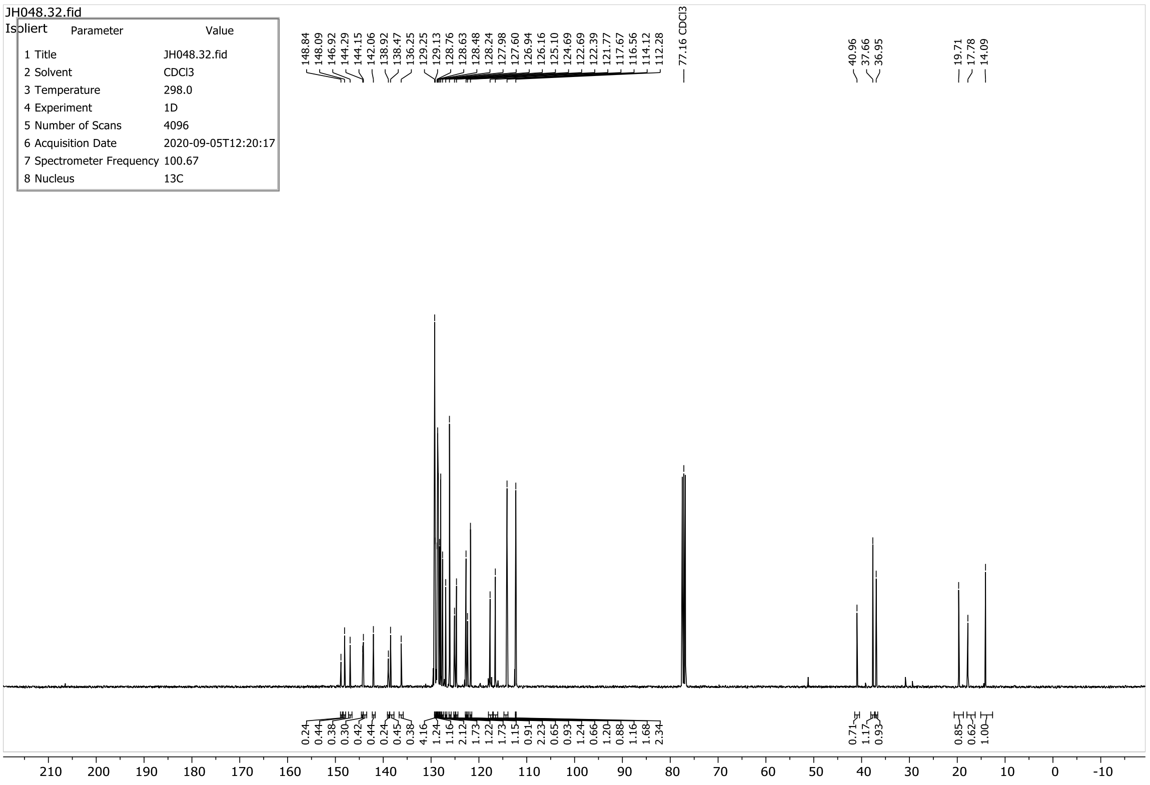

Figure 2.43: ^{13}C{^{1}H} NMR spectrum of ***N*-methyl-*N*-(1-phenylprop-1-en-1-yl)aniline** (Mixture of Isomers).

N,N-bis-(1-phenylvinyl)aniline

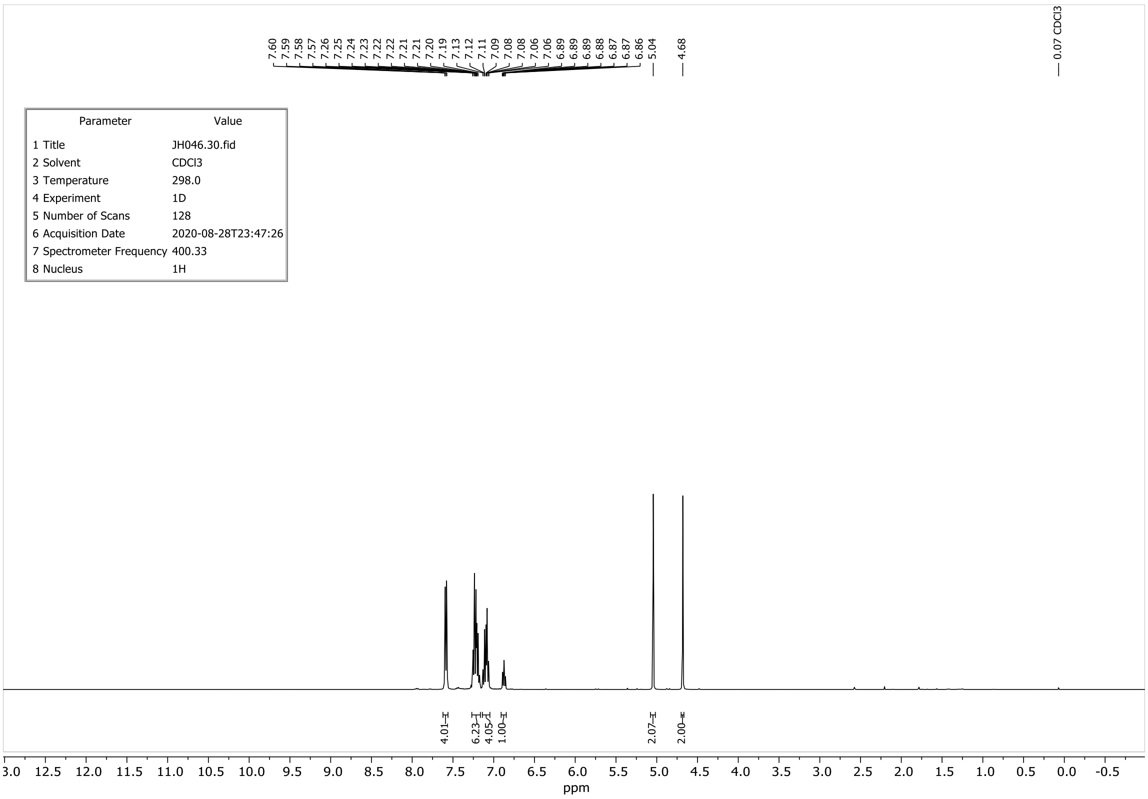

Figure 2.44: *^{1}H NMR spectrum of **N,N*-bis-(1-phenylvinyl)aniline**.

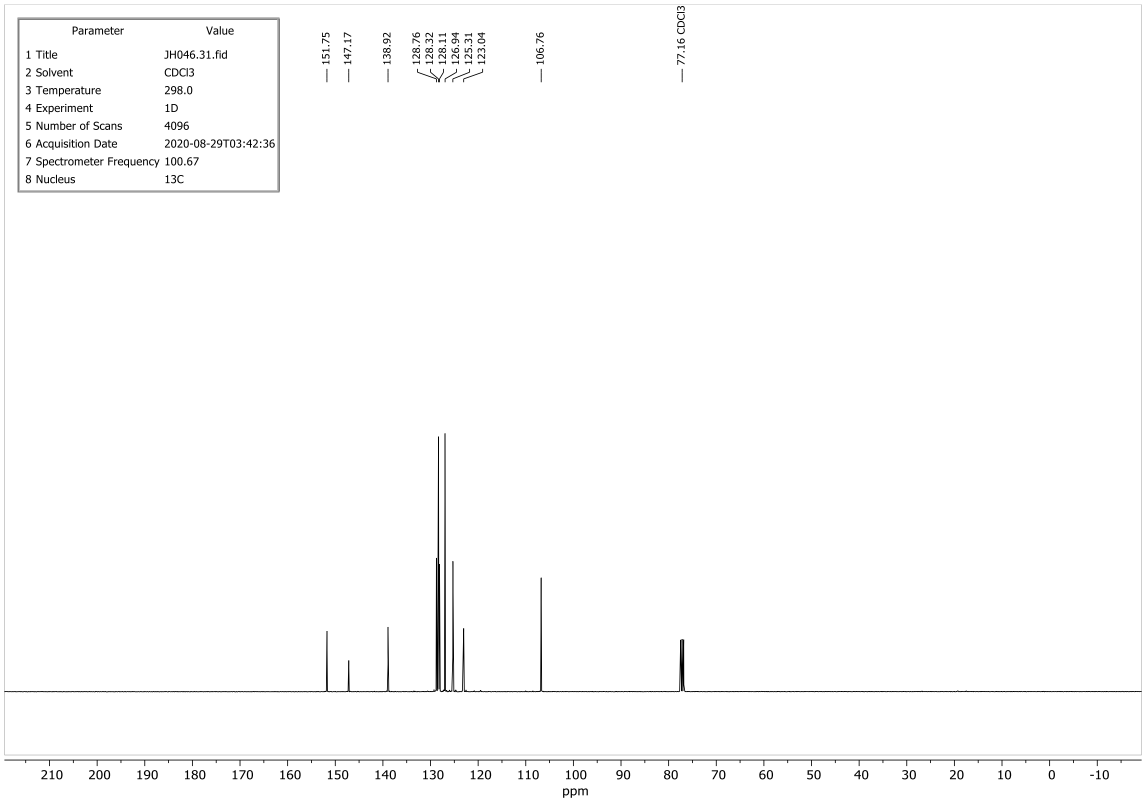

Figure 2.45: ^{13}C{^{1}H} NMR spectrum of ***N,N*-bis-(1-phenylvinyl)aniline.**

1,2-Dimethyl-2,4-diphenyl-1,2-dihydroquinoline

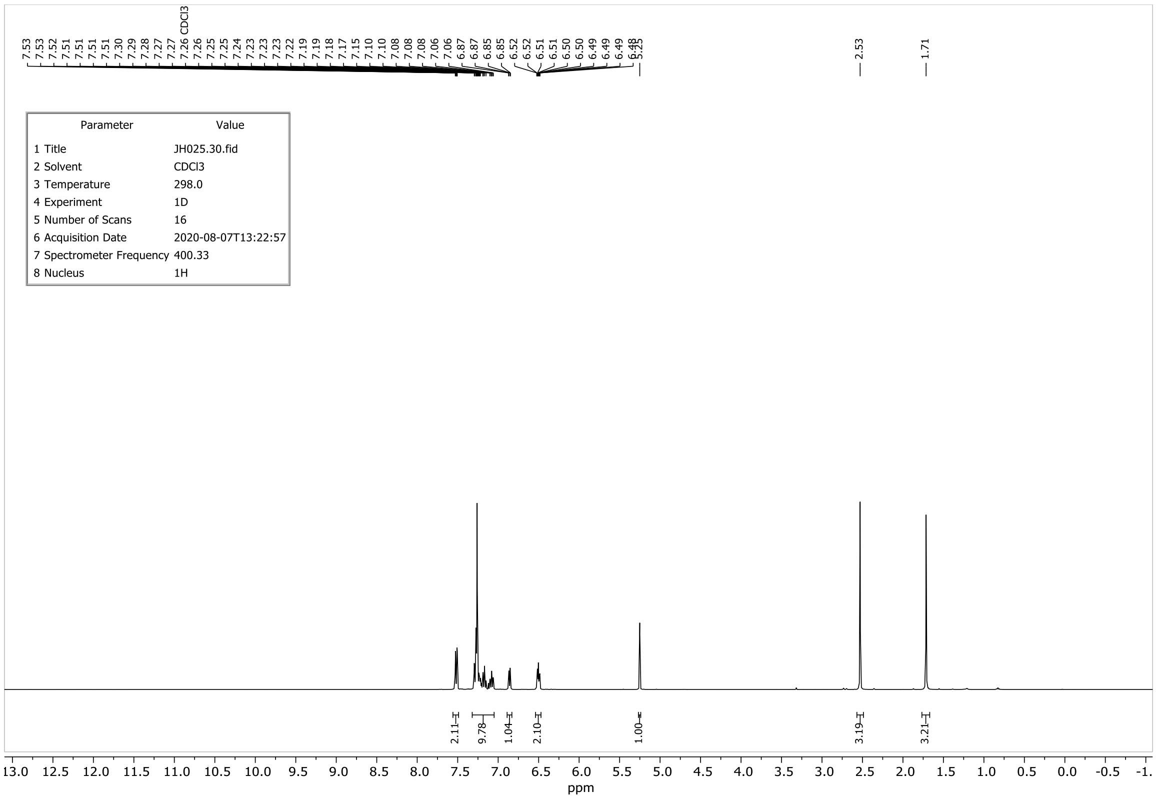

Figure 2.46: ^{1}H NMR spectrum of **1,2-Dimethyl-2,4-diphenyl-1,2-dihydroquinoline**.

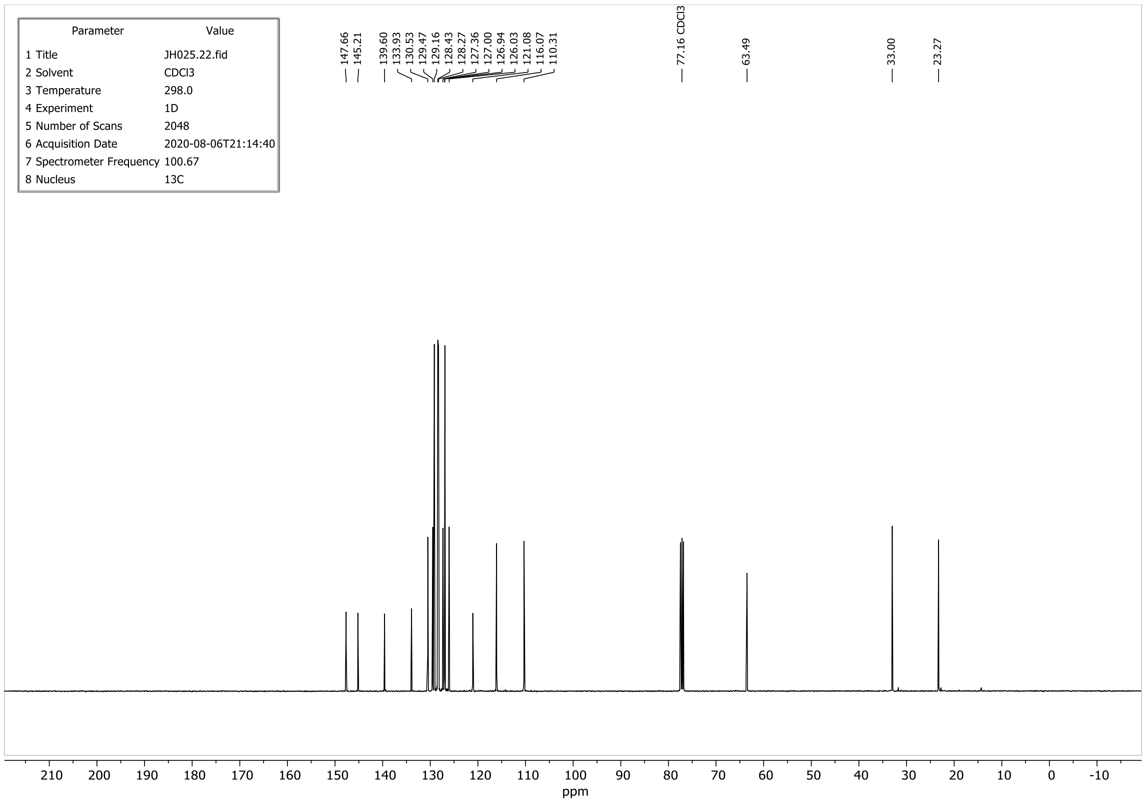

Figure 2.47: ^{13}C{^{1}H} NMR spectrum of **1,2-Dimethyl-2,4-diphenyl-1,2-dihydroquinoline**.

1-Ethyl-2,6-dimethyl-2,4-diphenyl-1,2-dihydroquinoline

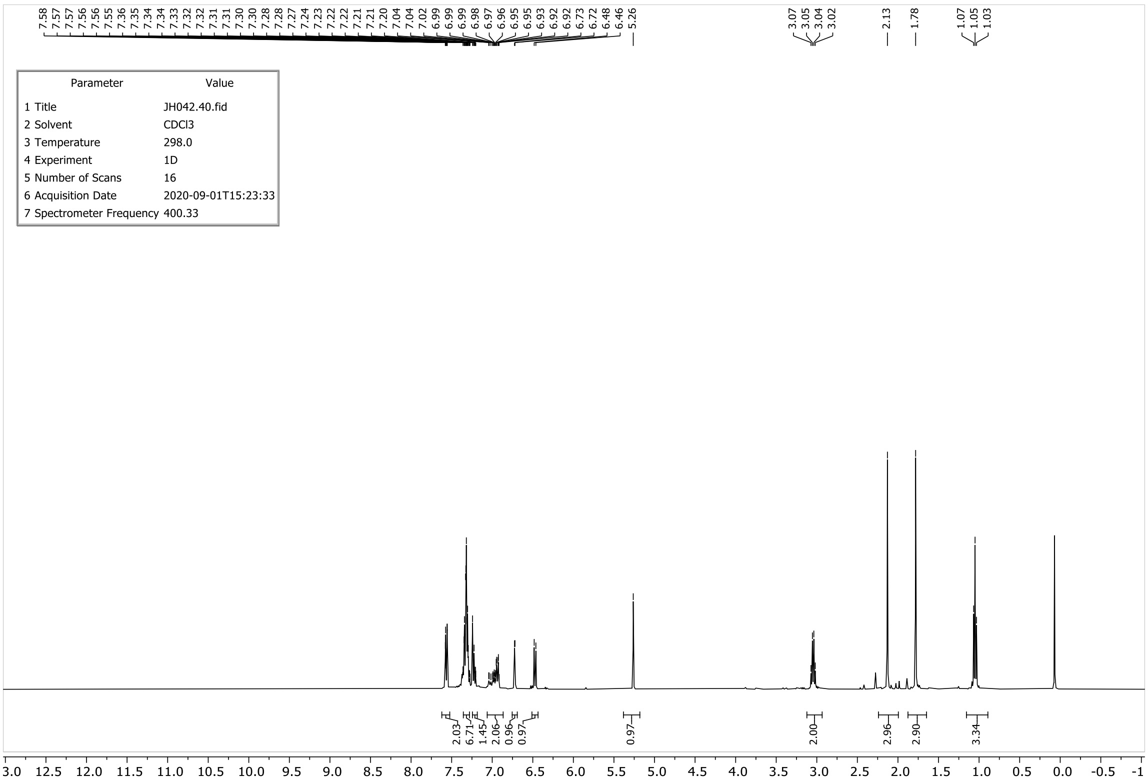

Figure 2.48: ^{1}H NMR spectrum of **1-Ethyl-2,6-dimethyl-2,4-diphenyl-1,2-dihydroquinoline**.

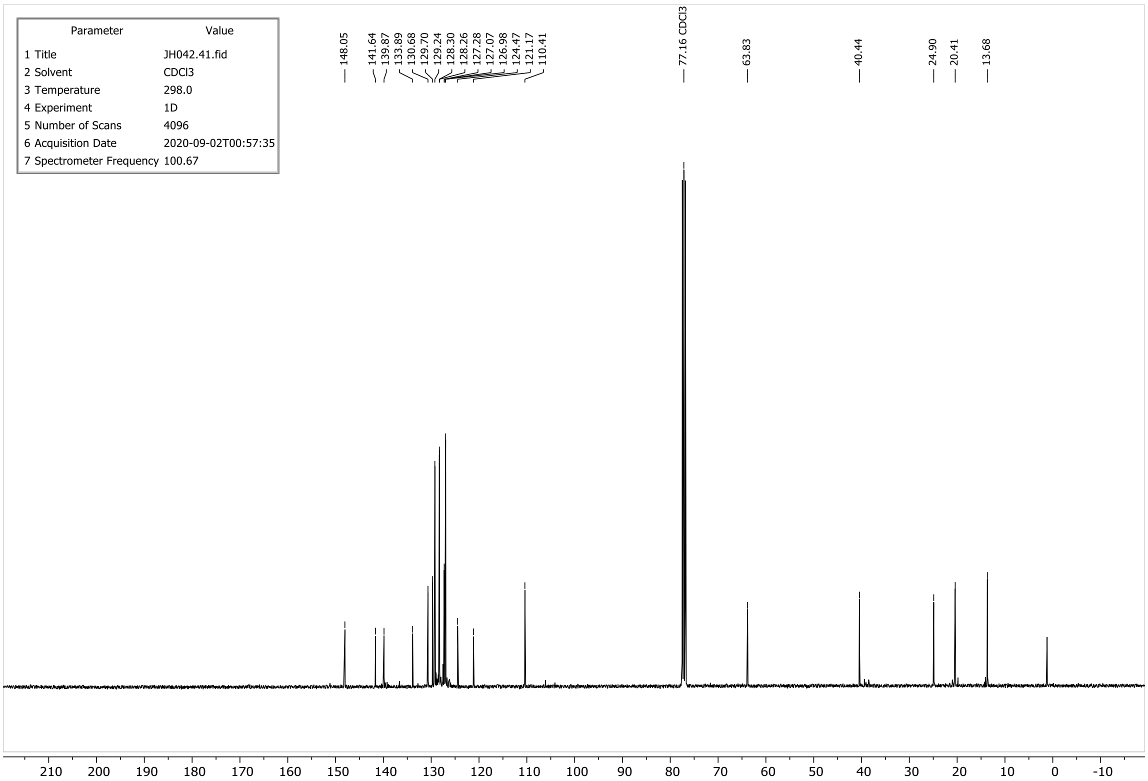

Figure 2.49: ^{13}C{^{1}H} NMR spectrum of **1-Ethyl-2,6-dimethyl-2,4-diphenyl-1,2-dihydroquinoline**.

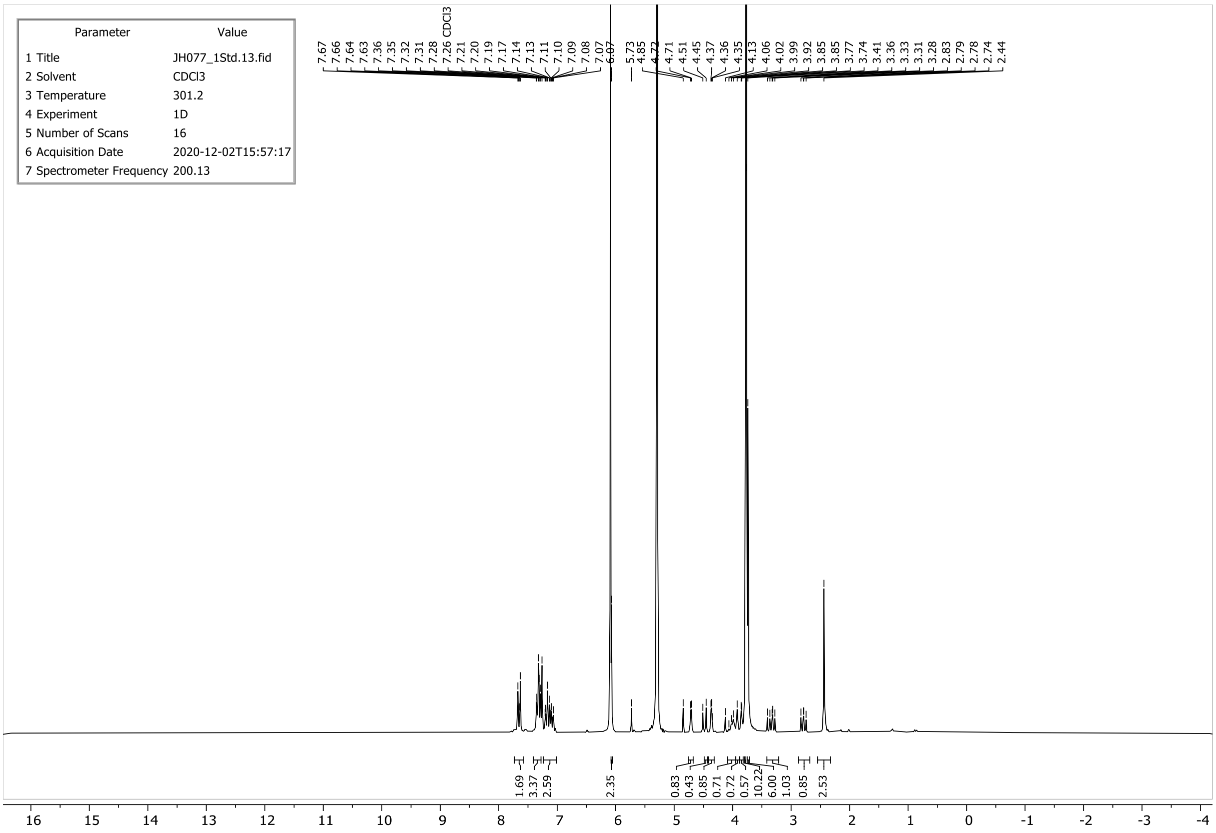

Figure 2.50: ^{1}H NMR spectrum of the reaction mixture of the **1,6-En-Yne-cyclization.**

3. Crystal Structure Determination

3.1. General information

Data collection of all compounds was conducted either with an Rigaku Synergy or Oxford SuperNova .The structures were solved using direct methods, refined with the Shelx software package[24–26] and expanded using Fourier techniques. The crystals of all compounds were mounted in an inert oil (perfluoropolyalkylether). Crystal structure determinations were affected at 100 K. Crystallographic data (including structure factors) have been deposited with the Cambridge Crystallographic Data Centre as supplementary publication no. CCDC-1983138-1983147. Copies of the data can be obtained free of charge on application to Cambridge Crystallographic Data Centre, 12 Union Road, Cambridge CB2 1EZ, UK; [fax: (+44) 1223-336-033; email: deposit@ccdc.cam.ac.uk].

Details on the structure solutions

The structure of **$Y_{Ph}PCy_2$ (1)** contained a toluene solvent molecule. The hydrogen atoms of the methyl group of the toluene solvent molecule is disordered over two positions with an occupancy of 0.57 and 0.43.

The structure of **$Y_{pOMe}PCy_2$ (2)** contained two cyclohexyl-groups disordered over two positions with an occupancy of 0.55 and 0.45. This was modeled by using the SIMU restraint.

The structure of **$Y_{Mes}PCy_2$ (5)** contained either a DCM or acetonitrile solvent molecule with a ratio of 0.21 to 0.79.

The structure of **$Y_{Ph}PCy_2$•AuCl (1•AuCl)** contained a THF solvent molecule disordered over two positions with an occupancy of 0.60 and 0.40. This was modelled by using the SAME RIGU and SIMU restraints.

The structure of **$Y_{pOMe}PCy_2$•AuCl (2•AuCl)** contained a highly disordered solvent molecule that was treated by using the PLATON/SQUEEZE routine[24–26].

Table 3.1: Data collection and refinement details for the compounds **$Y_{Ph}PCy_2$ (1)**, **$Y_{pOMe}PCy_2$ (2)** and **$Y_{pCF3}PCy_2$ (3)**.

Compound	**$Y_{Ph}PCy_2$ (1)**	**$Y_{pOMe}PCy_2$ (2)**	**$Y_{pCF3}PCy_2$ (3)**
CCDC No.	1983146	1983142	1983138
Formula	$C_{44}H_{50}P_2$	$C_{38}H_{44}OP_2$	$C_{38}H_{41}F_3P_2$
Formula weight [g·mol^{-1}]	640.78	578.67	616.65
Temperature [K]	100(2)	100(2)	100(2)
Wave length [Å]	1.54184	1.54184	1.54184
Crystal system	Triclinic	Orthorhombic	Monoclinic
Space group	P-1	$Pca2_1$	$P2_1/c$
a [Å]	9.3576(5)	11.39560(15)	9.60831(8)
b [Å]	13.3096(6)	15.6766(2)	33.8371(3)
c [Å]	14.9346(8)	18.1208(3)	10.09764(10)
α [°]	93.442(4)	90	90
β [°]	93.343(4)	90	99.1105(8)
γ [°]	107.914(4)	90	90
Volume [Å^3]	1760.89(16)	3237.16(8)	3241.50(5)
Z	2	4	4
Calc. density [Mg·m^{-3}]	1.209	1.187	1.264
μ [mm^{-1}]	1.334	1.422	1.567
F(000)	688	1240	1304
Crystal dimensions [mm]	0.184 x 0.176 x 0.153	0.268 x 0.079 x 0.036	0.102 x 0.054 x 0.027
Theta range [°]	4.406 to 74.992	3.728 to 74.987	2.612 to 74.979
Index ranges	$-11 \le h \le 10$<br>$-14 \le k \le 16$<br>$-18 \le l \le 18$	$-14 \le h \le 14$<br>$-19 \le k \le 19$<br>$-22 \le l \le 20$	$-11 \le h \le 12$<br>$-41 \le k \le 41$<br>$-9 \le l \le 12$
Reflections collected	14570	27709	26304
Independent reflections	7169 [R_{int} = 0.0358]	6357 [R(int) = 0.0364]	6600 [R_{int} = 0.0297]
Data/Restraints/Parameter	7169 / 0 / 417	6357 / 393 / 481	6600 / 0 / 388
Goodness-of-fit on F^2	1.023	1.013	1.040
Final R indices [I>2sigma(I)]	R1 = 0.0389, wR2 = 0.0917	R1 = 0.0365, wR2 = 0.0960	R1 = 0.0364, wR2 = 0.0953
R indices (all data)	R1 = 0.0503, wR2 = 0.0998	R1 = 0.0382, wR2 = 0.0979	R1 = 0.0404, wR2 = 0.0982
Largest diff. peak and hole	0.331 and -0.381	0.623 and -0.504	0.412 and -0.467

Table 3.2: Data collection and structure refinement details for the compounds **$Y_{oTol}PCy_2$ (4)** and **$Y_{Mes}PCy_2$ (5).**

Compound	**$Y_{oTol}PCy_2$ (4)**	**$Y_{Mes}PCy_2$ (5)**
CCDC No.	1983144	1983140
Formula	$C_{38}H_{44}P_2$	$C_{40}H_{48}P_2$
Formula weight [g·mol^{-1}]	526.67	640.81
Temperature [K]	100(2)	100(2)
Wave length [Å]	1.54184	1.54184
Crystal system	Monoclinic	Triclinic
Space group	$P2_1/n$	P-1
a [Å]	10.29027(19)	10.9245(2)
b [Å]	10.5467(2)	12.9655(3)
c [Å]	29.0177(6)	14.1731(2)
α [°]	90	63.574(2)
β [°]	96.2428(17)	81.1475(15)
γ [°]	90	75.1466(18)
Volume [Å^3]	3130.59(11)	1735.83(7)
Z	4	2
Calc. density [Mg·m^{-3}]	1.194	1.226
μ [mm^{-1}]	1.432	1.643
F(000)	1208	688
Crystal dimensions [mm]	0.186 x 0.131 x 0.110	0.102 x 0.093 x 0.057
Theta range [°]	4.426 to 74.994	3.486 to 74.980
Index ranges	$-12 \le h \le 12$	$-13 \le h \le 13$
	$-12 \le k \le 13$	$-16 \le k \le 16$
	$-35 \le l \le 36$	$-17 \le l \le 17$
Reflections collected	25763	25120
Independent reflections	6423 [R_{int} = 0.0463]	7085 [R_{int} = 0.0277]
Data/Restraints/Parameter	6423 / 0 / 363	7085 / 0 / 438
Goodness-of-fit on F^2	1.033	1.043
Final R indices	R1 = 0.0397,	R1 = 0.0334,
[I>2sigma(I)]	wR2 = 0.0993	wR2 = 0.0870
R indices (all data)	R1 = 0.0532,	R1 = 0.0350,
	wR2 = 0.1076	wR2 = 0.0880
Largest diff. peak and hole	0.418 and -0.328	0.403 and -0.355

Table 3.3: Data collection and structure refinement details for the compounds **$Y_{Ph}PCy_2$•AuCl (1•AuCl)**, **$Y_{pOMe}PCy_2$•AuCl (2•AuCl)** and **$Y_{pCF3}PCy_2$•AuCl (3•AuCl)**.

Compound	**$Y_{Ph}PCy_2$•AuCl (1•AuCl)**	**$Y_{pOMe}PCy_2$•AuCl (2•AuCl)**	**$Y_{pCF3}PCy_2$•AuCl (3•AuCl)**
CCDC No.	1983147	1983143	1983138
Formula	$C_{41}H_{50}AuClOP_2$	$C_{38}H_{44}AuClOP_2$	$C_{38}H_{41}AuClF_3P_2$
Formula weight [g·mol^{-1}]	853.16	811.09	849.06
Temperature [K]	100(2)	100(2)	100(2)
Wave length [Å]	1.54184	1.54184	1.54184
Crystal system	Triclinic	Monoclinic	Triclinic
Space group	P-1	C2/c	P-1
a [Å]	9.3045(4)	41.898(3)	11.01072(16)
b [Å]	10.5020(4)	15.2834(4)	17.1399(2)
c [Å]	18.6397(6)	11.6875(9)	18.8182(2)
α [°]	97.739(3)	90	80.1354(11)
β [°]	91.819(3)	93.097(8)	77.3539(12)
γ [°]	94.738(3)	90	89.9789(12)
Volume [Å^3]	1796.94(12)	7473.1(8)	3411.49(8)
Z	2	8	4
Calc. density [Mg·m^{-3}]	1.577	1.442	1.653
μ [mm^{-1}]	9.452	9.062	10.064
F(000)	860	3248	1688
Crystal dimensions [mm]	0.181 x 0.141 x 0.064	0.101 x 0.097 x 0.037	0.095 x 0.071 x 0.011
Theta range [°]	4.265 to 76.249	3.078 to 74.964	2.444 to 74.989
Index ranges	$-11 \le h \le 11$<br>$-13 \le k \le 13$<br>$-16 \le l \le 23$	$-52 \le h \le 51$<br>$-13 \le k \le 19$<br>$-14 \le l \le 14$	$-13 \le h \le 13$<br>$-21 \le k \le 21$<br>$-21 \le l \le 23$
Reflections collected	12891	31258	55562
Independent reflections	7546 [R_{int} = 0.0416]	7546 [R_{int} = 0.0428]	13943 [R_{int} = 0.0318]
Data/Restraints/Parameter	7276 / 0 / 461	7546 / 0 / 391	13943 / 0 / 811
Goodness-of-fit on F^2	1.081	1.061	1.093
Final R indices [I>2sigma(I)]	R1 = 0.0466, wR2 = 0.1291	R1 = 0.0285, wR2 = 0.0790	R1 = 0.0228, wR2 = 0.0588
R indices (all data)	R1 = 0.0483, wR2 = 0.1332	R1 = 0.0295, wR2 = 0.0798	R1 = 0.0244, wR2 = 0.0594
Largest diff. peak and hole	2.728 and -2.476	1.763 and -1.610	1.034 and -1.261

Table 3.4: Data collection and structure refinement details for the compounds **$Y_{oTol}PCy_2$•AuCl (4•AuCl)** and **$Y_{Mes}PCy_2$•AuCl (5•AuCl).**

Compound	**$Y_{oTol}PCy_2$•AuCl (4•AuCl)**	**$Y_{Mes}PCy_2$•AuCl (5•AuCl)**
CCDC No.	1983145	1983141
Formula	$C_{46}H_{52}AuClP_2$	$C_{40}H_{48}AuClP_2$
Formula weight [g·mol^{-1}]	887.22	823.14
Temperature [K]	100(2)	100.00(10)
Wave length [Å]	1.54184	1.54184
Crystal system	Monoclinic	Monoclinic
Space group	$P2_1/c$	$P2_1/n$
a [Å]	11.24594(15)	12.0432(2)
b [Å]	18.3601(2)	16.7764(2)
c [Å]	18.9125(3)	17.8978(2)
α [°]	90	90
β [°]	95.9476(12)	106.6500(10)
γ [°]	90	90
Volume [Å^3]	3883.96(9)	3464.49(8)
Z	4	4
Calc. density [Mg·m^{-3}]	1.517	1.578
μ [mm^{-1}]	8.752	9.759
F(000)	1792	1656
Crystal dimensions [mm]	0.206 x 0.111 x 0.033	0.108 x 0.095 x 0.027
Theta range [°]	3.364 to 74.962	3.686 to 77.387
Index ranges	$-13 \le h \le 11$<br>$-22 \le k \le 22$<br>$-23 \le l \le 21$	$-15 \le h \le 15$<br>$-13 \le k \le 21$<br>$-20 \le l \le 22$
Reflections collected	18357	26681
Independent reflections	7920 [R_{int} = 0.0223]	7185 [R_{int} = 0.0228]
Data/Restraints/Parameter	7920 / 0 / 444	7185 / 0 / 400
Goodness-of-fit on F^2	1.072	1.060
Final R indices [I>2sigma(I)]	R1 = 0.0247, wR2 = 0.0608	R1 = 0.0189, wR2 = 0.0490
R indices (all data)	R1 = 0.0261, wR2 = 0.0616	R1 = 0.0197, wR2 = 0.0494
Largest diff. peak and hole	1.081 and -0.987	0.598 and -0.675

3.2. Crystal strutures of the YPhos ligands

3.2.1. Crystal structure determination of **Y$_{Ph}$PCy$_2$ (1)**

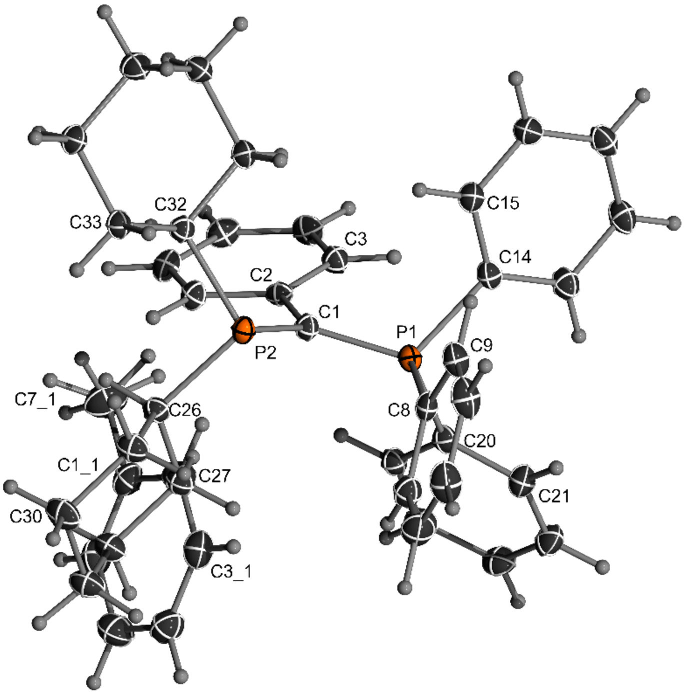

Figure 3.1: ORTEP of **Y$_{Ph}$PCy$_2$ (1)**. Ellipsoids are drawn at the 50% probability level.
Table 3.5: Atomic coordinates (x 10^4) and equivalent isotropic displacement parameters (Å^{2}x 10^3) for **Y$_{Ph}$PCy$_2$ (1).** U(eq) is defined as one third of the trace of the orthogonalized Uij tensor.

	x	y	z	U(eq)
**P(1)**	4044(1)	7781(1)	2460(1)	15(1)
**P(2)**	7017(1)	8475(1)	1685(1)	16(1)
**C(1)**	5914(2)	7852(1)	2569(1)	16(1)
**C(2)**	6516(2)	7502(1)	3383(1)	16(1)
**C(3)**	5829(2)	7439(1)	4206(1)	20(1)
**C(4)**	6397(2)	7085(1)	4960(1)	23(1)
**C(5)**	7693(2)	6782(1)	4938(1)	22(1)
**C(6)**	8388(2)	6832(1)	4142(1)	21(1)
**C(7)**	7824(2)	7185(1)	3384(1)	18(1)
**C(8)**	3519(2)	8130(1)	1365(1)	18(1)
**C(9)**	3526(2)	9159(1)	1222(1)	23(1)
**C(10)**	3173(2)	9410(2)	364(1)	29(1)
**C(11)**	2795(2)	8635(2)	-348(1)	31(1)
**C(12)**	2768(2)	7608(2)	-210(1)	28(1)
**C(13)**	3135(2)	7355(2)	644(1)	23(1)
**C(14)**	3467(2)	8634(1)	3285(1)	18(1)
**C(15)**	4442(2)	9655(1)	3502(1)	21(1)
**C(16)**	4084(2)	10343(1)	4124(1)	24(1)
**C(17)**	2755(2)	10010(2)	4542(1)	23(1)
**C(18)**	1788(2)	8995(2)	4341(1)	25(1)
**C(19)**	2133(2)	8305(1)	3714(1)	23(1)

**C(20)**	2731(2)	6459(1)	2542(1)	18(1)
**C(21)**	1180(2)	6243(1)	2318(1)	23(1)
**C(22)**	185(2)	5246(2)	2403(1)	26(1)
**C(23)**	717(2)	4442(1)	2688(1)	26(1)
**C(24)**	2249(2)	4637(1)	2879(1)	23(1)
**C(25)**	3254(2)	5641(1)	2809(1)	19(1)
**C(26)**	7713(2)	7454(1)	1072(1)	17(1)
**C(27)**	6662(2)	6320(1)	1091(1)	21(1)
**C(28)**	7216(2)	5523(1)	539(1)	23(1)
**C(29)**	7394(2)	5803(2)	-432(1)	26(1)
**C(30)**	8428(2)	6934(2)	-470(1)	28(1)
**C(31)**	7881(2)	7726(1)	90(1)	22(1)
**C(32)**	8810(2)	9355(1)	2299(1)	17(1)
**C(33)**	9949(2)	9885(1)	1636(1)	19(1)
**C(34)**	11368(2)	10674(1)	2123(1)	22(1)
**C(35)**	10998(2)	11517(1)	2722(1)	24(1)
**C(36)**	9884(2)	11000(1)	3392(1)	21(1)
**C(37)**	8462(2)	10199(1)	2918(1)	19(1)
**C1_1**	7044(2)	3509(2)	3737(1)	25(1)
**C2_1**	6341(2)	4158(2)	3309(1)	27(1)
**C3_1**	5498(2)	3816(2)	2493(1)	27(1)
**C4_1**	5325(2)	2818(2)	2094(1)	30(1)
**C5_1**	6014(2)	2167(2)	2509(1)	33(1)
**C6_1**	6867(2)	2516(2)	3324(1)	29(1)
**C7_1**	7971(3)	3891(2)	4620(1)	40(1)

Table 3.6: Anisotropic displacement parameters (Å^2 x 10^3) for **Y$_{Ph}$PCy$_2$ (1).** The anisotropic displacement factor exponent takes the form: $-2\pi^2$ [$h^2a^{\cdot 2}U^{11}$ + ... + 2 h k a·b·U^{12}].

	U^{11}	U^{22}	U^{22}	U^{23}	U^{13}	U^{12}
**P(1)**	14(1)	16(1)	15(1)	2(1)	3(1)	5(1)
**P(2)**	15(1)	17(1)	15(1)	3(1)	3(1)	5(1)
**C(1)**	15(1)	18(1)	16(1)	2(1)	3(1)	5(1)
**C(2)**	16(1)	14(1)	16(1)	1(1)	1(1)	3(1)
**C(3)**	19(1)	22(1)	18(1)	1(1)	3(1)	7(1)
**C(4)**	25(1)	26(1)	16(1)	3(1)	4(1)	6(1)
**C(5)**	24(1)	24(1)	18(1)	5(1)	-2(1)	6(1)
**C(6)**	17(1)	22(1)	25(1)	6(1)	2(1)	6(1)
**C(7)**	17(1)	20(1)	18(1)	4(1)	4(1)	4(1)
**C(8)**	13(1)	23(1)	19(1)	4(1)	3(1)	7(1)
**C(9)**	24(1)	26(1)	23(1)	6(1)	6(1)	13(1)
**C(10)**	32(1)	36(1)	28(1)	13(1)	9(1)	21(1)
**C(11)**	33(1)	49(1)	19(1)	12(1)	4(1)	22(1)
**C(12)**	27(1)	40(1)	18(1)	1(1)	0(1)	11(1)
**C(13)**	21(1)	27(1)	21(1)	3(1)	3(1)	7(1)
**C(14)**	18(1)	21(1)	18(1)	3(1)	2(1)	9(1)
**C(15)**	18(1)	24(1)	23(1)	0(1)	3(1)	6(1)
**C(16)**	23(1)	24(1)	24(1)	-4(1)	0(1)	8(1)
**C(17)**	27(1)	29(1)	17(1)	0(1)	3(1)	14(1)

**C(18)**	25(1)	29(1)	24(1)	4(1)	10(1)	10(1)
**C(19)**	23(1)	22(1)	24(1)	3(1)	8(1)	7(1)
**C(20)**	18(1)	20(1)	16(1)	2(1)	5(1)	6(1)
**C(21)**	18(1)	26(1)	24(1)	4(1)	3(1)	7(1)
**C(22)**	18(1)	30(1)	27(1)	1(1)	4(1)	2(1)
**C(23)**	26(1)	22(1)	25(1)	1(1)	8(1)	-1(1)
**C(24)**	28(1)	18(1)	22(1)	3(1)	6(1)	6(1)
**C(25)**	19(1)	20(1)	17(1)	1(1)	3(1)	6(1)
**C(26)**	18(1)	18(1)	15(1)	1(1)	2(1)	5(1)
**C(27)**	22(1)	19(1)	20(1)	1(1)	6(1)	5(1)
**C(28)**	28(1)	21(1)	21(1)	0(1)	4(1)	8(1)
**C(29)**	33(1)	26(1)	20(1)	-3(1)	3(1)	9(1)
**C(30)**	34(1)	28(1)	20(1)	1(1)	11(1)	8(1)
**C(31)**	28(1)	23(1)	17(1)	2(1)	5(1)	7(1)
**C(32)**	16(1)	17(1)	18(1)	2(1)	3(1)	5(1)
**C(33)**	18(1)	20(1)	19(1)	1(1)	4(1)	5(1)
**C(34)**	18(1)	22(1)	24(1)	1(1)	6(1)	3(1)
**C(35)**	23(1)	18(1)	28(1)	0(1)	5(1)	2(1)
**C(36)**	22(1)	21(1)	20(1)	-2(1)	3(1)	7(1)
**C(37)**	17(1)	21(1)	19(1)	1(1)	4(1)	6(1)
**C1_1**	21(1)	26(1)	26(1)	5(1)	5(1)	4(1)
**C2_1**	30(1)	21(1)	29(1)	3(1)	5(1)	6(1)
**C3_1**	24(1)	30(1)	30(1)	8(1)	5(1)	10(1)
**C4_1**	28(1)	33(1)	26(1)	2(1)	0(1)	7(1)
**C5_1**	39(1)	25(1)	36(1)	-2(1)	4(1)	10(1)
**C6_1**	27(1)	28(1)	34(1)	8(1)	4(1)	13(1)
**C7_1**	40(1)	40(1)	34(1)	5(1)	-7(1)	7(1)

3.2.2. Crystal structure determination of $\mathbf{Y_{pOMe}PCy_2}$ (2)

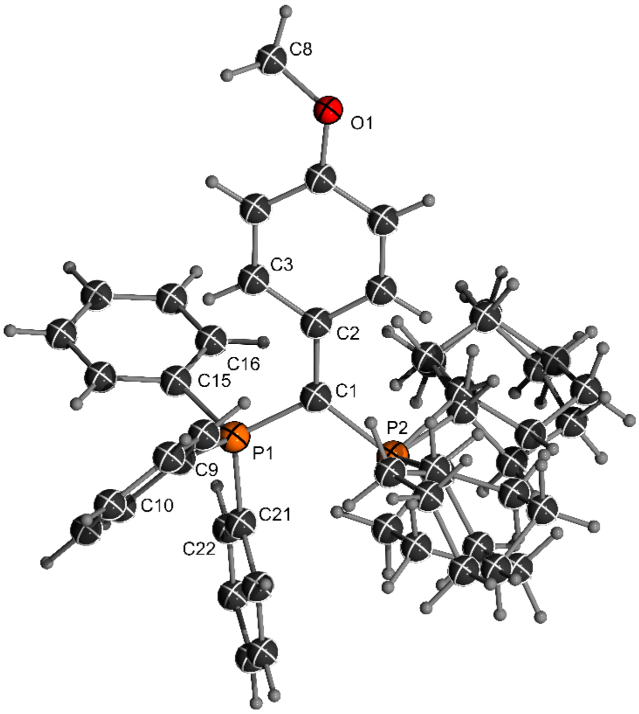

Figure 3.2: ORTEP of $\mathbf{Y_{pOMe}PCy_2}$ **(2).** Ellipsoids are drawn at the 50% probability level.

Table 3.7: Atomic coordinates (x 10^4) and equivalent isotropic displacement parameters ($Å^2$x 10^3) for $\mathbf{Y_{pOMe}PCy_2}$ **(2).** U(eq) is defined as one third of the trace of the orthogonalized Uij tensor.

	x	y	z	U(eq)
**P(1)**	7498(1)	3250(1)	5121(1)	21(1)
**P(2)**	6268(1)	1626(1)	5375(1)	29(1)
**O(1)**	2825(2)	3906(1)	2909(1)	31(1)
**C(1)**	6272(2)	2651(2)	4924(2)	24(1)
**C(2)**	5411(2)	2998(2)	4392(2)	23(1)
**C(3)**	5456(2)	3831(2)	4105(2)	26(1)
**C(4)**	4621(2)	4155(2)	3615(2)	26(1)
**C(5)**	3688(2)	3649(2)	3392(2)	25(1)
**C(6)**	3620(3)	2824(2)	3662(2)	32(1)
**C(7)**	4455(2)	2508(2)	4144(2)	30(1)
**C(8)**	2662(3)	4796(2)	2825(2)	35(1)
**C(9)**	8521(2)	3351(2)	4347(2)	24(1)
**C(10)**	9674(2)	3632(2)	4450(2)	28(1)
**C(11)**	10470(2)	3610(2)	3874(2)	31(1)
**C(12)**	10125(3)	3319(2)	3183(2)	33(1)
**C(13)**	8975(3)	3042(2)	3077(2)	35(1)
**C(14)**	8184(3)	3055(2)	3653(2)	30(1)
**C(15)**	7147(2)	4331(2)	5432(2)	23(1)
**C(16)**	6142(2)	4422(2)	5871(2)	27(1)
**C(17)**	5835(3)	5213(2)	6146(2)	32(1)
**C(18)**	6511(3)	5924(2)	5989(2)	36(1)
**C(19)**	7493(3)	5841(2)	5536(2)	37(1)
**C(20)**	7819(2)	5047(2)	5262(2)	29(1)

**C(21)**	8403(2)	2810(2)	5851(2)	23(1)
**C(22)**	8365(2)	3140(2)	6563(2)	25(1)
**C(23)**	9072(3)	2799(2)	7115(2)	30(1)
**C(24)**	9818(3)	2123(2)	6961(2)	31(1)
**C(25)**	9870(3)	1799(2)	6245(2)	31(1)
**C(26)**	9175(2)	2137(2)	5696(2)	28(1)
**C(27A)**	4866(15)	1412(8)	5794(8)	35(4)
**C(28A)**	4960(7)	759(4)	6423(4)	41(2)
**C(29A)**	3771(8)	579(5)	6776(5)	56(2)
**C(30A)**	3213(17)	1393(11)	7054(10)	61(5)
**C(31A)**	3184(16)	2084(13)	6470(13)	55(6)
**C(32A)**	4370(14)	2243(10)	6101(10)	34(4)
**C(27B)**	4660(18)	1534(10)	5732(9)	23(3)
**C(28B)**	4340(19)	2236(10)	6275(13)	29(4)
**C(29B)**	3097(14)	2131(10)	6595(14)	29(3)
**C(30B)**	3023(17)	1265(10)	6986(10)	44(4)
**C(31B)**	3299(9)	552(6)	6433(7)	48(2)
**C(32B)**	4516(8)	659(5)	6087(6)	41(2)
**C(33A)**	6314(12)	837(7)	4563(8)	38(2)
**C(34A)**	5884(7)	-56(4)	4762(4)	47(1)
**C(35A)**	6044(8)	-672(5)	4134(5)	60(2)
**C(36A)**	7340(8)	-711(6)	3919(6)	58(2)
**C(37A)**	7814(7)	158(5)	3694(4)	53(2)
**C(38A)**	7627(5)	791(4)	4331(4)	44(1)
**C(33B)**	6285(12)	797(9)	4679(8)	28(2)
**C(34B)**	6858(7)	0(4)	5023(4)	39(2)
**C(35B)**	6965(8)	-727(5)	4483(5)	46(2)
**C(36B)**	7684(10)	-445(7)	3800(6)	47(2)
**C(37B)**	7091(9)	312(5)	3434(5)	46(2)
**C(38B)**	6936(7)	1051(4)	3980(4)	36(2)

Table 3.8: Anisotropic displacement parameters (Å^2 x 10^3) for **Y$_{pOMe}$PCy$_2$ (2).** The anisotropic displacement factor exponent takes the form: $–2\pi^2$ [$h^2a^{\cdot 2}U^{11}$ + ... + 2 h k a·b·U^{12}].

	U^{11}	U^{22}	U^{22}	U^{23}	U^{13}	U^{12}
**P(1)**	17(1)	21(1)	25(1)	-2(1)	0(1)	-1(1)
**P(2)**	26(1)	23(1)	38(1)	3(1)	-5(1)	-4(1)
**O(1)**	22(1)	36(1)	34(1)	1(1)	-4(1)	1(1)
**C(1)**	19(1)	21(1)	32(1)	-1(1)	-1(1)	-1(1)
**C(2)**	18(1)	23(1)	28(1)	-4(1)	2(1)	1(1)
**C(3)**	23(1)	28(1)	27(1)	0(1)	-1(1)	-6(1)
**C(4)**	25(1)	28(1)	25(1)	1(1)	1(1)	-2(1)
**C(5)**	18(1)	29(1)	27(1)	-3(1)	1(1)	2(1)
**C(6)**	23(1)	28(1)	45(2)	-2(1)	-9(1)	-5(1)
**C(7)**	25(1)	22(1)	43(2)	-2(1)	-6(1)	-2(1)
**C(8)**	28(2)	39(2)	38(2)	5(1)	0(1)	5(1)
**C(9)**	20(1)	26(1)	27(1)	-2(1)	1(1)	0(1)
**C(10)**	25(1)	33(1)	28(1)	-4(1)	-1(1)	-5(1)
**C(11)**	22(1)	37(2)	35(2)	-2(1)	2(1)	-5(1)
**C(12)**	27(1)	44(2)	29(2)	-4(1)	6(1)	1(1)

**C(13)**	30(1)	48(2)	27(2)	-8(1)	-2(1)	3(1)
**C(14)**	22(1)	38(1)	30(2)	-5(1)	-2(1)	0(1)
**C(15)**	24(1)	22(1)	24(1)	1(1)	-4(1)	2(1)
**C(16)**	24(1)	27(1)	31(1)	0(1)	-1(1)	2(1)
**C(17)**	32(1)	34(2)	31(1)	-2(1)	-4(1)	12(1)
**C(18)**	42(2)	24(1)	41(2)	-5(1)	-13(1)	10(1)
**C(19)**	42(2)	24(1)	45(2)	1(1)	-12(1)	-3(1)
**C(20)**	28(1)	25(1)	32(2)	0(1)	-4(1)	-3(1)
**C(21)**	18(1)	22(1)	29(1)	0(1)	1(1)	-3(1)
**C(22)**	20(1)	28(1)	28(1)	-3(1)	2(1)	0(1)
**C(23)**	27(1)	38(2)	25(1)	-2(1)	-1(1)	-2(1)
**C(24)**	26(1)	32(1)	36(2)	5(1)	-5(1)	0(1)
**C(25)**	26(1)	26(1)	42(2)	-3(1)	-3(1)	4(1)
**C(26)**	26(1)	26(1)	31(1)	-5(1)	-2(1)	3(1)
**C(27A)**	34(8)	26(4)	46(6)	1(4)	-14(4)	-6(4)
**C(28A)**	50(4)	30(3)	44(4)	10(3)	-5(3)	-5(3)
**C(29A)**	61(5)	53(4)	55(5)	19(4)	-5(4)	-23(4)
**C(30A)**	52(9)	79(8)	52(7)	8(5)	23(7)	-24(6)
**C(31A)**	42(8)	81(11)	41(7)	13(6)	12(4)	6(6)
**C(32A)**	21(4)	47(6)	33(8)	15(4)	0(4)	-8(3)
**C(27B)**	14(5)	30(5)	26(5)	10(4)	4(4)	-3(4)
**C(28B)**	32(6)	25(5)	29(9)	-3(5)	12(5)	1(4)
**C(29B)**	13(5)	37(6)	36(8)	-2(4)	8(4)	-3(4)
**C(30B)**	20(5)	61(8)	49(7)	26(6)	-1(4)	1(6)
**C(31B)**	39(5)	45(5)	61(6)	15(4)	13(5)	-14(4)
**C(32B)**	38(4)	33(4)	51(5)	12(4)	9(4)	-4(3)
**C(33A)**	41(3)	23(3)	48(5)	-8(3)	4(3)	8(3)
**C(34A)**	58(3)	25(2)	59(3)	-5(2)	-10(3)	-2(2)
**C(35A)**	80(4)	35(3)	66(4)	-18(3)	-14(3)	3(3)
**C(36A)**	76(4)	43(4)	53(4)	-16(3)	-18(3)	27(3)
**C(37A)**	57(4)	55(3)	48(3)	-19(3)	-5(3)	22(3)
**C(38A)**	36(3)	46(3)	50(3)	-15(3)	-2(2)	11(2)
**C(33B)**	24(3)	28(3)	32(4)	-3(3)	-14(3)	1(3)
**C(34B)**	38(3)	30(3)	49(3)	3(3)	1(3)	7(2)
**C(35B)**	53(4)	34(3)	52(4)	-1(3)	-5(3)	15(3)
**C(36B)**	57(5)	40(4)	44(4)	-5(4)	-3(3)	15(4)
**C(37B)**	62(5)	37(3)	39(3)	-10(3)	-6(3)	11(3)
**C(38B)**	41(3)	31(3)	36(3)	-6(2)	-2(3)	6(3)

3.2.3. Crystal structure determination of **$Y_{pCF3}PCy_2$ (3)**

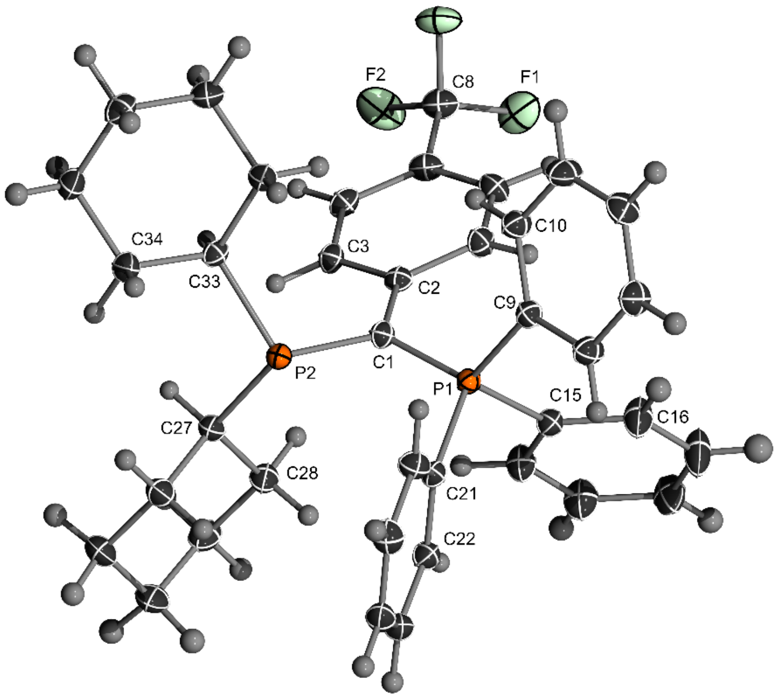

Figure 3.3: ORTEP of **$Y_{pCF3}PCy_2$.(3)** Ellipsoids are drawn at the 50% probability level.

Table 3.9: Atomic coordinates (x 10^4) and equivalent isotropic displacement parameters (Å^{2}x 10^3) for **$Y_{pCF3}PCy_2$ (2).** U(eq) is defined as one third of the trace of the orthogonalized Uij tensor.

	x	y	z	U(eq)
**P(1)**	2535(1)	4002(1)	4945(1)	15(1)
**P(2)**	2934(1)	3197(1)	6034(1)	15(1)
**F(1)**	8620(1)	4702(1)	9505(1)	39(1)
**F(2)**	8815(1)	4163(1)	10658(1)	41(1)
**F(3)**	9706(1)	4207(1)	8848(1)	33(1)
**C(1)**	3531(1)	3705(1)	6120(1)	16(1)
**C(2)**	4765(1)	3867(1)	6969(1)	16(1)
**C(3)**	5392(1)	3672(1)	8158(1)	18(1)
**C(4)**	6603(2)	3810(1)	8945(1)	20(1)
**C(5)**	7227(1)	4161(1)	8617(1)	20(1)
**C(6)**	6614(2)	4368(1)	7482(1)	22(1)
**C(7)**	5431(2)	4223(1)	6672(1)	20(1)
**C(8)**	8564(2)	4306(1)	9404(2)	23(1)
**C(9)**	3171(1)	4092(1)	3362(1)	18(1)
**C(10)**	4550(2)	3991(1)	3244(2)	22(1)
**C(11)**	5067(2)	4068(1)	2060(2)	28(1)
**C(12)**	4214(2)	4242(1)	983(2)	27(1)
**C(13)**	2828(2)	4338(1)	1086(2)	26(1)
**C(14)**	2309(2)	4264(1)	2267(2)	23(1)
**C(15)**	2280(1)	4491(1)	5632(1)	19(1)
**C(16)**	2274(2)	4833(1)	4875(2)	32(1)
**C(17)**	2020(2)	5197(1)	5424(2)	41(1)
**C(18)**	1755(2)	5224(1)	6724(2)	33(1)

**C(19)**	1772(2)	4883(1)	7490(2)	31(1)
**C(20)**	2045(2)	4519(1)	6956(2)	25(1)
**C(21)**	778(1)	3802(1)	4435(1)	17(1)
**C(22)**	-276(1)	3877(1)	5209(1)	20(1)
**C(23)**	-1619(2)	3720(1)	4834(2)	24(1)
**C(24)**	-1921(2)	3495(1)	3681(2)	25(1)
**C(25)**	-876(2)	3418(1)	2911(2)	25(1)
**C(26)**	472(2)	3568(1)	3289(1)	22(1)
**C(27)**	2211(1)	3077(1)	7621(1)	18(1)
**C(28)**	1782(2)	3437(1)	8388(1)	22(1)
**C(29)**	1185(2)	3310(1)	9645(2)	26(1)
**C(30)**	-67(2)	3030(1)	9291(2)	31(1)
**C(31)**	308(2)	2673(1)	8491(2)	28(1)
**C(32)**	928(2)	2803(1)	7255(2)	23(1)
**C(33)**	4562(1)	2887(1)	6298(1)	17(1)
**C(34)**	4138(2)	2450(1)	6247(2)	23(1)
**C(35)**	5420(2)	2176(1)	6389(2)	26(1)
**C(36)**	6349(2)	2269(1)	5341(2)	25(1)
**C(37)**	6797(2)	2700(1)	5421(2)	26(1)
**C(38)**	5510(2)	2975(1)	5255(2)	23(1)

Table 3.10: Anisotropic displacement parameters (Å^2 x 10^3) for **Y$_{pCF3}$PCy$_2$ (3).** The anisotropic displacement factor exponent takes the form: $-2\pi^2$ [$h^2a^{*2}U^{11}$ + ... + $2 h k a^* b^* U^{12}$].

	**U^{11}**	**U^{22}**	**U^{22}**	**U^{23}**	**U^{13}**	**U^{12}**
**P(1)**	14(1)	15(1)	15(1)	1(1)	2(1)	1(1)
**P(2)**	15(1)	15(1)	16(1)	1(1)	2(1)	0(1)
**F(1)**	33(1)	29(1)	51(1)	-16(1)	-8(1)	-3(1)
**F(2)**	33(1)	63(1)	22(1)	2(1)	-7(1)	-14(1)
**F(3)**	17(1)	44(1)	39(1)	-9(1)	5(1)	-1(1)
**C(1)**	17(1)	17(1)	15(1)	1(1)	2(1)	0(1)
**C(2)**	17(1)	17(1)	16(1)	-2(1)	4(1)	1(1)
**C(3)**	20(1)	16(1)	18(1)	-1(1)	3(1)	1(1)
**C(4)**	21(1)	20(1)	17(1)	-1(1)	1(1)	4(1)
**C(5)**	16(1)	24(1)	19(1)	-5(1)	2(1)	0(1)
**C(6)**	21(1)	22(1)	22(1)	-1(1)	4(1)	-4(1)
**C(7)**	20(1)	21(1)	18(1)	2(1)	1(1)	-1(1)
**C(8)**	20(1)	27(1)	23(1)	-6(1)	3(1)	0(1)
**C(9)**	18(1)	18(1)	18(1)	0(1)	3(1)	0(1)
**C(10)**	20(1)	25(1)	21(1)	2(1)	3(1)	3(1)
**C(11)**	24(1)	36(1)	26(1)	2(1)	10(1)	6(1)
**C(12)**	31(1)	31(1)	20(1)	2(1)	10(1)	0(1)
**C(13)**	28(1)	31(1)	19(1)	5(1)	2(1)	2(1)
**C(14)**	20(1)	28(1)	21(1)	4(1)	3(1)	2(1)
**C(15)**	18(1)	17(1)	21(1)	0(1)	4(1)	1(1)
**C(16)**	52(1)	21(1)	29(1)	4(1)	20(1)	7(1)
**C(17)**	72(1)	19(1)	40(1)	7(1)	28(1)	8(1)
**C(18)**	46(1)	19(1)	37(1)	-4(1)	16(1)	2(1)
**C(19)**	44(1)	25(1)	23(1)	-3(1)	10(1)	1(1)

**C(20)**	33(1)	20(1)	21(1)	0(1)	5(1)	0(1)
**C(21)**	16(1)	16(1)	19(1)	4(1)	1(1)	1(1)
**C(22)**	19(1)	19(1)	22(1)	0(1)	3(1)	1(1)
**C(23)**	17(1)	21(1)	34(1)	3(1)	6(1)	1(1)
**C(24)**	18(1)	22(1)	32(1)	5(1)	-1(1)	-2(1)
**C(25)**	26(1)	24(1)	23(1)	-1(1)	-2(1)	-4(1)
**C(26)**	21(1)	24(1)	20(1)	0(1)	3(1)	-1(1)
**C(27)**	17(1)	19(1)	20(1)	2(1)	5(1)	1(1)
**C(28)**	25(1)	21(1)	21(1)	1(1)	8(1)	2(1)
**C(29)**	31(1)	27(1)	23(1)	2(1)	11(1)	8(1)
**C(30)**	28(1)	34(1)	35(1)	8(1)	18(1)	4(1)
**C(31)**	24(1)	28(1)	33(1)	6(1)	10(1)	-3(1)
**C(32)**	21(1)	25(1)	25(1)	2(1)	6(1)	-3(1)
**C(33)**	17(1)	17(1)	16(1)	0(1)	3(1)	2(1)
**C(34)**	23(1)	18(1)	29(1)	1(1)	7(1)	1(1)
**C(35)**	30(1)	18(1)	32(1)	4(1)	10(1)	5(1)
**C(36)**	26(1)	24(1)	26(1)	-1(1)	6(1)	8(1)
**C(37)**	21(1)	26(1)	31(1)	2(1)	9(1)	4(1)
**C(38)**	22(1)	22(1)	26(1)	4(1)	9(1)	3(1)

3.2.4. Crystal structure determination of **Y$_{oTol}$PCy$_2$ (4)**

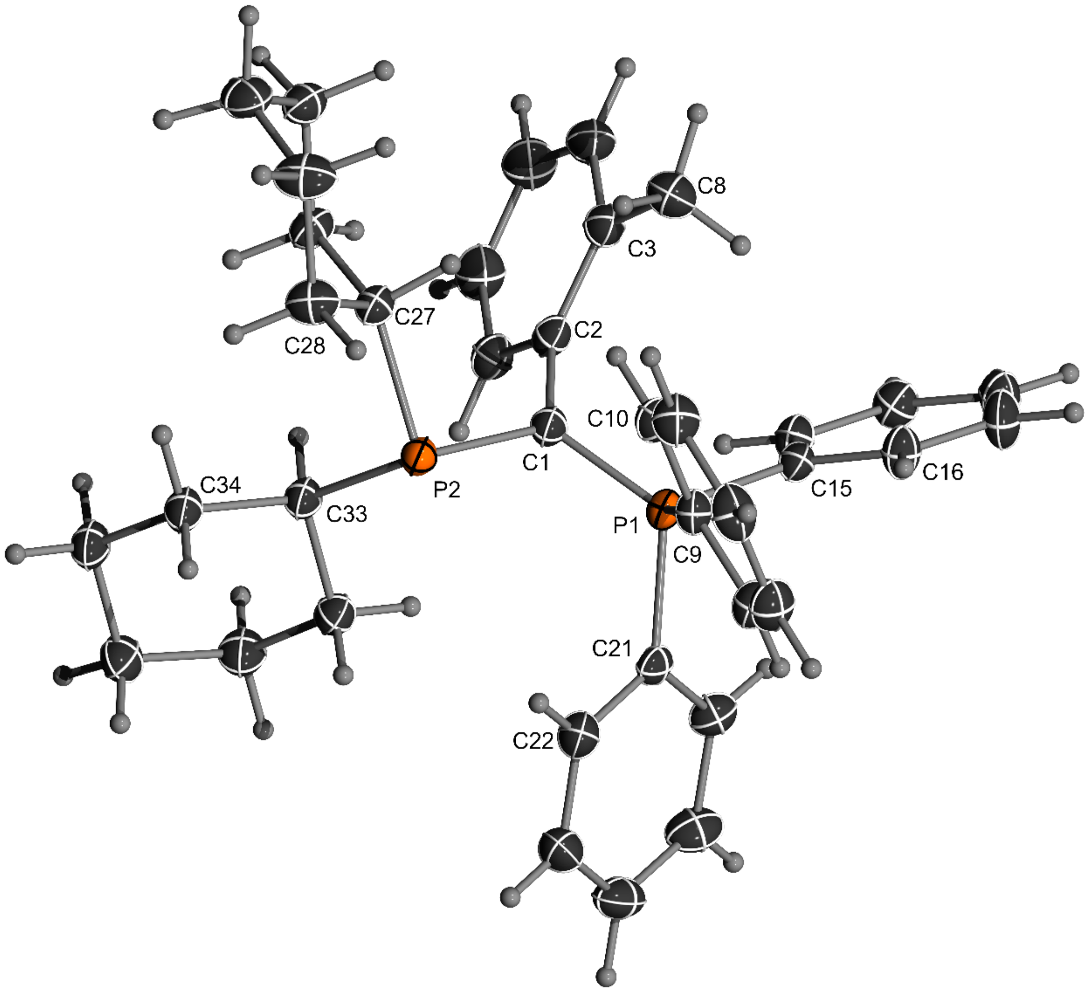

Figure 3.4:ORTEP of **Y$_{oTol}$PCy$_2$.(4)** Ellipsoids are drawn at the 50% probability level.

Table 3.11: Atomic coordinates (x 10^4) and equivalent isotropic displacement parameters ($Å^2$x 10^3) for **Y$_{oTol}$PCy$_2$ (4).** U(eq) is defined as one third of the trace of the orthogonalized Uij tensor.

	**x**	**y**	**z**	**U(eq)**
**P(1)**	5371(1)	4005(1)	6270(1)	18(1)
**P(2)**	2863(1)	3367(1)	6599(1)	20(1)
**C(1)**	3767(2)	4394(2)	6254(1)	21(1)
**C(2)**	3138(2)	5481(2)	5986(1)	23(1)
**C(3)**	3296(2)	6756(2)	6129(1)	27(1)
**C(4)**	2550(2)	7696(2)	5886(1)	34(1)
**C(5)**	1673(2)	7421(2)	5506(1)	38(1)
**C(6)**	1530(2)	6174(2)	5351(1)	34(1)
**C(7)**	2259(2)	5229(2)	5589(1)	27(1)
**C(8)**	4225(2)	7139(2)	6539(1)	33(1)
**C(9)**	6212(2)	3794(2)	6854(1)	21(1)
**C(10)**	5722(2)	4391(2)	7226(1)	26(1)
**C(11)**	6333(2)	4233(2)	7676(1)	31(1)
**C(12)**	7433(2)	3473(2)	7755(1)	31(1)
**C(13)**	7932(2)	2877(2)	7386(1)	31(1)

**C(14)**	7331(2)	3038(2)	6938(1)	26(1)
**C(15)**	6268(2)	5196(2)	5980(1)	21(1)
**C(16)**	7484(2)	5628(2)	6174(1)	27(1)
**C(17)**	8171(2)	6511(2)	5936(1)	33(1)
**C(18)**	7653(2)	6969(2)	5510(1)	30(1)
**C(19)**	6440(2)	6554(2)	5317(1)	26(1)
**C(20)**	5748(2)	5668(2)	5547(1)	24(1)
**C(21)**	5744(2)	2509(2)	5988(1)	21(1)
**C(22)**	5468(2)	1365(2)	6201(1)	29(1)
**C(23)**	5627(2)	223(2)	5979(1)	35(1)
**C(24)**	6054(2)	202(2)	5540(1)	34(1)
**C(25)**	6334(2)	1324(2)	5329(1)	33(1)
**C(26)**	6193(2)	2478(2)	5553(1)	26(1)
**C(27)**	2010(2)	4558(2)	6944(1)	22(1)
**C(28)**	1776(2)	4082(2)	7426(1)	30(1)
**C(29)**	1380(2)	5187(2)	7724(1)	36(1)
**C(30)**	185(2)	5884(2)	7494(1)	33(1)
**C(31)**	390(2)	6314(2)	7009(1)	27(1)
**C(32)**	786(2)	5210(2)	6711(1)	25(1)
**C(33)**	1499(2)	2665(2)	6202(1)	22(1)
**C(34)**	545(2)	1909(2)	6470(1)	25(1)
**C(35)**	-553(2)	1339(2)	6139(1)	28(1)
**C(36)**	-23(2)	484(2)	5778(1)	31(1)
**C(37)**	970(2)	1189(2)	5519(1)	31(1)
**C(38)**	2053(2)	1786(2)	5851(1)	25(1)

Table 3.12: Anisotropic displacement parameters (Å^2 x 10^3) for **Y$_{oTol}$PCy$_2$ (4).** The anisotropic displacement factor exponent takes the form: $-2\pi^2$ [$h^2a^{*2}U^{11}$ + ... + 2 h k a·b·U^{12}].

	U^{11}	U^{22}	U^{22}	U^{23}	U^{13}	U^{12}
**P(1)**	15(1)	21(1)	19(1)	0(1)	2(1)	0(1)
**P(2)**	17(1)	22(1)	23(1)	1(1)	4(1)	0(1)
**C(1)**	18(1)	22(1)	24(1)	2(1)	3(1)	0(1)
**C(2)**	20(1)	27(1)	24(1)	4(1)	6(1)	2(1)
**C(3)**	25(1)	25(1)	31(1)	2(1)	8(1)	1(1)
**C(4)**	32(1)	27(1)	44(1)	3(1)	7(1)	3(1)
**C(5)**	35(1)	34(1)	45(1)	11(1)	-1(1)	6(1)
**C(6)**	31(1)	39(1)	32(1)	7(1)	-2(1)	0(1)
**C(7)**	26(1)	30(1)	26(1)	1(1)	6(1)	0(1)
**C(8)**	36(1)	27(1)	34(1)	-1(1)	4(1)	-1(1)
**C(9)**	18(1)	25(1)	21(1)	2(1)	2(1)	-2(1)
**C(10)**	24(1)	28(1)	25(1)	0(1)	1(1)	1(1)
**C(11)**	32(1)	37(1)	22(1)	-2(1)	2(1)	-4(1)
**C(12)**	30(1)	38(1)	23(1)	5(1)	-5(1)	-7(1)
**C(13)**	23(1)	35(1)	34(1)	7(1)	-3(1)	2(1)
**C(14)**	21(1)	31(1)	26(1)	3(1)	3(1)	1(1)
**C(15)**	18(1)	22(1)	22(1)	0(1)	5(1)	0(1)
**C(16)**	21(1)	34(1)	26(1)	6(1)	-1(1)	-3(1)
**C(17)**	21(1)	41(1)	36(1)	9(1)	-1(1)	-7(1)

**C(18)**	27(1)	31(1)	32(1)	7(1)	6(1)	-3(1)
**C(19)**	27(1)	30(1)	23(1)	5(1)	3(1)	2(1)
**C(20)**	20(1)	28(1)	22(1)	0(1)	2(1)	-1(1)
**C(21)**	13(1)	24(1)	24(1)	-3(1)	-1(1)	2(1)
**C(22)**	24(1)	30(1)	35(1)	0(1)	7(1)	-1(1)
**C(23)**	30(1)	25(1)	49(1)	0(1)	5(1)	-2(1)
**C(24)**	32(1)	28(1)	42(1)	-10(1)	-3(1)	4(1)
**C(25)**	36(1)	38(1)	25(1)	-6(1)	-1(1)	8(1)
**C(26)**	26(1)	29(1)	23(1)	0(1)	1(1)	5(1)
**C(27)**	19(1)	25(1)	22(1)	-1(1)	2(1)	1(1)
**C(28)**	37(1)	29(1)	23(1)	1(1)	4(1)	5(1)
**C(29)**	53(1)	34(1)	22(1)	-1(1)	6(1)	6(1)
**C(30)**	39(1)	30(1)	32(1)	-6(1)	12(1)	2(1)
**C(31)**	23(1)	30(1)	29(1)	-4(1)	2(1)	4(1)
**C(32)**	21(1)	31(1)	24(1)	-3(1)	1(1)	4(1)
**C(33)**	18(1)	26(1)	24(1)	-1(1)	3(1)	-1(1)
**C(34)**	20(1)	30(1)	26(1)	-3(1)	4(1)	-3(1)
**C(35)**	20(1)	34(1)	30(1)	-4(1)	3(1)	-4(1)
**C(36)**	26(1)	34(1)	34(1)	-10(1)	2(1)	-6(1)
**C(37)**	31(1)	36(1)	27(1)	-8(1)	6(1)	-3(1)
**C(38)**	23(1)	27(1)	27(1)	-3(1)	6(1)	-1(1)

3.2.5. Crystal structure determination of **$Y_{Mes}PCy_2$ (5)**

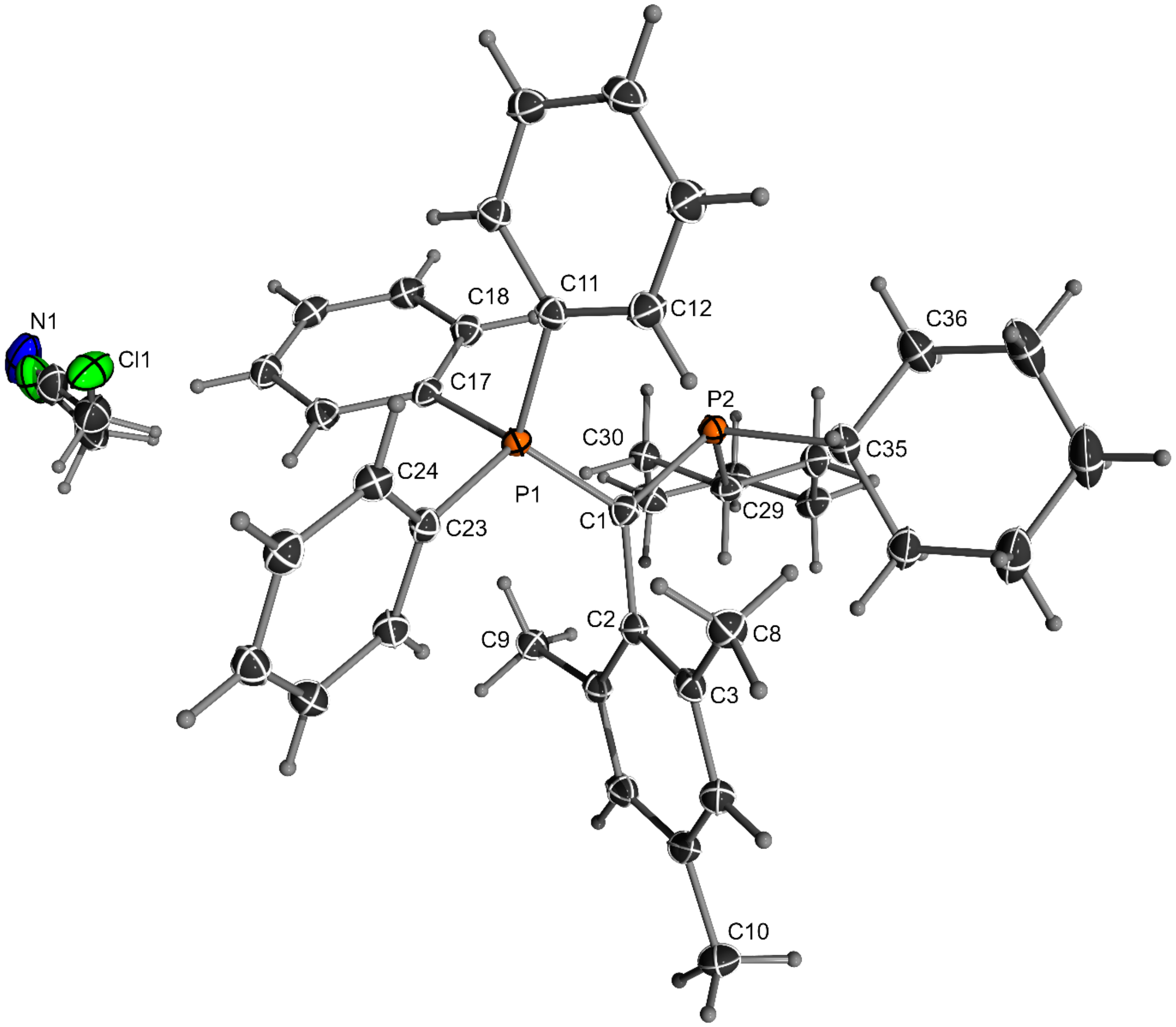

Figure 3.5: ORTEP of **$Y_{Mes}PCy_2$.(5)** Ellipsoids are drawn at the 50% probability level.

Table 3.13: Atomic coordinates (x 10^4) and equivalent isotropic displacement parameters ($Å^2$x 10^3) for **$Y_{Mes}PCy_2$ (5).** U(eq) is defined as one third of the trace of the orthogonalized U^{ij} tensor.

	x	y	z	U(eq)
**P(1)**	3774(1)	1600(1)	6403(1)	13(1)
**P(2)**	1499(1)	3165(1)	6762(1)	15(1)
**C(1)**	3105(1)	2381(1)	7118(1)	16(1)
**C(2)**	3809(1)	2458(1)	7908(1)	15(1)
**C(3)**	3764(1)	1657(1)	8991(1)	17(1)
**C(4)**	4218(1)	1861(1)	9746(1)	20(1)
**C(5)**	4782(1)	2812(1)	9470(1)	21(1)
**C(6)**	4950(1)	3516(1)	8405(1)	19(1)
**C(7)**	4484(1)	3360(1)	7623(1)	17(1)
**C(8)**	3250(1)	557(1)	9368(1)	20(1)
**C(9)**	4789(1)	4149(1)	6488(1)	20(1)
**C(10)**	5185(1)	3065(1)	10304(1)	28(1)

**C(11)**	2742(1)	717(1)	6345(1)	16(1)
**C(12)**	2028(1)	148(1)	7255(1)	21(1)
**C(13)**	1203(1)	-500(1)	7242(1)	23(1)
**C(14)**	1076(1)	-593(1)	6323(1)	21(1)
**C(15)**	1796(1)	-48(1)	5421(1)	20(1)
**C(16)**	2630(1)	598(1)	5432(1)	18(1)
**C(17)**	4203(1)	2377(1)	4993(1)	15(1)
**C(18)**	3250(1)	3193(1)	4316(1)	19(1)
**C(19)**	3508(1)	3740(1)	3238(1)	21(1)
**C(20)**	4726(1)	3483(1)	2818(1)	21(1)
**C(21)**	5681(1)	2693(1)	3482(1)	21(1)
**C(22)**	5424(1)	2140(1)	4565(1)	19(1)
**C(23)**	5261(1)	598(1)	6932(1)	15(1)
**C(24)**	5512(1)	-578(1)	7085(1)	18(1)
**C(25)**	6682(1)	-1314(1)	7425(1)	20(1)
**C(26)**	7618(1)	-889(1)	7615(1)	21(1)
**C(27)**	7383(1)	280(1)	7458(1)	21(1)
**C(28)**	6216(1)	1021(1)	7120(1)	19(1)
**C(29)**	1440(1)	4743(1)	6456(1)	17(1)
**C(30)**	1943(1)	5346(1)	5308(1)	19(1)
**C(31)**	1988(1)	6627(1)	4996(1)	23(1)
**C(32)**	677(1)	7338(1)	5132(1)	23(1)
**C(33)**	124(1)	6747(1)	6253(1)	23(1)
**C(34)**	104(1)	5458(1)	6556(1)	20(1)
**C(35)**	504(1)	2595(1)	8012(1)	21(1)
**C(36)**	-887(1)	2753(1)	7801(1)	27(1)
**C(37)**	-1582(1)	2016(1)	8809(1)	36(1)
**C(38)**	-1497(2)	2339(2)	9705(1)	42(1)
**C(39)**	-125(1)	2204(2)	9917(1)	37(1)
**C(40)**	588(1)	2923(1)	8913(1)	25(1)
**N51**	8533(6)	5871(5)	-1001(4)	58(2)
**C61**	8325(2)	5379(3)	-133(3)	40(1)
**C71**	8019(3)	4711(3)	977(2)	42(1)
**Cl11**	7648(2)	3934(2)	1196(2)	40(1)
**Cl21**	8532(5)	5824(5)	-684(5)	46(1)
**C31**	8202(9)	5249(10)	692(10)	37(2)

Table 3.14: Anisotropic displacement parameters (Å^2 x 10^3) for **Y$_{Mes}$PCy$_2$ (5).** The anisotropic displacement factor exponent takes the form: $-2\pi^2$ [$h^2a^{*2}U^{11}$ + ... + 2 h k a·b·U^{12}].

	U^{11}	U^{22}	U^{22}	U^{23}	U^{13}	U^{12}
**P(1)**	13(1)	13(1)	14(1)	-6(1)	0(1)	-3(1)
**P(2)**	14(1)	15(1)	16(1)	-7(1)	-1(1)	-3(1)
**C(1)**	16(1)	15(1)	16(1)	-7(1)	-1(1)	-3(1)
**C(2)**	13(1)	16(1)	18(1)	-9(1)	-1(1)	-1(1)
**C(3)**	13(1)	19(1)	18(1)	-8(1)	-1(1)	-1(1)
**C(4)**	17(1)	24(1)	17(1)	-8(1)	-2(1)	-2(1)
**C(5)**	16(1)	28(1)	22(1)	-15(1)	-3(1)	-2(1)
**C(6)**	17(1)	20(1)	24(1)	-12(1)	-1(1)	-4(1)

**C(7)**	14(1)	17(1)	19(1)	-9(1)	-1(1)	-1(1)
**C(8)**	21(1)	19(1)	18(1)	-5(1)	-2(1)	-4(1)
**C(9)**	20(1)	19(1)	20(1)	-9(1)	0(1)	-7(1)
**C(10)**	28(1)	40(1)	25(1)	-19(1)	-3(1)	-10(1)
**C(11)**	14(1)	15(1)	19(1)	-8(1)	-1(1)	-2(1)
**C(12)**	24(1)	22(1)	19(1)	-10(1)	1(1)	-8(1)
**C(13)**	26(1)	24(1)	22(1)	-9(1)	4(1)	-12(1)
**C(14)**	20(1)	19(1)	28(1)	-11(1)	-2(1)	-6(1)
**C(15)**	21(1)	19(1)	22(1)	-11(1)	-4(1)	-2(1)
**C(16)**	17(1)	18(1)	19(1)	-9(1)	0(1)	-4(1)
**C(17)**	19(1)	14(1)	16(1)	-9(1)	1(1)	-6(1)
**C(18)**	19(1)	18(1)	20(1)	-9(1)	-1(1)	-3(1)
**C(19)**	26(1)	17(1)	19(1)	-8(1)	-5(1)	-3(1)
**C(20)**	32(1)	18(1)	16(1)	-8(1)	2(1)	-10(1)
**C(21)**	23(1)	22(1)	21(1)	-12(1)	5(1)	-8(1)
**C(22)**	19(1)	18(1)	20(1)	-9(1)	0(1)	-4(1)
**C(23)**	16(1)	16(1)	13(1)	-6(1)	0(1)	-3(1)
**C(24)**	19(1)	18(1)	17(1)	-8(1)	-1(1)	-4(1)
**C(25)**	23(1)	16(1)	19(1)	-7(1)	-2(1)	-2(1)
**C(26)**	17(1)	22(1)	20(1)	-8(1)	-2(1)	0(1)
**C(27)**	17(1)	25(1)	24(1)	-12(1)	-2(1)	-5(1)
**C(28)**	18(1)	18(1)	23(1)	-10(1)	1(1)	-5(1)
**C(29)**	17(1)	16(1)	19(1)	-8(1)	-3(1)	-2(1)
**C(30)**	20(1)	17(1)	20(1)	-7(1)	-2(1)	-4(1)
**C(31)**	23(1)	18(1)	26(1)	-7(1)	-4(1)	-6(1)
**C(32)**	27(1)	16(1)	28(1)	-9(1)	-7(1)	-2(1)
**C(33)**	25(1)	19(1)	27(1)	-13(1)	-6(1)	2(1)
**C(34)**	19(1)	18(1)	23(1)	-10(1)	-2(1)	-1(1)
**C(35)**	17(1)	21(1)	20(1)	-7(1)	2(1)	-4(1)
**C(36)**	19(1)	27(1)	30(1)	-8(1)	0(1)	-6(1)
**C(37)**	20(1)	36(1)	39(1)	-5(1)	5(1)	-8(1)
**C(38)**	27(1)	50(1)	30(1)	-6(1)	10(1)	-4(1)
**C(39)**	29(1)	45(1)	22(1)	-8(1)	4(1)	1(1)
**C(40)**	20(1)	29(1)	19(1)	-9(1)	1(1)	0(1)
**N51**	63(2)	58(2)	38(3)	-25(2)	-16(2)	27(2)
**C61**	34(1)	46(2)	39(2)	-26(1)	-9(1)	12(1)
**C71**	38(1)	51(2)	36(1)	-24(1)	-2(1)	-1(1)
**Cl11**	50(1)	46(1)	38(1)	-24(1)	14(1)	-29(1)
**Cl21**	38(1)	44(2)	41(3)	-8(2)	-1(2)	-1(1)
**C31**	25(4)	35(5)	60(8)	-27(5)	3(4)	-8(4)

3.3. Crystal structures of the gold complexes

3.3.1. Crystal structure determination of **Y$_{Ph}$PCy$_2$•AuCl**

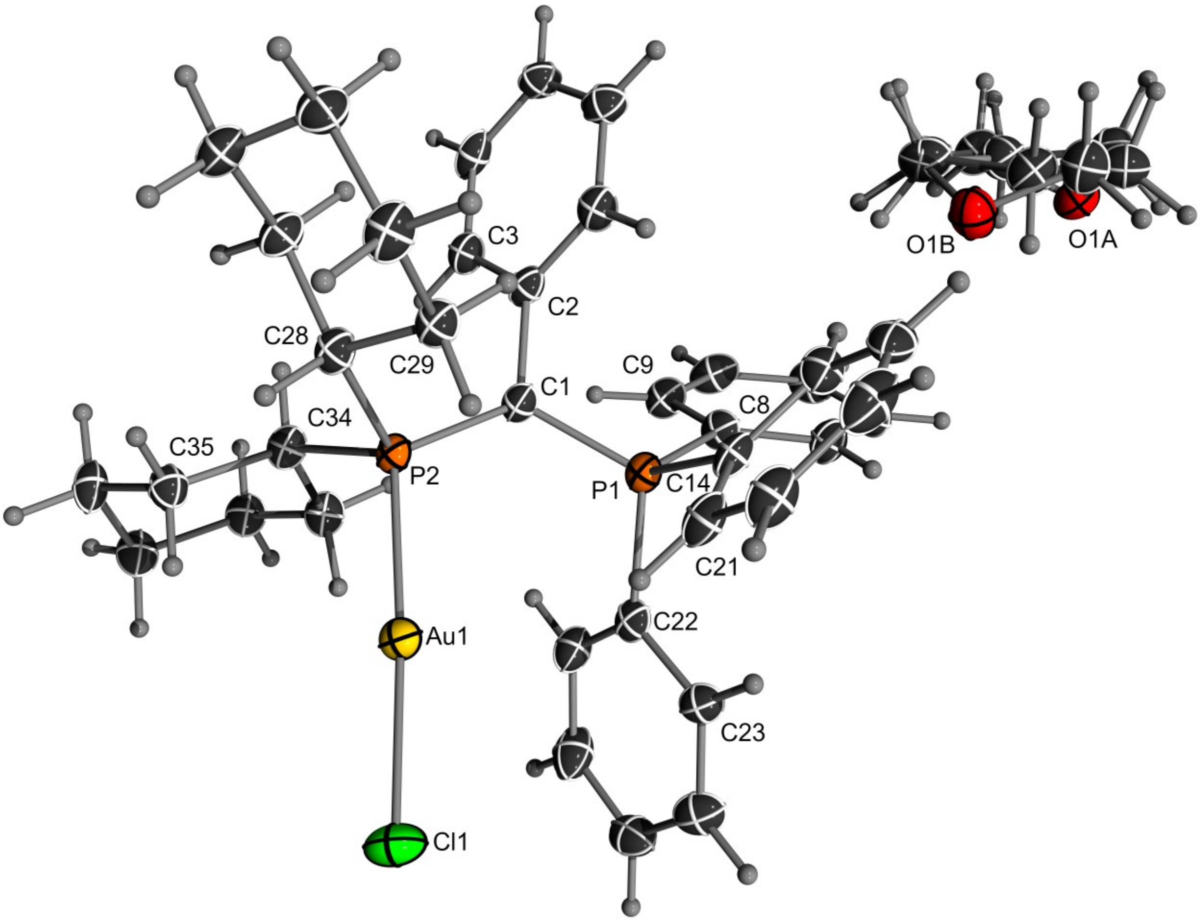

Figure 3.6: ORTEP of **Y$_{Ph}$PCy$_2$•AuCl (1•AuCl)** Ellipsoids are drawn at the 50% probability level.

Table 3.15: Atomic coordinates (x 10^4) and equivalent isotropic displacement parameters ($Å^2$x 10^3) for **Y$_{Ph}$PCy$_2$•AuCl (1•AuCl)**. U(eq) is defined as one third of the trace of the orthogonalized U^{ij} tensor.

	x	y	z	U(eq)
**Au(1)**	9918(1)	6286(1)	2171(1)	22(1)
**Cl(1)**	12158(1)	7151(1)	2626(1)	33(1)
**P(1)**	7210(1)	4364(1)	3060(1)	19(1)
**C(1)**	6616(4)	4681(4)	2222(2)	21(1)
**P(2)**	7760(1)	5476(1)	1661(1)	18(1)
**C(2)**	5256(4)	3920(4)	1900(2)	21(1)
**C(3)**	4098(5)	4545(5)	1643(2)	24(1)
**C(4)**	2833(5)	3845(5)	1348(2)	28(1)
**C(9)**	4438(5)	4792(5)	3460(3)	27(1)
**C(6)**	3797(5)	1872(5)	1568(3)	28(1)
**C(5)**	2665(5)	2515(5)	1311(2)	26(1)
**C(8)**	5653(5)	4149(5)	3607(2)	23(1)
**C(7)**	5071(5)	2581(5)	1853(2)	23(1)
**C(10)**	3237(5)	4712(6)	3877(3)	31(1)
**C(11)**	3249(6)	4024(6)	4460(3)	35(1)

**C(14)**	8238(5)	2942(4)	3062(2)	22(1)
**C(13)**	5668(5)	3498(5)	4214(2)	28(1)
**C(12)**	4455(6)	3427(5)	4635(3)	32(1)
**C(15)**	7567(5)	1755(5)	3159(2)	26(1)
**C(18)**	8341(6)	662(5)	3066(3)	31(1)
**C(19)**	9743(6)	762(6)	2862(3)	36(1)
**C(20)**	10431(6)	1944(5)	2773(3)	31(1)
**C(21)**	9674(5)	3036(5)	2862(2)	28(1)
**C(22)**	8298(5)	5696(4)	3603(2)	22(1)
**C(23)**	9485(5)	5489(5)	4044(2)	24(1)
**C(24)**	10192(5)	6503(5)	4513(3)	30(1)
**C(25)**	9712(6)	7735(5)	4564(3)	33(1)
**C(26)**	8500(7)	7935(5)	4152(3)	37(1)
**C(27)**	7794(6)	6915(5)	3672(3)	29(1)
**C(28)**	8088(5)	4366(4)	824(2)	22(1)
**C(29)**	8597(5)	3105(4)	1033(2)	22(1)
**C(30)**	9006(5)	2188(5)	373(2)	27(1)
**C(31)**	7809(5)	1961(5)	-223(3)	28(1)
**C(32)**	7356(5)	3230(5)	-421(2)	28(1)
**C(33)**	6854(5)	4075(5)	245(2)	25(1)
**C(34)**	6832(5)	6778(4)	1318(2)	22(1)
**C(35)**	7786(5)	7457(5)	796(2)	25(1)
**C(36)**	7063(6)	8580(5)	540(3)	30(1)
**C(37)**	6701(6)	9567(5)	1185(3)	30(1)
**C(38)**	5745(5)	8904(4)	1703(3)	25(1)
**C(39)**	6440(5)	7756(4)	1955(2)	24(1)
**O(1A)**	2911(7)	-153(6)	4677(3)	32(2)
**C(1A)**	3844(12)	-1115(14)	4419(7)	34(2)
**C(2A)**	4609(10)	-634(11)	3785(5)	34(2)
**C(3A)**	3522(19)	270(30)	3508(13)	31(2)
**C(4A)**	2309(12)	242(14)	4034(6)	34(2)
**O(1B)**	4960(10)	100(11)	3666(6)	37(2)
**C(1B)**	4799(15)	-1097(14)	3948(9)	35(3)
**C(2B)**	3441(19)	-1080(20)	4376(11)	33(3)
**C(3B)**	2626(19)	20(20)	4116(11)	36(3)
**C(4B)**	3520(30)	370(50)	3490(20)	31(3)

Table 3.16: Anisotropic displacement parameters ($Å^2$ x 10^3) for **Y$_{Ph}$PCy$_2$•AuCl (1•AuCl).** The anisotropic displacement factor exponent takes the form: $-2\pi^2$ [$h^2a^{\cdot 2}U^{11}$ + ... + 2 h k $a\cdot b\cdot U^{12}$].

	U^{11}	U^{22}	U^{22}	U^{23}	U^{13}	U^{12}
**Au(1)**	18(1)	26(1)	20(1)	1(1)	3(1)	2(1)
**Cl(1)**	22(1)	39(1)	35(1)	-4(1)	-3(1)	-2(1)
**P(1)**	17(1)	24(1)	17(1)	3(1)	4(1)	4(1)
**C(1)**	18(2)	29(2)	17(2)	5(2)	1(1)	3(2)
**P(2)**	16(1)	22(1)	16(1)	2(1)	3(1)	3(1)
**C(2)**	18(2)	29(2)	16(2)	3(2)	2(2)	3(2)
**C(3)**	23(2)	27(2)	22(2)	3(2)	4(2)	5(2)
**C(4)**	19(2)	47(3)	21(2)	12(2)	3(2)	9(2)

**C(9)**	22(2)	35(2)	23(2)	3(2)	3(2)	4(2)
**C(6)**	26(2)	33(2)	24(2)	1(2)	2(2)	1(2)
**C(5)**	19(2)	32(2)	25(2)	3(2)	-1(2)	-1(2)
**C(8)**	18(2)	33(2)	18(2)	3(2)	6(2)	0(2)
**C(7)**	19(2)	29(2)	22(2)	4(2)	5(2)	5(2)
**C(10)**	21(2)	45(3)	26(2)	-4(2)	4(2)	5(2)
**C(11)**	28(2)	49(3)	26(2)	0(2)	15(2)	-4(2)
**C(14)**	22(2)	29(2)	17(2)	3(2)	-1(2)	10(2)
**C(13)**	30(2)	34(2)	20(2)	5(2)	5(2)	5(2)
**C(12)**	38(3)	33(2)	24(2)	4(2)	11(2)	-2(2)
**C(15)**	28(2)	26(2)	24(2)	3(2)	2(2)	7(2)
**C(18)**	42(3)	22(2)	28(2)	-1(2)	-8(2)	3(2)
**C(19)**	43(3)	39(3)	28(2)	-3(2)	-6(2)	24(2)
**C(20)**	32(2)	35(3)	27(2)	3(2)	1(2)	17(2)
**C(21)**	29(2)	39(3)	16(2)	4(2)	3(2)	10(2)
**C(22)**	20(2)	29(2)	17(2)	4(2)	6(2)	3(2)
**C(23)**	19(2)	30(2)	21(2)	1(2)	3(2)	1(2)
**C(24)**	20(2)	41(3)	27(2)	-1(2)	5(2)	2(2)
**C(25)**	37(3)	35(3)	24(2)	-3(2)	6(2)	-8(2)
**C(26)**	53(3)	34(3)	24(2)	3(2)	2(2)	9(2)
**C(27)**	34(2)	32(2)	21(2)	5(2)	-1(2)	6(2)
**C(28)**	21(2)	26(2)	19(2)	2(2)	5(2)	4(2)
**C(29)**	19(2)	28(2)	21(2)	3(2)	3(2)	4(2)
**C(30)**	24(2)	34(2)	24(2)	4(2)	6(2)	11(2)
**C(31)**	32(2)	27(2)	24(2)	-1(2)	5(2)	4(2)
**C(32)**	28(2)	35(3)	19(2)	2(2)	0(2)	6(2)
**C(33)**	25(2)	28(2)	21(2)	1(2)	1(2)	7(2)
**C(34)**	22(2)	25(2)	21(2)	3(2)	3(2)	7(2)
**C(35)**	28(2)	27(2)	22(2)	4(2)	5(2)	2(2)
**C(36)**	41(3)	30(2)	21(2)	7(2)	10(2)	7(2)
**C(37)**	41(3)	22(2)	28(2)	3(2)	6(2)	8(2)
**C(38)**	32(2)	19(2)	25(2)	1(2)	5(2)	10(2)
**C(39)**	24(2)	25(2)	24(2)	4(2)	5(2)	9(2)
**O(1A)**	33(3)	36(3)	27(3)	3(2)	0(2)	3(2)
**C(1A)**	33(5)	33(4)	34(4)	0(3)	1(4)	1(4)
**C(2A)**	32(4)	38(5)	30(4)	3(3)	0(3)	5(3)
**C(3A)**	31(4)	32(6)	29(4)	3(4)	1(3)	2(4)
**C(4A)**	32(4)	41(5)	29(4)	4(3)	0(3)	5(3)
**O(1B)**	34(4)	38(5)	40(5)	10(4)	2(3)	4(3)
**C(1B)**	33(5)	39(6)	34(6)	8(4)	4(5)	6(4)
**C(2B)**	33(6)	37(6)	30(5)	4(4)	3(5)	1(5)
**C(3B)**	36(6)	37(6)	36(6)	6(4)	2(5)	1(5)
**C(4B)**	33(5)	27(7)	31(6)	2(5)	-1(4)	0(5)

3.3.2. Crystal structure determination of $\mathbf{Y_{pOMe}PCy_2}$•AuCl

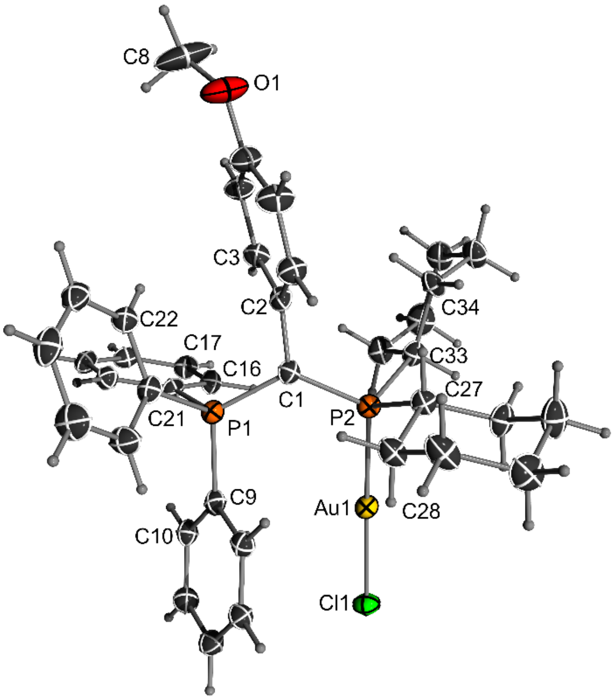

Figure 3.7: ORTEP of **$Y_{pOMe}PCy_2$•AuCl .(2•AuCl)** Ellipsoids are drawn at the 50% probability level.

Table 3.17: Atomic coordinates (x 10^4) and equivalent isotropic displacement parameters ($Å^2$x 10^3) for **$Y_{pOMe}PCy_2$•AuCl .(2•AuCl)**. U(eq) is defined as one third of the trace of the orthogonalized U^{ij} tensor.

	x	y	z	U(eq)
**Au(1)**	3699(1)	9787(1)	6168(1)	20(1)
**Cl(1)**	3459(1)	11160(1)	6258(1)	27(1)
**P(1)**	3263(1)	7605(1)	5628(1)	19(1)
**P(2)**	3941(1)	8463(1)	6108(1)	18(1)
**O(1)**	4005(1)	3955(2)	5898(2)	48(1)
**C(1)**	3675(1)	7569(2)	5911(2)	21(1)
**C(2)**	3798(1)	6640(2)	5994(2)	21(1)
**C(3)**	3733(1)	6112(2)	6929(2)	24(1)
**C(4)**	3806(1)	5209(2)	6950(3)	28(1)
**C(5)**	3944(1)	4832(2)	6012(3)	32(1)
**C(6)**	4032(1)	5361(2)	5101(3)	33(1)
**C(7)**	3954(1)	6245(2)	5091(3)	27(1)
**C(8)**	3863(2)	3379(2)	6678(4)	68(2)
**C(9)**	3099(1)	8656(2)	5148(2)	21(1)
**C(10)**	2841(1)	9045(2)	5690(2)	23(1)
**C(11)**	2705(1)	9822(2)	5258(3)	27(1)
**C(12)**	2821(1)	10210(2)	4282(3)	27(1)
**C(13)**	3075(1)	9827(2)	3735(3)	25(1)
**C(14)**	3214(1)	9057(2)	4168(2)	23(1)
**C(15)**	3030(1)	7312(2)	6864(2)	20(1)
**C(16)**	3138(1)	7642(2)	7940(2)	24(1)
**C(17)**	2973(1)	7453(2)	8916(2)	25(1)
**C(18)**	2698(1)	6920(2)	8822(2)	26(1)

**C(19)**	2587(1)	6594(2)	7760(3)	26(1)
**C(20)**	2750(1)	6787(2)	6778(2)	23(1)
**C(21)**	3138(1)	6800(2)	4514(2)	22(1)
**C(22)**	3173(1)	5900(2)	4727(3)	26(1)
**C(23)**	3105(1)	5289(2)	3853(3)	30(1)
**C(24)**	2996(1)	5568(2)	2768(3)	32(1)
**C(25)**	2955(1)	6457(2)	2555(3)	37(1)
**C(26)**	3028(1)	7070(2)	3422(3)	31(1)
**C(27)**	4238(1)	8423(2)	4971(2)	21(1)
**C(28)**	4064(1)	8568(2)	3790(2)	25(1)
**C(29)**	4303(1)	8534(2)	2831(2)	33(1)
**C(30)**	4575(1)	9191(3)	3036(3)	38(1)
**C(31)**	4753(1)	9032(3)	4203(3)	36(1)
**C(32)**	4518(1)	9075(2)	5176(2)	29(1)
**C(33)**	4204(1)	8316(2)	7433(2)	23(1)
**C(34)**	4442(1)	7527(2)	7451(2)	25(1)
**C(35)**	4670(1)	7560(2)	8531(3)	32(1)
**C(36)**	4479(1)	7571(2)	9621(3)	35(1)
**C(37)**	4241(1)	8338(2)	9594(3)	37(1)
**C(38)**	4011(1)	8316(2)	8517(2)	28(1)

Table 3.18: Anisotropic displacement parameters (Å^2 x 10^3) for **$Y_{pOMe}PCy_2$•AuCl (2•AuCl).** The anisotropic displacement factor exponent takes the form: $-2\pi^2$ [$h^2a^{*2}U^{11}$ + ... + 2 h k a·b·U^{12}].

	**U^{11}**	**U^{22}**	**U^{22}**	**U^{23}**	**U^{13}**	**U^{12}**
**Au(1)**	23(1)	19(1)	17(1)	-1(1)	3(1)	-1(1)
**Cl(1)**	33(1)	21(1)	26(1)	-2(1)	0(1)	3(1)
**P(1)**	21(1)	19(1)	17(1)	-2(1)	2(1)	0(1)
**P(2)**	21(1)	20(1)	14(1)	-1(1)	3(1)	0(1)
**O(1)**	73(2)	24(1)	49(2)	5(1)	21(1)	14(1)
**C(1)**	21(1)	21(1)	20(1)	-1(1)	3(1)	-1(1)
**C(2)**	21(1)	22(1)	21(1)	-4(1)	1(1)	0(1)
**C(3)**	27(1)	25(1)	21(1)	-2(1)	3(1)	3(1)
**C(4)**	35(2)	26(2)	24(2)	5(1)	1(1)	3(1)
**C(5)**	37(2)	24(2)	36(2)	-1(1)	6(1)	5(1)
**C(6)**	44(2)	31(1)	26(2)	-1(1)	12(1)	8(1)
**C(7)**	30(1)	27(1)	25(1)	-2(1)	7(1)	2(1)
**C(8)**	123(4)	22(2)	65(3)	8(2)	41(3)	15(2)
**C(9)**	22(1)	22(1)	20(1)	-3(1)	0(1)	-2(1)
**C(10)**	23(1)	24(1)	23(1)	-3(1)	2(1)	-3(1)
**C(11)**	24(2)	26(2)	31(2)	-5(1)	4(1)	1(1)
**C(12)**	30(2)	23(2)	29(2)	0(1)	-3(1)	0(1)
**C(13)**	26(2)	26(2)	24(2)	0(1)	-2(1)	-1(1)
**C(14)**	24(1)	25(1)	20(1)	-1(1)	0(1)	-2(1)
**C(15)**	21(1)	18(1)	19(1)	1(1)	4(1)	0(1)
**C(16)**	24(1)	24(1)	24(1)	-2(1)	2(1)	-2(1)
**C(17)**	30(1)	26(1)	21(1)	-4(1)	3(1)	2(1)
**C(18)**	31(1)	24(1)	23(1)	2(1)	8(1)	2(1)
**C(19)**	25(1)	24(1)	28(1)	2(1)	5(1)	-4(1)

**C(20)**	25(1)	22(1)	22(1)	-1(1)	0(1)	-1(1)
**C(21)**	22(1)	23(1)	21(1)	-4(1)	4(1)	-2(1)
**C(22)**	25(1)	26(1)	26(1)	1(1)	1(1)	-1(1)
**C(23)**	28(2)	23(1)	38(2)	-6(1)	4(1)	0(1)
**C(24)**	34(2)	33(2)	30(2)	-12(1)	4(1)	-7(1)
**C(25)**	50(2)	39(2)	21(1)	-8(1)	-5(1)	0(2)
**C(26)**	39(2)	27(1)	25(2)	-1(1)	-5(1)	2(1)
**C(27)**	23(1)	26(1)	15(1)	0(1)	2(1)	0(1)
**C(28)**	23(1)	34(2)	18(1)	-1(1)	3(1)	4(1)
**C(29)**	28(2)	53(2)	17(1)	2(1)	3(1)	7(1)
**C(30)**	37(2)	53(2)	24(2)	9(1)	12(1)	1(2)
**C(31)**	31(2)	51(2)	29(2)	1(1)	10(1)	-10(1)
**C(32)**	30(2)	35(2)	22(1)	0(1)	8(1)	-8(1)
**C(33)**	26(1)	26(1)	17(1)	1(1)	2(1)	-5(1)
**C(34)**	22(1)	33(1)	20(1)	2(1)	-1(1)	2(1)
**C(35)**	28(1)	44(2)	26(2)	9(1)	-4(1)	-4(1)
**C(36)**	36(2)	48(2)	21(1)	10(1)	-5(1)	-5(1)
**C(37)**	45(2)	49(2)	16(1)	-3(1)	0(1)	-8(2)
**C(38)**	31(1)	36(2)	17(1)	-3(1)	3(1)	-1(1)

3.3.3. Crystal structure determination of **Y$_{pCF3}$PCy$_2$•AuCl**

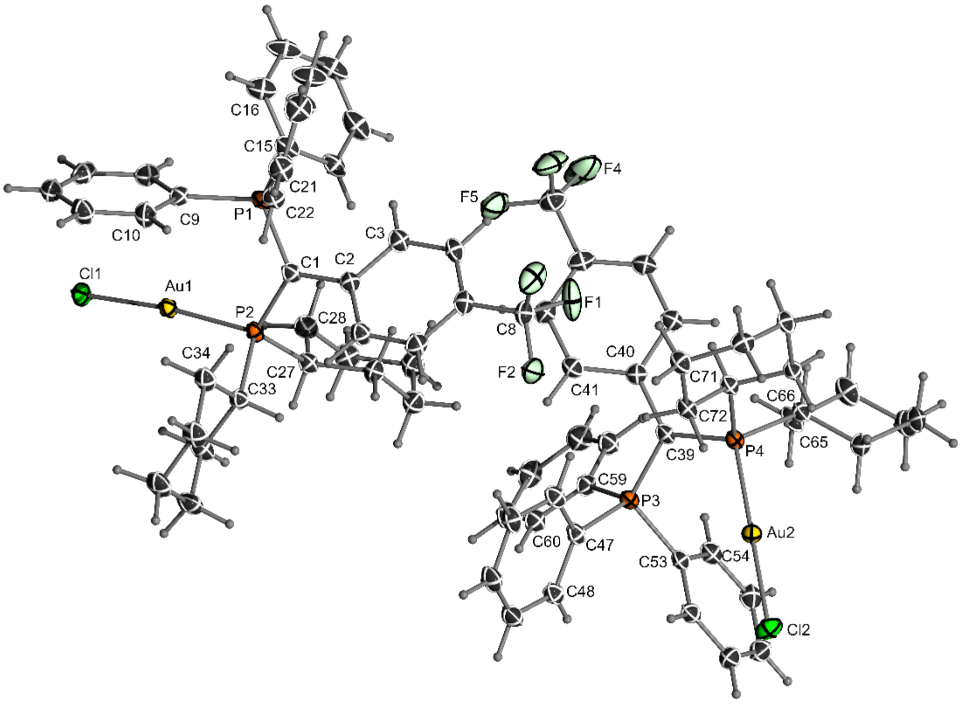

Figure 3.8: ORTEP of **Y$_{pCF3}$PCy$_2$•AuCl (3•AuCl)** Ellipsoids are drawn at the 50% probability level.

Table 3.19: Atomic coordinates (x 10^4) and equivalent isotropic displacement parameters ($Å^2$x 10^3) for **$Y_{pCF3}PCy_2$•AuCl (3•AuCl)**. U(eq) is defined as one third of the trace of the orthogonalized U^{ij} tensor.

	x	y	z	U(eq)
**Au(1)**	8167(1)	-776(1)	2978(1)	16(1)
**Au(2)**	4192(1)	6981(1)	1417(1)	17(1)
**Cl(1)**	7999(1)	-2093(1)	3540(1)	23(1)
**Cl(2)**	3058(1)	7392(1)	524(1)	25(1)
**P(1)**	10744(1)	768(1)	2594(1)	16(1)
**P(2)**	8064(1)	506(1)	2473(1)	16(1)
**P(3)**	3940(1)	5016(1)	2533(1)	15(1)
**P(4)**	5371(1)	6594(1)	2245(1)	15(1)
**F(1)**	9041(2)	4916(1)	1782(1)	43(1)
**F(2)**	8596(2)	4694(1)	779(1)	39(1)
**F(3)**	10498(2)	4820(1)	850(1)	38(1)
**F(4)**	8706(3)	4079(2)	4797(1)	60(1)
**F(5)**	8669(2)	3120(1)	4210(1)	44(1)
**F(6)**	9966(2)	4090(1)	3753(1)	45(1)
**C(1)**	9420(2)	1127(1)	2326(1)	17(1)
**C(2)**	9442(2)	1994(1)	2027(1)	18(1)
**C(3)**	9932(2)	2560(2)	2362(1)	20(1)
**C(4)**	9913(2)	3367(2)	2104(1)	21(1)
**C(5)**	9401(2)	3639(2)	1496(1)	20(1)
**C(6)**	8945(3)	3096(2)	1141(1)	22(1)
**C(7)**	8981(2)	2292(2)	1398(1)	20(1)
**C(8)**	9380(3)	4509(2)	1227(2)	23(1)
**C(9)**	11039(2)	-252(2)	2491(1)	18(1)
**C(10)**	11414(3)	-425(2)	1775(2)	25(1)
**C(11)**	11672(3)	-1189(2)	1672(2)	28(1)
**C(12)**	11539(3)	-1798(2)	2278(2)	26(1)
**C(13)**	11152(2)	-1638(2)	2983(2)	24(1)
**C(14)**	10912(2)	-865(2)	3095(1)	22(1)
**C(15)**	10867(2)	813(2)	3540(1)	20(1)
**C(16)**	11850(3)	469(2)	3834(1)	26(1)
**C(17)**	11935(3)	540(2)	4544(2)	31(1)
**C(18)**	11053(3)	955(2)	4968(2)	31(1)
**C(19)**	10076(3)	1289(2)	4687(2)	29(1)
**C(20)**	9975(3)	1214(2)	3973(1)	22(1)
**C(21)**	12090(2)	1338(1)	1992(1)	19(1)
**C(22)**	12167(3)	1476(2)	1229(1)	22(1)
**C(23)**	13247(3)	1809(2)	747(1)	24(1)
**C(24)**	14250(3)	2029(2)	1010(2)	28(1)
**C(25)**	14157(3)	1931(2)	1766(2)	33(1)
**C(26)**	13082(3)	1582(2)	2259(2)	28(1)
**C(27)**	6719(2)	901(1)	3070(1)	19(1)
**C(28)**	6649(3)	610(2)	3899(1)	22(1)
**C(29)**	5484(3)	895(2)	4372(1)	24(1)
**C(30)**	5390(3)	1792(2)	4205(2)	27(1)
**C(31)**	5431(3)	2065(2)	3387(2)	25(1)
**C(32)**	6624(3)	1804(2)	2911(1)	22(1)

**C(33)**	7546(2)	536(1)	1594(1)	19(1)
**C(34)**	8637(3)	378(2)	979(1)	22(1)
**C(35)**	8236(3)	444(2)	241(2)	27(1)
**C(36)**	7143(3)	-133(2)	309(2)	31(1)
**C(37)**	6055(3)	-15(2)	938(2)	30(1)
**C(38)**	6465(3)	-58(2)	1672(2)	26(1)
**C(38)**	6465(3)	-58(2)	1672(2)	26(1)
**C(39)**	5205(2)	5562(1)	2619(1)	17(1)
**C(40)**	6148(2)	5152(1)	2981(1)	17(1)
**C(41)**	6412(2)	4351(2)	2947(1)	20(1)
**C(42)**	7243(2)	3946(2)	3323(1)	22(1)
**C(43)**	7867(2)	4329(2)	3738(1)	23(1)
**C(44)**	7655(2)	5127(2)	3768(1)	22(1)
**C(45)**	6820(2)	5528(2)	3395(1)	20(1)
**C(46)**	8793(3)	3913(2)	4124(2)	29(1)
**C(47)**	4154(2)	4612(1)	1682(1)	18(1)
**C(48)**	3160(3)	4321(2)	1430(1)	21(1)
**C(49)**	3396(3)	4004(2)	786(2)	26(1)
**C(50)**	4611(3)	3960(2)	392(2)	26(1)
**C(51)**	5597(3)	4227(2)	641(1)	24(1)
**C(52)**	5370(2)	4563(2)	1284(1)	21(1)
**C(53)**	2491(2)	5541(1)	2626(1)	18(1)
**C(54)**	1880(3)	5663(2)	3334(1)	22(1)
**C(55)**	777(3)	6057(2)	3434(2)	25(1)
**C(56)**	248(3)	6327(2)	2835(2)	27(1)
**C(57)**	842(3)	6202(2)	2136(2)	26(1)
**C(58)**	1958(3)	5818(1)	2028(1)	21(1)
**C(59)**	3532(2)	4180(1)	3296(1)	19(1)
**C(60)**	2938(2)	3498(2)	3200(1)	22(1)
**C(61)**	2471(3)	2906(2)	3807(2)	25(1)
**C(62)**	2600(3)	2993(2)	4507(2)	27(1)
**C(63)**	3213(3)	3664(2)	4601(2)	28(1)
**C(64)**	3675(3)	4257(2)	4002(1)	23(1)
**C(65)**	5112(2)	7202(1)	2993(1)	19(1)
**C(66)**	4241(3)	6792(2)	3710(1)	25(1)
**C(67)**	4150(3)	7307(2)	4311(2)	30(1)
**C(68)**	3644(3)	8113(2)	4063(2)	32(1)
**C(69)**	4454(3)	8534(2)	3336(2)	28(1)
**C(70)**	4602(3)	8013(2)	2736(2)	22(1)
**C(71)**	7031(2)	6815(1)	1767(1)	17(1)
**C(72)**	7338(2)	6520(2)	1024(1)	20(1)
**C(73)**	8723(2)	6671(2)	643(1)	22(1)
**C(74)**	9138(2)	7541(2)	551(1)	22(1)
**C(75)**	8787(2)	7845(2)	1284(2)	21(1)
**C(76)**	7397(2)	7702(1)	1641(1)	18(1)

Table 3.20: Anisotropic displacement parameters (Å^2 x 10^3) for **Y$_{pCF3}$PCy$_2$•AuCl (3•AuCl).** The anisotropic displacement factor exponent takes the form: $-2\pi^2$ [$h^2a^{*2}U^{11}$ + ... + 2 h k a·b·U^{12}].

	U^{11}	U^{22}	U^{22}	U^{23}	U^{13}	U^{12}
**Au(1)**	19(1)	15(1)	16(1)	-2(1)	-5(1)	-1(1)
**Au(2)**	19(1)	17(1)	15(1)	-3(1)	-4(1)	1(1)
**Cl(1)**	28(1)	17(1)	25(1)	1(1)	-9(1)	-2(1)
**Cl(2)**	26(1)	32(1)	17(1)	-2(1)	-6(1)	7(1)
**P(1)**	18(1)	17(1)	12(1)	-2(1)	-4(1)	-1(1)
**P(2)**	19(1)	15(1)	14(1)	-2(1)	-4(1)	-1(1)
**P(3)**	17(1)	15(1)	15(1)	-2(1)	-4(1)	-2(1)
**P(4)**	17(1)	15(1)	15(1)	-3(1)	-4(1)	-1(1)
**F(1)**	73(1)	17(1)	32(1)	-5(1)	1(1)	2(1)
**F(2)**	44(1)	20(1)	58(1)	1(1)	-28(1)	2(1)
**F(3)**	28(1)	25(1)	48(1)	11(1)	3(1)	-3(1)
**F(4)**	78(2)	80(2)	39(1)	-25(1)	-38(1)	44(1)
**F(5)**	44(1)	30(1)	57(1)	7(1)	-23(1)	4(1)
**F(6)**	22(1)	48(1)	61(1)	6(1)	-14(1)	2(1)
**C(1)**	20(1)	17(1)	16(1)	-3(1)	-5(1)	-1(1)
**C(2)**	17(1)	17(1)	17(1)	-4(1)	-1(1)	-1(1)
**C(3)**	21(1)	19(1)	18(1)	-2(1)	-3(1)	-2(1)
**C(4)**	22(1)	18(1)	23(1)	-5(1)	-2(1)	-4(1)
**C(5)**	20(1)	17(1)	21(1)	-2(1)	0(1)	-1(1)
**C(6)**	24(1)	21(1)	21(1)	0(1)	-5(1)	1(1)
**C(7)**	25(1)	19(1)	18(1)	-3(1)	-6(1)	0(1)
**C(8)**	26(1)	20(1)	24(1)	-3(1)	-4(1)	1(1)
**C(9)**	17(1)	18(1)	19(1)	-3(1)	-4(1)	2(1)
**C(10)**	35(2)	21(1)	19(1)	-4(1)	-7(1)	3(1)
**C(11)**	38(2)	26(1)	24(1)	-8(1)	-10(1)	2(1)
**C(12)**	24(1)	20(1)	35(2)	-4(1)	-9(1)	2(1)
**C(13)**	20(1)	20(1)	29(1)	5(1)	-6(1)	0(1)
**C(14)**	22(1)	23(1)	19(1)	1(1)	-4(1)	-1(1)
**C(15)**	23(1)	22(1)	14(1)	0(1)	-3(1)	-5(1)
**C(16)**	23(1)	36(2)	18(1)	1(1)	-5(1)	-5(1)
**C(17)**	26(1)	45(2)	19(1)	4(1)	-9(1)	-12(1)
**C(18)**	36(2)	43(2)	13(1)	1(1)	-5(1)	-18(1)
**C(19)**	36(2)	31(1)	17(1)	-6(1)	1(1)	-13(1)
**C(20)**	25(1)	22(1)	18(1)	-2(1)	-3(1)	-8(1)
**C(21)**	22(1)	18(1)	16(1)	-2(1)	-2(1)	-1(1)
**C(22)**	27(1)	22(1)	18(1)	-2(1)	-7(1)	0(1)
**C(23)**	30(1)	25(1)	16(1)	1(1)	-2(1)	-2(1)
**C(24)**	28(1)	28(1)	21(1)	3(1)	2(1)	-3(1)
**C(25)**	25(1)	48(2)	25(1)	1(1)	-7(1)	-11(1)
**C(26)**	26(1)	40(2)	16(1)	-2(1)	-4(1)	-8(1)
**C(27)**	20(1)	19(1)	17(1)	-4(1)	-4(1)	-1(1)
**C(28)**	25(1)	23(1)	16(1)	-1(1)	-4(1)	0(1)
**C(29)**	26(1)	25(1)	20(1)	-2(1)	-2(1)	-1(1)
**C(30)**	29(1)	28(1)	23(1)	-6(1)	-4(1)	3(1)
**C(31)**	27(1)	25(1)	24(1)	-5(1)	-6(1)	4(1)
**C(32)**	23(1)	19(1)	22(1)	-2(1)	-5(1)	2(1)

**C(33)**	24(1)	17(1)	18(1)	-3(1)	-9(1)	0(1)
**C(34)**	31(1)	20(1)	17(1)	-4(1)	-7(1)	1(1)
**C(35)**	37(2)	29(1)	18(1)	-6(1)	-9(1)	1(1)
**C(36)**	46(2)	28(1)	24(1)	-9(1)	-17(1)	1(1)
**C(37)**	36(2)	32(2)	27(1)	-6(1)	-17(1)	-5(1)
**C(38)**	30(1)	26(1)	22(1)	-5(1)	-8(1)	-6(1)
**C(38)**	30(1)	26(1)	22(1)	-5(1)	-8(1)	-6(1)
**C(39)**	19(1)	16(1)	16(1)	-5(1)	-5(1)	-2(1)
**C(40)**	17(1)	18(1)	14(1)	-2(1)	-2(1)	0(1)
**C(41)**	19(1)	20(1)	21(1)	-4(1)	-4(1)	-1(1)
**C(42)**	21(1)	21(1)	21(1)	-2(1)	-3(1)	3(1)
**C(43)**	19(1)	29(1)	19(1)	2(1)	-4(1)	1(1)
**C(44)**	20(1)	26(1)	20(1)	-4(1)	-6(1)	-2(1)
**C(45)**	21(1)	21(1)	18(1)	-2(1)	-5(1)	-2(1)
**C(46)**	28(1)	33(2)	25(1)	-4(1)	-9(1)	4(1)
**C(47)**	24(1)	14(1)	16(1)	-1(1)	-6(1)	-2(1)
**C(48)**	24(1)	18(1)	21(1)	-3(1)	-6(1)	-1(1)
**C(49)**	31(1)	23(1)	26(1)	-7(1)	-12(1)	-3(1)
**C(50)**	35(2)	25(1)	20(1)	-9(1)	-7(1)	0(1)
**C(51)**	29(1)	22(1)	19(1)	-5(1)	-2(1)	-1(1)
**C(52)**	23(1)	18(1)	20(1)	-2(1)	-5(1)	-3(1)
**C(53)**	18(1)	14(1)	23(1)	-4(1)	-6(1)	-2(1)
**C(54)**	24(1)	21(1)	21(1)	-4(1)	-6(1)	-3(1)
**C(55)**	22(1)	22(1)	27(1)	-5(1)	0(1)	-2(1)
**C(56)**	21(1)	22(1)	38(2)	-4(1)	-8(1)	2(1)
**C(57)**	26(1)	21(1)	33(1)	-4(1)	-12(1)	2(1)
**C(58)**	24(1)	18(1)	23(1)	-3(1)	-10(1)	-3(1)
**C(59)**	18(1)	18(1)	19(1)	-2(1)	-3(1)	1(1)
**C(60)**	23(1)	20(1)	21(1)	-2(1)	-4(1)	-1(1)
**C(61)**	25(1)	18(1)	29(1)	-1(1)	-1(1)	-3(1)
**C(62)**	31(2)	21(1)	22(1)	5(1)	2(1)	0(1)
**C(63)**	32(2)	30(1)	19(1)	-2(1)	-5(1)	4(1)
**C(64)**	26(1)	21(1)	22(1)	-3(1)	-7(1)	-1(1)
**C(65)**	19(1)	18(1)	21(1)	-6(1)	-4(1)	-2(1)
**C(66)**	27(1)	27(1)	21(1)	-8(1)	-1(1)	-5(1)
**C(67)**	35(2)	33(2)	20(1)	-11(1)	-2(1)	-1(1)
**C(68)**	32(2)	41(2)	28(1)	-20(1)	-7(1)	8(1)
**C(69)**	32(2)	25(1)	32(1)	-15(1)	-11(1)	7(1)
**C(70)**	22(1)	21(1)	26(1)	-8(1)	-6(1)	3(1)
**C(71)**	19(1)	15(1)	17(1)	-3(1)	-4(1)	-2(1)
**C(72)**	22(1)	20(1)	19(1)	-5(1)	-4(1)	-4(1)
**C(73)**	22(1)	24(1)	18(1)	-6(1)	-2(1)	-1(1)
**C(74)**	18(1)	23(1)	21(1)	0(1)	-1(1)	-2(1)
**C(75)**	22(1)	15(1)	27(1)	-2(1)	-7(1)	-4(1)
**C(76)**	21(1)	14(1)	21(1)	-4(1)	-5(1)	-2(1)

3.3.4. Crystal structure determination of **$Y_{oTol}PCy_2$•AuCl**

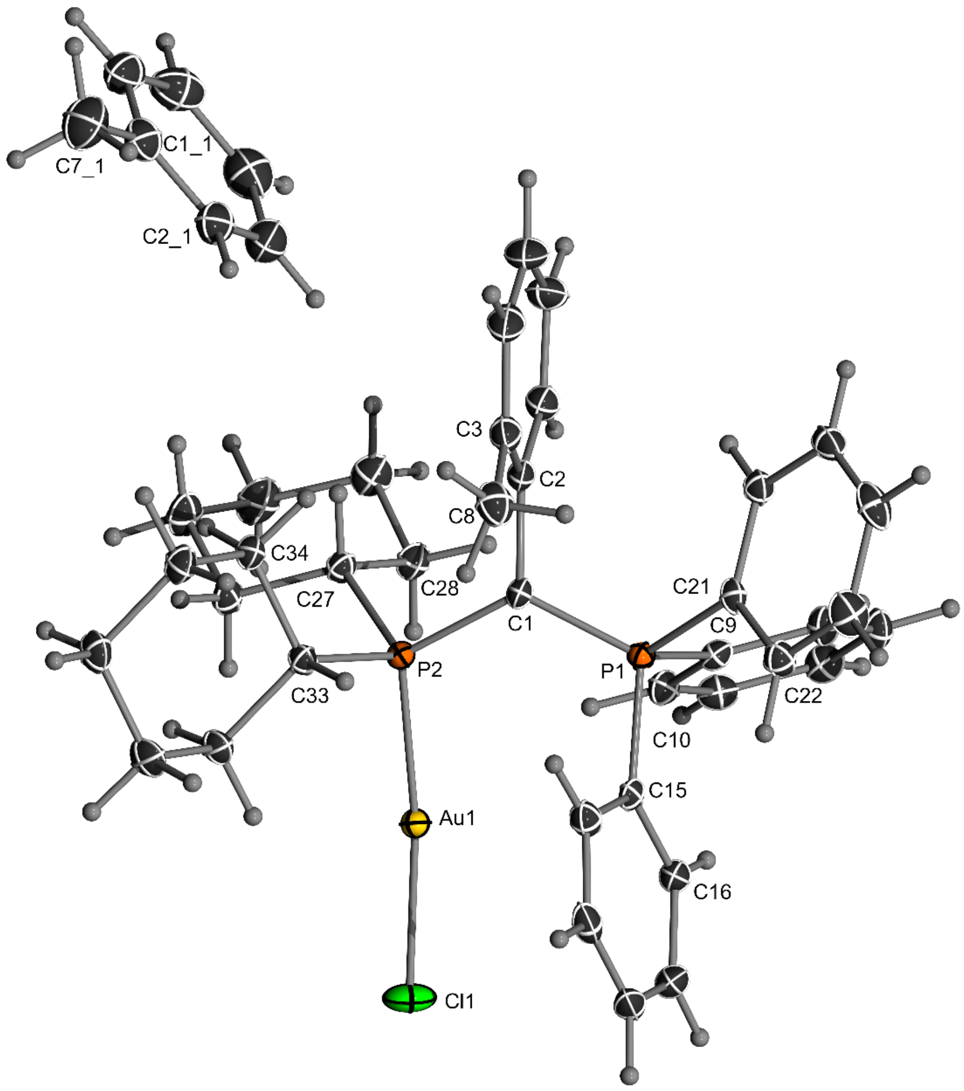

Figure 3.9:ORTEP of **$Y_{oTol}PCy_2$•AuCl (4•AuCl)** Ellipsoids are drawn at the 50% probability level.

Table 3.21: Atomic coordinates (x 10^4) and equivalent isotropic displacement parameters ($Å^2$x 10^3) for **$Y_{oTol}PCy_2$•AuCl (4•AuCl)**. U(eq) is defined as one third of the trace of the orthogonalized U^{ij} tensor.

	x	y	z	U(eq)
**Au(1)**	6256(1)	8156(1)	5968(1)	14(1)
**Cl(1)**	5581(1)	8891(1)	6827(1)	27(1)
**P(1)**	8729(1)	8404(1)	4806(1)	12(1)
**P(2)**	6696(1)	7387(1)	5107(1)	12(1)
**C(1)**	7908(2)	7629(1)	4629(1)	13(1)
**C(2)**	8407(2)	7084(2)	4140(1)	18(1)
**C(3)**	8041(2)	7064(2)	3405(2)	22(1)
**C(4)**	8530(3)	6530(2)	2992(2)	30(1)
**C(5)**	9401(3)	6055(2)	3271(2)	32(1)
**C(6)**	9813(3)	6092(2)	3989(2)	28(1)
**C(7)**	9305(3)	6594(2)	4419(2)	22(1)
**C(9)**	9942(2)	8383(1)	5534(1)	15(1)

**C(8)**	7199(3)	7613(2)	3046(2)	26(1)
**C(10)**	9684(2)	8127(1)	6197(1)	18(1)
**C(11)**	10557(3)	8124(2)	6772(2)	20(1)
**C(12)**	11702(2)	8367(2)	6695(2)	21(1)
**C(13)**	11975(2)	8615(2)	6036(2)	21(1)
**C(14)**	11095(2)	8628(2)	5456(1)	18(1)
**C(15)**	7810(2)	9168(1)	5006(1)	15(1)
**C(16)**	8067(2)	9600(1)	5608(1)	18(1)
**C(17)**	7374(3)	10218(2)	5704(2)	24(1)
**C(18)**	6462(3)	10413(2)	5191(2)	26(1)
**C(19)**	6209(2)	9987(2)	4582(2)	24(1)
**C(20)**	6861(2)	9359(2)	4496(2)	19(1)
**C(21)**	9467(2)	8684(1)	4032(1)	14(1)
**C(22)**	9262(2)	9374(2)	3734(1)	19(1)
**C(23)**	9809(3)	9582(2)	3142(2)	25(1)
**C(24)**	10589(2)	9113(2)	2842(1)	24(1)
**C(25)**	10813(2)	8430(2)	3141(1)	20(1)
**C(26)**	10259(2)	8212(1)	3731(1)	17(1)
**C(27)**	7004(2)	6461(1)	5468(1)	15(1)
**C(28)**	8102(2)	6465(1)	6026(1)	18(1)
**C(29)**	8464(3)	5684(2)	6235(2)	24(1)
**C(30)**	7442(3)	5297(2)	6556(2)	28(1)
**C(31)**	6301(2)	5315(2)	6037(2)	24(1)
**C(32)**	5961(2)	6094(1)	5795(2)	19(1)
**C(33)**	5311(2)	7307(1)	4477(1)	15(1)
**C(34)**	5233(2)	6628(1)	3993(1)	17(1)
**C(35)**	4120(2)	6664(2)	3453(2)	21(1)
**C(36)**	2977(2)	6755(2)	3824(2)	23(1)
**C(37)**	3059(2)	7424(2)	4302(2)	22(1)
**C(38)**	4160(2)	7394(2)	4846(1)	18(1)
**C11**	6365(3)	4051(2)	2925(2)	28(1)
**C21**	6665(3)	4725(2)	3226(2)	28(1)
**C31**	7451(3)	4781(2)	3838(2)	34(1)
**C41**	7968(3)	4159(2)	4155(2)	38(1)
**C51**	7683(3)	3482(2)	3856(2)	37(1)
**C61**	6880(3)	3428(2)	3253(2)	31(1)
**C71**	5486(3)	3997(2)	2267(2)	40(1)

Table 3.22: Anisotropic displacement parameters ($Å^2$ x 10^3) for **$Y_{oTol}PCy_2$•AuCl (4•AuCl).** The anisotropic displacement factor exponent takes the form: $–2\pi^2$ [$h^2a^{*2}U^{11}$ + ... + 2 h k $a^*b^*U^{12}$].

	U^{11}	U^{22}	U^{22}	U^{23}	U^{13}	U^{12}
**Au(1)**	15(1)	13(1)	15(1)	-1(1)	3(1)	1(1)
**Cl(1)**	37(1)	25(1)	21(1)	-6(1)	7(1)	10(1)
**P(1)**	12(1)	11(1)	14(1)	0(1)	1(1)	0(1)
**P(2)**	12(1)	11(1)	14(1)	0(1)	2(1)	0(1)
**C(1)**	13(1)	11(1)	16(1)	-1(1)	2(1)	-3(1)
**C(2)**	16(1)	17(1)	21(1)	-6(1)	6(1)	-6(1)
**C(3)**	20(1)	24(1)	21(1)	-4(1)	6(1)	-6(1)

**C(4)**	32(2)	32(2)	27(2)	-12(1)	13(1)	-10(1)
**C(5)**	35(2)	28(2)	36(2)	-13(1)	15(1)	-2(1)
**C(6)**	28(1)	21(1)	36(2)	-2(1)	12(1)	5(1)
**C(7)**	23(1)	19(1)	24(1)	-3(1)	6(1)	-3(1)
**C(9)**	17(1)	11(1)	16(1)	-1(1)	0(1)	0(1)
**C(8)**	25(1)	32(2)	22(1)	1(1)	4(1)	-4(1)
**C(10)**	18(1)	16(1)	18(1)	2(1)	3(1)	-1(1)
**C(11)**	25(1)	19(1)	16(1)	1(1)	1(1)	3(1)
**C(12)**	21(1)	20(1)	20(1)	0(1)	-6(1)	2(1)
**C(13)**	14(1)	22(1)	24(1)	1(1)	-2(1)	-3(1)
**C(14)**	16(1)	18(1)	18(1)	3(1)	0(1)	-2(1)
**C(15)**	15(1)	10(1)	22(1)	3(1)	4(1)	-2(1)
**C(16)**	17(1)	15(1)	24(1)	-2(1)	5(1)	-2(1)
**C(17)**	24(1)	17(1)	34(2)	-7(1)	10(1)	-4(1)
**C(18)**	19(1)	13(1)	48(2)	1(1)	12(1)	1(1)
**C(19)**	14(1)	20(1)	38(2)	10(1)	6(1)	1(1)
**C(20)**	16(1)	17(1)	25(1)	4(1)	3(1)	0(1)
**C(21)**	12(1)	18(1)	14(1)	0(1)	0(1)	-3(1)
**C(22)**	18(1)	18(1)	21(1)	3(1)	3(1)	0(1)
**C(23)**	25(1)	27(2)	23(1)	10(1)	3(1)	1(1)
**C(24)**	19(1)	38(2)	17(1)	6(1)	4(1)	-3(1)
**C(25)**	14(1)	27(1)	20(1)	-4(1)	2(1)	-1(1)
**C(26)**	14(1)	17(1)	20(1)	0(1)	1(1)	-1(1)
**C(27)**	15(1)	12(1)	18(1)	0(1)	0(1)	0(1)
**C(28)**	19(1)	16(1)	19(1)	2(1)	-1(1)	-1(1)
**C(29)**	24(1)	21(1)	27(1)	4(1)	-5(1)	3(1)
**C(30)**	33(2)	19(1)	31(2)	10(1)	-3(1)	2(1)
**C(31)**	25(1)	17(1)	31(2)	7(1)	1(1)	-2(1)
**C(32)**	18(1)	14(1)	26(1)	4(1)	4(1)	-1(1)
**C(33)**	12(1)	14(1)	19(1)	0(1)	0(1)	0(1)
**C(34)**	15(1)	16(1)	19(1)	-2(1)	1(1)	-2(1)
**C(35)**	18(1)	23(1)	22(1)	-4(1)	-1(1)	-4(1)
**C(36)**	14(1)	26(1)	28(1)	-1(1)	-2(1)	-3(1)
**C(37)**	12(1)	26(1)	28(1)	-1(1)	-3(1)	4(1)
**C(38)**	13(1)	20(1)	22(1)	-2(1)	0(1)	1(1)
**C11**	27(1)	28(2)	28(1)	1(1)	8(1)	-2(1)
**C21**	29(1)	24(2)	32(2)	3(1)	9(1)	-1(1)
**C31**	34(2)	31(2)	38(2)	-6(1)	8(1)	-6(1)
**C41**	34(2)	47(2)	33(2)	1(2)	3(1)	-1(2)
**C51**	42(2)	35(2)	36(2)	6(1)	10(1)	9(2)
**C61**	36(2)	23(2)	37(2)	0(1)	12(1)	-1(1)
**C71**	42(2)	40(2)	37(2)	-3(2)	-1(2)	-6(2)

3.3.5. Crystal structure determination of **$Y_{Mes}PCy_2$•AuCl**

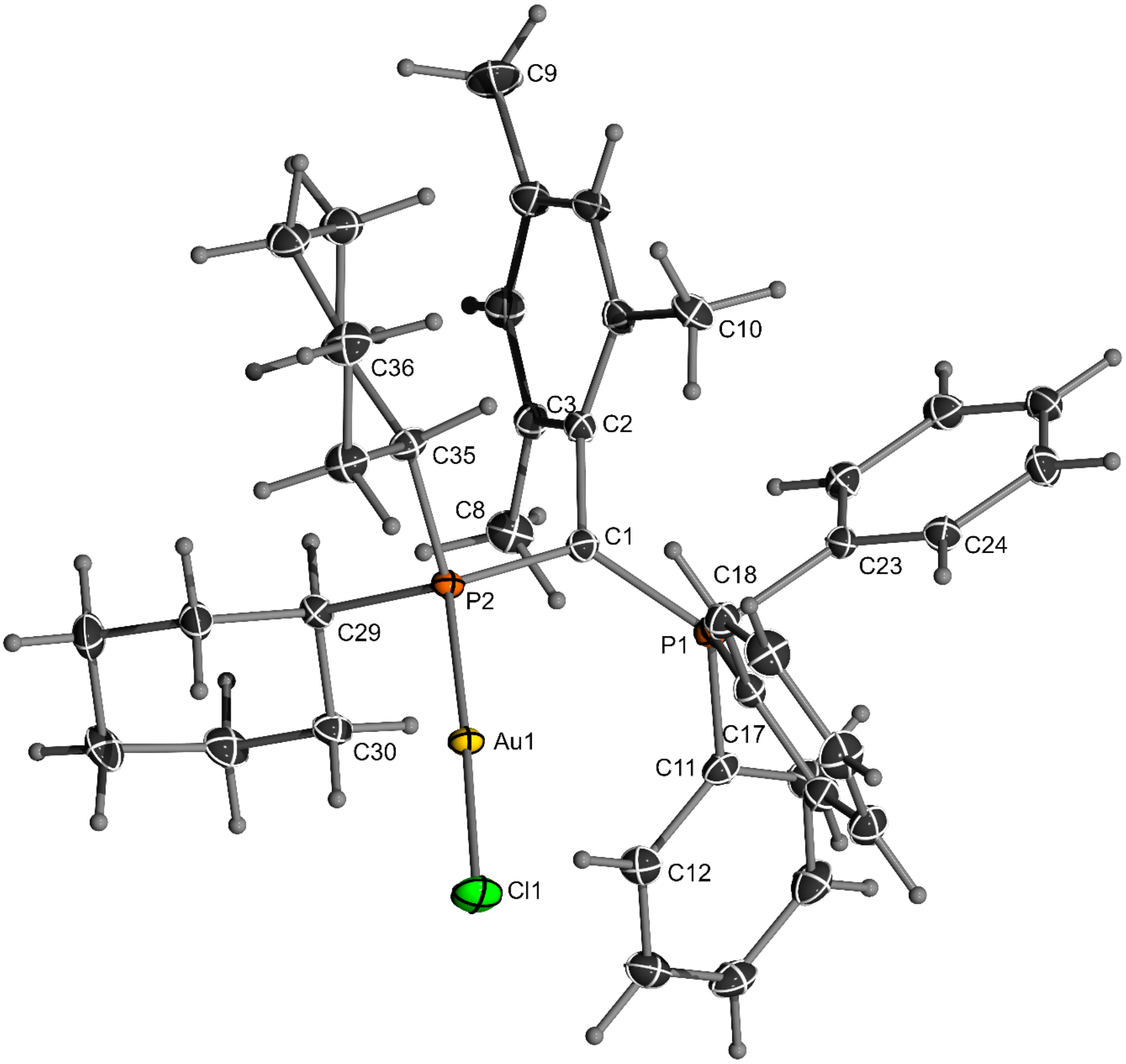

Figure 3.10: ORTEP of **$Y_{Mes}PCy_2$•AuCl (5•AuCl)** Ellipsoids are drawn at the 50% probability level.

Table 3.23: Atomic coordinates (x 10^4) and equivalent isotropic displacement parameters ($Å^2$x 10^3) for **$Y_{Mes}PCy_2$•AuCl (5•AuCl)**. U(eq) is defined as one third of the trace of the orthogonalized U^{ij} tensor.

	**x**	**y**	**z**	**U(eq)**
**Au(1)**	6421(1)	8696(1)	3112(1)	13(1)
**Cl(1)**	6852(1)	9863(1)	3813(1)	22(1)
**P(1)**	3562(1)	7733(1)	2368(1)	12(1)
**P(2)**	6117(1)	7519(1)	2482(1)	11(1)
**C(1)**	4668(2)	7164(1)	2237(1)	14(1)
**C(2)**	4474(2)	6311(1)	1954(1)	14(1)
**C(3)**	4327(2)	6118(1)	1161(1)	16(1)
**C(4)**	4404(2)	5327(1)	941(1)	19(1)

**C(5)**	4582(2)	4704(1)	1476(1)	21(1)
**C(6)**	4592(2)	4886(1)	2233(1)	19(1)
**C(7)**	4516(2)	5672(1)	2478(1)	16(1)
**C(8)**	4033(2)	6738(1)	522(1)	20(1)
**C(9)**	4740(3)	3862(1)	1232(2)	32(1)
**C(10)**	4443(2)	5784(1)	3298(1)	20(1)
**C(11)**	2896(2)	8484(1)	1628(1)	15(1)
**C(12)**	3561(2)	9135(1)	1521(1)	18(1)
**C(13)**	3077(2)	9718(1)	974(1)	22(1)
**C(14)**	1931(2)	9652(1)	518(1)	22(1)
**C(15)**	1268(2)	9004(1)	612(1)	22(1)
**C(16)**	1748(2)	8427(1)	1168(1)	19(1)
**C(17)**	3888(2)	8293(1)	3274(1)	14(1)
**C(18)**	4657(2)	7978(1)	3951(1)	17(1)
**C(19)**	4776(2)	8332(1)	4667(1)	22(1)
**C(20)**	4128(2)	9004(1)	4721(1)	23(1)
**C(21)**	3373(2)	9323(1)	4058(1)	21(1)
**C(22)**	3253(2)	8974(1)	3333(1)	17(1)
**C(23)**	2360(2)	7091(1)	2397(1)	14(1)
**C(24)**	1889(2)	7136(1)	3024(1)	17(1)
**C(25)**	964(2)	6651(1)	3047(1)	20(1)
**C(26)**	501(2)	6113(1)	2448(1)	20(1)
**C(27)**	965(2)	6066(1)	1822(1)	18(1)
**C(28)**	1880(2)	6554(1)	1792(1)	16(1)
**C(29)**	6695(2)	7565(1)	1631(1)	14(1)
**C(30)**	6110(2)	8226(1)	1062(1)	16(1)
**C(31)**	6555(2)	8232(1)	340(1)	22(1)
**C(32)**	7871(2)	8327(1)	562(1)	25(1)
**C(33)**	8474(2)	7705(2)	1162(2)	26(1)
**C(34)**	8016(2)	7716(1)	1876(1)	19(1)
**C(35)**	6970(2)	6723(1)	3107(1)	15(1)
**C(36)**	7289(2)	5998(1)	2681(1)	17(1)
**C(37)**	7710(2)	5315(1)	3250(1)	22(1)
**C(38)**	8733(2)	5568(1)	3938(1)	22(1)
**C(39)**	8463(2)	6329(1)	4330(1)	23(1)
**C(40)**	8028(2)	7003(1)	3749(1)	20(1)
**Au(1)**	6421(1)	8696(1)	3112(1)	13(1)
**Cl(1)**	6852(1)	9863(1)	3813(1)	22(1)

Table 3.24: Anisotropic displacement parameters (Å^2 x 10^3) for **Y$_{Mes}$PCy$_2$•AuCl (5•AuCl).** The anisotropic displacement factor exponent takes the form: $-2\pi^2$ [$h^2a^{*2}U^{11}$ + ... + 2 h k a·b·U^{12}].

	**U^{11}**	**U^{22}**	**U^{22}**	**U^{23}**	**U^{13}**	**U^{12}**
**Au(1)**	14(1)	10(1)	13(1)	-1(1)	4(1)	0(1)
**Cl(1)**	30(1)	14(1)	23(1)	-7(1)	7(1)	-2(1)
**P(1)**	12(1)	11(1)	12(1)	-1(1)	3(1)	-1(1)
**P(2)**	13(1)	9(1)	11(1)	0(1)	4(1)	0(1)
**C(1)**	15(1)	12(1)	14(1)	0(1)	5(1)	1(1)
**C(2)**	13(1)	13(1)	16(1)	-1(1)	5(1)	-1(1)

**C(3)**	14(1)	16(1)	17(1)	-2(1)	5(1)	-1(1)
**C(4)**	19(1)	19(1)	20(1)	-6(1)	6(1)	-4(1)
**C(5)**	19(1)	14(1)	30(1)	-4(1)	8(1)	-2(1)
**C(6)**	18(1)	13(1)	27(1)	3(1)	7(1)	-1(1)
**C(7)**	13(1)	14(1)	19(1)	1(1)	4(1)	-3(1)
**C(8)**	27(1)	20(1)	14(1)	-2(1)	4(1)	-2(1)
**C(9)**	40(2)	15(1)	43(1)	-8(1)	16(1)	-1(1)
**C(10)**	24(1)	19(1)	18(1)	4(1)	7(1)	-3(1)
**C(11)**	16(1)	14(1)	14(1)	-2(1)	5(1)	2(1)
**C(12)**	18(1)	17(1)	17(1)	-1(1)	3(1)	-1(1)
**C(13)**	29(1)	16(1)	22(1)	0(1)	9(1)	-2(1)
**C(14)**	28(1)	20(1)	18(1)	4(1)	6(1)	8(1)
**C(15)**	18(1)	26(1)	21(1)	3(1)	2(1)	6(1)
**C(16)**	18(1)	18(1)	21(1)	1(1)	5(1)	0(1)
**C(17)**	15(1)	14(1)	14(1)	-2(1)	6(1)	-3(1)
**C(18)**	17(1)	17(1)	17(1)	-1(1)	6(1)	0(1)
**C(19)**	25(1)	27(1)	14(1)	0(1)	4(1)	-1(1)
**C(20)**	28(1)	26(1)	18(1)	-9(1)	9(1)	-2(1)
**C(21)**	21(1)	19(1)	24(1)	-6(1)	9(1)	0(1)
**C(22)**	16(1)	17(1)	19(1)	-1(1)	5(1)	0(1)
**C(23)**	11(1)	12(1)	17(1)	2(1)	4(1)	0(1)
**C(24)**	17(1)	15(1)	19(1)	-2(1)	5(1)	-1(1)
**C(25)**	21(1)	21(1)	22(1)	1(1)	11(1)	-2(1)
**C(26)**	16(1)	15(1)	28(1)	2(1)	5(1)	-1(1)
**C(27)**	16(1)	15(1)	22(1)	-2(1)	1(1)	-1(1)
**C(28)**	16(1)	17(1)	16(1)	0(1)	3(1)	1(1)
**C(29)**	14(1)	13(1)	14(1)	1(1)	5(1)	-1(1)
**C(30)**	21(1)	14(1)	14(1)	2(1)	6(1)	0(1)
**C(31)**	30(1)	22(1)	16(1)	4(1)	9(1)	0(1)
**C(32)**	30(1)	26(1)	24(1)	6(1)	17(1)	-2(1)
**C(33)**	21(1)	31(1)	33(1)	7(1)	17(1)	3(1)
**C(34)**	14(1)	22(1)	22(1)	5(1)	7(1)	0(1)
**C(35)**	19(1)	14(1)	13(1)	1(1)	4(1)	4(1)
**C(36)**	18(1)	12(1)	20(1)	-1(1)	5(1)	3(1)
**C(37)**	24(1)	17(1)	25(1)	2(1)	6(1)	2(1)
**C(38)**	20(1)	19(1)	25(1)	6(1)	3(1)	3(1)
**C(39)**	20(1)	25(1)	19(1)	2(1)	-1(1)	2(1)
**C(40)**	19(1)	19(1)	19(1)	-2(1)	2(1)	0(1)
**Au(1)**	14(1)	10(1)	13(1)	-1(1)	4(1)	0(1)
**Cl(1)**	30(1)	14(1)	23(1)	-7(1)	7(1)	-2(1)

4. Computational Studies

4.1. General information

All computational studies were carried out without semmetry restrictions. If it was not possible to obtain starting coordinates from crystal structures *GaussView 6.0*[27] was used. Calculations were performed with the *Gaussian16 Revision C.01*[28] program package using Density-Functional Theory (DFT).[29] Energy optimizations were carried out with the PW6B95D3[30] functional with Grimmes D3 dispersion correction with Becke-Johnson damping.[31] The def2svp[32] basis set together with the MWB60 ECP[33] as implemented in *Gaussian* for Gold were used. To determine the nature of the structure harmonic vibrational frequency analysis were performed on the same level of theory.[34] No imaginary frequencies were observed. Single point energies were also calculated with the PW6B95D3[30] functional and the def2tzvp[32] basis set together with the MWB60 ECP[33] as implemented in *Gaussian* for Gold.
NBO analyses[35] were performed with the NBO7[36] program package, quantum theorie of atoms in molecules (QTAIM) analyses[37] with Multiwfn 3.7(dev).[38]

4.2. Energies of the structures

Table 4.1. Energies of the calculated structures

	E(SCF)	Corr(H)	Corr(G)
**1•AuCl**	-2717.50760938	0.745193	0.633164
**2•AuCl**	-2832.20697585	0.780535	0.659855
**3•AuCl**	-3055.06939653	0.753695	0.630856
**4•AuCl**	-2756.88652887	0.773994	0.657008
**5•AuCl**	-2835.64667582	0.832989	0.710372
**[1•AuNCMe]$^+$**	-2389.69194590	0.794523	0.673080
**[2•AuNCMe]$^+$**	-2504.39591860	0.830178	0.700257
**[3•AuNCMe]$^+$**	-2727.25237835	0.802935	0.670785
**[4•AuNCMe]$^+$**	-2429.07131589	0.823478	0.695022
**[5•AuNCMe]$^+$**	-2507.84207199	0.882124	0.749889

4.3. Natural charges and BCP analysis

Table 4.2. Natural charges at Au, the electron density at the bond critical points of the Au–Ph-Interaction, and their ellipticity.

	Natural Charge at Au	Bond critical point analysis *electron density p*
**1•AuCl**	0.28188	0.01378
**2•AuCl**	0.26238	0.01371
**3•AuCl**	0.28082	0.01126
**4•AuCl**	0.29036	0.01527
**5•AuCl**	0.28482	0.01529
**[1•AuNCMe]$^+$**	0.36107	0.01618
**[2•AuNCMe]$^+$**	0.34916	0.01483
**[3•AuNCMe]$^+$**	0.35522	0.01176
**[4•AuNCMe]$^+$**	0.35910	0.01475
**[5•AuNCMe]$^+$**	0.36123	0.01695

4.4. Calculated Au–C distances

Table 4.3: Selected Au–C-Distances in Å.

	Au–^{ipso}C	Au–^{ortho}C	Sum (Au-C distances)
**1•AuCl**	3.23378	3.24389	6.47767
**2•AuCl**	3.19467	3.29720	6.49187
**3•AuCl**	3.36099	3.36903	6.73002
**4•AuCl**	3.20700	3.13049	6.33749
**5•AuCl**	3.24099	3.10633	6.34732
**[1•AuNCMe]$^+$**	3.12357	3.15290	6.27647
**[2•AuNCMe]$^+$**	3.15457	3.21119	6.36576
**[3•AuNCMe]$^+$**	3.34987	3.29529	6.64516
**[4•AuNCMe]$^+$**	3.13810	3.35415	6.49225
**[5•AuNCMe]$^+$**	3.12686	3.08247	6.20933

4.5. Coordinates of the energy-optimized structures

4.5.1. 1•AuCl

E = -2717.50760938
Au -1.703843 -1.351576 0.522718
Cl -2.204272 -3.571235 1.054351
P 1.596798 -0.535354 -0.293324
C 0.588720 0.839061 -0.598991
P -1.083916 0.775150 -0.003195
C 1.188936 2.170707 -0.860095
C 0.707577 2.971358 -1.909444
H -0.056236 2.568352 -2.569558
C 1.200988 4.250397 -2.135211
H 0.797039 4.844584 -2.953276
C 3.299003 0.397886 -2.281852
H 2.400551 0.827546 -2.717178
C 2.735760 3.977091 -0.314231
H 3.536713 4.358305 0.316934
C 2.218450 4.764089 -1.337273
H 2.609154 5.763666 -1.516340
C 3.226373 -0.271754 -1.056828
C 2.221077 2.708767 -0.076825
H 2.612661 2.123062 0.750364
C 4.515974 0.516452 -2.938626
H 4.565072 1.047768 -3.886766
C 5.666178 -0.041343 -2.388597
H 6.619430 0.055440 -2.904672
C 1.916305 -0.974543 1.456700
C 4.377676 -0.851806 -0.520623
H 4.328797 -1.404325 0.414896
C 5.593829 -0.731049 -1.184205
H 6.487061 -1.181830 -0.756594
C 2.803142 -0.190728 2.204516
H 3.433427 0.544505 1.707511
C 2.896078 -0.344422 3.580741
H 3.589488 0.273926 4.147340
C 2.100659 -1.284001 4.231782
H 2.163323 -1.396107 5.312480
C 1.235736 -2.084552 3.495662
H 0.614335 -2.828751 3.989163
C 1.145164 -1.937922 2.114825
H 0.453873 -2.574707 1.566133
C 1.017229 -2.074625 -1.067981
C 1.564024 -3.303419 -0.692771
H 2.315637 -3.354762 0.092793
C 1.131959 -4.469085 -1.310619
H 1.541890 -5.427498 -0.999732
C 0.170463 -4.412968 -2.314390
H -0.178583 -5.330649 -2.783136
C -0.341917 -3.186249 -2.719625
H -1.089220 -3.137530 -3.508746
C 0.083494 -2.018229 -2.100981
H -0.317409 -1.053019 -2.393103
C -1.267024 1.880446 1.481996

H -2.251067 1.606719 1.894260
C -0.207383 1.515108 2.517257
H -0.244998 0.439170 2.734273
H 0.781669 1.711974 2.082221
C -0.359180 2.326584 3.795171
H -1.307002 2.059010 4.286816
H 0.441555 2.059964 4.497905
C -0.346153 3.819728 3.501928
H -0.480887 4.397249 4.425823
H 0.638772 4.099244 3.096611
C -1.420111 4.181880 2.486080
H -1.393786 5.256362 2.262574
H -2.411100 3.977144 2.920238
C -1.262927 3.383174 1.196290
H -2.067982 3.647948 0.499277
H -0.320455 3.666001 0.709297
C -2.260312 1.502453 -1.232629
H -1.945969 2.543723 -1.397716
C -3.682618 1.503498 -0.668203
H -3.959943 0.466633 -0.420743
H -3.729097 2.069563 0.270025
C -4.677349 2.078963 -1.669086
H -4.447727 3.142951 -1.834417
H -5.690417 2.041522 -1.248525
C -4.623462 1.341453 -2.998859
H -4.953254 0.302375 -2.848267
H -5.322657 1.790244 -3.716102
C -3.209764 1.337448 -3.562191
H -2.912526 2.368978 -3.807494
H -3.169474 0.770409 -4.501295
C -2.218893 0.756574 -2.562282
H -1.200998 0.776023 -2.973512
H -2.462807 -0.301209 -2.373845

4.5.2.2•AuCl

E = -2832.20697585
Au 2.546823 0.441297 -0.551888
P 0.492069 1.194841 0.091433
Cl 4.645606 -0.248218 -1.287728
P -0.494730 -1.737531 0.184245
C -0.746110 -0.043625 0.324775
C 1.204299 -2.251916 0.520569
C -0.885485 -2.447695 -1.453832
C -1.565244 -2.648933 1.351023
C -0.053270 2.434387 -1.193392
C 0.585587 2.244561 1.619187
O -6.082776 1.819281 0.670827
C -4.834565 1.329316 0.528905
C -6.961529 1.738014 -0.414576
C -2.168422 0.389289 0.408026
C -3.042823 0.258119 -0.676778

C -2.687279 0.977847 1.572650
C -4.358734 0.710485 -0.628991
C -3.986056 1.451404 1.633858
C 1.796405 -1.830793 1.716591
C 1.932339 -3.041695 -0.370394
C 3.249580 -3.376637 -0.079807
C 3.840834 -2.937152 1.097269
C 3.109100 -2.174711 2.002744
C -0.297720 -1.812824 -2.552160
C -1.706117 -3.557209 -1.654706
C -0.534430 -2.276780 -3.838418
C -1.372004 -3.372404 -4.037148
C -1.953261 -4.011014 -2.947837
C -1.010927 -3.434755 2.365043
C -2.959785 -2.548877 1.257421
C -3.773563 -3.198025 2.176846
C -3.212554 -3.962216 3.194700
C -1.831310 -4.084268 3.281310
C 0.953309 1.414016 2.842823
C 1.568782 3.401740 1.443417
C 1.031794 2.268661 4.100336
C 2.016848 3.415201 3.924279
C 1.662350 4.252833 2.704600
C -0.280087 1.743479 -2.534646
C -1.219499 3.348449 -0.808369
C -1.546575 4.325468 -1.932139
C -1.834055 3.593737 -3.234436
C -0.647043 2.730450 -3.634401
H -2.683669 -0.213544 -1.590323
H -4.996406 0.587542 -1.499825
H -4.379044 1.915972 2.535208
H -2.045987 1.057869 2.448213
H -7.160436 0.694378 -0.701980
H -6.573489 2.274515 -1.293573
H -7.898825 2.203558 -0.098718
H 1.485799 -3.375256 -1.302855
H 3.824302 -3.961618 -0.793451
H 4.880805 -3.178138 1.304828
H 3.569567 -1.827693 2.925280
H 1.224554 -1.220729 2.409392
H 0.360993 -0.960875 -2.384782
H -0.063842 -1.783442 -4.686496
H -1.566623 -3.732196 -5.045715
H -2.601143 -4.871722 -3.102162
H -2.152139 -4.070410 -0.805987
H -3.417247 -1.968785 0.461195
H -4.854444 -3.104664 2.093429
H -3.852729 -4.468761 3.914432
H -1.383026 -4.692004 4.064749
H 0.066757 -3.549819 2.441737
H -0.425029 2.654782 1.770005
H 1.931331 0.940903 2.660207
H 0.223890 0.602820 2.970457
H 0.033630 2.677061 4.322785

H 1.312079 1.646642 4.960585
H 3.029286 3.002824 3.796621
H 2.045968 4.041394 4.825397
H 2.399668 5.052869 2.559199
H 0.694329 4.748738 2.875812
H 2.558698 2.984481 1.199181
H 1.281101 4.033109 0.594055
H 0.842664 3.065306 -1.306162
H -2.113281 2.754870 -0.583591
H -0.979487 3.907665 0.104349
H -2.400847 4.950611 -1.641220
H -0.696184 5.008454 -2.083398
H -2.720779 2.954257 -3.099645
H -2.078289 4.306624 -4.032794
H 0.217424 3.379887 -3.839997
H -0.856107 2.187941 -4.565848
H -1.081730 1.000774 -2.425201
H 0.629073 1.191598 -2.810658

4.5.3.3•AuCl

E = -3055.07208032
Au 2.435137 -0.759504 -0.672448
P 0.463029 -1.182489 0.386499
Cl 4.409964 -0.177106 -1.773506
P -0.014767 1.754041 -0.082263
C -0.667992 0.178360 0.208206
C 1.209972 2.279066 1.168909
C 0.804051 1.999793 -1.683429
C -1.356120 2.977865 0.019980
C -0.368327 -2.639547 -0.398562
C 0.704315 -1.689467 2.164349
F -6.864470 -1.151444 1.207627
C -6.344660 -0.918038 -0.002756
F -7.042735 0.080079 -0.555591
F -6.587458 -2.010001 -0.741150
C -2.111796 -0.074608 0.156833
C -2.929331 0.443240 -0.865658
C -2.739326 -0.880128 1.125500
C -4.291928 0.200208 -0.901780
C -4.885756 -0.601747 0.070415
C -4.098546 -1.147321 1.081278
C 0.510061 1.105747 -2.713356
C 1.701265 3.048046 -1.905781
C 2.309839 3.183804 -3.146403
C 2.023013 2.282327 -4.166959
C 1.117811 1.248963 -3.953825
C 2.578302 2.052685 0.991769
C 0.756602 2.800630 2.384830
C 1.649510 3.054290 3.416976
C 3.005180 2.783944 3.250418
C 3.466984 2.292391 2.035186

C -1.444744 4.019492 -0.903732
C -2.307865 2.880830 1.042275
C -2.468435 4.956133 -0.802322
C -3.407173 4.858498 0.217157
C -3.325058 3.819346 1.139876
C 0.520310 -0.522601 3.127751
C 2.087290 -2.306420 2.386997
C 0.669140 -0.972241 4.574487
C 2.022125 -1.630362 4.808113
C 2.257045 -2.771245 3.827742
C -0.651879 -2.356442 -1.872466
C 0.452208 -3.918340 -0.228545
C -1.293811 -3.550724 -2.565611
C -0.449443 -4.805765 -2.408463
C -0.198038 -5.100547 -0.937995
H -2.474243 1.048391 -1.648813
H -4.901429 0.625981 -1.695740
H -4.560026 -1.763789 1.849612
H -2.141508 -1.286761 1.939873
H 1.937569 3.746801 -1.105207
H 3.023360 3.987763 -3.313117
H 2.517543 2.380665 -5.131011
H 0.901184 0.537376 -4.747402
H -0.170920 0.280163 -2.517190
H -0.300010 3.003345 2.535662
H 1.283134 3.459284 4.358075
H 3.702444 2.969496 4.065166
H 4.525735 2.092608 1.884318
H 2.967570 1.686004 0.044044
H -0.721567 4.097609 -1.711725
H -2.532303 5.762681 -1.529712
H -4.210021 5.589385 0.290919
H -4.064475 3.731011 1.932905
H -2.264542 2.052451 1.746594
H -0.068160 -2.450779 2.369859
H -0.444898 -0.025809 2.962677
H 1.294333 0.225431 2.908043
H 0.539410 -0.113475 5.246318
H -0.133426 -1.685246 4.819376
H 2.811757 -0.875312 4.673395
H 2.104286 -1.987684 5.842766
H 1.542980 -3.583678 4.033853
H 3.259302 -3.196628 3.966233
H 2.277838 -3.127816 1.686929
H 2.840233 -1.537382 2.154491
H -1.328002 -2.770782 0.123025
H 0.299166 -2.103981 -2.369056
H -1.306268 -1.481163 -1.960331
H -2.290335 -3.724682 -2.131986
H -1.453348 -3.320358 -3.626873
H -0.936051 -5.661563 -2.893615
H 0.514651 -4.661523 -2.919478
H 0.433357 -5.990873 -0.822631
H -1.156100 -5.331825 -0.447625

H 1.462417 -3.750244 -0.634874
H 0.573452 -4.155135 0.835337

4.5.4.4•AuCl

E = -2756.88652887
Au 1.714419 -1.299115 -0.499485
P 0.961198 0.790818 0.027926
Cl 2.287253 -3.503786 -1.041777
P -1.667949 -0.638564 0.098178
C -0.805579 0.844854 0.135509
C -1.352612 -1.704110 -1.344572
C -3.453335 -0.277916 0.080133
C -1.355384 -1.719017 1.547588
C 1.759415 1.323422 1.610312
C 1.391675 2.067302 -1.258778
C -1.446508 2.177603 0.302324
C -2.175222 2.829095 -0.717593
C -1.264283 2.862507 1.517633
C -2.644369 4.127773 -0.491752
C -2.493237 2.173961 -2.026397
C -2.426885 4.794367 0.705022
H -3.194095 4.627437 -1.289121
C -1.734992 4.150209 1.725300
H -2.800453 5.807381 0.842357
H -1.566412 4.645871 2.679613
H -0.741065 2.347967 2.319914
C -1.909284 -1.347360 2.777278
C -0.437615 -2.772933 1.508000
H -3.513565 1.765560 -2.019381
H -1.818025 1.340672 -2.233039
H -2.436103 2.891489 -2.853203
C -0.064006 -3.420082 2.682226
H 0.014078 -3.094006 0.571913
C -0.608182 -3.034866 3.900763
H 0.664886 -4.226193 2.630466
C -1.541874 -2.002363 3.944351
H -0.310751 -3.540478 4.817520
H -1.982641 -1.701755 4.892849
H -2.626685 -0.532383 2.831067
C -1.727483 -3.050540 -1.336337
C -0.715584 -1.175574 -2.469203
C -1.437137 -3.860401 -2.426197
H -2.227126 -3.472478 -0.466081
C -0.784889 -3.331067 -3.534649
H -1.708456 -4.913531 -2.403187
C -0.435850 -1.986303 -3.561073
H -0.539314 -3.973201 -4.377788
H 0.082634 -1.571044 -4.422332
H -0.403824 -0.132972 -2.463959
C -4.290177 -0.898067 -0.851804
C -4.001382 0.653246 0.970865
C -5.646360 -0.593959 -0.890922

H -3.882906 -1.612412 -1.561979
C -6.181746 0.333400 -0.005117
H -6.284160 -1.082415 -1.624756
C -5.356169 0.954432 0.926129
H -7.242244 0.575059 -0.041656
H -5.764333 1.687258 1.618878
H -3.367795 1.171291 1.684872
C 3.283686 1.251909 1.517687
C 1.254337 0.437558 2.747454
H 1.459270 2.365824 1.797099
C 1.908656 0.793938 4.073874
H 0.162716 0.501857 2.822673
H 1.474445 -0.613570 2.497938
C 3.426420 0.731024 3.976083
H 1.605123 1.811209 4.367418
H 1.543180 0.119844 4.859798
C 3.938101 1.613008 2.845870
H 3.730502 -0.309295 3.785268
H 3.887375 1.021980 4.928958
H 5.029152 1.530792 2.757379
H 3.722562 2.666854 3.080893
H 3.657995 1.908053 0.722175
H 3.566032 0.226219 1.231924
C 2.580965 1.670580 -2.133428
C 1.547711 3.493475 -0.727675
H 0.489502 2.051709 -1.893055
C 1.737743 4.477532 -1.875679
H 0.679478 3.780042 -0.123560
H 2.425017 3.541430 -0.065995
C 2.921798 4.091488 -2.750511
H 1.863966 5.493470 -1.479474
H 0.820925 4.493760 -2.485141
C 2.780148 2.666953 -3.268654
H 3.028543 4.794895 -3.586548
H 3.846923 4.169552 -2.158649
H 3.655779 2.383844 -3.866904
H 1.911570 2.614720 -3.943615
H 3.491854 1.622383 -1.518242
H 2.432234 0.657460 -2.529689

4.5.5.5•AuCl

E = -2835.64667582
Au 2.255102 0.566528 -0.556513
P 0.121938 1.168660 0.002594
Cl 4.420396 -0.181313 -1.031934
P -0.313249 -1.796161 0.103822
C -0.979554 -0.215532 0.174521
C -1.697991 -2.975798 0.040796
C 0.742032 -2.192923 -1.331165
C 0.734080 -2.232491 1.544945
C 0.257823 2.154005 1.560926
C -0.697617 2.251247 -1.268655

C -2.447533 0.072077 0.231482
C -3.263494 0.037039 -0.923882
C -3.050242 0.494829 1.442701
C -4.346474 1.007353 1.440309
C -2.369971 0.333291 2.771599
C -5.109456 1.087823 0.279131
H -4.779116 1.340832 2.384360
C -4.556905 0.557118 -0.881375
C -6.474724 1.706524 0.281737
H -5.152979 0.543563 -1.794689
C -2.820838 -0.578772 -2.218018
H -3.083344 -0.047359 3.513083
H -1.977039 1.275982 3.172205
H -1.530667 -0.362936 2.701733
H -7.130713 1.238444 -0.460773
H -6.419829 2.776472 0.036883
H -6.952827 1.623112 1.264101
H -2.962174 0.108660 -3.062168
H -3.415772 -1.477790 -2.428189
H -1.775645 -0.882636 -2.191581
C 0.122898 -2.556817 2.760950
C 2.119546 -2.045720 1.511826
C 2.867804 -2.138926 2.681040
H 2.633231 -1.814401 0.580780
C 2.248647 -2.434495 3.889211
H 3.942048 -1.972532 2.634180
C 0.874773 -2.658429 3.923735
H 2.835532 -2.501832 4.803268
H 0.384260 -2.912096 4.861440
H -0.948147 -2.733618 2.807198
C 1.554029 -3.330245 -1.313417
C 0.742889 -1.368745 -2.458414
C 1.547024 -1.671896 -3.549236
H 0.137287 -0.465309 -2.462769
C 2.362991 -2.796075 -3.519152
H 1.555588 -1.010263 -4.412410
C 2.364280 -3.625559 -2.402265
H 3.011595 -3.020556 -4.363242
H 3.008391 -4.501717 -2.373188
H 1.566730 -3.976980 -0.437625
C -1.770190 -3.950406 -0.957711
C -2.741132 -2.874541 0.968614
C -2.861979 -4.810062 -1.019851
H -0.978201 -4.035518 -1.697156
C -3.890836 -4.705476 -0.091471
H -2.907044 -5.562811 -1.804241
C -3.827532 -3.735852 0.903640
H -4.745385 -5.377181 -0.145643
H -4.632397 -3.639779 1.629170
H -2.717971 -2.105961 1.734684
C 0.915403 1.292762 2.640326
C 1.080033 3.428766 1.369406
H -0.764071 2.429288 1.862917
C 1.028486 2.041359 3.959507

H 0.382370 0.345466 2.768351
H 1.924305 1.023447 2.288596
C 1.812789 3.334068 3.783435
H 0.019503 2.271071 4.337917
H 1.504530 1.399171 4.712065
C 1.213158 4.195813 2.680840
H 1.855446 3.894476 4.726512
H 2.851781 3.085996 3.519146
H 1.821294 5.096429 2.525073
H 0.216341 4.543801 2.993436
H 2.078081 3.144826 0.998496
H 0.637726 4.077036 0.604813
C 0.277424 3.010293 -2.168822
C -1.788764 3.181507 -0.732001
H -1.195170 1.495207 -1.896592
C -2.533204 3.842989 -1.884866
H -2.494020 2.639110 -0.093380
H -1.330692 3.961364 -0.106834
C -1.580376 4.597771 -2.800971
H -3.308451 4.515179 -1.494246
H -3.059299 3.062490 -2.457293
C -0.468294 3.690791 -3.310746
H -1.133945 5.434700 -2.241960
H -2.126083 5.043905 -3.642685
H -0.905985 2.915614 -3.959207
H 0.235347 4.256614 -3.934980
H 0.822038 3.762910 -1.580476
H 1.039416 2.323272 -2.559883

4.5.6.[1•AuNCMe]$^+$

E = -2389.69194590
Au -2.072379 0.353388 0.494667
P 0.863141 -1.482100 -0.284381
C 1.097022 0.204619 -0.579143
P -0.079693 1.376657 0.023825
C 2.476925 0.715386 -0.820469
C 2.730788 1.594290 -1.884629
H 1.926767 1.835458 -2.575928
C 3.992073 2.141521 -2.085882
H 4.155242 2.826592 -2.915683
C 2.566506 -1.956611 -2.430149
H 2.307794 -0.951877 -2.751583
C 4.825761 0.906360 -0.204990
H 5.642607 0.625729 0.457191
C 5.047175 1.802210 -1.245059
H 6.036016 2.226442 -1.404642
C 2.069365 -2.455397 -1.223514
C 3.556794 0.380377 0.007312
H 3.391774 -0.284919 0.850705
C 3.395498 -2.746352 -3.216176
H 3.789246 -2.348838 -4.148957
C 3.720545 -4.037574 -2.813920

H 4.372822 -4.651992 -3.431031
C 0.971913 -2.056050 1.451227
C 2.376088 -3.762593 -0.834398
H 1.967618 -4.174208 0.086379
C 3.204375 -4.547235 -1.627191
H 3.445198 -5.561441 -1.316306
C 2.224674 -2.316003 2.018459
H 3.112340 -2.347487 1.389710
C 2.347830 -2.547066 3.382711
H 3.328470 -2.749046 3.808164
C 1.222067 -2.525718 4.200361
H 1.320029 -2.705688 5.268794
C -0.029305 -2.283522 3.644809
H -0.914979 -2.273663 4.276816
C -0.153780 -2.048689 2.280652
H -1.139198 -1.850925 1.862555
C -0.740318 -2.107022 -0.894104
C -1.281724 -3.296570 -0.400717
H -0.791849 -3.828802 0.411634
C -2.446986 -3.814143 -0.956241
H -2.857574 -4.745656 -0.571315
C -3.069792 -3.157918 -2.014210
H -3.973574 -3.572821 -2.457314
C -2.516979 -1.988231 -2.528230
H -2.986719 -1.482982 -3.369617
C -1.352580 -1.468352 -1.975946
H -0.905637 -0.560812 -2.372688
C 0.548729 2.277221 1.518898
H -0.333722 2.813604 1.902213
C 0.985506 1.270411 2.579271
H 0.152848 0.594711 2.813686
H 1.786785 0.643587 2.163666
C 1.486945 1.955708 3.842660
H 0.650846 2.480667 4.329186
H 1.835878 1.197619 4.555761
C 2.591528 2.953769 3.528135
H 2.931078 3.452197 4.444364
H 3.461745 2.416305 3.120865
C 2.117151 3.977880 2.508443
H 2.916668 4.691766 2.275146
H 1.291198 4.564238 2.939439
C 1.644785 3.306002 1.224007
H 1.280694 4.065830 0.521423
H 2.498584 2.814375 0.741312
C -0.393779 2.705786 -1.216980
H 0.580216 3.189137 -1.383874
C -1.369689 3.750769 -0.672891
H -2.321768 3.250818 -0.429270
H -0.997063 4.185261 0.262436
C -1.617255 4.854640 -1.694424
H -0.681122 5.410485 -1.853441
H -2.340051 5.575358 -1.292611
C -2.101657 4.292579 -3.022601
H -3.091174 3.831176 -2.881370

H -2.237732 5.099720 -3.752544
C -1.135633 3.246662 -3.560158
H -0.174939 3.726289 -3.801481
H -1.511309 2.817096 -4.497282
C -0.895210 2.140085 -2.541823
H -0.181208 1.402632 -2.931254
H -1.838612 1.599301 -2.356882
C -4.841459 -1.287367 0.834251
C -6.039411 -2.094254 0.861313
H -6.241591 -2.428165 1.884668
H -6.890033 -1.506423 0.499587
H -5.901453 -2.967872 0.214726
N -3.884551 -0.650922 0.801753

4.5.7.[2•AuNCMe]$^+$

E = -2504.39591860
Au -2.419634 -0.594673 -0.372225
P -0.280556 -1.280534 0.068590
P 0.518130 1.729008 0.199962
C 0.857415 0.042640 0.203636
C -1.199281 2.137876 0.625386
C 0.802282 2.576297 -1.389188
C 1.598127 2.598382 1.378387
C 0.189971 -2.476831 -1.276158
C -0.238718 -2.339588 1.586926
O 6.337270 -1.366571 0.305435
C 5.051126 -0.995950 0.215666
C 7.169259 -1.215463 -0.813698
C 2.312303 -0.295408 0.215178
C 3.114862 -0.108269 -0.914591
C 2.933142 -0.819392 1.360405
C 4.465416 -0.444394 -0.927144
C 4.269406 -1.174435 1.363627
C -1.737447 1.648774 1.821696
C -1.983579 2.946149 -0.200552
C -3.289855 3.255796 0.167634
C -3.818264 2.767518 1.357236
C -3.040141 1.962964 2.186058
C 0.420205 1.903535 -2.551383
C 1.310300 3.874147 -1.474159
C 0.551213 2.517089 -3.790671
C 1.070329 3.806475 -3.873710
C 1.447263 4.482660 -2.717799
C 1.109955 3.143475 2.568279
C 2.969794 2.671581 1.103162
C 3.835554 3.254890 2.018262
C 3.345079 3.780331 3.209401
C 1.982467 3.730668 3.478130
C -0.567414 -1.544676 2.844970
C -1.155843 -3.556236 1.464506
C -0.488703 -2.416262 4.091204
C -1.406597 -3.624130 3.973785

C -1.097737 -4.423685 2.717084
C 0.296262 -1.773577 -2.624982
C 1.427221 -3.333286 -0.990260
C 1.680487 -4.311909 -2.131794
C 1.810246 -3.592399 -3.465711
C 0.570096 -2.759093 -3.753309
H 2.673843 0.321064 -1.812892
H 5.048042 -0.279921 -1.828849
H 4.745967 -1.585053 2.250477
H 2.347688 -0.937829 2.270352
H 7.254163 -0.161294 -1.115563
H 6.804987 -1.801517 -1.670036
H 8.156102 -1.583466 -0.523437
H -1.577392 3.336142 -1.130296
H -3.890256 3.893880 -0.477810
H -4.835354 3.023206 1.648934
H -3.446084 1.582414 3.120966
H -1.128363 1.019501 2.463419
H 0.025192 0.892537 -2.471695
H 0.254383 1.987283 -4.693583
H 1.183158 4.285828 -4.844025
H 1.852152 5.490357 -2.783465
H 1.601302 4.409725 -0.573023
H 3.364718 2.275822 0.170611
H 4.899551 3.299882 1.796503
H 4.025420 4.237804 3.924701
H 1.590756 4.155984 4.399622
H 0.046537 3.127982 2.789741
H 0.804213 -2.685052 1.660228
H -1.590296 -1.145211 2.747343
H 0.109366 -0.683426 2.928590
H 0.548942 -2.756376 4.227597
H -0.739945 -1.823050 4.979493
H -2.451972 -3.280534 3.937436
H -1.320503 -4.260707 4.862787
H -1.791018 -5.267866 2.615439
H -0.090551 -4.858421 2.802046
H -2.188050 -3.202276 1.304840
H -0.893905 -4.159503 0.587326
H -0.681179 -3.149326 -1.323397
H 2.306621 -2.690513 -0.861562
H 1.304319 -3.888209 -0.052504
H 2.582834 -4.898300 -1.918896
H 0.848178 -5.030364 -2.186907
H 2.691370 -2.933145 -3.435995
H 1.984756 -4.311622 -4.275373
H -0.297279 -3.426199 -3.870958
H 0.677259 -2.217716 -4.701936
H 1.113274 -1.042055 -2.575751
H -0.627752 -1.209739 -2.822638
C -5.459420 0.277119 -1.074337
C -6.815746 0.667505 -1.384702
H -7.518483 -0.010724 -0.888627
H -6.994625 1.690482 -1.036550

H -6.975348 0.621696 -2.467433
N -4.380526 -0.033262 -0.827152

4.5.8.[3•AuNCMe]$^+$

E = -2727.25237835
Au 2.366200 -0.806282 -0.454767
P 0.323031 -1.137978 0.534963
P -0.122950 1.794107 -0.028660
C -0.768640 0.207281 0.195891
C 0.956328 2.341955 1.338509
C 0.887219 2.000006 -1.531343
C -1.479780 2.987679 -0.114636
C -0.407902 -2.637077 -0.262113
C 0.476744 -1.572592 2.335772
F -7.021008 -1.162425 0.646640
C -6.390236 -0.982028 -0.515610
F -7.031460 -0.021565 -1.185161
F -6.532386 -2.110851 -1.218528
C -2.206392 -0.070454 0.018951
C -2.917015 0.383791 -1.104674
C -2.915856 -0.825241 0.967650
C -4.267723 0.119337 -1.262728
C -4.945211 -0.639099 -0.311725
C -4.262373 -1.115319 0.803293
C 0.699742 1.089100 -2.573578
C 1.809159 3.040795 -1.675412
C 2.541603 3.158533 -2.851655
C 2.353779 2.244784 -3.886517
C 1.430510 1.212630 -3.749617
C 2.321795 2.042629 1.351565
C 0.382922 2.942108 2.463603
C 1.152611 3.199879 3.590665
C 2.501506 2.858110 3.611878
C 3.086860 2.288741 2.486200
C -1.496744 4.000244 -1.074813
C -2.525139 2.890373 0.812527
C -2.549658 4.909120 -1.103287
C -3.583357 4.811541 -0.179563
C -3.569571 3.802057 0.779221
C 0.165777 -0.406071 3.267489
C 1.858637 -2.137048 2.670629
C 0.203520 -0.856346 4.721902
C 1.556718 -1.459349 5.074124
C 1.925201 -2.590211 4.123719
C -0.568311 -2.424590 -1.766770
C 0.396434 -3.901827 0.039955
C -1.161689 -3.652521 -2.444754
C -0.344144 -4.901166 -2.152227
C -0.206658 -5.117946 -0.653257
H -2.393873 0.960789 -1.866438
H -4.801476 0.494807 -2.132769
H -4.794687 -1.694272 1.554540

H -2.399096 -1.176990 1.860167
H 1.956499 3.757489 -0.869027
H 3.254372 3.973055 -2.964245
H 2.920959 2.347416 -4.810087
H 1.277059 0.502056 -4.559103
H -0.010206 0.273813 -2.441765
H -0.670465 3.207869 2.467808
H 0.693981 3.668024 4.458864
H 3.099389 3.050452 4.500213
H 4.146431 2.041208 2.486226
H 2.797801 1.619477 0.469756
H -0.697852 4.078613 -1.808376
H -2.561716 5.694112 -1.856135
H -4.407203 5.521532 -0.208956
H -4.382013 3.717553 1.497374
H -2.531330 2.085915 1.545214
H -0.280709 -2.359559 2.493624
H -0.799999 0.051278 3.015931
H 0.926963 0.370949 3.119122
H -0.020172 -0.006430 5.378943
H -0.588471 -1.600763 4.894545
H 2.324358 -0.672686 5.010761
H 1.560990 -1.815136 6.111613
H 1.232625 -3.432923 4.269089
H 2.928522 -2.972477 4.349467
H 2.131030 -2.955700 1.993227
H 2.598371 -1.337824 2.498963
H -1.408200 -2.747564 0.181361
H 0.423321 -2.205584 -2.198524
H -1.203291 -1.550694 -1.953327
H -2.191238 -3.795653 -2.084855
H -1.234185 -3.478570 -3.525595
H -0.801535 -5.778795 -2.624734
H 0.657162 -4.794097 -2.597602
H 0.407078 -6.002343 -0.442278
H -1.199205 -5.319079 -0.223441
H 1.434810 -3.757641 -0.302218
H 0.441863 -4.081890 1.120609
C 5.082249 -0.231334 -2.130977
C 6.241330 0.069495 -2.939345
H 7.070981 0.383680 -2.296879
H 6.539826 -0.821057 -3.503110
H 5.999154 0.876665 -3.639217
N 4.157230 -0.470132 -1.490979

4.5.9.[4•AuNCMe]$^+$

E = -2429.07131589
Au 2.087714 -0.621560 -0.076788
P 0.603205 1.124749 0.076991
P -1.546873 -1.036569 -0.041330
C -1.082452 0.614486 0.102173
C -0.519420 -1.898427 -1.268485

C -3.268632 -1.170403 -0.596955
C -1.486629 -2.119102 1.432219
C 0.996816 2.095969 1.601034
C 0.871143 2.259847 -1.369410
C -2.161623 1.610890 0.405489
C -2.947653 2.188897 -0.617744
C -2.446379 1.952128 1.734259
C -3.968807 3.073593 -0.264721
C -2.725218 1.876166 -2.066853
C -4.230803 3.402662 1.059519
H -4.576178 3.512955 -1.054717
C -3.462407 2.838740 2.069183
H -5.034776 4.095705 1.299023
H -3.655973 3.078421 3.112739
H -1.864148 1.486419 2.525225
C -2.489774 -3.052408 1.705917
C -0.373053 -2.044727 2.271633
H -2.795476 0.800908 -2.264010
H -1.732885 2.199566 -2.401480
H -3.467344 2.381604 -2.692455
C -0.262074 -2.885450 3.371470
H 0.410695 -1.321047 2.059180
C -1.269023 -3.807061 3.645072
H 0.608440 -2.816169 4.020848
C -2.379331 -3.889211 2.812089
H -1.188733 -4.460964 4.511036
H -3.167830 -4.608445 3.023400
H -3.360032 -3.129587 1.058588
C -0.052132 -3.198602 -1.069503
C -0.204685 -1.218692 -2.451368
C 0.744971 -3.802527 -2.037566
H -0.301396 -3.734426 -0.156367
C 1.073776 -3.116665 -3.201806
H 1.105201 -4.817471 -1.880742
C 0.591235 -1.826282 -3.412960
H 1.696507 -3.592050 -3.957226
H 0.835262 -1.293948 -4.329892
H -0.584996 -0.209295 -2.600163
C -3.586790 -1.763620 -1.820857
C -4.290610 -0.669000 0.217454
C -4.912642 -1.835134 -2.234607
H -2.803572 -2.170662 -2.456255
C -5.923162 -1.319006 -1.431684
H -5.154388 -2.297734 -3.189000
C -5.611074 -0.740612 -0.204732
H -6.959261 -1.373011 -1.759471
H -6.399604 -0.341261 0.429172
H -4.057550 -0.218706 1.180016
C 2.374215 2.758941 1.561581
C 0.894197 1.216613 2.845383
H 0.220065 2.876590 1.648879
C 1.131944 2.017916 4.117381
H -0.073263 0.705239 2.881694
H 1.660146 0.426092 2.767832

C 2.489450 2.703932 4.084303
H 0.338270 2.773321 4.219228
H 1.050526 1.361003 4.992535
C 2.637966 3.557243 2.834196
H 3.279857 1.937756 4.096138
H 2.635814 3.314177 4.983842
H 3.638912 4.004117 2.788050
H 1.926395 4.395221 2.879411
H 2.472579 3.418411 0.694674
H 3.139404 1.973171 1.447054
C 2.296895 2.278268 -1.923106
C 0.319171 3.673093 -1.166411
H 0.250754 1.767876 -2.132613
C 0.396529 4.468654 -2.464740
H -0.712128 3.636477 -0.796152
H 0.905334 4.192287 -0.396263
C 1.815715 4.499281 -3.013711
H 0.022335 5.486504 -2.300014
H -0.272847 4.009130 -3.208773
C 2.360391 3.091648 -3.210748
H 1.849070 5.056434 -3.957960
H 2.463906 5.043442 -2.309976
H 3.392731 3.124774 -3.580915
H 1.767830 2.580313 -3.985236
H 2.991697 2.703264 -1.186617
H 2.635640 1.249046 -2.108236
C 4.268956 -2.998487 -0.362166
C 5.250007 -4.047679 -0.520363
H 6.118469 -3.661315 -1.064904
H 5.572037 -4.404883 0.463776
H 4.813513 -4.880716 -1.081760
N 3.490369 -2.161890 -0.236995

4.5.10.[5•AuNCMe]$^{+}$

E = -2507.84207199
Au -2.225472 -0.664213 -0.339191
P -0.035831 -1.206036 0.066738
P 0.462741 1.819476 0.079769
C 1.031360 0.198545 0.119976
C 1.805158 2.886785 -0.502269
C -0.931848 2.087708 -1.067692
C -0.128931 2.560287 1.643750
C -0.059964 -2.200261 1.626208
C 0.683913 -2.311533 -1.241098
C 2.502992 -0.100027 0.166520
C 3.275484 -0.178487 -1.016999
C 3.144237 -0.388853 1.394030
C 4.446048 -0.887999 1.399270
C 2.503779 -0.105391 2.722807
C 5.170813 -1.083054 0.226796
H 4.915091 -1.115916 2.356624
C 4.574439 -0.681347 -0.964490

C 6.543777 -1.681783 0.247466
H 5.142119 -0.754813 -1.892437
C 2.795174 0.314149 -2.350699
H 3.259384 0.250505 3.432093
H 2.051500 -0.997323 3.173495
H 1.721648 0.656537 2.643602
H 7.177806 -1.263345 -0.541777
H 6.496386 -2.767047 0.083073
H 7.039409 -1.520482 1.210584
H 2.980809 -0.421004 -3.143409
H 3.335975 1.230965 -2.623130
H 1.733231 0.559046 -2.340583
C 0.479488 3.662658 2.245618
C -1.229909 1.961739 2.264608
C -1.708124 2.447078 3.472910
H -1.718626 1.110264 1.789972
C -1.085826 3.536759 4.078121
H -2.566223 1.974008 3.946267
C 0.002441 4.142858 3.462717
H -1.454569 3.915638 5.029030
H 0.484580 5.000081 3.927795
H 1.320558 4.157379 1.766574
C -1.903254 3.056871 -0.810195
C -0.971668 1.379851 -2.276045
C -1.966994 1.639024 -3.209571
H -0.224132 0.614545 -2.474132
C -2.935239 2.604659 -2.944227
H -1.985234 1.086018 -4.146252
C -2.903293 3.309256 -1.745492
H -3.708970 2.814784 -3.680425
H -3.650672 4.072802 -1.538957
H -1.879044 3.620307 0.119390
C 1.677600 3.621470 -1.683145
C 3.012627 2.916741 0.205271
C 2.754293 4.359259 -2.162283
H 0.743229 3.614655 -2.238946
C 3.959363 4.366886 -1.469437
H 2.649810 4.927207 -3.084129
C 4.085144 3.650941 -0.282064
H 4.802964 4.938293 -1.851137
H 5.023831 3.660928 0.267238
H 3.119646 2.358680 1.132765
C -0.645673 -1.388411 2.778037
C -0.859405 -3.496340 1.477694
H 0.992610 -2.445520 1.838625
C -0.606018 -2.169791 4.083435
H -0.133176 -0.427978 2.877939
H -1.695861 -1.158986 2.529343
C -1.355617 -3.487673 3.952476
H 0.442775 -2.366890 4.354445
H -1.026278 -1.564009 4.896229
C -0.839604 -4.300292 2.773728
H -1.280814 -4.068816 4.879724
H -2.425808 -3.276852 3.803476

H -1.429946 -5.216896 2.650642
H 0.193129 -4.621711 2.975731
H -1.898840 -3.234380 1.218701
H -0.482471 -4.110817 0.654840
C -0.338125 -3.077260 -2.082332
C 1.803900 -3.236167 -0.750050
H 1.147169 -1.567382 -1.904191
C 2.492266 -3.893072 -1.939037
H 2.533612 -2.688085 -0.143736
H 1.382945 -4.020324 -0.106536
C 1.496369 -4.659172 -2.798240
H 3.290347 -4.558193 -1.586492
H 2.982926 -3.112555 -2.541449
C 0.352375 -3.764093 -3.256004
H 1.087639 -5.497010 -2.213032
H 1.998479 -5.105159 -3.665648
H 0.748381 -2.991547 -3.933160
H -0.379376 -4.340244 -3.836229
H -0.854821 -3.825216 -1.464236
H -1.114632 -2.390378 -2.447778
C -5.294644 0.128042 -0.873192
C -6.661349 0.519594 -1.131328
H -7.115811 -0.174477 -1.846581
H -7.234572 0.500983 -0.198150
H -6.681504 1.532415 -1.547702
N -4.207615 -0.183202 -0.668964

4.6. Exemplary Input Files

4.6.1. Input File for Optimization, Frequency Calculation and Single Point Calculation

```
%mem=56000MB
%nprocs=16
%chk=29.chk
#p opt freq pw6b95d3 genecp

title

1 1
 Au                 2.43513700   -0.75950400   -0.67244800
 P                  0.46302900   -1.18248900    0.38649900
 P                 -0.01476700    1.75404100   -0.08226300
 C                 -0.66799200    0.17836000    0.20820600
 C                  1.20997200    2.27906600    1.16890900
 C                  0.80405100    1.99979300   -1.68342900
 C                 -1.35612000    2.97786500    0.01998000
 C                 -0.36832700   -2.63954700   -0.39856200
 C                  0.70431500   -1.68946700    2.16434900
 F                 -6.86447000   -1.15144400    1.20762700
 C                 -6.34466000   -0.91803800   -0.00275600
 F                 -7.04273500    0.08007900   -0.55559100
 F                 -6.58745800   -2.01000100   -0.74115000
```

C	-2.11179600	-0.07460800	0.15683300
C	-2.92933100	0.44324000	-0.86565800
C	-2.73932600	-0.88012800	1.12550000
C	-4.29192800	0.20020800	-0.90178000
C	-4.88575600	-0.60174700	0.07041500
C	-4.09854600	-1.14732100	1.08127800
C	0.51006100	1.10574700	-2.71335600
C	1.70126500	3.04804600	-1.90578100
C	2.30983900	3.18380400	-3.14640300
C	2.02301300	2.28232700	-4.16695900
C	1.11781100	1.24896300	-3.95382500
C	2.57830200	2.05268500	0.99176900
C	0.75660200	2.80063000	2.38483000
C	1.64951000	3.05429000	3.41697600
C	3.00518000	2.78394400	3.25041800
C	3.46698400	2.29239100	2.03518600
C	-1.44474400	4.01949200	-0.90373200
C	-2.30786500	2.88083000	1.04227500
C	-2.46843500	4.95613300	-0.80232200
C	-3.40717300	4.85849800	0.21715700
C	-3.32505800	3.81934600	1.13987600
C	0.52031000	-0.52260100	3.12775100
C	2.08729000	-2.30642000	2.38699700
C	0.66914000	-0.97224100	4.57448700
C	2.02212500	-1.63036200	4.80811300
C	2.25704500	-2.77124500	3.82774200
C	-0.65187900	-2.35644200	-1.87246600
C	0.45220800	-3.91834000	-0.22854500
C	-1.29381100	-3.55072400	-2.56561100
C	-0.44944300	-4.80576500	-2.40846300
C	-0.19803800	-5.10054700	-0.93799500
H	-2.47424300	1.04839100	-1.64881300
H	-4.90142900	0.62598100	-1.69574000
H	-4.56002600	-1.76378900	1.84961200
H	-2.14150800	-1.28676100	1.93987300
H	1.93756900	3.74680100	-1.10520700
H	3.02336000	3.98776300	-3.31311700
H	2.51754300	2.38066500	-5.13101100
H	0.90118400	0.53737600	-4.74740200
H	-0.17092000	0.28016300	-2.51719000
H	-0.30001000	3.00334500	2.53566200
H	1.28313400	3.45928400	4.35807500
H	3.70244400	2.96949600	4.06516600
H	4.52573500	2.09260800	1.88431800
H	2.96757000	1.68600400	0.04404400
H	-0.72156700	4.09760900	-1.71172500
H	-2.53230300	5.76268100	-1.52971200
H	-4.21002100	5.58938500	0.29091900
H	-4.06447500	3.73101100	1.93290500
H	-2.26454200	2.05245100	1.74659400
H	-0.06816000	-2.45077900	2.36985900
H	-0.44489800	-0.02580900	2.96267700
H	1.29433300	0.22543100	2.90804300
H	0.53941000	-0.11347500	5.24631800

H	-0.13342600	-1.68524600	4.81937600
H	2.81175700	-0.87531200	4.67339500
H	2.10428600	-1.98768400	5.84276600
H	1.54298000	-3.58367800	4.03385300
H	3.25930200	-3.19662800	3.96623300
H	2.27783800	-3.12781600	1.68692900
H	2.84023300	-1.53738200	2.15449100
H	-1.32800200	-2.77078200	0.12302500
H	0.29916600	-2.10398100	-2.36905600
H	-1.30626800	-1.48116300	-1.96033100
H	-2.29033500	-3.72468200	-2.13198600
H	-1.45334800	-3.32035800	-3.62687300
H	-0.93605100	-5.66156300	-2.89361500
H	0.51465100	-4.66152300	-2.91947800
H	0.43335700	-5.99087300	-0.82263100
H	-1.15610000	-5.33182500	-0.44762500
H	1.46241700	-3.75024400	-0.63487400
H	0.57345200	-4.15513500	0.83533700
C	5.23578251	0.06643688	-2.23393827
C	6.53832964	0.45057222	-2.96016894
H	7.33898710	-0.16444529	-2.60576648
H	6.41589431	0.30494588	-4.01311840
H	6.76512366	1.47811522	-2.76621057
N	4.21979574	-0.23318869	-1.66747834

```
P C F H N 0
def2svp
****
Au 0
MWB60
****

Au 0
MWB60



--Link1--
%chk=29_SP.chk
%oldchk=29.chk
%nprocs=16
%mem=32000MB
#p pw6b95d3 genecp guess=read geom=allcheck

P C F H N 0
def2tzvp
****
Au 0
MWB60
****

Au 0
MWB60
```

4.6.2. Input File for NBO Analysis

```
%mem=56000MB
%nprocs=16
%chk=29_nbo.chk
#p pw6b95d3 genecp pop=(savenbo,nbo7read) guess=read geom=allcheck

P C F H N 0
def2svp
****
Au 0
MWB60
****

Au 0
MWB60


$nbo bndidx $end
```

5. References

1 W. Voskuil and J. F. Arens, *Recl. Trav. Chim. Pays-Bas*, 1963, **82**, 302.

2 R. Uson, A. Laguna, M. Laguna, D. A. Briggs, H. H. Murray and J. P. Fackler, in *Inorganic syntheses*, ed. H. D. Kaesz, Wiley, New York, 1989, vol. 103, pp. 85–91.

3 T. Guarr, M. E. McGuire and G. McLendon, *J. Am. Chem. Soc.*, 1985, **107**, 5104.

4 I. Rodstein, D. S. Prendes, L. Wickert, M. Paaßen and V. Gessner, *The Journal of organic chemistry*, 2020.

5 Y. Tang, I. Benaissa, M. Huynh, L. Vendier, N. Lugan, S. Bastin, P. Belmont, V. César and V. Michelet, *Angew. Chem., Int. Ed.*, 2019, **58**, 7977.

6 K. Coetzee, C. E. Strasser, S. Cronje and H. G. Raubenheimer, *Z. Naturforsch. B*, 2009, **64**, 1449.

7 D. Armesto, M. J. Ortiz, A. R. Agarrabeitia, M. Martin-Fontecha, N. El-Boulifi, G. Duran-Sampedro and D. Enma, *Org. Lett.*, 2009, **11**, 4148.

8 S. Nomura, K. Endo-Umeda, A. Aoyama, M. Makishima, Y. Hashimoto and M. Ishikawa, *ACS Med. Chem. Lett.*, 2015, **6**, 902.

9 P. Langer, L. Yang, C. R. Pfeiffer, W. Lewis and N. R. Champness, *Dalton Trans.*, 2018, **48**, 58.

10 M. C. Hansen and S. L. Buchwald, *Org. Lett.*, 2000, **2**, 713.

11 A. A. Mikhailine, M. I. Maishan and R. H. Morris, *Org. Lett.*, 2012, **14**, 4638.

12 W. Iali, F. La Paglia, X.-F. Le Goff, D. Sredojević, M. Pfeffer and J.-P. Djukic, *Chemical communications (Cambridge, England)*, 2012, **48**, 10310.

13 E. Kumaran and W. K. Leong, *Organometallics*, 2012, **31**, 1068.

14 C. Schwarz, J. Handelmann, D. M. Baier, A. Ouissa and V. H. Gessner, *Catal. Sci. Technol.*, 2019, **9**, 6808.

15 J. S. M. Samec, A. H. Ell and J.-E. Bäckvall, *Chemistry (Weinheim an der Bergstrasse, Germany)*, 2005, **11**, 2327.

16 W.-J. Zhao, M. Yan, D. Huang and S.-J. Ji, *Tetrahedron*, 2005, **61**, 5585.

17 T. Scherpf, C. Schwarz, L. T. Scharf, J.-A. Zur, A. Helbig and V. H. Gessner, *Angewandte Chemie (International ed. in English)*, 2018, **57**, 12859.

18 X. Zeng, G. D. Frey, R. Kinjo, B. Donnadieu and G. Bertrand, *J. Am. Chem. Soc.*, 2009, **131**, 8690.

19 T. O. Petersen, E. Tausch, J. Schaefer, H. Scherer, P. W. Roesky and I. Krossing, *Chemistry (Weinheim an der Bergstrasse, Germany)*, 2015, **21**, 13696.

20 A. Leyva-Pérez, J. R. Cabrero-Antonino, Á. Cantín and A. Corma, *The Journal of organic chemistry*, 2010, **75**, 7769.

21 L. Li, G. Huang, Z. Chen, W. Liu, X. Wang, Y. Chen, L. Yang, W. Li and Y. Li, *Eur. J. Org. Chem.*, 2012, **2012**, 5564.

22 C. H. M. Amijs, C. Ferrer and A. M. Echavarren, *Chem. Comm.*, 2007, 698.

23 L. Chen, P. Ren and B. P. Carrow, *J. Am. Chem. Soc.*, 2016, **138**, 6392.

24 G. M. Sheldrick, *Acta crystallographica. Section A, Foundations of crystallography*, 2008, **64**, 112.

25 A. Thorn, B. Dittrich and G. M. Sheldrick, *Acta crystallographica. Section A, Foundations of crystallography*, 2012, **68**, 448.

26 G. M. Sheldrick, *Acta crystallographica. Section C, Structural chemistry*, 2015, **71**, 3.

27 R. Dennington, T. A. Keith and J. M. Millam, GaussView, Version 6.0, Semichem Inc., Shawnee Mission, 2016.

28 M. J. Frisch, G. W. Trucks, H. B. Schlegel, G. E. Scuseria, M. A. Robb, J. R. Cheeseman, G. Scalmani, V. Barone, G. A. Petersson, H. Nakatsuji, X. Li, M. Caricato, A. V. Marenich, J. Bloino, B. G. Janesko, R. Gomperts, B. Mennucci, H. P. Hratchian, J. V. Ortiz, A. F. Izmaylov, J. L. Sonnenberg, D. Williams-Young, F. Ding, F. Lipparini, F. Egidi, J. Goings, B. Peng, A. Petrone, T. Henderson, D. Ranasinghe, V. G. Zakrzewski, J. Gao, N. Rega, G. Zheng, W. Liang, M. Hada, M. Ehara, K. Toyota, R. Fukuda, J. Hasegawa, M. Ishida, T. Nakajima, Y. Honda, O. Kitao, H. Nakai, T. Vreven, K. Throssell, J. A. Montgomery, JR., J. E. Peralta, F. Ogliaro, M. J. Bearpark, J. J. Heyd, E. N. Brothers, K. N. Kudin, V. N. Staroverov, T. A. Keith, R. Kobayashi, J. Normand, K. Raghavachari, A. P. Rendell, J. C. Burant, S. S. Iyengar, J. Tomasi, M. Cossi, J. M. Millam, M. Klene, C. Adamo, R. Cammi, J. W. Ochterski, R. L. Martin, K. Morokuma, O. Farkas, J. B. Foresman and D. J. Fox, Gaussian 16, Revision C.01, Gaussian, Inc., Wallingford CT, 2016.

29 a) P. Hohenberg and W. Kohn, *Phys. Rev.*, 1964, **136**, B864-B871; b) W. Kohn and L. J. Sham, *Phys. Rev.*, 1965, **140**, A1133-A1138;

30 Y. Zhao and D. G. Truhlar, *J. Phys. Chem. A*, 2005, **109**, 5656.

31 a) S. Grimme, J. Antony, S. Ehrlich and H. Krieg, *J. Chem. Phys.*, 2010, **132**, 154104; b) S. Grimme, S. Ehrlich and L. Goerigk, *J. Comput. Chem.*, 2011, **32**, 1456; c) D. G. A. Smith, L. A. Burns, K. Patkowski and C. D. Sherrill, *J. Phys. Chem. Lett.*, 2016, **7**, 2197;

32 F. Weigend and R. Ahlrichs, *Phys. Chem. Chem. Phys.*, 2005, **7**, 3297.

33 D. Andrae, U. Huermann, M. Dolg, H. Stoll and H. Preu, *Theoret. Chim. Acta*, 1990, **77**, 123.

34 P. Deglmann and F. Furche, *J. Am. Chem. Soc.*, 2002, **117**, 9535.

35 a) J. P. Foster and F. Weinhold, *J. Am. Chem. Soc.*, 1980, **102**, 7211; b) A. E. Reed and F. Weinhold, *J. Chem. Phys.*, 1983, **78**, 4066; c) A. E. Reed and F. Weinhold, *J. Chem. Phys.*, 1985, **83**, 1736; d) A. E. Reed, R. B. Weinstock and F. Weinhold, *J. Chem. Phys.*, 1985, **83**, 735; e) A. E. Reed, L. A. Curtiss and F. Weinhold, *Chem. Rev.*, 1988, **88**, 899;

36 E. D. Glendening, J, K. Badenhoop, A. E. Reed, J. E. Carpenter, J. A. Bohmann, C. M. Morales, P. Karafiloglou, C. R. Landis, and F. Weinhold, NBO7, Theoretical Chemistry Institute, University of Wisconsin, Madison, 2018.

37 R. F. W. Bader, *Acc. Chem. Res.*, 1985, **18**, 9.

38 T. Lu and F. Chen, *J. Comput. Chem.*, 2012, **33**, 580.
